# Identification of shared diagnostic genes between osteoporosis and Crohn’s disease through integrated transcriptomic analysis and machine learning

**DOI:** 10.3389/fgene.2025.1609915

**Published:** 2025-10-07

**Authors:** Guirong Yi, Peng Zhou, Qinxu Yang, Maosheng Zhao, Qiaoqiao Yang, Shensong Li, Chenpo Dang

**Affiliations:** 1 Department of Gastroenterology, The Second Hospital & Clinical Medical School, Lanzhou University, Lanzhou, Gansu, China; 2 Department of sports medicine, The 940th Hospital of Joint Logistic Support Force of Chinese People’s Liberation Army, Lanzhou, Gansu, China; 3 Department of orthopedics, The 941th Hospital of Joint Logistic Support Force of Chinese People’s Liberation Army, Xining, Qinghai, China

**Keywords:** Osteoporosis, Crohn’s disease, co-diagnosis, weighted gene co-expression network analysis, machine learning

## Abstract

**Introduction:**

Crohn’s disease (CD) is a chronic inflammatory bowel disease. CD-related inflammation can lead to enhanced bone resorption and destruction, thereby increasing the risk of osteoporosis (OP). This study aimed to screen the hub co-diagnostic gene of CD and OP.

**Methods:**

The gene expression profiles of CD and OP were obtained from the GEO database to select differentially expressed genes (DEGs). Module genes were identified by weighted gene co-expression network analysis. Two machine learning algorithms were employed to screen potential shared genes, and nomograms were constructed to assess their clinical predictive value. Receiver operating characteristic curves, calibration curves, and decision curve analysis were used to evaluate the diagnostic performance of the hub genes. Gene set enrichment analysis (GSEA) and immune infiltration analysis were performed to explore the underlying mechanisms of the hub genes in CD and OP. *In vitro* experiments were conducted to validate the bioinformatics results.

**Results:**

The result showed that a total of 8 DEGs and 15 key module genes were found to be related to both CD and OP, from which machine learning screened out 5 potential shared genes. Subsequently, *ABO* was identified as the hub co-diagnostic gene with good diagnostic value. GSEA results showed that *ABO* was involved in the mitochondrial matrix, chromosomal region, and ribosome in both CD and OP. Immune infiltration analysis found that activated CD8 T cell, effector memory CD4 T cell, and immature B cell were all significantly negatively correlated with *ABO* in both diseases. *In vitro* experiments confirmed the downregulation of ABO in CD and OP cell models.

**Discussion:**

Overall, *ABO* was identified as a hub co-diagnostic gene for CD and OP, providing new insights into their co-management.

## 1 Introduction

Osteoporosis (OP) is a common metabolic bone disease characterized by bone loss and increased fracture risk (Zhang et al., 2023). Bone homeostasis disruption due to changes in osteoblast and osteoclast activity plays a key role in the development of OP (Adejuyigbe et al., 2023). Studies have shown that the activities of osteoblasts and osteoclasts are regulated by various factors secreted from immune cells, and the gut microbiota is involved in interactions between the immune system and bone cells (Ponzetti and Rucci, 2019; Locantore et al., 2020). It is reported that the global prevalence of OP is 18.3%, with the elderly being more susceptible (Salari et al., 2021; Hassan et al., 2024; Hadji et al., 2024). The increasing incidence of fractures and deaths related to OP brings a huge burden to society (Shen et al., 2022).

Gastrointestinal diseases have been proven to be an important risk factor for osteoporotic fractures, with inflammatory bowel disease (IBD) being strongly correlated with them (Xu et al., 2023). Crohn’s disease (CD) is a relapsing chronic IBD that involves the entire gastrointestinal tract from the mouth to the anus and is accompanied by parenteral complications and immune disorders, affecting millions of people worldwide (Veauthier and Hornecker, 2018). Its clinical manifestations are the alternation of inflammation (exacerbation) and asymptomatic periods (Pinto et al., 2024). Symptoms including abdominal pain, fever, intestinal obstruction, or diarrhea occur during the exacerbation period (Baumgart and Sandborn, 2012). Immune system disorders, gut microbiota dysbiosis, and genetic and environmental factors influence CD development (Baumgart and Sandborn, 2012). However, the pathogenesis of CD remains unclear, with a lack of recognized diagnostic criteria and management methods (Liu et al., 2024). Considering the potential impact of CD on bone metabolism (Wu et al., 2012), it is essential to explore its relationship with OP to improve patient management.

CD patients have been found to have significant cortical and trabecular bone loss (Haschka et al., 2016). A case of combined CD and OP has been reported with the recovery of vertebral density and structure after treatment of CD (Thearle et al., 2000). A prospective study revealed that 14.6% of CD patients were diagnosed with OP, and the risk of OP was increased in people with CD compared to the normal group (Lo et al., 2020). The above evidence suggests that OP and CD may be involved in common pathological mechanisms. In addition, corticosteroids, a drug for the treatment of CD, can reduce bone density in patients and increase the risk of OP (Dear et al., 2001). Adalimumab, an anti-tumor necrosis factor-α antibody, demonstrates the ability to promote osteonecrosis in CD patients (Preidl et al., 2014). Therefore, screening for shared diagnostic markers of CD and OP is necessary.

In this study, the differentially expressed genes (DEGs) of CD and OP were screened based on the gene expression profile data in the Gene Expression Omnibus (GEO) database, and the co-expression module genes of CD and OP were obtained by weighted gene co-expression network analysis (WGCNA). Gene ontology (GO) and Kyoto Encyclopedia of Genes and Genomes (KEGG) enrichment analysis were used to explore the common biological pathways of these genes. Machine learning was employed to identify potential shared diagnostic genes and their predictive abilities were assessed to identify key genes for co-diagnosis. Single-gene Gene Set Enrichment Analysis (GSEA) was used to explore its biological pathways, and immune infiltration analysis was utilized to identify immune cells closely associated with the hub co-diagnostic gene. *In vitro* experiments were conducted to validate the bioinformatics results.

**FIGURE 1:**
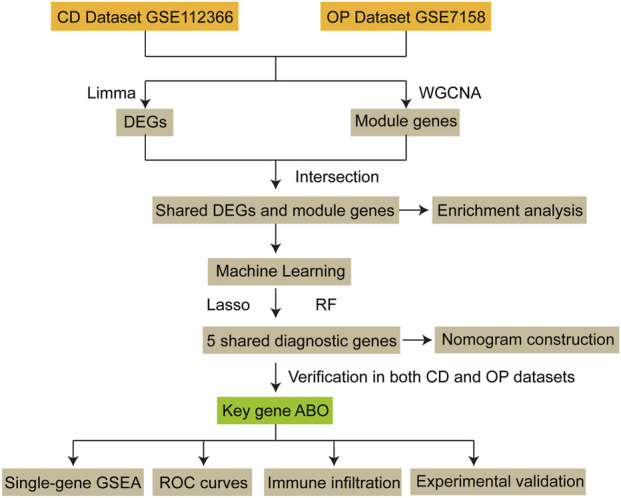
The workflow of this study. CD, Crohn’s disease; OP, osteoporosis; DEG, differentially expressed gene; WGCNA, weighted gene co-expression network analysis; Lasso, Least Absolute Shrinkage and Selection Operator; RF, Random Forest; GSEA, Gene Set Enrichment Analysis; ROC, receiver operating characteristic.

The workflow is shown in Figure 1.

## 2 Materials and methods

### 2.1 Data acquisition

CD and OP gene expression profile data were downloaded from the GEO database (https://www.ncbi.nlm.nih.gov/geo/). GSE112366 contains 362 CD samples and 26 normal controls, while GSE207022 includes 125 CD samples and 23 normal controls. GSE7158 is comprised of 40 high bone mineral density (BMD) samples and 40 low-BMD samples. GSE13850 encompasses 10 high-BMD samples and 10 low-BMD samples. GSE112366 and GSE7158 were used as training sets to identify DEGs and module genes. GSE207022 and GSE13850 were utilized for external validation. The summary of datasets is shown in Table 1.

**TABLE 1:** Details of datasets in this study.

GEO series	Platform	Year	Sample	Attribute
GSE112366	GPL13158	2018	26 controls and 362 cases of CD	Training set
GSE207022	GPL13158	2022	23 controls and 125 cases of CD	Validation set
GSE7158	GPL570	2007	40 low-BMD and 40 high-BMD individuals	Training set
GSE13850	GPL96	2008	10 low-BMD and 10 high-BMD individuals	Validation set

CD, Crohn’s disease.

### 2.2 Identification of DEGs

Limma package (Liu et al., 2021) in R (version 3.58.1) was used to screen CD-related and OP-related DEGs, the threshold was set as P < 0.05 and |log2 Fold Change (FC)| > log2 (1.2). Ggplot2 package (version 3.5.0) and pheatmap package (version 1.0.12) were utilized to draw heatmaps and volcano plots.

### 2.3 WGCNA

WGCNA package in R (version 1.72.5) was used to construct gene co-expression networks for the top 5,000 genes with the most variance. The function “hclust” was carried out to perform hierarchical clustering analysis to remove outliers from the samples. The “pickSoftThreshold” function was applied to estimate the optimal soft threshold automatically. If there was no optimal soft threshold, the first fit index greater than 0.85 was selected. The weighted adjacency matrix was then converted to a topological overlap matrix (TOM). The hierarchical clustering tree was constructed based on the average link hierarchical clustering, and the dynamic tree algorithm (minModuleSize = 30) was used to find different gene modules.

### 2.4 Identification of shared genes and enrichment analysis

The DEGs of CD and OP were intersected, as well as the module genes, to obtain shared genes linked with both diseases. GO and KEGG pathway analysis were performed in the clusterProfiler package (version 4.10.1) to explore potential biological functions and signaling pathways associated with these shared genes. The p-values were corrected by the false discovery rate (FDR).

### 2.5 Machine learning

Two machine learning algorithms, Least Absolute Shrinkage and Selection Operator (LASSO) and Random Forest (RF), were used to select key diagnostic biomarkers in shared genes. To ensure repeatability, the seeds for both disease groups were set to 1,234. The glmnet package in R (version 4.1.8) was applied to construct the LASSO model and the key genes were selected by 10-fold cross-validation. In each cross-validation fold, the model was trained on a series of λ values, and the corresponding average errors were calculated. The λ value that minimized the cross-validation error was selected as the optimal λ. RF algorithm was utilized for significant gene classification using the randomForest package in R (version 4.7.1.1). According to the default settings of this package, the number of decision trees was set to 500, which was derived from extensive research and usually provides good model performance and stability. Mean decrease accuracy was utilized to quantify the importance of the genes. The machine learning results were intersected to identify the overlapping genes between CD and OP.

### 2.6 Nomogram construction

The diagnostic nomograms of CD and OP were established using multivariable logistic regression analysis based on the overlapping genes. The dependent variable of the model was the sample grouping (patients were coded as 1, and the control group was coded as 0), and the independent variable was the expression levels of the screened genes. The model was fitted and visualized using the rms (version 6.3.0) and Hmisc (version 4.7.1) packages. First, the “datadist” function was used to calculate the distribution summaries of all variables. Subsequently, the “lrm” function was utilized to fit the full-variable logistic regression model, based on which the nomogram was generated using the “nomogram” function. The performance of the model was evaluated through receiver operating characteristic (ROC) curves, calibration curves, and decision curve analysis (DCA).

### 2.7 Identification of the shared hub diagnostic gene

Gene expression levels were examined in the training and validation datasets to assess the predictive abilities of these shared diagnostic genes. ROC curves, calibration curves, and DCA were used to evaluate the diagnostic value of the shared hub diagnostic gene.

### 2.8 Single-gene GSEA

To explore the functions of the shared hub diagnostic gene, single-gene GSEA was performed in clusterProfiler package (version 4.10.1). Genes in the expression profile were sorted according to their correlation coefficients with the hub gene, and then GSEA analysis was conducted based on the GO and KEGG pathways.

### 2.9 Immune infiltration analysis

To quantify the relative enrichment of 28 immune cells in each sample, single-sample GSEA (ssGSEA) was conducted using the “gsva” function from the GSVA package, and an enrichment score matrix of “sample × immune cell type” was generated. This matrix was standardized by Z-score to eliminate the interference of expression level differences among samples on visualization. Boxplots were drawn using the ggplot2 package to show the distribution of enrichment scores in different disease groups.

### 2.10 Cell culture

HT-29 and RAW264.7 cells were purchased from Wuhan Saio Biotechnology Co., Ltd. and grown in RPMI-1640 medium (Gibco, C11875500BT) and DMEM medium (Gibco, C11885500BT), respectively, at 37 °C in 5% CO_2_. All media were supplemented with 10% FBS (Solarbio, S9030). To verify the role of *ABO* in CD, HT-29 cells were divided into the Control and LPS groups. Cells were treated with 10 μg/mL LPS (Beyotime, S1732) for 24 h. To assess the role of *ABO* in OP, RAW264.7 cells were divided into the Control and RANKL groups. Cells were cultured in a medium containing 50 ng/mL RANKL (MCE, HY-P73388) for 5 days to induce osteoclast differentiation.

### 2.11 ELISA

After LPS treatment, the supernatant of HT-29 cells was collected. The levels of IL-1β and IL-18 in the supernatant were detected using ELISA kits (Beyotime), following the manufacturer’s instructions. Absorbance at 450 nm was measured using a microplate reader (Wuxi Hiwell Diatek, DR-3518G).

### 2.12 RT-qPCR

*ABO* levels in HT-29 cells, as well as *CTSK*, *MMP9*, and *ABO* levels in RAW264.7 cells, were detected by RT-qPCR. Total RNA was extracted from cells using Trizol (Invitrogen, 15596018), and then reverse-transcribed into cDNA using the cDNA first-strand synthesis kit (TIANGEN, KR118-02). PCR reactions were conducted using the SYBR Green-based detection system on a real-time quantitative fluorescence PCR instrument (Bio-Rad, CFX96 Touch). The mRNA levels were calculated using the 2^−ΔΔCT^ method and normalized to *ACTB*. Primer sequences are listed in Table 2.

**TABLE 2:** Primer sequences used for RT-qPCR.

Gene	Forward primer (5′-3′)	Reverse primer (5′-3′)
*ABO* (Human)	ACC​AAA​ATG​CCA​CGC​ACT​TC	TTG​TTC​AGG​TGG​CTC​TCG​TC
*ACTB* (Human)	AGA​CCT​GTA​CGC​CAA​CAC​AG	TTC​TGC​ATC​CTG​TCG​GCA​AT
*CTSK* (mouse)	TAC​CCA​TAT​GTG​GGC​CAG​GA	TTC​AGG​GCT​TTC​TCG​TTC​CC
*MMP9* (mouse)	CAG​CCA​GAC​ACT​AAA​GGC​CA	ACA​ACT​CGT​CGT​CGT​CGA​AA
*ABO* (mouse)	AGC​CGA​GAG​GCC​TTT​ACC​TA	ATT​GCC​TGA​TGG​TCC​TTG​GG
*ACTB* (mouse)	AGG​GAA​ATC​GTG​CGT​GAC​AT	GGA​AAA​GAG​CCT​CAG​GGC​AT

### 2.13 Western blot

Total proteins were extracted from HT-29 and RAW264.7 cells using RIPA buffer (Beyotime, P0013B). Protein samples were separated through SDS-PAGE gels and then transferred onto PVDF membranes (Beyotime, FFP24). The membranes were blocked with 5% skimmed milk (Beyotime, P0216) for 1 h, followed by incubation with the ABO primary antibody (1:500; Affinity, DF6481) at 4 °C overnight. Goat anti-rabbit IgG H&L/HRP (1:2000; Abcam, ab6721) was applied to the membranes for 1 h. Protein bands were visualized using enhanced chemiluminescence (Servicebio, G2019).

### 2.14 TRAP staining

TRAP staining was used to assess the osteoclast formation of RAW264.7 cells using a TRAP staining kit (Solarbio, G1492). Cells were fixed with TRAP fixation solution for 30 s-3 min. Subsequently, cells were incubated with TRAP incubation solution at 37 °C for 45–60 min, and then stained with methyl green staining solution for 2–3 min. Finally, cells were observed under a microscope.

### 2.15 Statistical analysis

All statistical analyses were performed using R language (v4.3.3) or GraphPad Prism 7.0. The Wilcoxon rank sum test and unpaired t-tests were used to compare the differences between groups. Spearman correlation analysis was employed to calculate the correlation between groups. *P* < 0.05 was considered statistically significant.

## 3 Results

### 3.1 Identification of CD-related and OP-related DEGs

**FIGURE 2:**
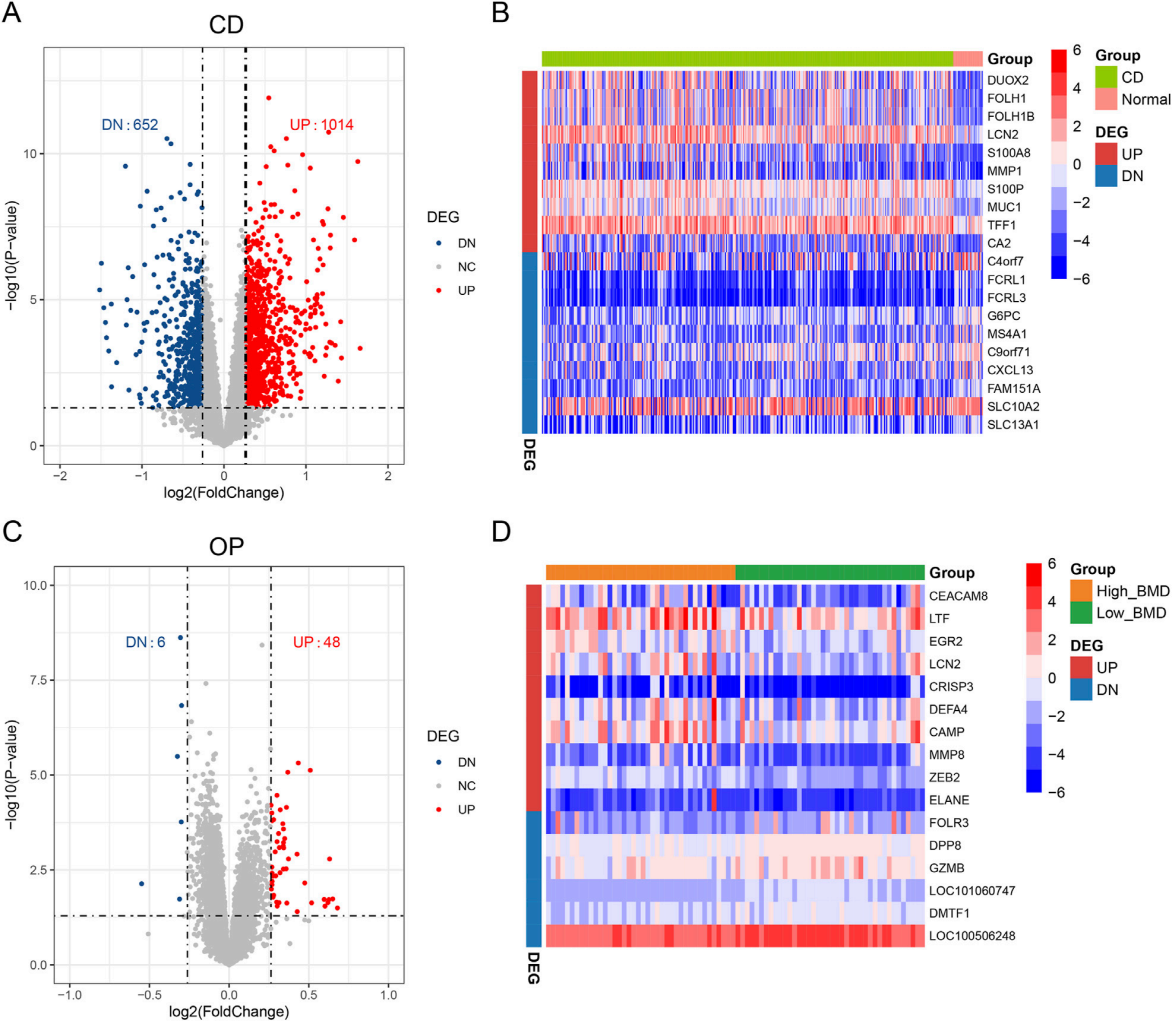
Identification of DEGs in CD and OP. **(A)** Volcano plot of DEGs in CD. **(B)** Heatmap of the top 10 upregulated and top 10 downregulated DEGs in CD. **(C)** Volcano plot of DEGs in OP. **(D)** Heatmap of the top 10 upregulated and top 6 downregulated DEGs in OP. Blue represents low expression, and red represents high expression.

The limma package was used to analyze the differential expression of genes to identify DEGs. CD obtained 1,666 DEGs with 1,014 upregulated genes and 652 downregulated genes (Figure 2A). Figure 2B presents the top 10 upregulated and top 10 downregulated DEGs for CD. A total of 54 OP-related DEGs were screened, consisting of 48 upregulated genes and 6 downregulated genes (Figure 2C). Figure 2D shows the top 10 upregulated and top 6 downregulated DEGs for OP.

### 3.2 Identification of CD-related and OP-related module genes by WGCNA

**FIGURE 3:**
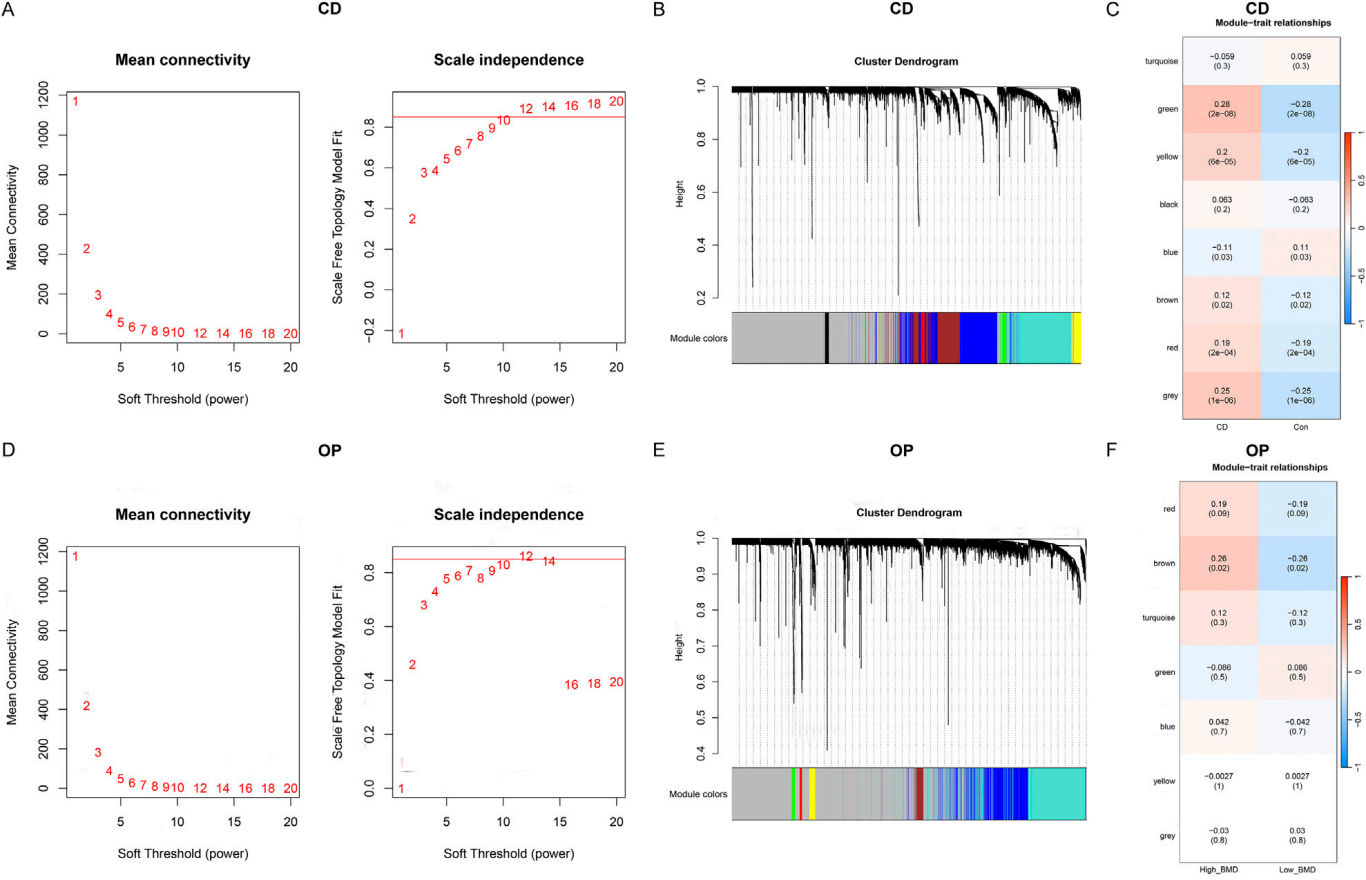
WGCNA of CD and OP. **(A)** Determination of the optimal soft threshold for CD. **(B)** Cluster dendrogram for CD, different colors represent different modules. **(C)** Relationships between modules and traits in CD. **(D)** Determination of the optimal soft threshold for OP. **(E)** Cluster dendrogram for OP. **(F)** Relationships between modules and traits in OP.

WGCNA was conducted to screen CD-related and OP-related module genes. The soft threshold of the CD group was set to 12, and the hierarchical clustering was constructed (Figures 3A,B). Eight gene modules with significant differences were identified, of which five (green, yellow, brown, red, and grey) were positively associated with CD, and one (blue) was negatively associated (Figure 3C). In particular, the green module, containing 81 genes, had the strongest positive correlation with CD. For the OP group, the soft threshold was determined as 12 (Figure 3D). The hierarchical clustering results are shown in Figure 3E. A single gene module (brown) containing 118 genes was identified, which was significantly associated with OP. (Figure 3F).

### 3.3 Enrichment analysis of shared genes between CD and OP

**FIGURE 4:**
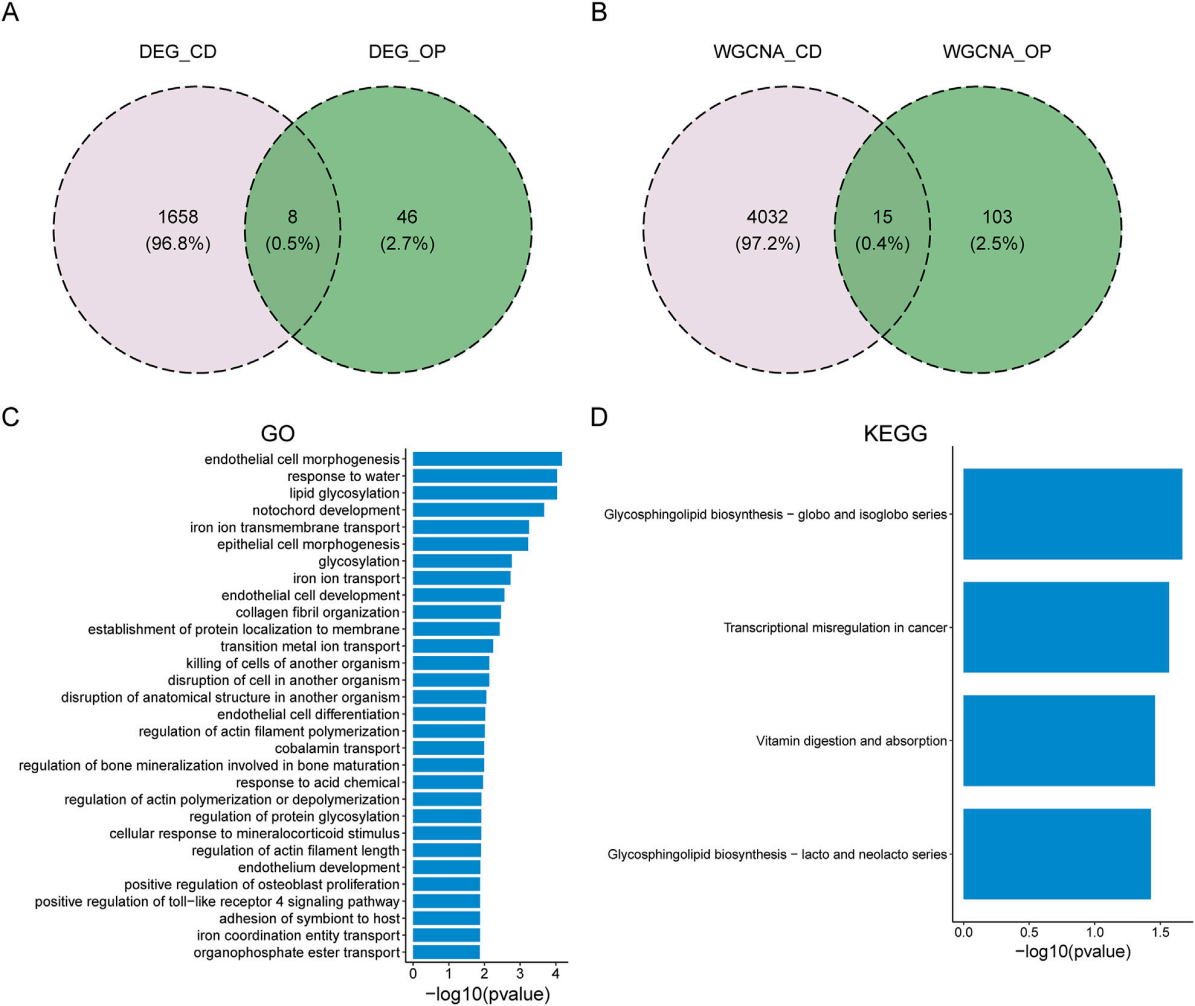
Identification of the overlapping genes between CD and OP and enrichment analysis. **(A)** Overlapping DEGs between CD and OP. **(B)** Overlapping module genes between CD and OP. **(C)** GO enrichment analysis for the 23 overlapping genes. **(D)** KEGG enrichment analysis for the 23 overlapping genes.

To study the common pathogenesis of CD and OP, the DEGs and module genes were intersected, respectively. There were eight overlapping DEGs (*LCN2*, *TPM4*, *DMTF1*, *GZMB*, *GRINA*, *LTF*, *FAM129A*, and *ABO*) between CD and OP (Figure 4A). A total of 15 overlapping module genes (*ALS2CL*, *AMN*, *GJB1*, *ATP8B2*, *IGLV4-60*, *COL18A1*, *ZDHHC11*, *SCNN1A*, *TLX1*, *CCL24*, *RAMP1*, *NPDC1*, *CGB*, *KRT15*, and *KANK3*) were screened (Figure 4B). These 23 shared genes might be involved in the pathogenesis of CD and OP. Subsequently, enrichment analysis was performed to reveal common biological changes between these two diseases. GO enrichment analysis demonstrated that endothelial cell morphogenesis, lipid glycosylation, and other pathways were significantly enriched (Figure 4C). KEGG enrichment results indicated that shared genes mainly participated in glycosphingolipid biosynthesis-globo and isoglobo series, vitamin digestion and absorption, and the glycosphingolipid biosynthesis-lacto and neolacto series (Figure 4D).

### 3.4 Identification of potential diagnostic genes by machine learning

**FIGURE 5:**
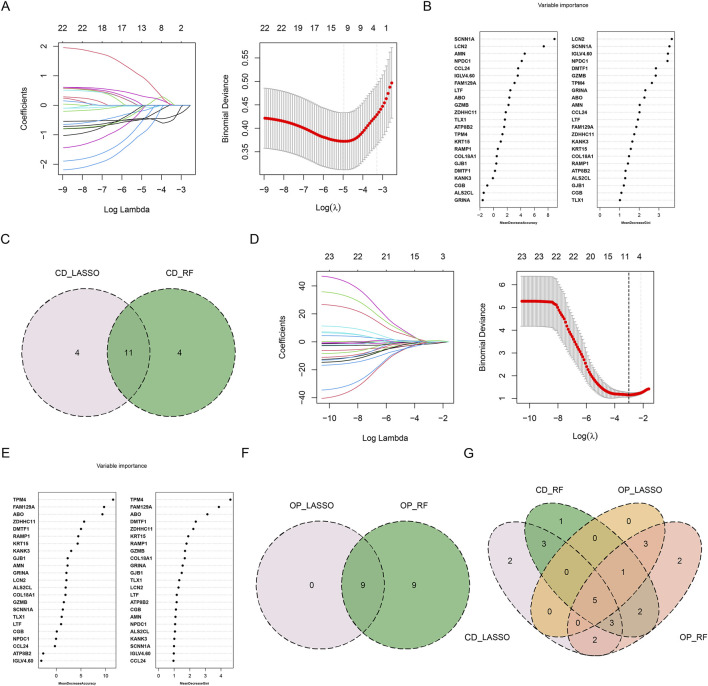
Identification of shared diagnostic genes by machine learning. **(A)** LASSO regression analysis for CD. **(B)** Gene importance ranking for CD by RF algorithm. **(C)** Venn diagrams. The intersection of the two machine learning results of CD obtained 11 shared diagnostic genes. **(D)** LASSO regression analysis for OP. **(E)** Gene importance ranking for OP by RF algorithm. **(F)** Venn diagrams. The intersection of the two machine learning results of OP obtained nine shared diagnostic genes. **(G)** The Venn diagrams showing five shared diagnostic genes identified after the intersection of machine learning results for CD and OP.

Two machine learning algorithms were further used to screen potential diagnostic genes with significant characteristic values. In CD group, λ was set to 0.00703 based on the LASSO coefficient profiles and the optimal tuning parameter selection map, and 15 non-zero coefficient genes were obtained (Figure 5A). The 23 shared genes were input into the RF classifier, and genes was ranked by their importance. Finally, 15 genes were identified (Figure 5B). The potential diagnostic genes for CD identified by LASSO and RF are listed in Table 3. To enhance biomarker credibility and reduce the influence of noise, we intersected the two machine learning results and screened 11 shared genes (*LCN2*, *GZMB*, *LTF*, *FAM129A*, *ABO*, *AMN*, *ATP8B2*, *SCNN1A*, *CCL24*, *NPDC1*, and *KRT15*) for CD diagnosis (Figure 5C). The λ was set to 0.0497, and 9 genes were obtained by the LASSO algorithm in OP group (Figure 5D). Similarly, the RF algorithm selected 18 genes (Figure 5E). The potential diagnostic genes for OP identified by LASSO and RF are listed in Table 4. The intersection of these two algorithms obtained 9 potential diagnostic genes for OP (*TPM4*, *DMTF1*, *GZMB*, *LTF*, *FAM129A*, *ABO*, *ALS2CL*, *COL18A1*, and *KRT15*) (Figure 5F). The machine learning results of CD and OP were intersected, and five overlapping diagnostic genes (*GZMB*, *LTF*, *FAM129A*, *ABO*, and *KRT15*) were obtained (Figure 5G).

**TABLE 3:** Potential diagnostic genes for CD identified by machine learning.

LASSO	RF
*LCN2*	LCN2
*GZMB*	*TPM4*
*GRINA*	*GZMB*
*LTF*	*LTF*
*FAM129A*	*FAM129A*
*ABO*	*ABO*
*AMN*	*AMN*
*ATP8B2*	*ATP8B2*
*IGLV4-60*	*IGLV4.60*
*SCNN1A*	*ZDHHC11*
*CCL24*	*SCNN1A*
*RAMP1*	*TLX1*
*NPDC1*	*CCL24*
*CGB*	*NPDC1*
*KRT15*	*KRT15*

LASSO, least absolute shrinkage and selection operator; RF, random forest.

**TABLE 4:** Potential diagnostic genes for OP identified by machine learning.

LASSO	RF
*TPM4*	*LNC2*
*DMTF1*	*TPM4*
*GZMB*	*DMTF1*
*LTF*	*GZMB*
*FAM129A*	*GRINA*
*ABO*	*LTF*
*ALS2CL*	*FAM129A*
*COL18A1*	*ABO*
*KRT15*	*ALS2CL*
	*AMN*
	*GJB1*
	*COL18A1*
	*ZDHHC11*
	*SCNN1A*
	*TLX1*
	*RAMP1*
	*KRT15*
	*KANK3*

OP, osteoporosis.

### 3.5 Nomogram construction and validation

**FIGURE 6:**
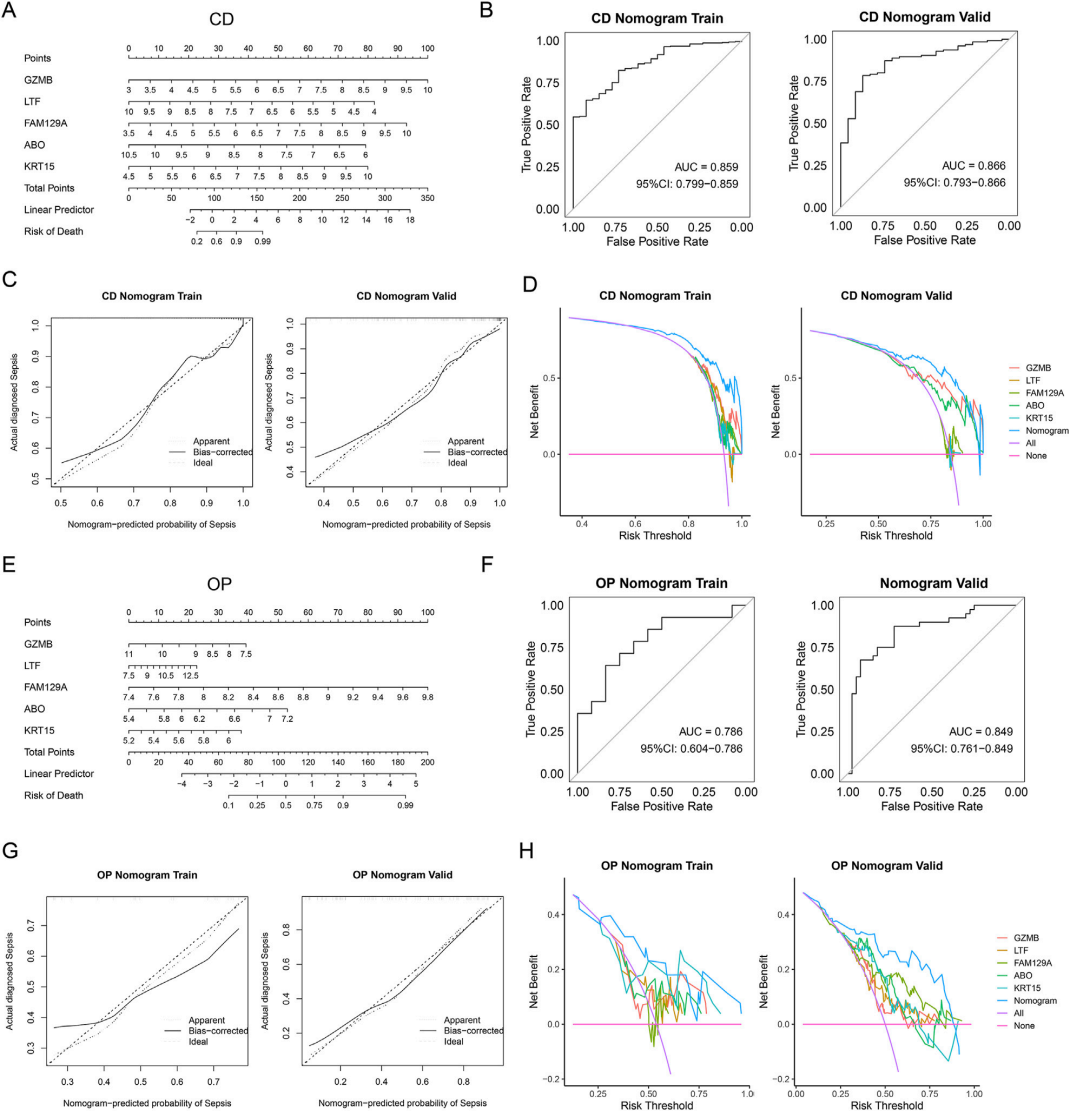
Construction of nomogram for CD and OP patients. **(A)** Nomogram for CD patients based on *GZMB*, *LTF*, *FAM129A*, *ABO*, and *KRT15*. **(B)** ROC curves of CD nomogram in training and validation datasets. **(C)** Calibration curves of CD nomogram in training and validation datasets. **(D)** DCA of the CD nomogram to training and validation datasets. **(E)** Nomogram for OP patients based on *GZMB*, *LTF*, *FAM129A*, *ABO*, and *KRT15*. **(F)** ROC curves of OP nomogram in training and validation datasets. **(G)** Calibration curves of OP nomogram in training and validation datasets. **(H)** DCA of the OP nomogram to training and validation datasets. DCA, decision curve analysis.

We developed a nomogram prediction model for CD patients based on *GZMB*, *LTF*, *FAM129A*, *ABO*, and *KRT15* (Figure 6A). ROC curves of the nomogram in the training and validation datasets showed area under the curve (AUC) values of 0.859 and 0.866 respectively (Figure 6B). Calibration curves of training and validation datasets demonstrated a good consistency between observed and predicted results (Figure 6C). Moreover, DCA demonstrated better clinical efficacy compared to the baselines (Figure 6D). A nomogram for OP patients based on these five genes was also constructed (Figure 6E). ROC curves of the nomogram in the training and validation datasets respectively showed an AUC of 0.786 and 0.849 (Figure 6F). Calibration curves confirmed the robust performance of nomogram in both the training and validation datasets (Figure 6G). DCA indicated that the nomogram can provide support for OP identification (Figure 6H).

### 3.6 Identification and validation of the hub diagnostic biomarker

**FIGURE 7:**
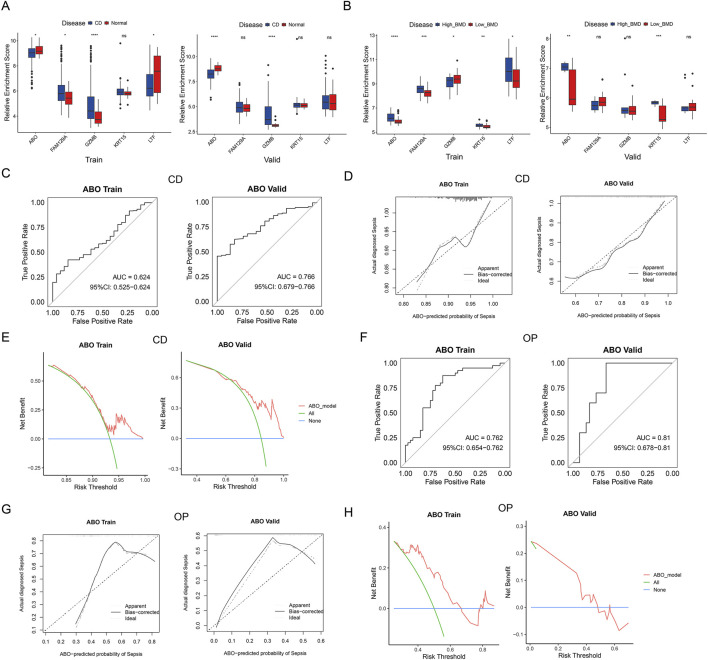
Selection and validation of the shared hub diagnostic gene. **(A)** Expression of shared diagnostic genes in CD training and validation datasets. **(B)** Expression of shared diagnostic genes in OP training and validation datasets. **(C)** ROC curves, **(D)** Calibration curves, and **(E)** DCA of ABO model in CD training and validation datasets. **(F)** ROC curves, **(G)** Calibration curves, and **(H)** DCA of ABO model in OP training and validation datasets.

Subsequently, the expression patterns of five shared diagnostic genes were analyzed to evaluate their predictive abilities. Based on the training set, *GZMB*, *LTF*, *FAM129A*, and *ABO* showed significant differences between CD patients and controls, while only *ABO* and *GZMB* displayed differential expression in the validation set, specifically, *ABO* was consistently low expression in CD patients and *GZMB* showed the opposite trend (Figure 7A). All five genes showed significant differences between high-BMD and low-BMD individuals in the training set, but only *ABO* and *KRT15* presented differential expression in the validation set, as shown in Figure 7B. *ABO* and *KRT15* were always lowly expressed in low-BMD samples. Since *ABO* was significantly differentially expressed in both the training and validation sets, with a consistent trend in CD and OP, it was identified as the hub diagnostic biomarker for both diseases. In addition, the expression of *ABO* was decreased in the two diseases.

ROC curves, calibration curves, and DCA demonstrated the good diagnostic performance of ABO in both CD training and validation sets (Figures 7C–E). The AUC of ABO in OP training and validation sets was 0.76 and 0.81, respectively, both exceeding 0.75 (Figure 7F). Calibration curve of the ABO model showed excellent consistency between observed and predicted results (Figure 7G). DCA showed that the ABO model demonstrates substantial net benefit at most of the threshold probabilities (Figure 7H). The results validated the diagnostic ability of ABO as the hub diagnostic biomarker for CD and OP.

### 3.7 Single-gene GSEA for the hub diagnostic biomarker

**FIGURE 8:**
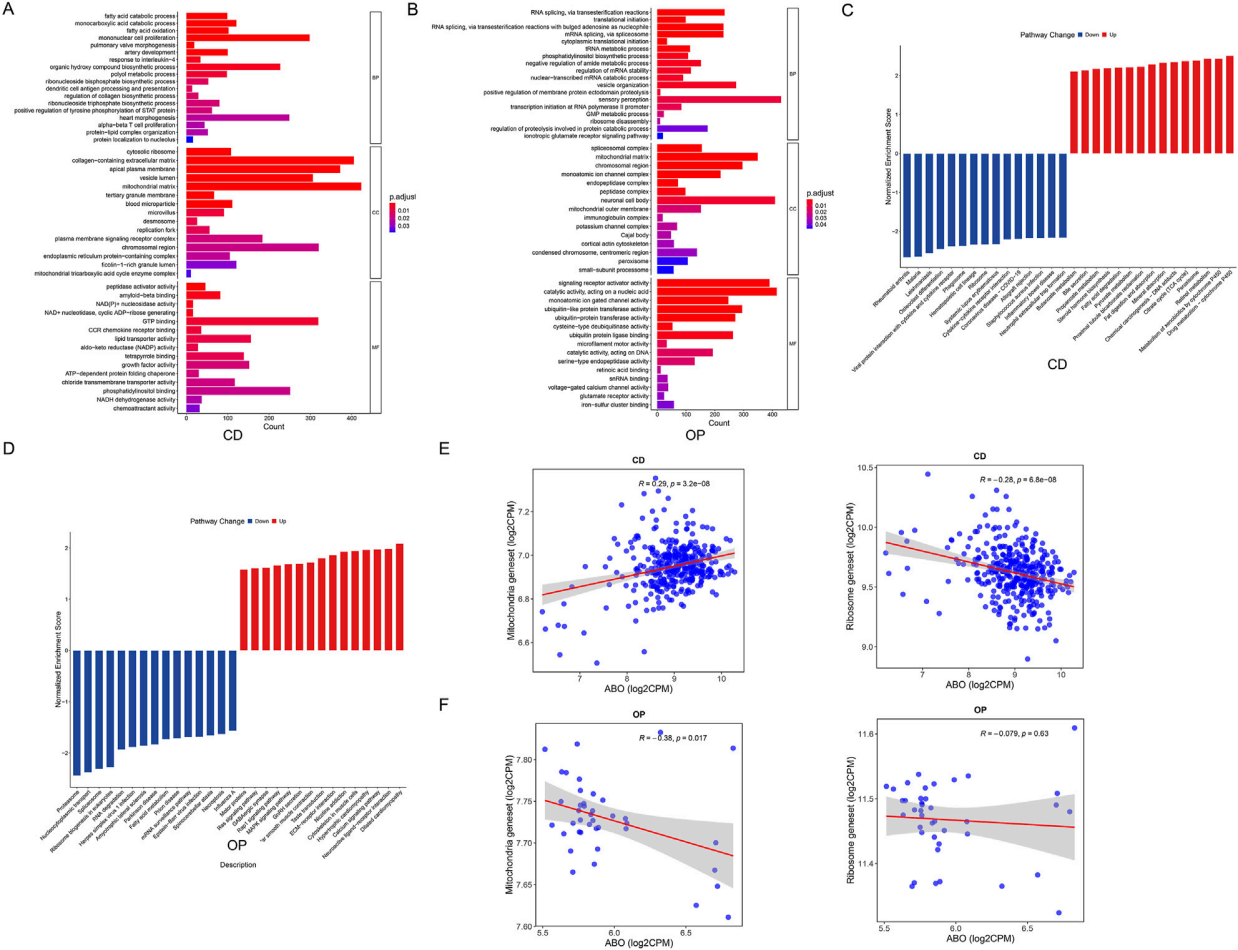
Single-gene GSEA for *ABO*. **(A)** Single-gene GSEA for *ABO* in CD based on GO terms. **(B)** Single-gene GSEA for *ABO* in OP based on GO terms. **(C)** Single-gene GSEA for *ABO* in CD based on KEGG pathway. **(D)** Single-gene GSEA for *ABO* in OP based on KEGG pathway. **(E)** Correlation analysis of ABO and mitochondrial and ribosomal pathways in CD. **(F)** Correlation analysis of ABO and mitochondrial and ribosomal pathways in OP.

Single-gene GSEA was performed based on the training sets of CD and OP. Enrichment analysis based on GO terms revealed that *ABO* was mostly enriched in mononuclear cell proliferation, collagen-containing extracellular matrix, apical plasma membrane, mitochondrial matrix, chromosomal region, and GTP binding in CD (Figure 8A). In OP, *ABO* was mainly enriched in sensory perception, mitochondrial matrix, chromosomal region, neuronal cell body, signaling receptor activator activity, and catalytic activity acting on a nucleic acid (Figure 8B). *ABO* was involved in mitochondrial matrix and chromosomal region in the two diseases. In the KEGG pathways, pathways involved in digestion were upregulated by *ABO* in CD, such as fatty acid degradation, pyruvate metabolism, fat digestion and absorption, and mineral absorption, while osteoclast differentiation, phagosome, ribosome, cytokine-cytokine receptor interaction, and inflammatory bowel disease were downregulated (Figure 8C). In OP, neuroactive ligand-receptor interaction, calcium signaling pathway, and cytoskeleton in muscle cells were upregulated, while proteasome, ribosome biogenesis in eukaryotes, and fatty acid metabolism were downregulated (Figure 8D). The ribosome was downregulated by *ABO* in both diseases. Besides, fatty acid degradation was activated in CD, while its metabolism was inhibited in OP, suggesting that the regulation of *ABO* in CD and OP might not be directly related to fatty acid metabolism/degradation. We further analyzed the correlation of *ABO* with the mitochondrial and ribosomal pathways in CD and OP. The results showed that *ABO* was significantly positively correlated with the mitochondrial pathway, whereas it was markedly negatively correlated with the ribosomal pathway in CD (Figure 8E). Furthermore, *ABO* was significantly negatively correlated with the mitochondrial pathway, and was not notably correlated with the ribosomal pathway in OP (Figure 8F). This evidence suggests that mitochondria may play an important role in the common pathological mechanism of CD and OP.

### 3.8 Immune infiltration analysis for the hub diagnostic biomarker

**FIGURE 9:**
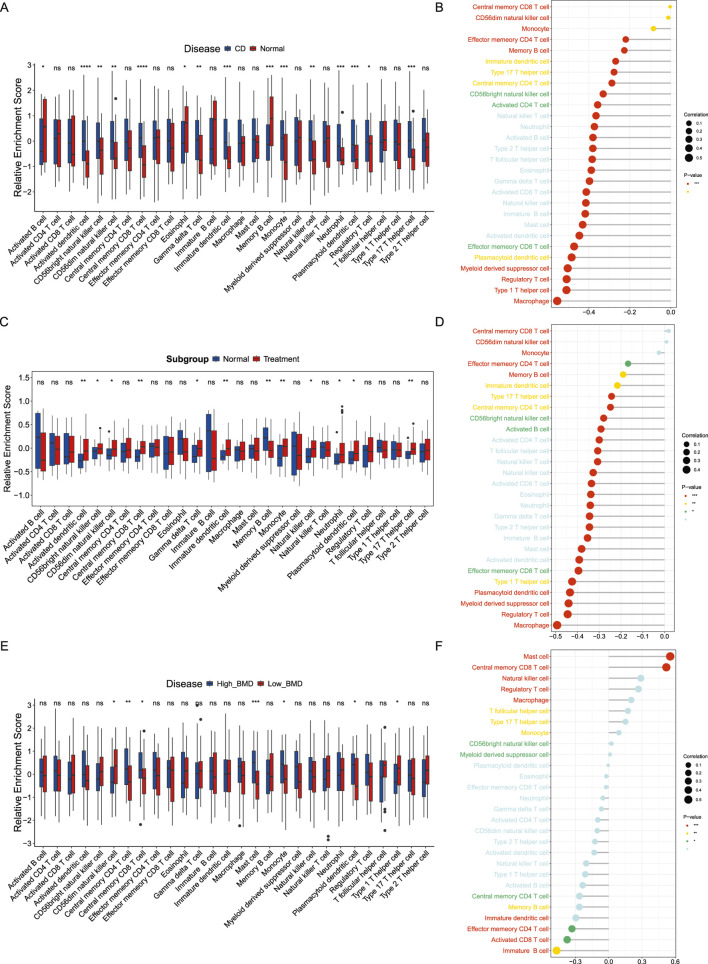
Immune infiltration analysis **(A)** Infiltrating abundance of immune cells between CD patients and healthy controls. **(B)** Immune infiltration analysis of *ABO* in CD. **(C)** Infiltrating abundance of immune cells between hormone-treated and untreated CD patients. **(D)** Immune infiltration analysis of *ABO* in hormone-treated CD patients. **(E)** Infiltrating abundance of immune cells between high-BMD and low-BMD individuals. **(F)** Immune infiltration analysis of *ABO* in low-BMD individuals.

Since the immune system has been proven to influence the progression of CD and OP, the abundances of immune cell infiltration were analyzed. A total of 15 immune cells, including activated B cell, activated dendritic cell, CD56bright natural killer cell, CD56dim natural killer cell, central memory CD8 T cell, eosinophil, gamma delta T cell, immature dendritic cell, memory B cell, monocyte, natural killer cell, neutrophil, plasmacytoid dendritic cell, regulatory T cell, and type 17 T helper cell, showed significant differences in CD group (Figure 9A). Correlation analysis indicated that the expression of *ABO* was negatively correlated with most immune cells in CD patients (Figure 9B). To analyze the role of ABO in CD progression, we conducted a subgroup analysis based on whether CD patients received hormone therapy. A total of 12 immune cells, including activated dendritic cell, CD56bright natural killer cell, CD56dim natural killer cell, central memory CD8 T cell, gamma delta T cell, immature dendritic cell, memory B cell, monocyte, natural killer cell, neutrophil, plasmacytoid dendritic cell, and type 17 T helper cell, demonstrated notable differences between treated and untreated CD groups (Figure 9C). *ABO* expression was negatively correlated with most immune cells in CD patients treated with hormones (Figure 9D).

There were significant differences in seven immune cells (CD56dim natural killer cell, central memory CD4 T cell, central memory CD8 T cell, mast cell, monocyte, plasmacytoid dendritic cell, and type 1 T helper cell) between high-BMD and low-BMD individuals (Figure 9E). A total of 5 immune cells were significantly correlated with the expression of *ABO* (Figure 9F). Specifically, mast cell and central memory CD8 T cell were significantly positively correlated, while effector memory CD4 T cell, activated CD8 T cell, and immature B cell were significantly negatively correlated with *ABO*.

### 3.9 Validation of the role of *ABO* in CD and OP *in vitro*

**FIGURE 10:**
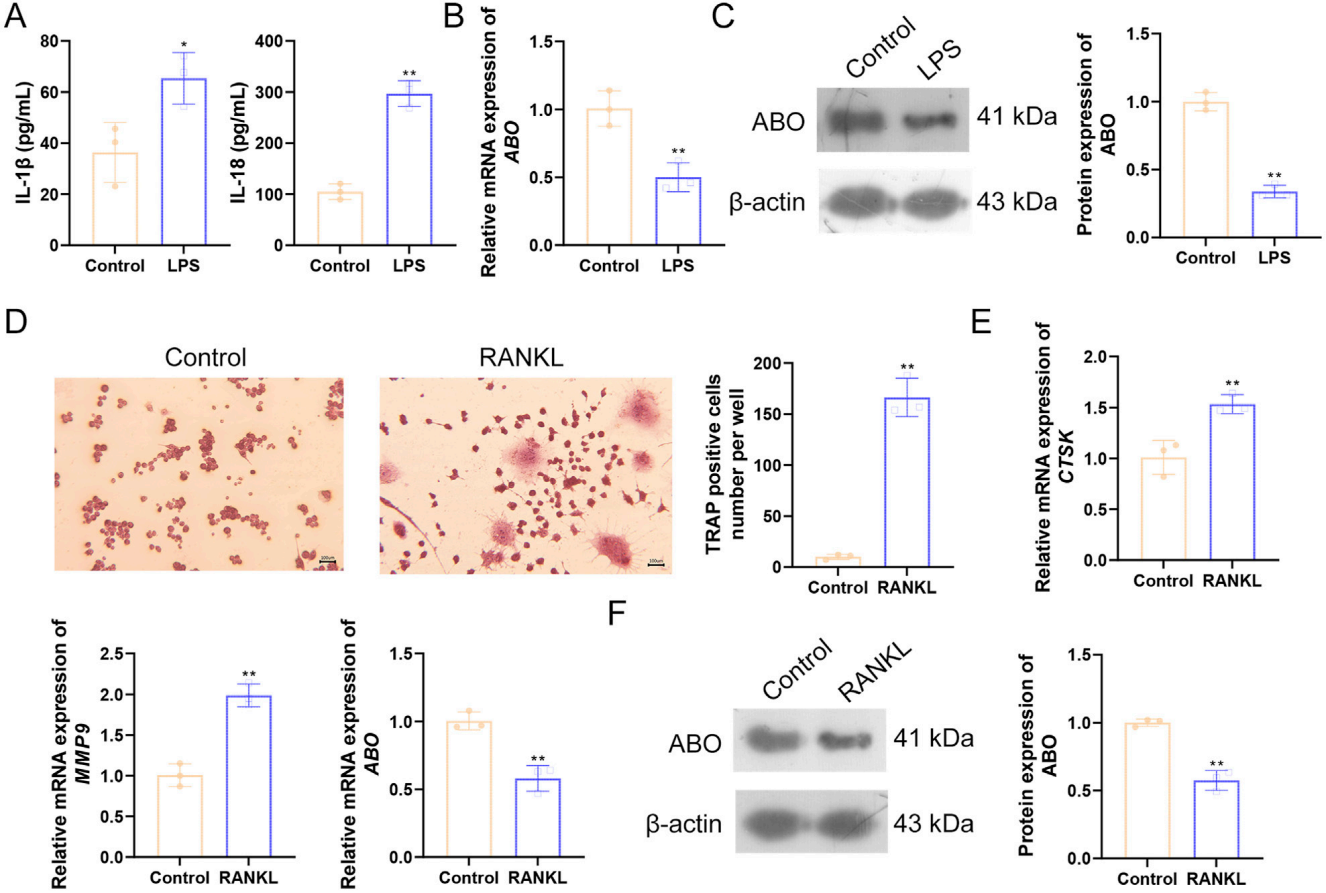
Validation of the role of ABO in CD and OP *in vitro*. **(A)** HT-29 cells were treated with 10 μg/mL LPS for 24 h, and the levels of IL-1β and IL-18 in the supernatant were detected using ELISA. The mRNA and protein levels of ABO in HT-29 cells were detected by **(B)** RT-qPCR and **(C)** Western blot. **(D)** RAW264.7 cells were cultured in a medium containing 50 ng/mL RANKL for 5 days, and the formation of osteoclasts was evaluated using TRAP staining. Scale bar = 100 μm. **(E)** The levels of *CTSK*, *MMP9*, and *ABO* were detected by RT-qPCR. **(F)** The protein levels of ABO were detected by Western blot. LPS, lipopolysaccharide; RANKL, receptor activator of nuclear factor κB ligand. *P < 0.05, **P < 0.01 vs Control group.

*In vitro* experiments were conducted to validate the role of *ABO* in CD and OP. HT-29 cells were treated with LPS to mimic the inflammatory environment of CD. LPS treatment significantly increased IL-1β and IL-18 levels in the cell supernatant (Figure 10A). Moreover, ABO levels in cells were markedly decreased after LPS treatment (Figures 10B,C). RAW264.7 cells were treated with RANKL to induce osteoclast differentiation. TRAP staining showed that the purple-red positive areas were significantly increased in RAW264.7 cells after RANKL stimulation compared to the control group, indicating their differentiation into mature osteoclasts (Figure 10D). The levels of bone resorption markers (*CTSK* and *MMP9*) were increased, while *ABO* levels were decreased after RANKL treatment (Figure 10E). Furthermore, RANKL induction also significantly reduced the protein level of ABO (Figure 10F). The expression pattern of *ABO* in the *in vitro* CD and OP models was consistent with the results of bioinformatics.

## 4 Discussion

OP is a common issue in CD patients (Zhao et al., 2023). Therefore, it is crucial to explore the common pathogenesis of both diseases. This study screened five potential shared diagnostic genes by WGCNA and two machine learning algorithms. *ABO* was identified as the hub co-diagnosis gene after verification. GSEA found that the biological pathways of *ABO* involved in the two diseases were mainly enriched in the mitochondrial matrix, chromosomal region, and ribosome. Immune infiltration analysis recognized immune cells significantly associated with diseases and *ABO*. *In vitro* experiments validated the results of bioinformatics. Overall, this study revealed a novel co-diagnostic gene and its underlying mechanisms, which provides new insights into the diagnosis and treatment of CD and OP.

CD is a non-specific autoimmune disease in which inflammation plays a vital role, it involves innate immunity to the intestinal mucosal barrier and extracellular matrix remodeling (Petagna et al., 2020). In addition, the immune system is linked to various types of osteoporosis development through different mechanisms. For example, estrogen deficiency mediates immune cell stimulation of osteoclast activation in postmenopausal osteoporosis, and aging promotes an immune imbalance that leads to bone loss in senile osteoporosis (Zhang et al., 2022). Hence, immune cells play an important role in both diseases. Immune infiltration analysis found that central memory CD8 T cell, monocyte, and plasmacytoid dendritic cell were markedly increased in CD and low-BMD individuals. Consistent with this finding, pro-inflammatory T cells promote bone resorption in osteoclasts, and long-term exposure to these cytokines can induce OP (Buchwald et al., 2012). CD8 T cells were significantly increased in peripheral blood mononuclear cells in CD patients (Chen et al., 2024). Postmenopausal OP and CD patients had higher monocyte levels than normal individuals (Li et al., 2024; Hu et al., 2024). Furthermore, functional osteoclasts derived from dendritic cells are directly involved in osteolytic osteopathy, and CD patients have increased dendritic cell levels in the intestinal lamina propria (Rivollier et al., 2004; Magnusson and Wick, 2011).

To further explore the co-pathogenesis of CD and OP, we performed enrichment analysis on the shared genes of the two diseases. The results showed that these genes were mainly enriched in endothelial cell morphogenesis, lipid glycosylation, glycosphingolipid biosynthesis-globo and isoglobo series, vitamin digestion and absorption, and glycosphingolipid biosynthesis-lacto and neolacto series. Enrichment of endothelial cell morphogenesis suggests possible changes in bone and intestinal microvascular development, thereby affecting intestinal barrier function and bone formation. Enrichment in lipid glycosylation and glycosphingolipid biosynthesis indicates shared lipid metabolism disturbances in intestinal inflammation and bone turnover. Enrichment of vitamin digestion and absorption indicates that abnormal utilization of nutrients may simultaneously regulate intestinal homeostasis and bone mineralization. Consistent with these results, specific deletion of *ZEB1* in endothelial cells leads to reduced osteogenesis (Fu et al., 2020). Dysfunction of the intestinal vascular barrier, which includes intestinal vascular endothelial cells, glial cells, and pericytes, is closely related to CD (Jingjie and June 2023). Congenital disorders of glycosylation, characterized by impaired glycosylation of proteins and lipids, contribute to decreased bone mineral density (Lipiński et al., 2021). In addition, glycosylation changes often occur in the colon epithelial cells of CD (Rhodes et al., 2008). Excess glycosphingolipids will affect the number and activity of osteoblasts and osteoclasts (Hughes et al., 2019). Bioactive sphingolipids can regulate biological functions and affect CD development (Gomez-Larrauri et al., 2020). Vitamin supplementation can improve CD and osteoporosis (Valvano et al., 2024; Rusu et al., 2024).

Machine learning further identified *GZMB*, *LTF*, *FAM129A*, *ABO*, and *KRT15* as the shared hub diagnostic genes for CD and OP. These genes all have certain biological significance and may have potential effects in CD and/or OP. GZMB is a cysteine protease-like serine proteolytic enzyme that is widely expressed in various hematopoietic and non-hematopoietic origin cells (Majchrzak et al., 2025). It is contained within the cytotoxic granules of cytotoxic T cells and natural killer cells. After target recognition, these granules are secreted into the immune synapse to promote apoptosis in infected or cancer cells (Cao et al., 2007). Studies have shown that GZMB can detect active IBD and predict the response to treatment (Heidari et al., 2024). LTF is a multifunctional protein composed of a polypeptide chain and is a component of most mammalian whey proteins (Kowalczyk et al., 2022). It possesses various properties, such as anti-inflammatory and immune regulation (Artym and Zimecki, 2021). LTF is a fecal inflammatory biomarker for CD in clinical practice, and it is used to predict disease recurrence after resection (Yamamoto and Shimoyama, 2017). Moreover, Samsonraj et al. identified LTF as a protein secreted by bone marrow-derived senescent mesenchymal stem cells, and the senescence-associated secretory phenotype is closely related to age-related changes in bone tissue (Samsonraj et al., 2023). FAM129A (also known as Niban) is an endoplasmic reticulum stress-related protein that regulates cell death by modulating eIF2a and S6K1/4E-BP1 phosphorylation (Sun et al., 2007). Wen et al. discovered that GBF1 regulates osteoclast activation by targeting EIF2a-mediated endoplasmic reticulum stress and FAM129A (Wen et al., 2021). KRT15 is a type I cytoskeletal protein mainly expressed in keratinocytes of stratified epithelial cells, and it regulates the renewal and repair of basal cells (Alsaegh et al., 2019). Studies have shown that *NOD2* mutations are associated with an increased risk of CD, and KRT15 is a protein that interacts with NOD2, suggesting its potential role in CD (Thiébaut et al., 2016). The *ABO* gene is located on chromosome 9 and contains seven exons, about 19.5 kb long (Yamamoto et al., 1995). A or B glycosyltransferase (GT) encoded by the *A* and *B* genes can synthesize ABO antigens in red blood cells (Morgan and Watkins, 2000). Therefore, genetic changes in *ABO* can cause weak ABO phenotypes by affecting GTs (Lei et al., 2024). ABO blood group antigens were initially applied in blood transfusion and transplantation (Heal et al., 2005). Additionally, ABO blood type is associated with the susceptibility of the body to various diseases, such as cancer, cardiovascular diseases, infectious diseases, and cognitive disorders (Franchini et al., 2012; Bullerdiek et al., 2022; Alexander et al., 2014). Nevertheless, the research on ABO in CD and OP is relatively limited. A study revealed that ABO blood group distribution among CD patients is significantly different from that of normal individuals, with the AB blood group presenting the highest risk (Jiang et al., 2024). In addition, previous studies have shown that women with blood group AB have the lowest bone density and are more likely to develop OP (Kaur, 2014). Interestingly, patients with the AB blood group show a high risk for both diseases.

We analyzed the expression patterns of these shared hub diagnostic genes in the CD and OP datasets and *in vitro* models, and found that *ABO* was consistently and significantly downregulated in the disease groups. Therefore, it was identified as a novel hub co-diagnostic gene for CD and OP. Moreover, ROC curves, calibration curves, and DCA of the ABO model indicate that it exhibits excellent discrimination, calibration performance and clinical utility in both diseases. GSEA results found that the biological pathways of *ABO* involvement in both diseases are enriched in the mitochondrial matrix, chromosomal region, and ribosome. Aldehyde dehydrogenase 2 is present in the mitochondrial matrix, and its inactivation mutations increase the tendency for OP (Chen et al., 2014). In addition, mutations in carnitine transporter genes *OCTN1* and *OCTN2* are closely related to CD, and carnitine-dependent entry of long-chain fatty acids into the mitochondrial matrix regulates fatty acid oxidative to improve intestinal function (Shekhawat et al., 2007). The *STAT6* gene is located in the 12p13.2-q24.1 region of the chromosome, which has polymorphism and participates in CD development by regulating TH1/TH2 immune response (Klein et al., 2005). Besides, dysfunction at the sites of ribosome transcription and synthesis leads to childhood progerias, which is characterized by OP, and disruption of ribosome biogenesis also affects mitochondria (Phan et al., 2019). Correlation analysis confirmed a significant correlation between ABO and the mitochondrial pathway in both CD and OP, suggesting the potential involvement of this pathway in ABO-based co-diagnosis of CD and OP. T cells are involved in regulating the production of anti-A natural antibodies (Adam et al., 2023). B cell response to T cell-independent antigens leads to the rejection of ABO-incompatible allografts (Sakai et al., 2021). Similarly, immune infiltration results found that *ABO* was significantly negatively correlated with activated CD8 T cell, effector memory CD4 T cell, and immature B cell in both CD and OP. Notably, the correlation between *ABO* and these immune cells still exists in CD patients who have received hormone therapy, suggesting that the association between them may be partially independent of the drug intervention. Activated CD8 T cells directly kill intestinal epithelial cells through the perforin/granzyme pathway, damaging the barrier, and produce pro-inflammatory cytokines such as IFN-γ and TNF-α, further driving intestinal inflammation (Kappeler and Mueller, 2000). The pathology of CD involves the dysregulation of the CD4 T cell homeostasis controlled by the TNF-α/IL-6 and IL-10/TGF-β networks (Ma et al., 2025). These cell-mediated intestinal mucosal inflammation may indirectly contribute to the occurrence of OP. Furthermore, activated T lymphocytes are the main source of RANKL and TNF-α, and are closely related to bone destruction. CD4 T and CD8 T cells also possess anti-osteoclast formation properties, as their depletion leads to a reduction in the formation of osteoprotegerin (Srivastava et al., 2018). However, it remains to be further investigated whether *ABO* affects this process by regulating these immune cells.

Many studies have reported an association between OP and CD, but few have investigated the correlation and mechanisms between them. This study explored the co-pathogenesis of CD and OP and screened for co-diagnostic markers. However, this study has some limitations. First, only two datasets were used for CD and OP analysis, and the sample size was small. Moreover, the results were only preliminarily validated in cell experiments, and *in vivo* studies and further functional assays are still needed. Additionally, this study did not incorporate the common risk factors for CD and OP (such as age, gender, BMI, and gut microbiota) into the analysis, thus we were unable to eliminate the interference of these factors on the conclusions. Furthermore, we did not conduct a combined diagnostic analysis of ABO with the existing clinical indicators, which prevented us from determining whether ABO could enhance the accuracy of the existing diagnostic protocols.

## 5 Conclusion

*ABO* was screened as the hub co-diagnostic gene for CD and OP, showing good diagnostic value and providing a theoretical basis for their diagnosis and treatment.

## Data Availability

The data that support the findings of this study are available from the corresponding author upon reasonable request.
