# Correction: Identification of shared diagnostic genes between osteoporosis and Crohn’s disease through integrated transcriptomic analysis and machine learning

**DOI:** 10.3389/fgene.2025.1722642

**Published:** 2025-11-07

**Authors:** Guirong Yi, Peng Zhou, Qinxu Yang, Maosheng Zhao, Qiaoqiao Yang, Shensong Li, Chenpo Dang

**Affiliations:** 1 Department of Gastroenterology, The Second Hospital and Clinical Medical School, Lanzhou University, Lanzhou, Gansu, China; 2 Department of sports medicine, The 940th Hospital of Joint Logistic Support Force of Chinese People’s Liberation Army, Lanzhou, Gansu, China; 3 Department of orthopedics, The 941th Hospital of Joint Logistic Support Force of Chinese People’s Liberation Army, Xining, Qinghai, China

**Keywords:** osteoporosis, Crohn’s disease, co-diagnosis, weighted gene co-expression network analysis, machine learning

The **title** of this article was corrected from “Title identification of shared diagnostic genes between osteoporosis and Crohn’s disease through integrated transcriptomic analysis and machine learning” to “Identification of shared diagnostic genes between osteoporosis and Crohn’s disease through integrated transcriptomic analysis and machine learning”.

The original article has been updated.

