# Supplementary material for: Identification of shared diagnostic genes between osteoporosis and Crohn’s disease through integrated transcriptomic analysis and machine learning
Source: Front Genet. 2025 Oct 7;16:1609915. doi: 10.3389/fgene.2025.1609915 (PMC12538133; doi:10.3389/fgene.2025.1609915)
Supplement: Supplementary file 1 [file Supplementaryfile1.zip › Supplementary Material/02_WGCNA/4.1_WGCNA_OP_GSE56815/02_Clustering_with_pheno.pdf]

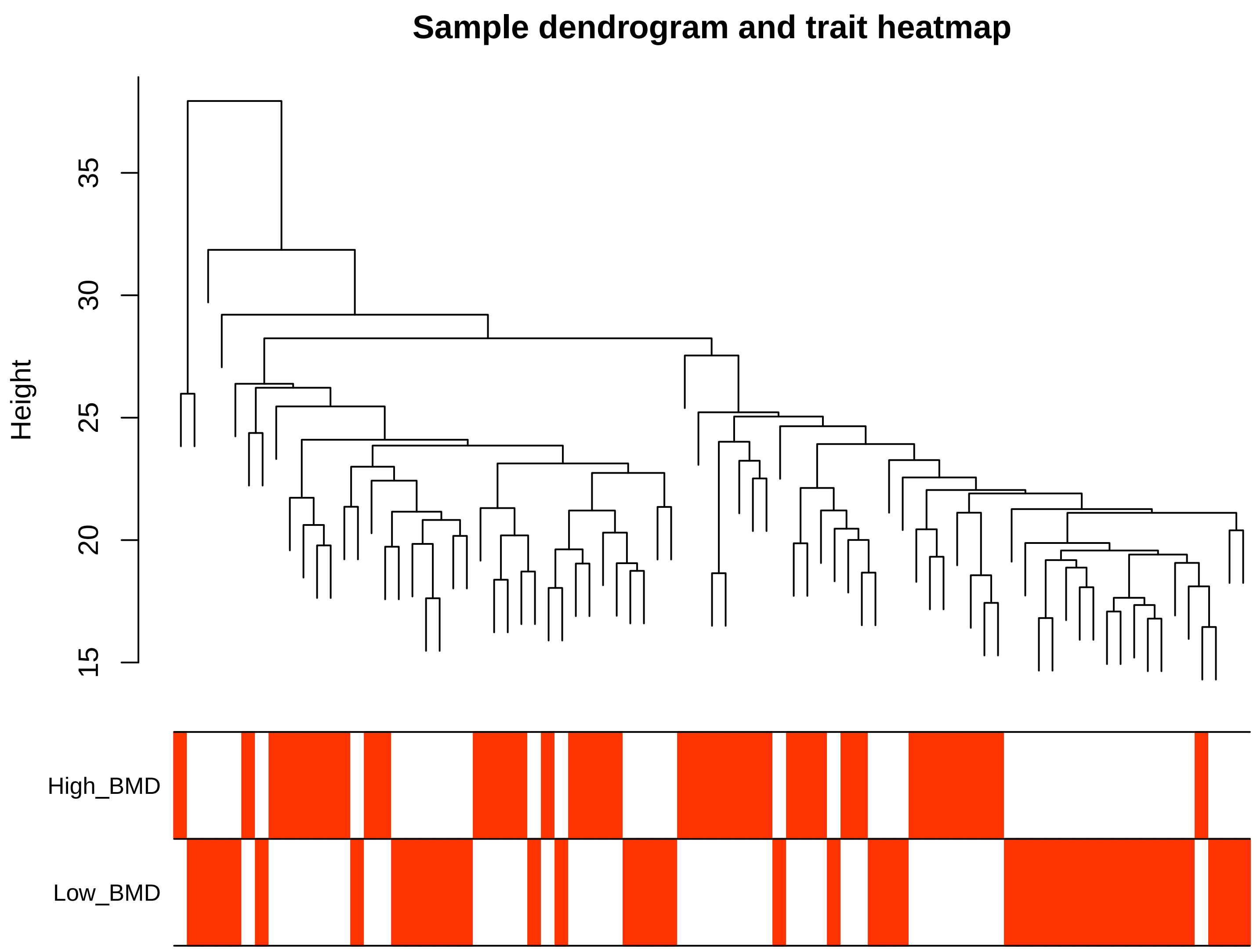
Sample dendrogram and trait heatmap
Height
35
30
25
20
15
High_BMD
Low_BMD
