# Supplementary figures and images for: Identification of shared diagnostic genes between osteoporosis and Crohn’s disease through integrated transcriptomic analysis and machine learning (part 2 of 2)
Source: Front Genet. 2025 Oct 7;16:1609915. doi: 10.3389/fgene.2025.1609915 (PMC12538133; doi:10.3389/fgene.2025.1609915)

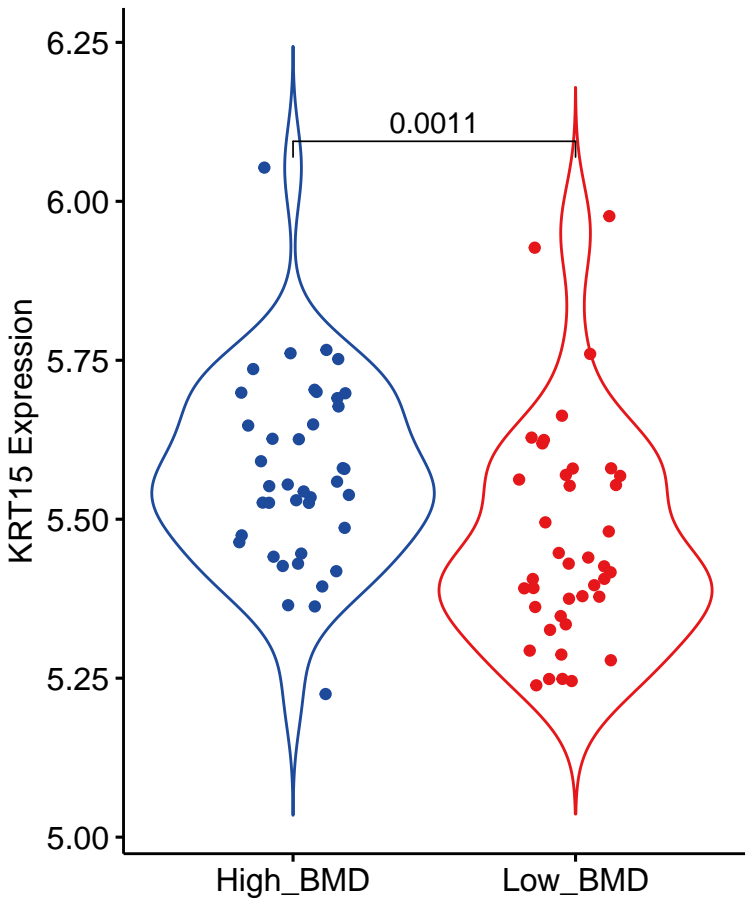

Supplement: Supplementary file 1 [file Supplementaryfile1.zip › Supplementary Material/03_ML/5.3_OP_Train_KRT15.pdf]

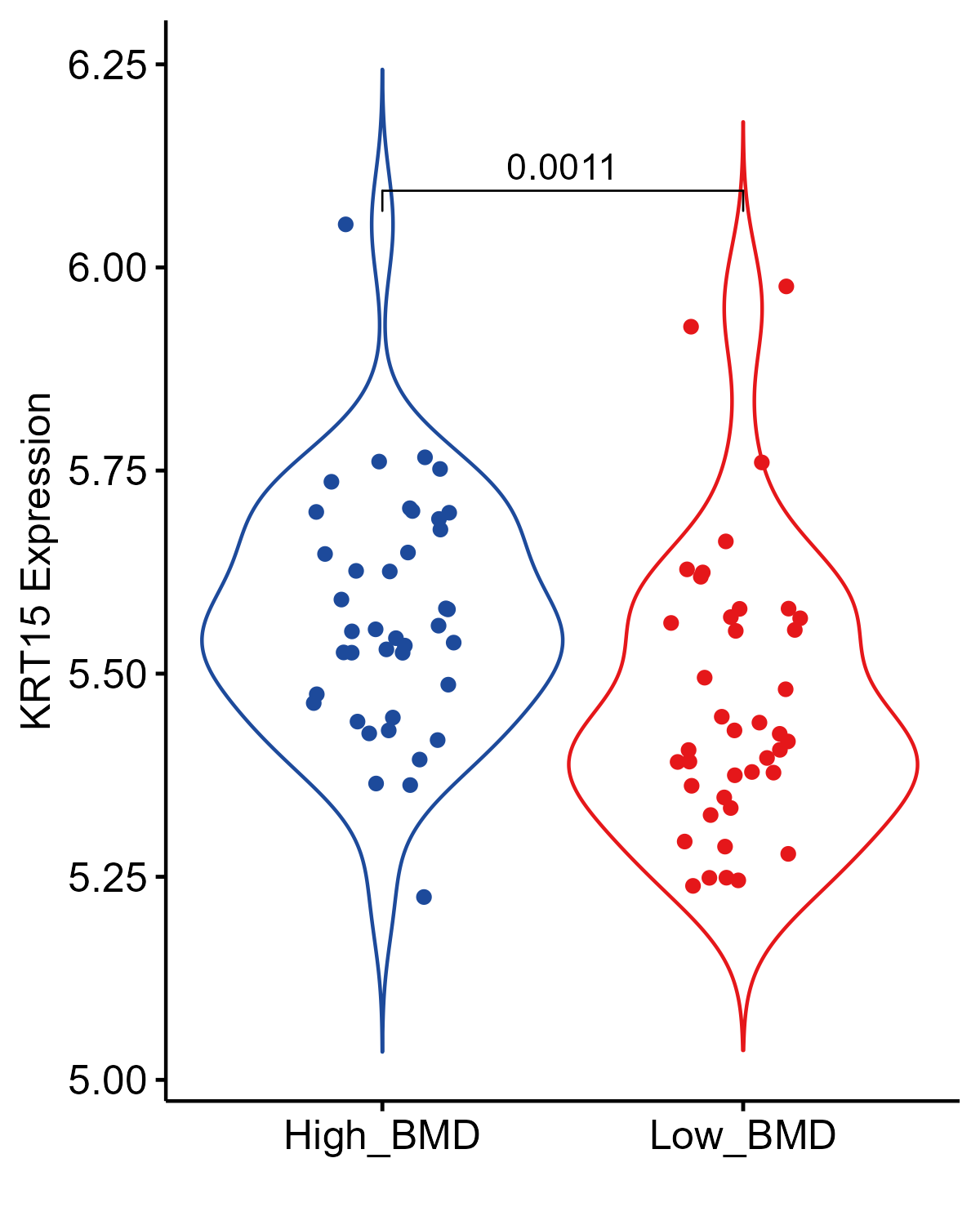

Supplement: Supplementary file 1 [file Supplementaryfile1.zip › Supplementary Material/03_ML/5.3_OP_Train_KRT15.png]

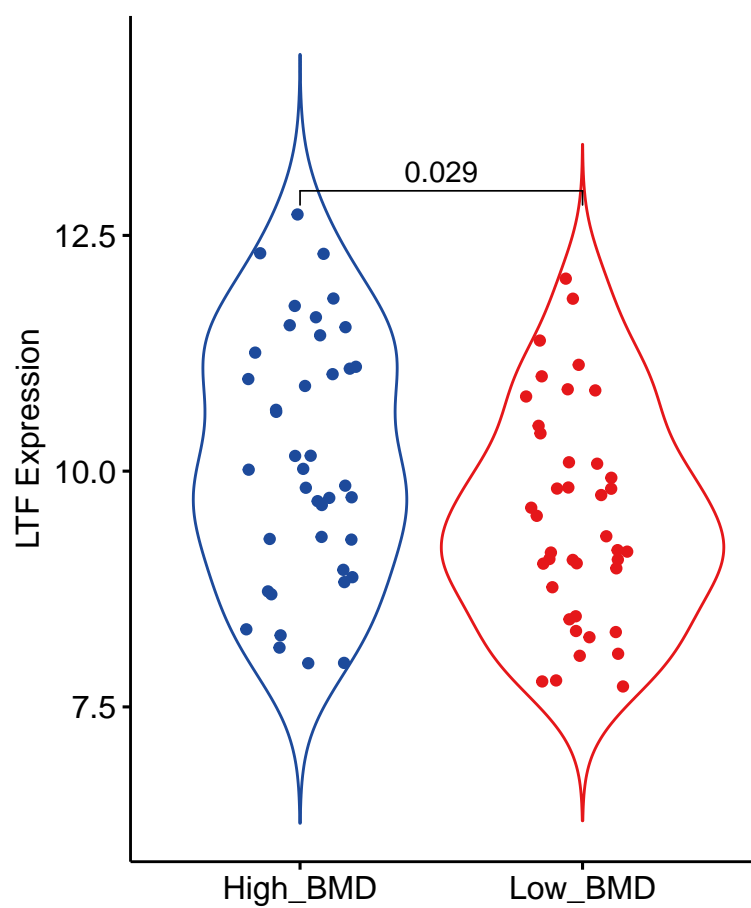

Supplement: Supplementary file 1 [file Supplementaryfile1.zip › Supplementary Material/03_ML/5.3_OP_Train_LTF.pdf]

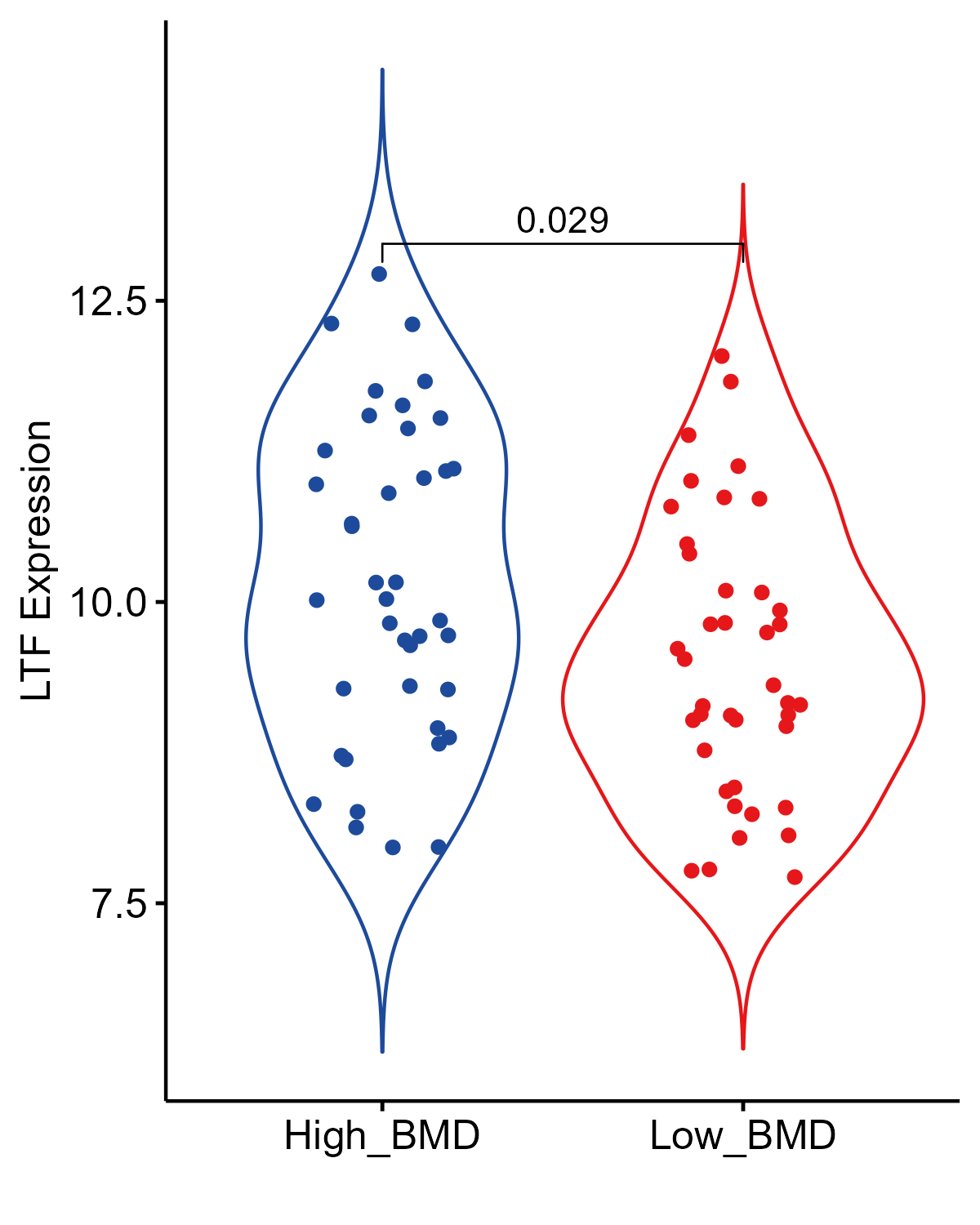

Supplement: Supplementary file 1 [file Supplementaryfile1.zip › Supplementary Material/03_ML/5.3_OP_Train_LTF.png]

## ABO Train

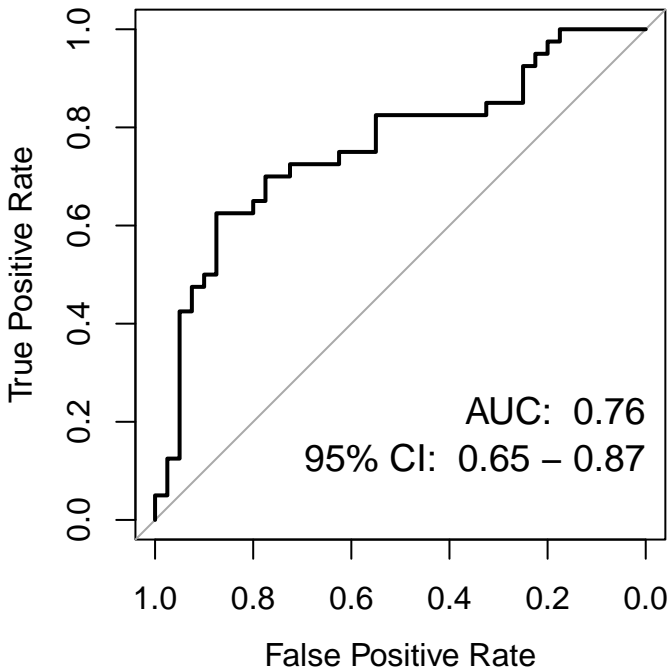

Supplement: Supplementary file 1 [file Supplementaryfile1.zip › Supplementary Material/03_ML/5.3_OP_Train_ROC_ABO.pdf]

## FAM129A Train

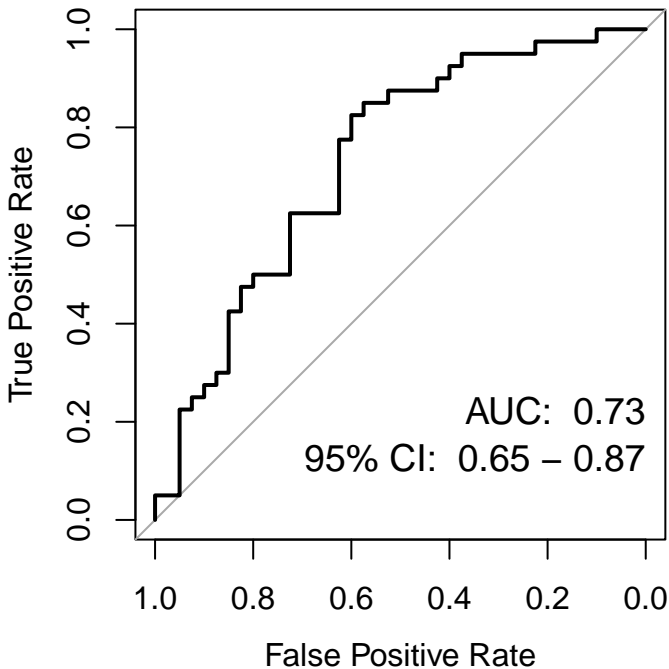

Supplement: Supplementary file 1 [file Supplementaryfile1.zip › Supplementary Material/03_ML/5.3_OP_Train_ROC_FAM129A.pdf]

## GZMB Train

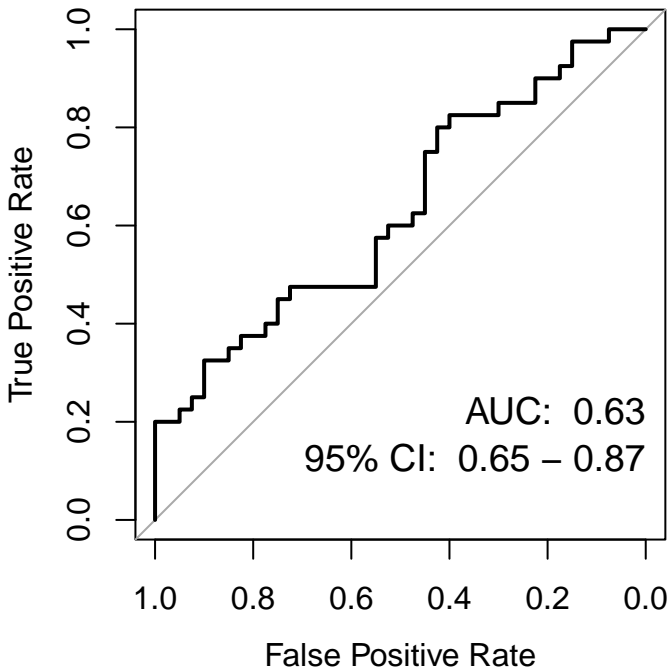

Supplement: Supplementary file 1 [file Supplementaryfile1.zip › Supplementary Material/03_ML/5.3_OP_Train_ROC_GZMB.pdf]

## KRT15 Train

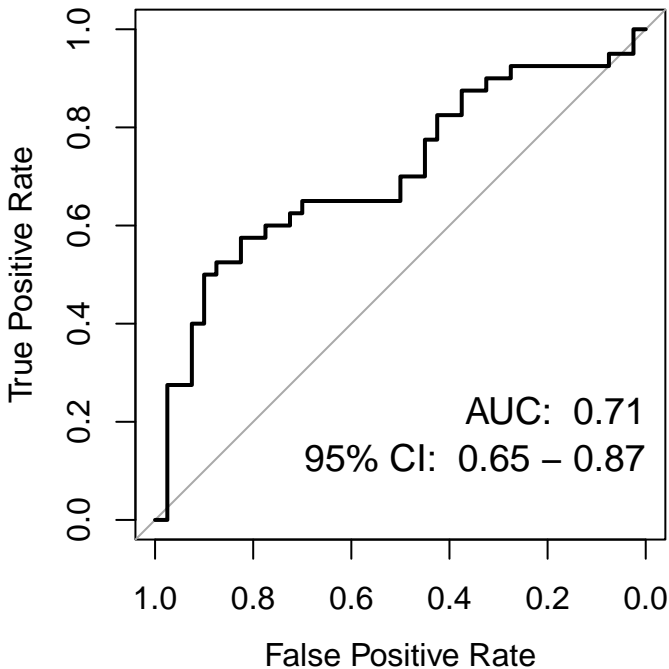

Supplement: Supplementary file 1 [file Supplementaryfile1.zip › Supplementary Material/03_ML/5.3_OP_Train_ROC_KRT15.pdf]

## LTF Train

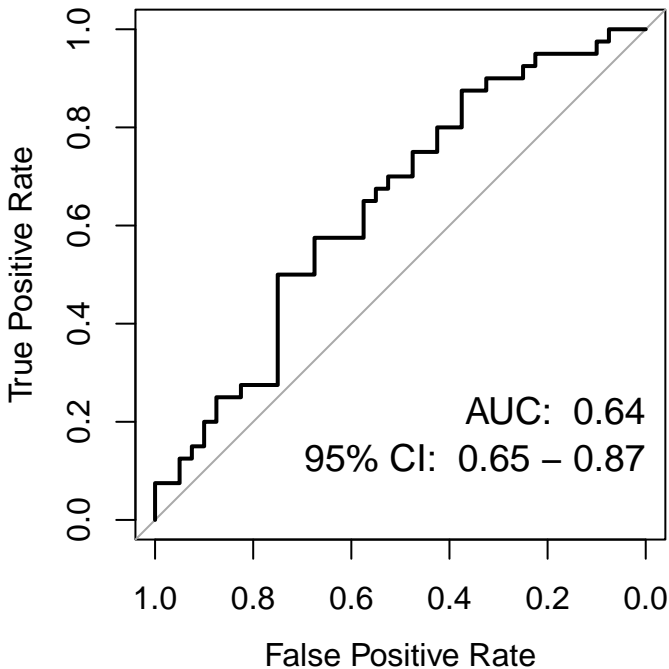

Supplement: Supplementary file 1 [file Supplementaryfile1.zip › Supplementary Material/03_ML/5.3_OP_Train_ROC_LTF.pdf]

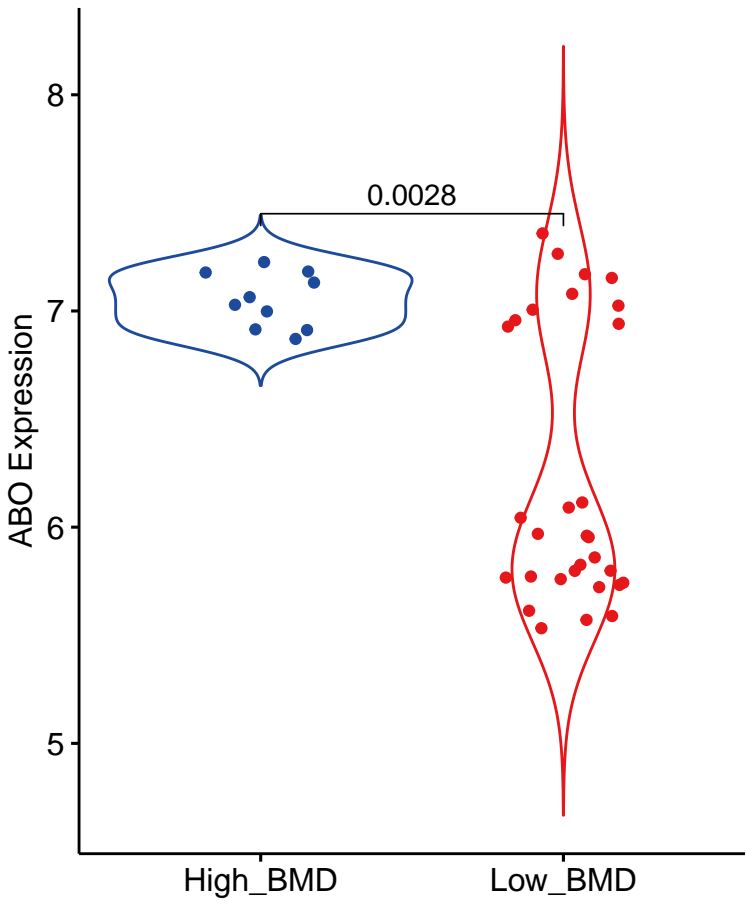

Supplement: Supplementary file 1 [file Supplementaryfile1.zip › Supplementary Material/03_ML/5.4_OP_Valid_ABO.pdf]

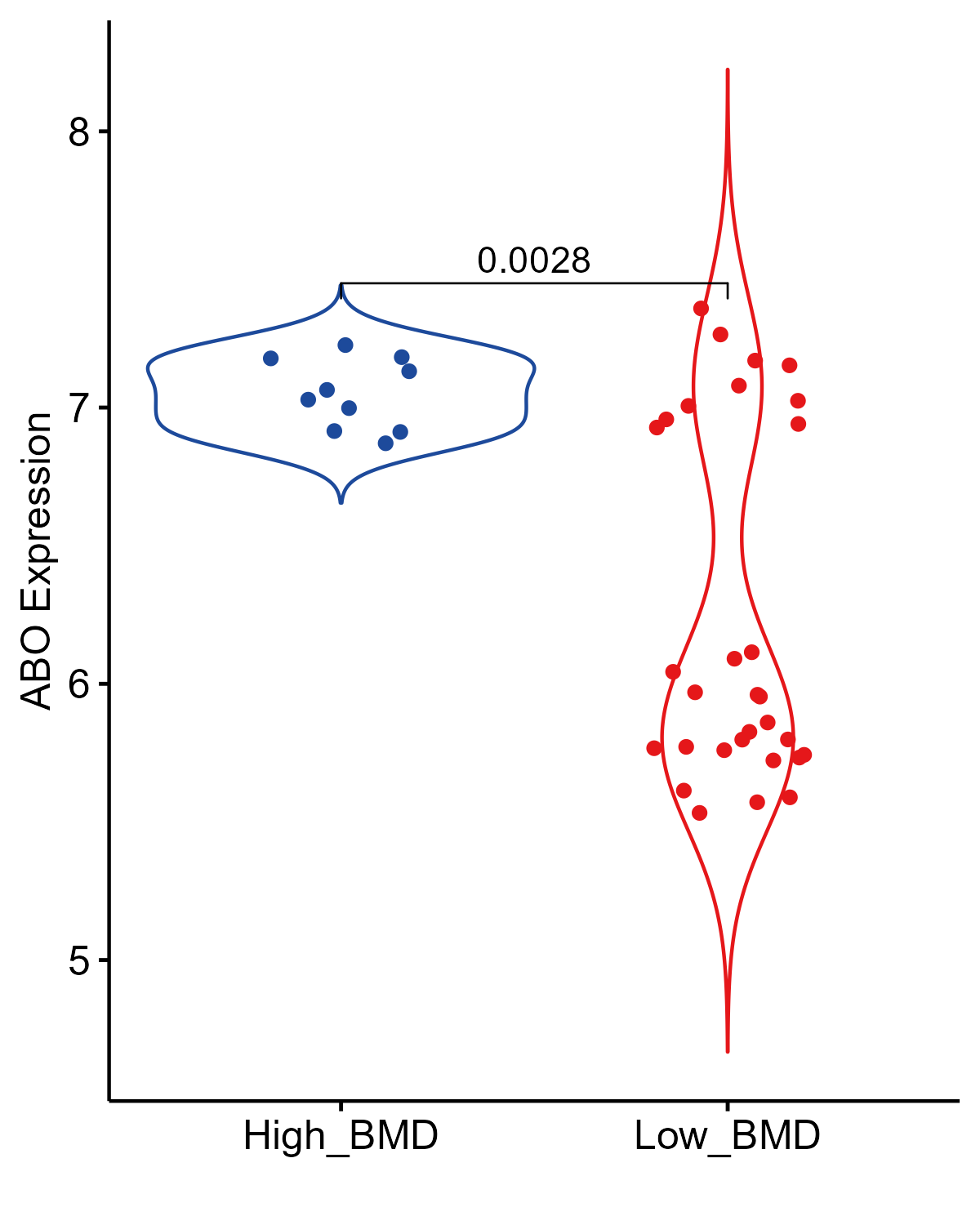

Supplement: Supplementary file 1 [file Supplementaryfile1.zip › Supplementary Material/03_ML/5.4_OP_Valid_ABO.png]

KRT15 Expression

6.0

5.5

5.0

4.5

0.0001

High\_BMD

Low\_BMD

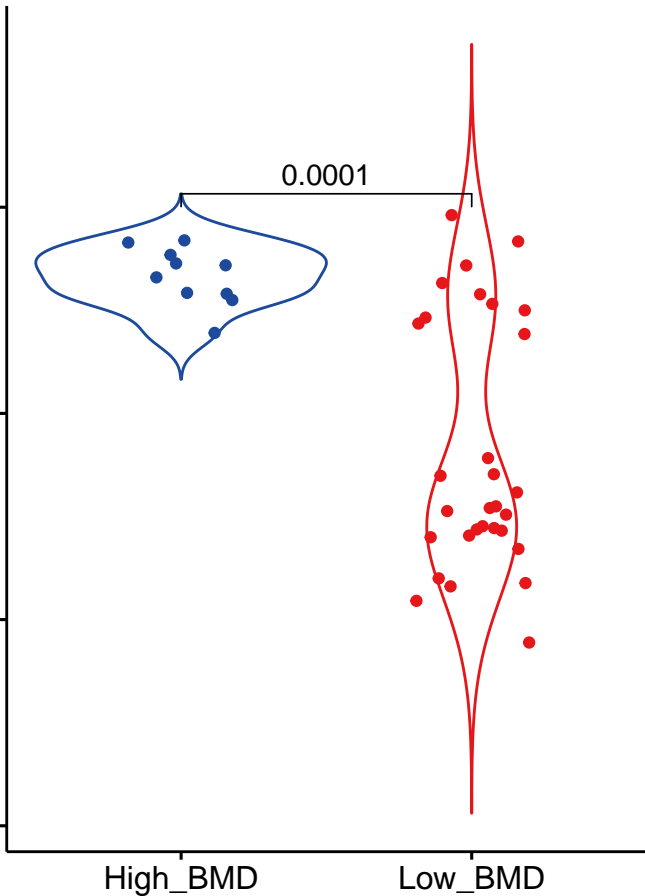

Supplement: Supplementary file 1 [file Supplementaryfile1.zip › Supplementary Material/03_ML/5.4_OP_Valid_KRT15.pdf]

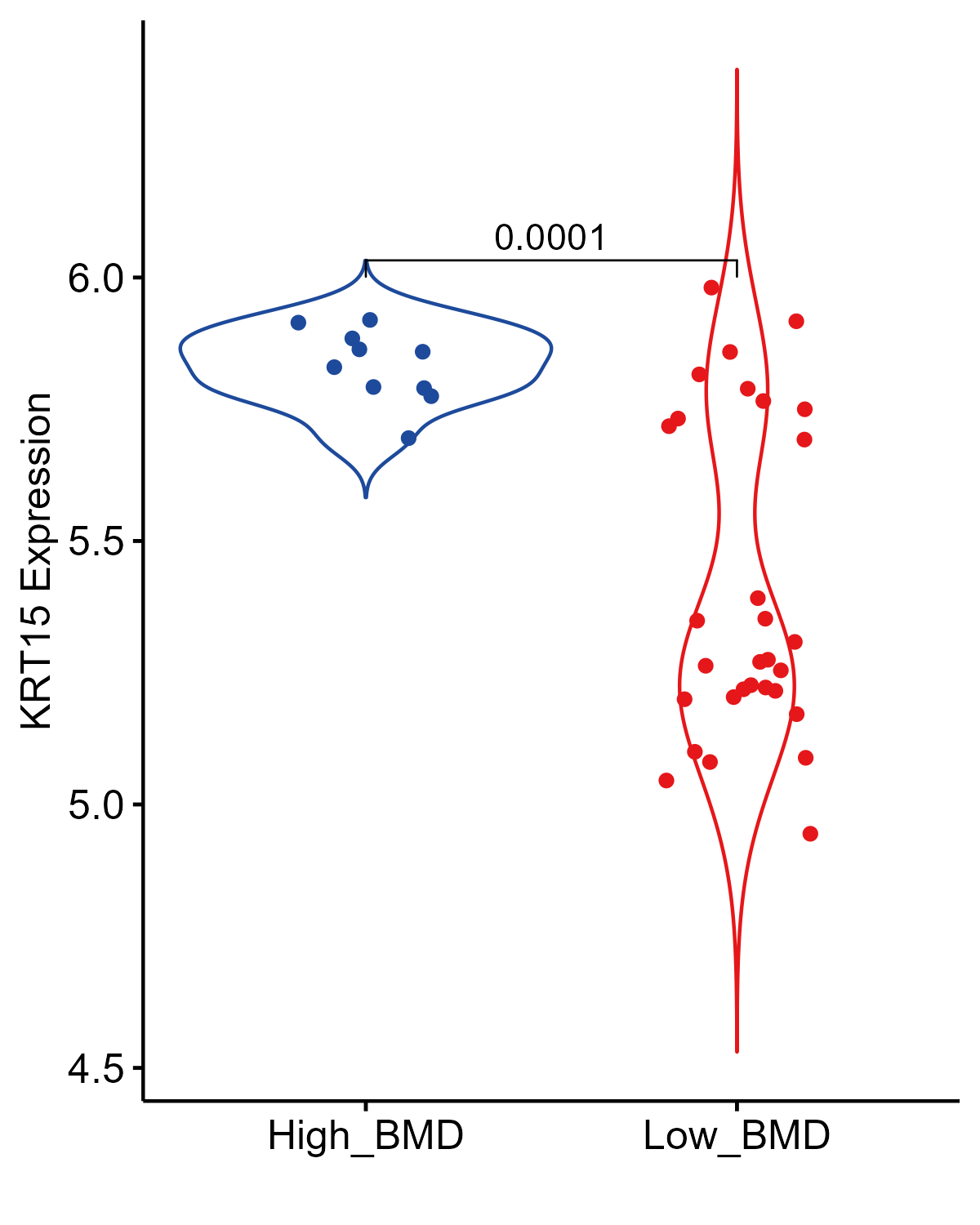

Supplement: Supplementary file 1 [file Supplementaryfile1.zip › Supplementary Material/03_ML/5.4_OP_Valid_KRT15.png]

## ABO Valid

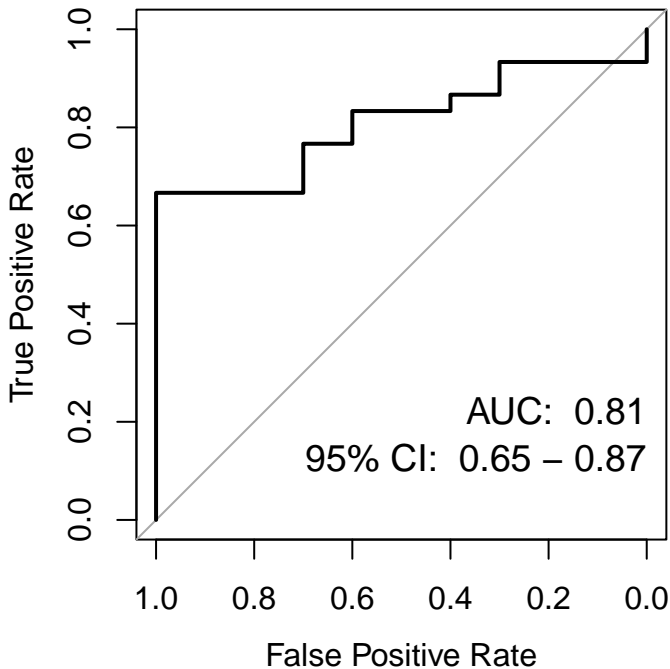

Supplement: Supplementary file 1 [file Supplementaryfile1.zip › Supplementary Material/03_ML/5.4_OP_Valid_ROC_ABO.pdf]

## KRT15 Valid

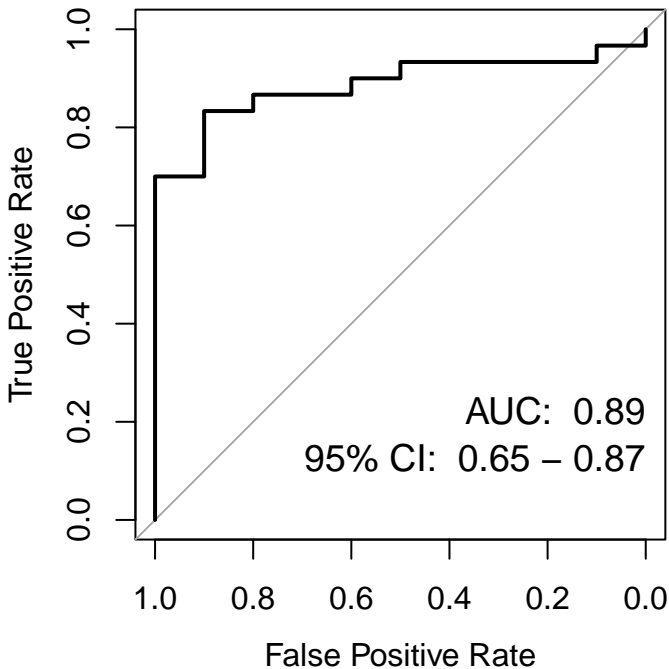

Supplement: Supplementary file 1 [file Supplementaryfile1.zip › Supplementary Material/03_ML/5.4_OP_Valid_ROC_KRT15.pdf]

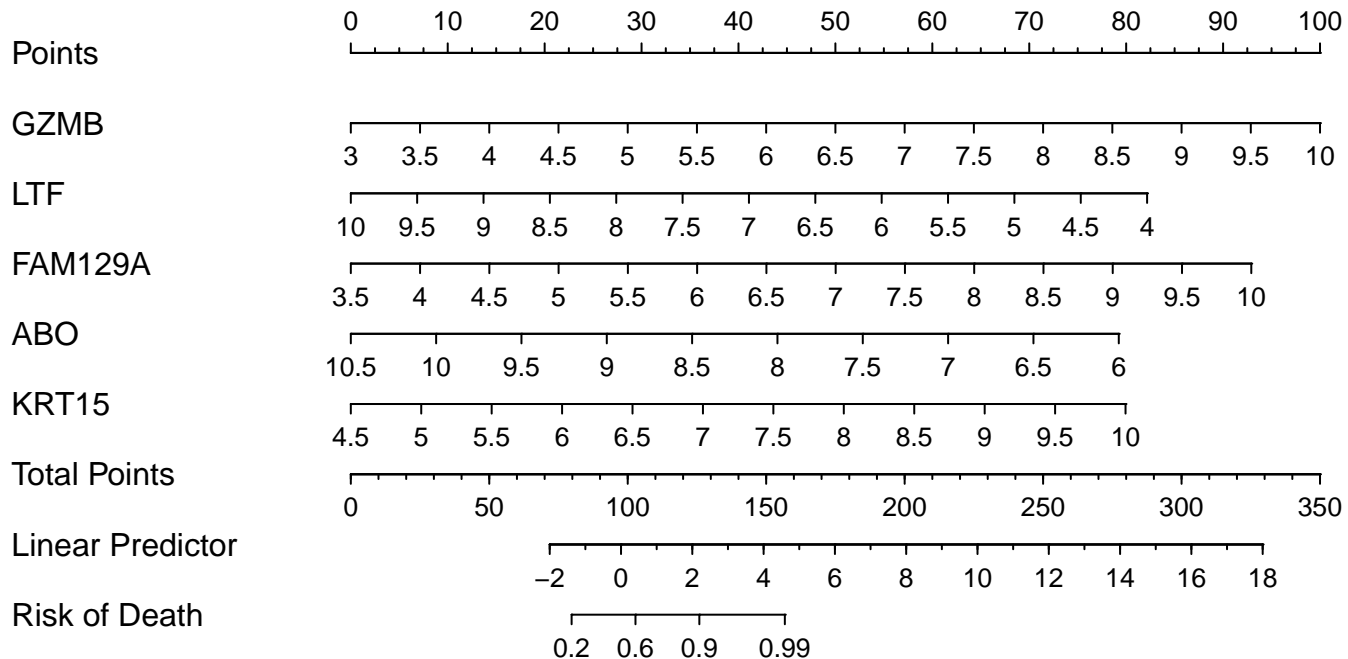

Supplement: Supplementary file 1 [file Supplementaryfile1.zip › Supplementary Material/03_ML/6.1_Nomo_CD.pdf]

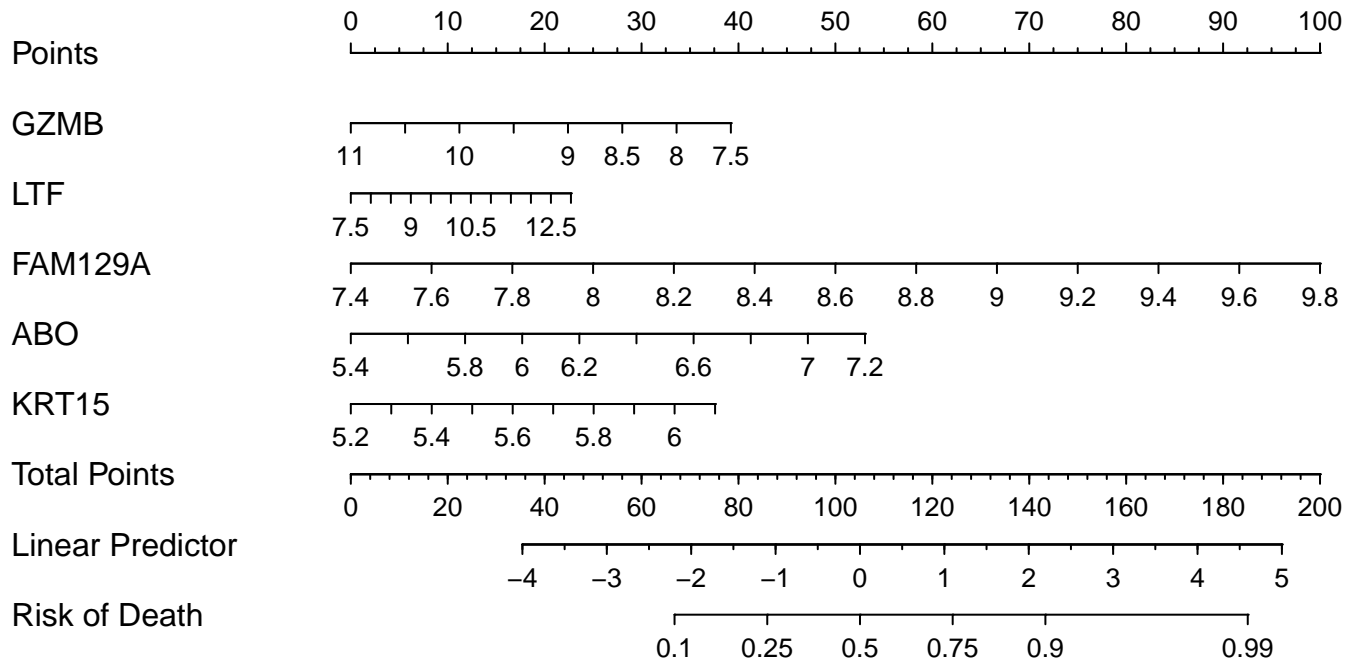

Supplement: Supplementary file 1 [file Supplementaryfile1.zip › Supplementary Material/03_ML/6.2_Nomo_OP.pdf]

Disease ■ CD ■ Normal

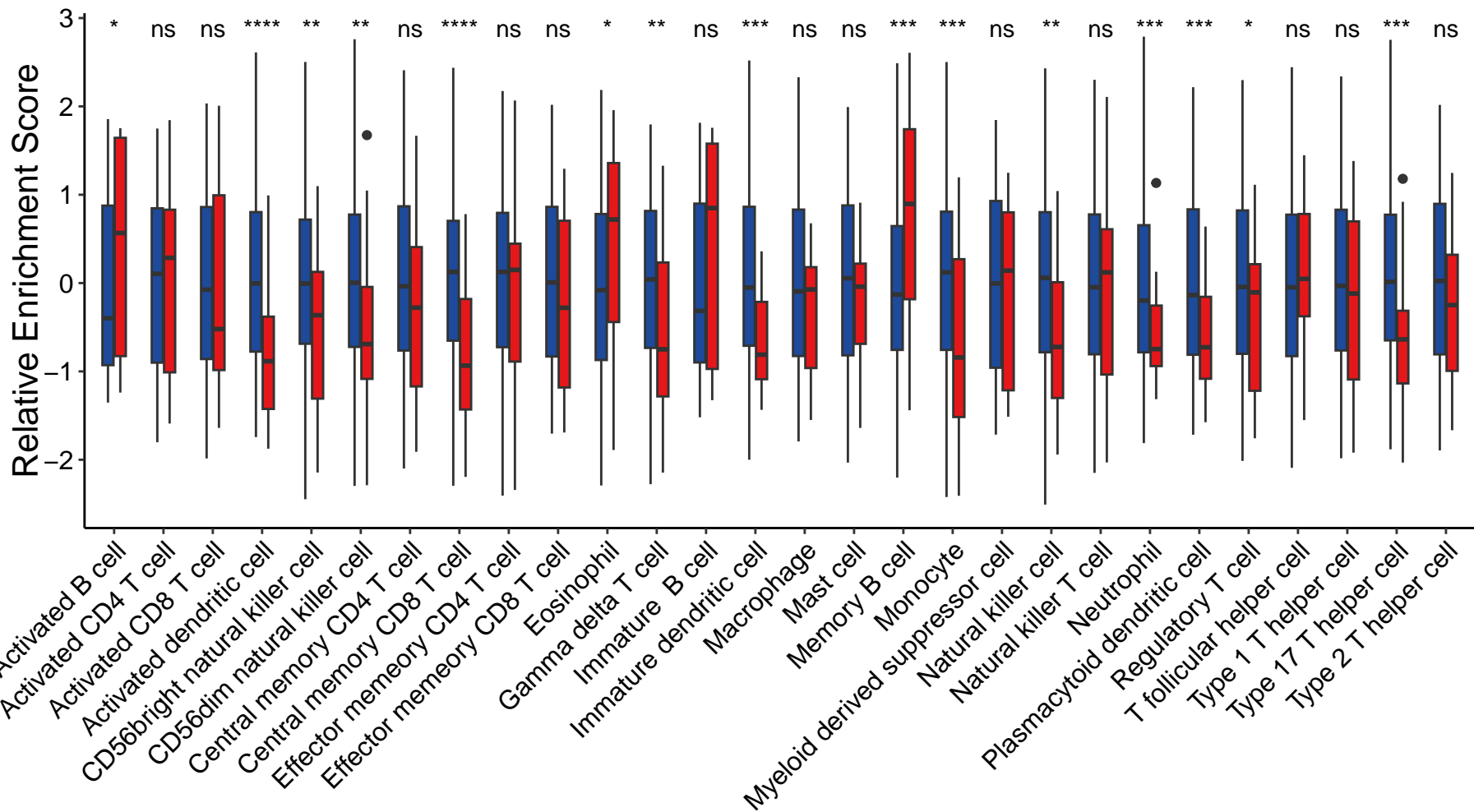

Supplement: Supplementary file 1 [file Supplementaryfile1.zip › Supplementary Material/04_Immune/1.3_CD_ESTIMATE_boxplot.pdf]

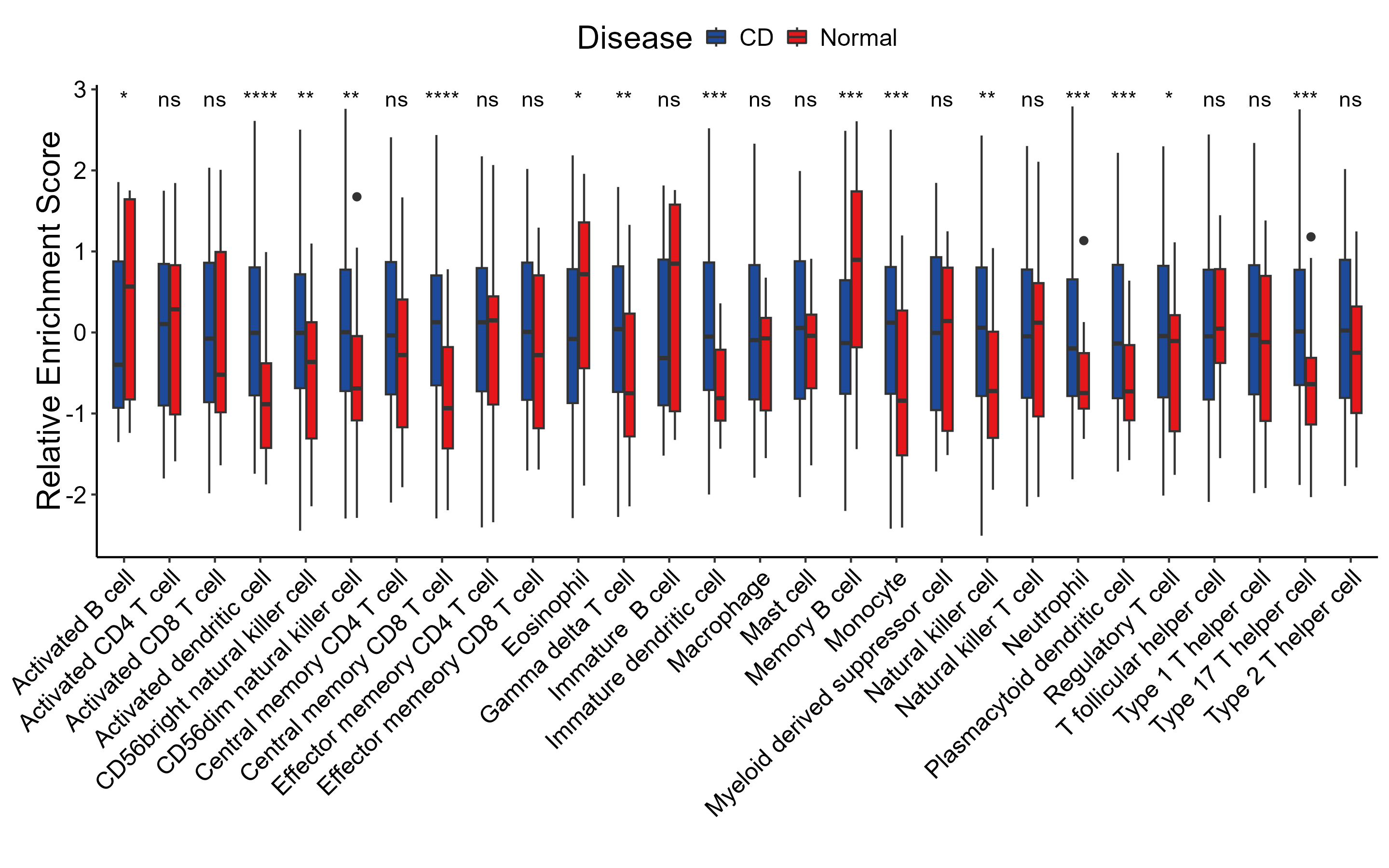

Supplement: Supplementary file 1 [file Supplementaryfile1.zip › Supplementary Material/04_Immune/1.3_CD_ESTIMATE_boxplot.png]

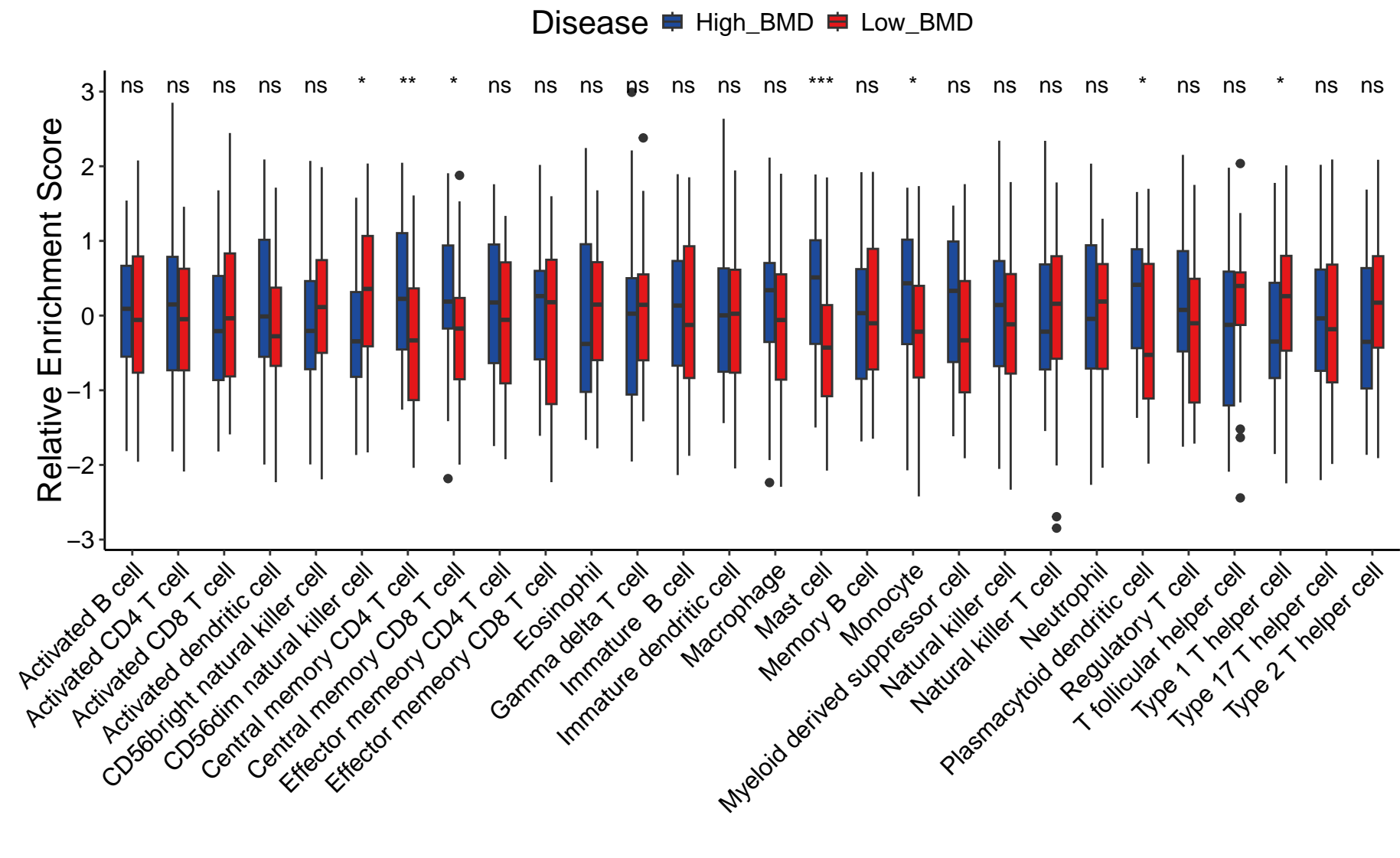

Supplement: Supplementary file 1 [file Supplementaryfile1.zip › Supplementary Material/04_Immune/2.2_OP_ESTIMATE_boxplot.pdf]

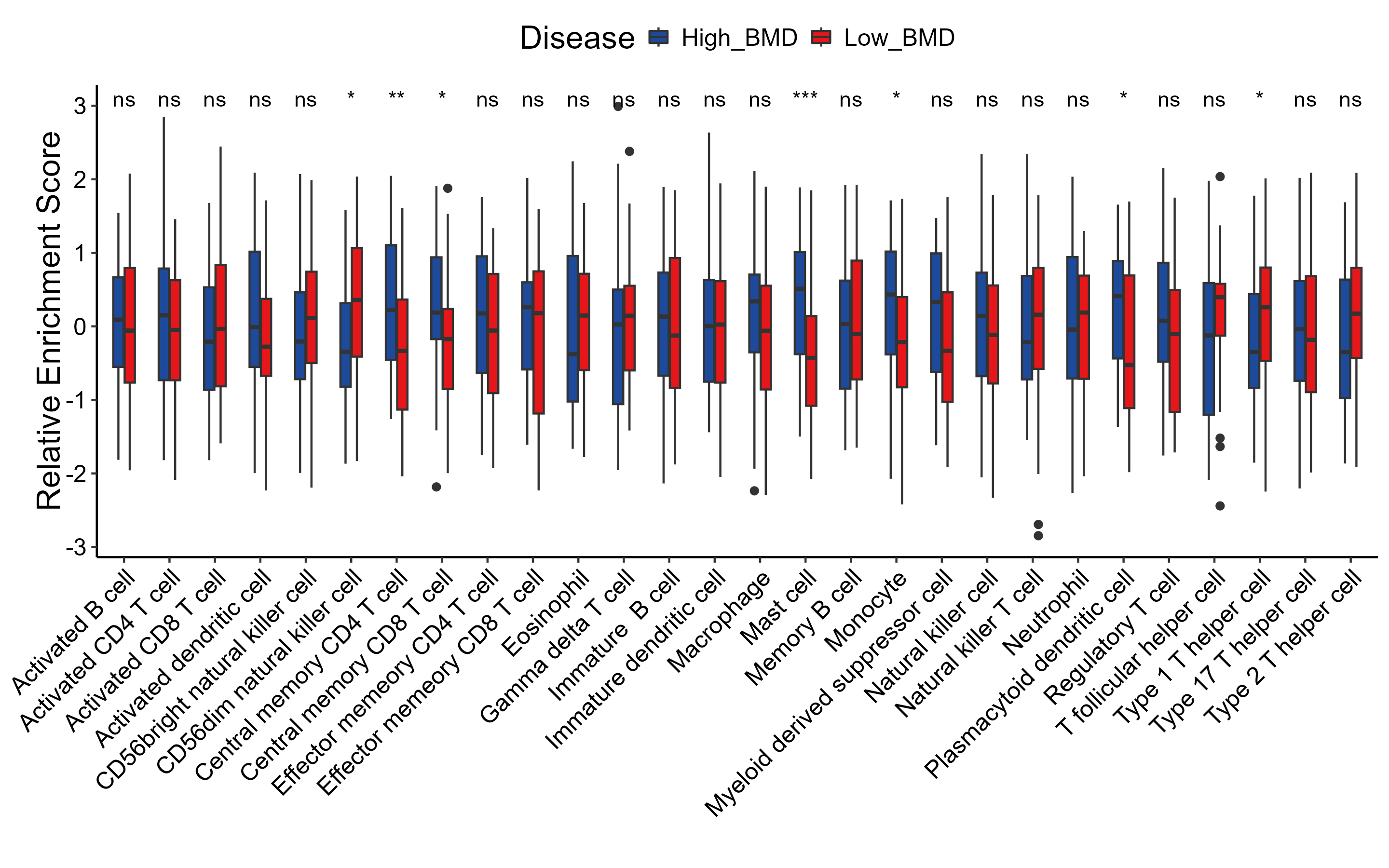

Supplement: Supplementary file 1 [file Supplementaryfile1.zip › Supplementary Material/04_Immune/2.2_OP_ESTIMATE_boxplot.png]

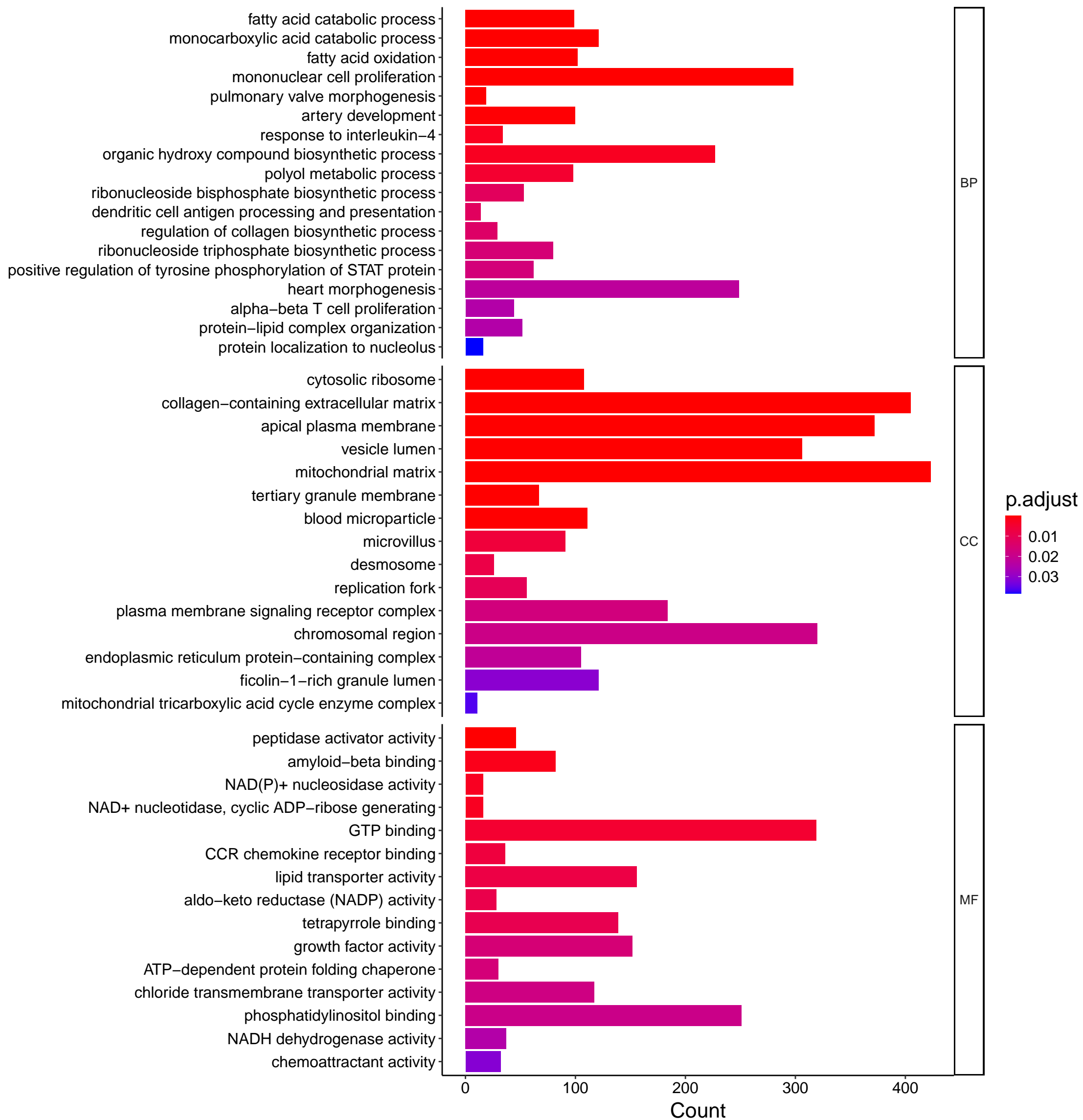

Supplement: Supplementary file 1 [file Supplementaryfile1.zip › Supplementary Material/04_Immune/3.1_CD_ABO_ssGSEA_GO_barplot.pdf.pdf]

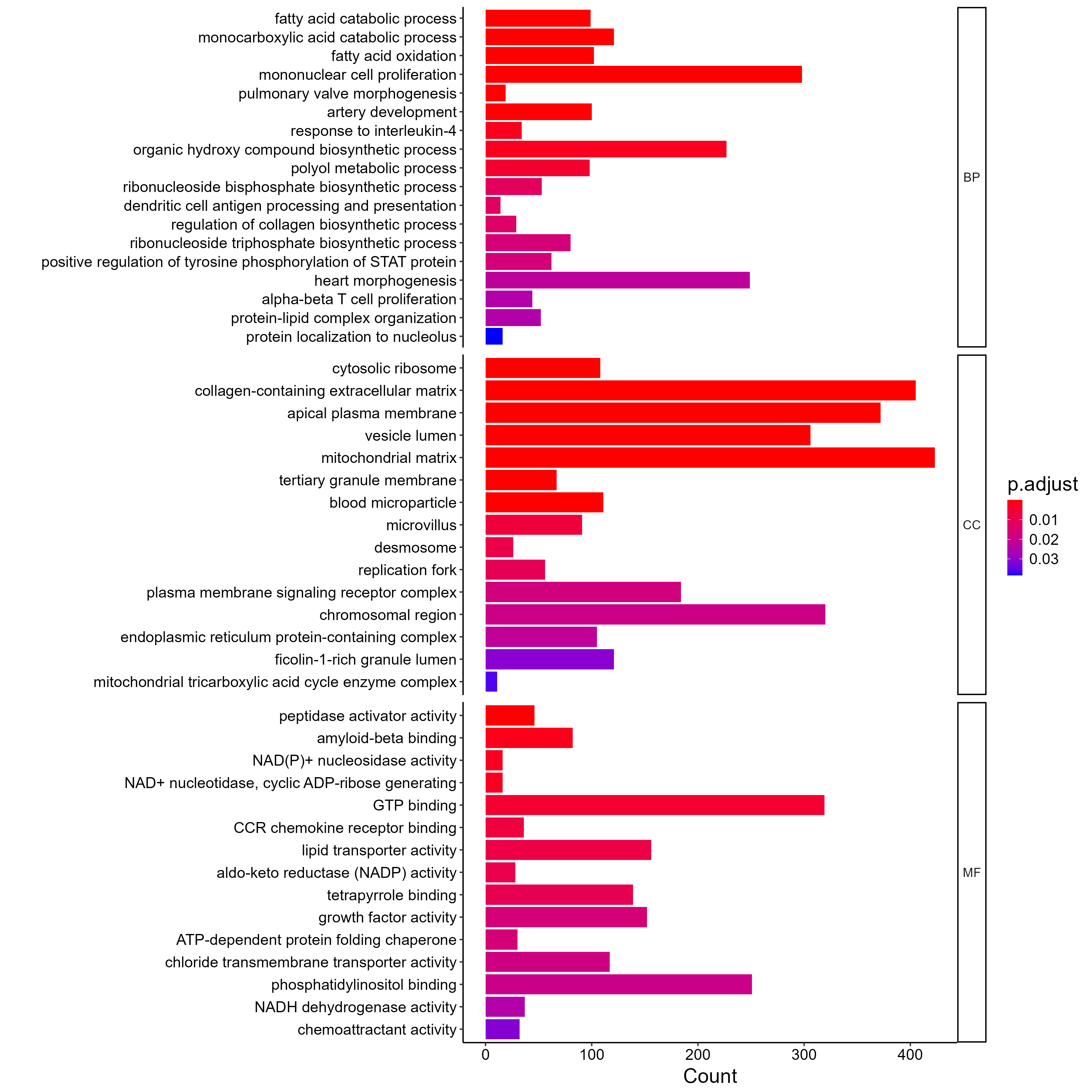

Supplement: Supplementary file 1 [file Supplementaryfile1.zip › Supplementary Material/04_Immune/3.1_CD_ABO_ssGSEA_GO_barplot.pdf.png]

Pathway Change    ■ Down    ■ Up

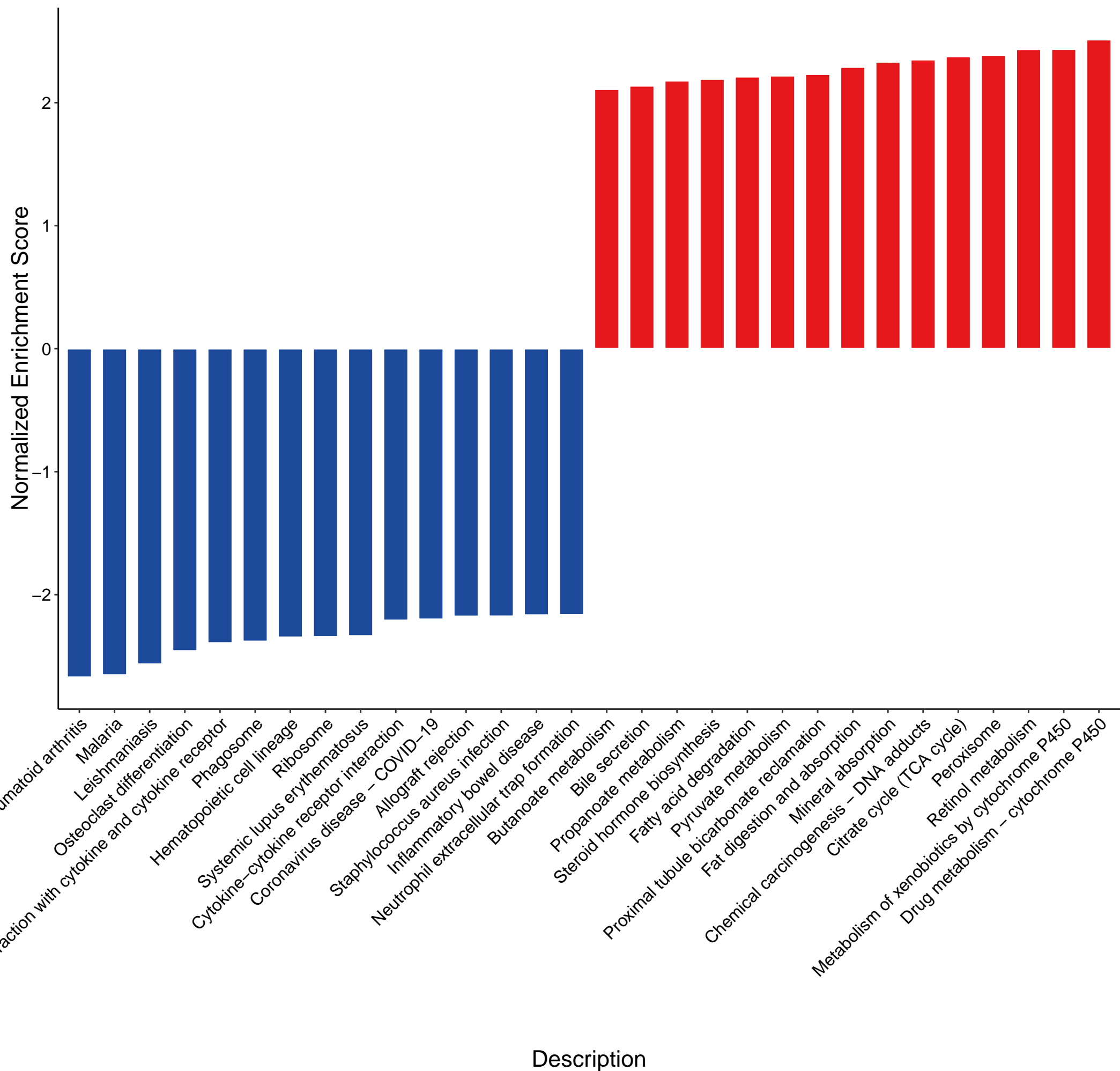

Supplement: Supplementary file 1 [file Supplementaryfile1.zip › Supplementary Material/04_Immune/3.3_CD_ABO_ssGSEA__GSEA_KEGG.pdf]

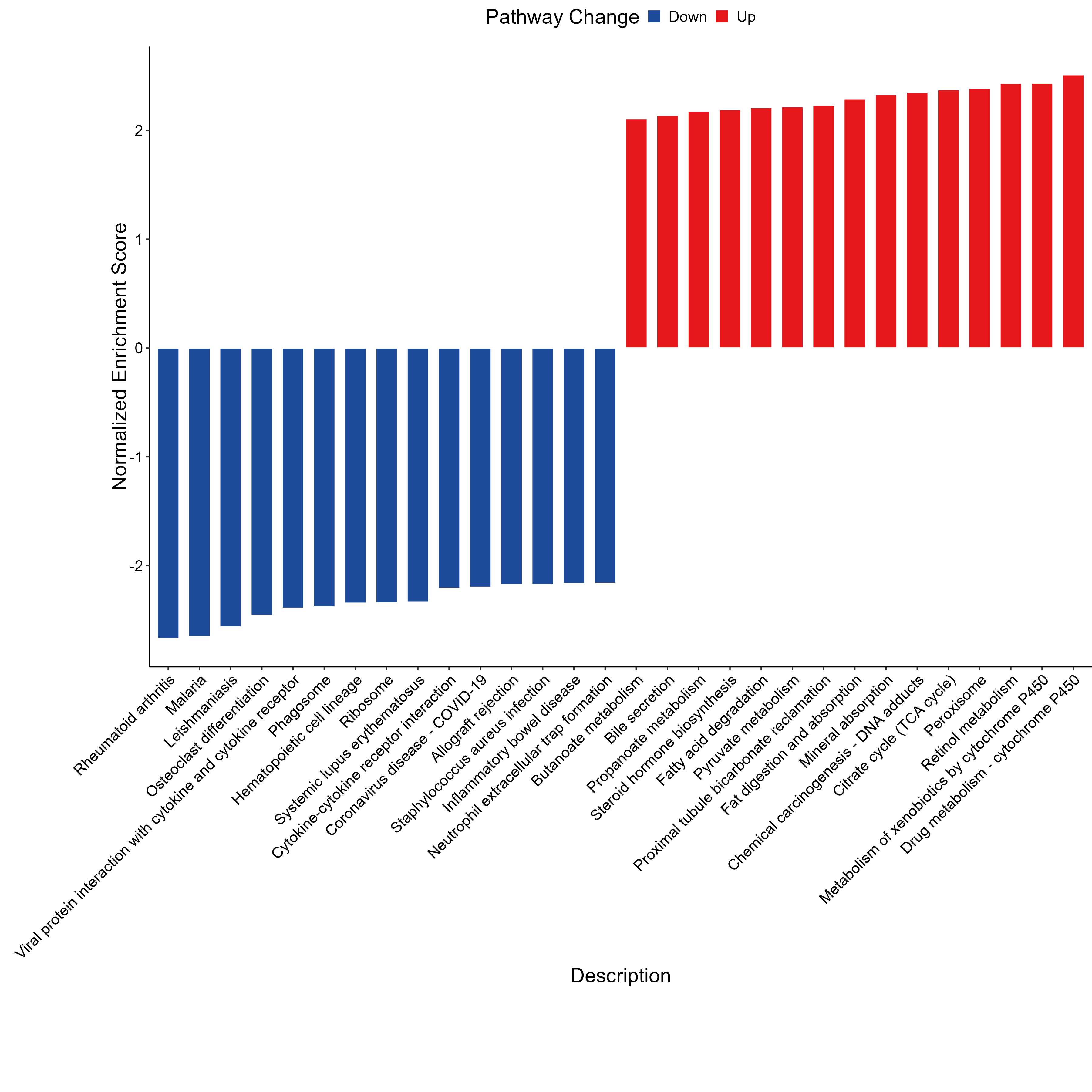

Supplement: Supplementary file 1 [file Supplementaryfile1.zip › Supplementary Material/04_Immune/3.3_CD_ABO_ssGSEA__GSEA_KEGG.png]

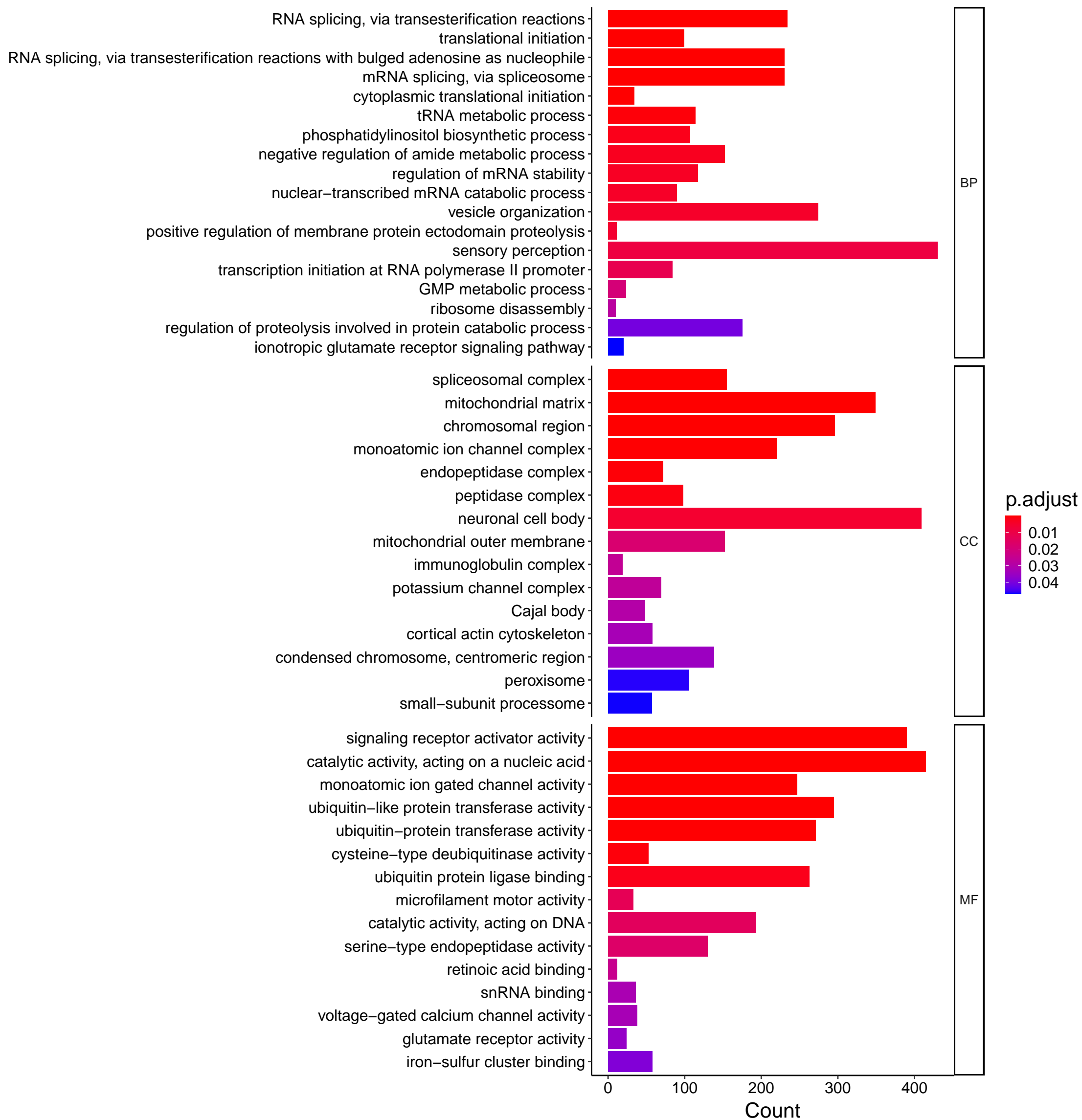

Supplement: Supplementary file 1 [file Supplementaryfile1.zip › Supplementary Material/04_Immune/3.3_OP_ABO_ssGSEA_GO_barplot.pdf.pdf]

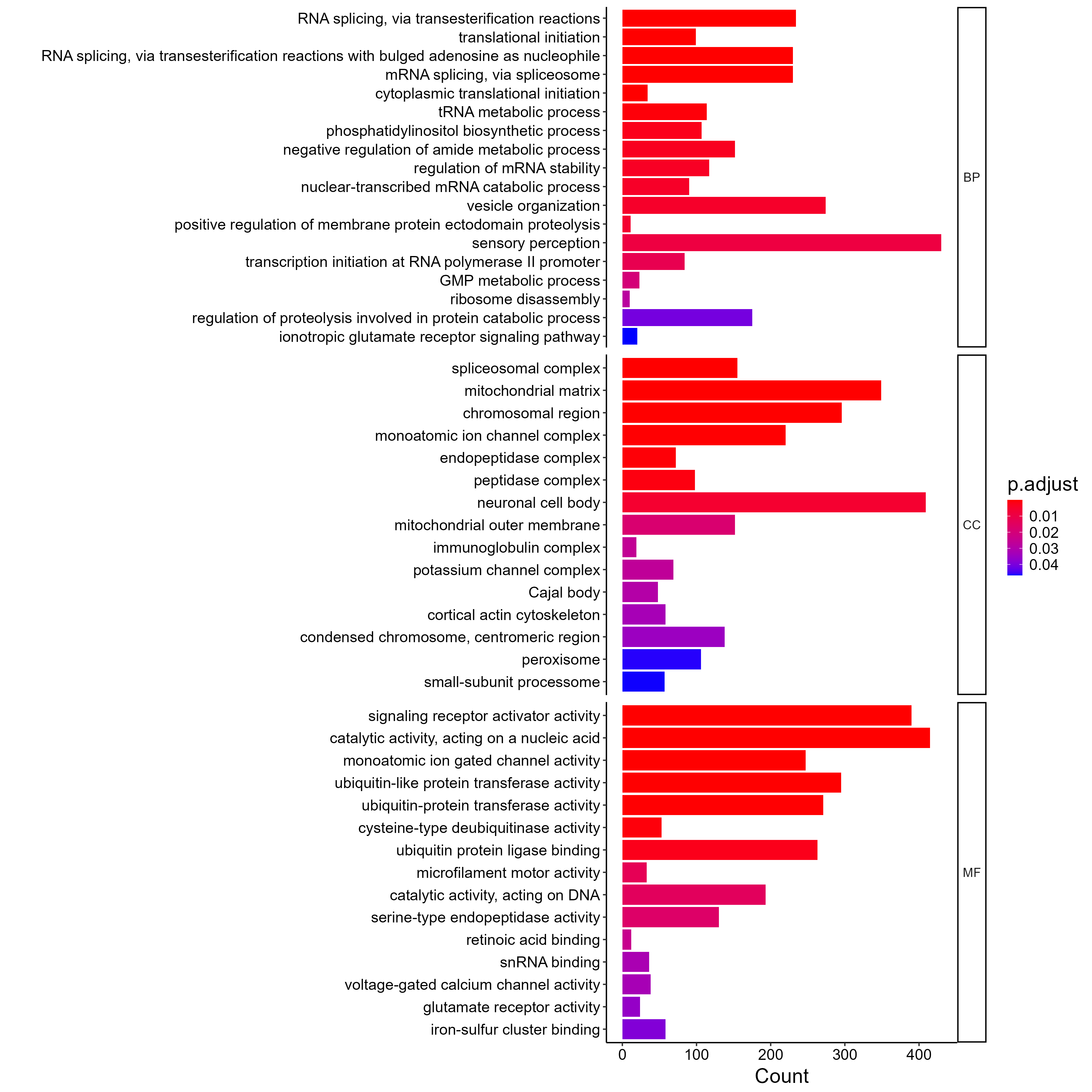

Supplement: Supplementary file 1 [file Supplementaryfile1.zip › Supplementary Material/04_Immune/3.3_OP_ABO_ssGSEA_GO_barplot.pdf.png]

Pathway Change    Down    Up

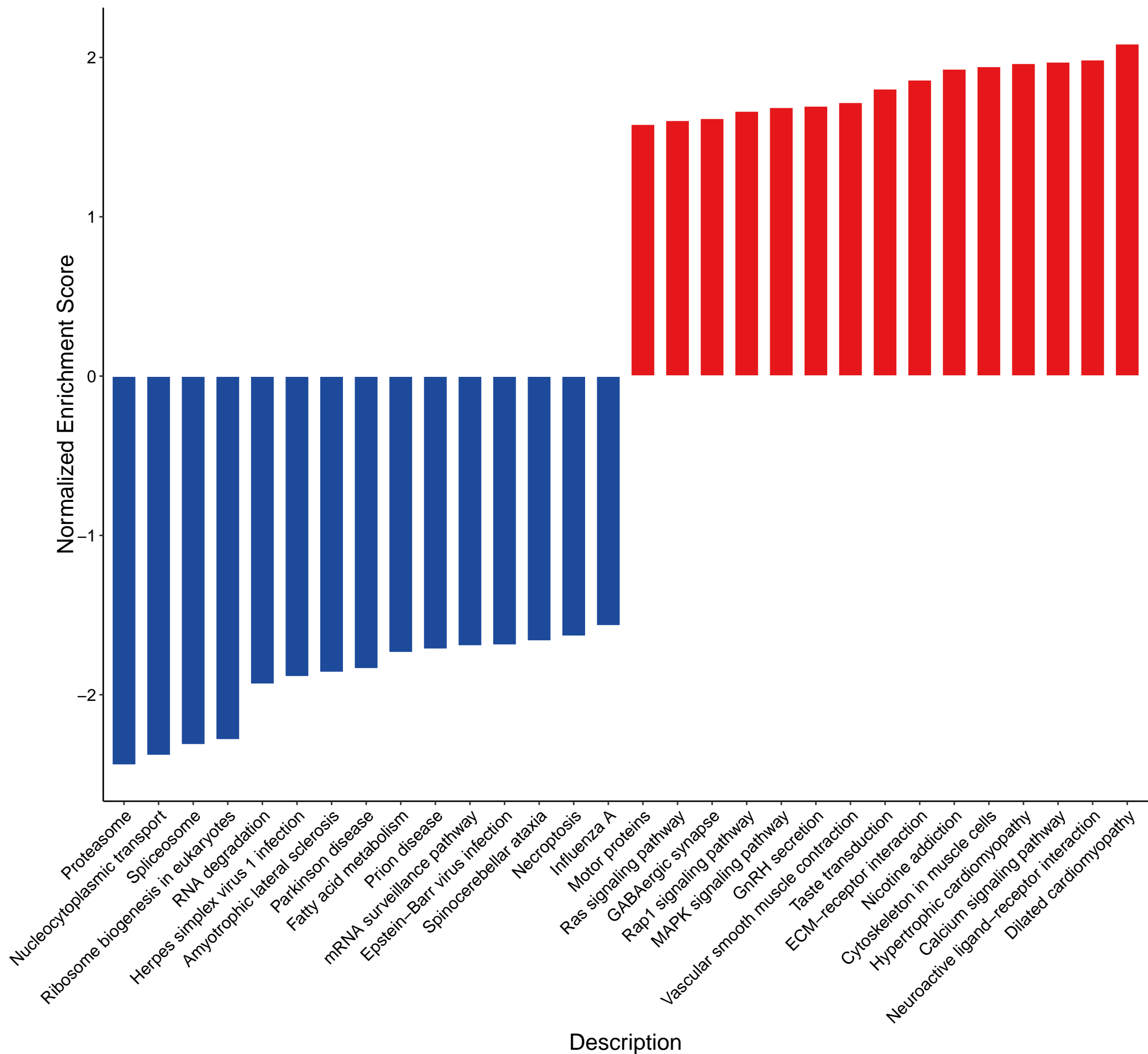

Supplement: Supplementary file 1 [file Supplementaryfile1.zip › Supplementary Material/04_Immune/3.5_OP_ABO_ssGSEA__GSEA_KEGG.pdf]

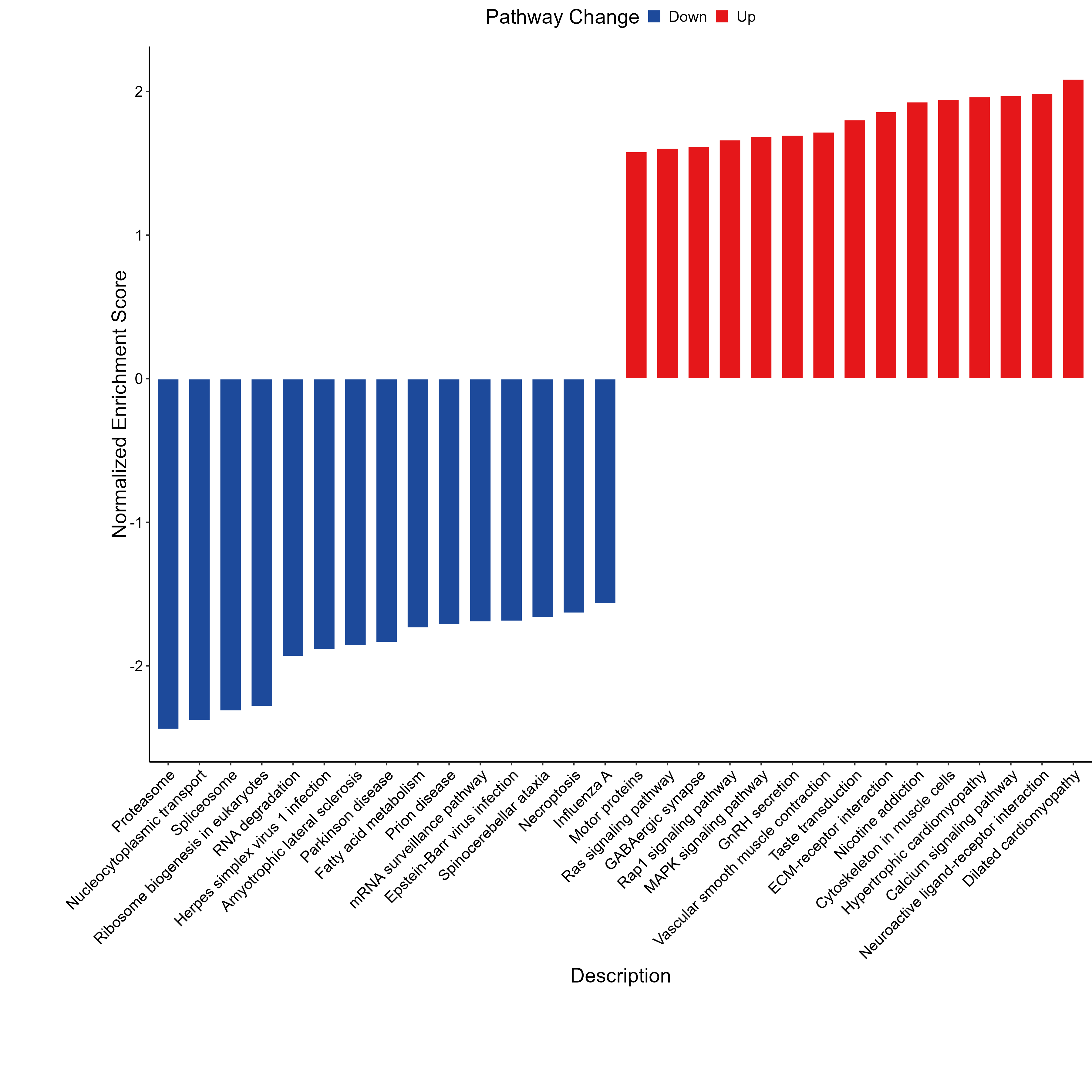

Supplement: Supplementary file 1 [file Supplementaryfile1.zip › Supplementary Material/04_Immune/3.5_OP_ABO_ssGSEA__GSEA_KEGG.png]

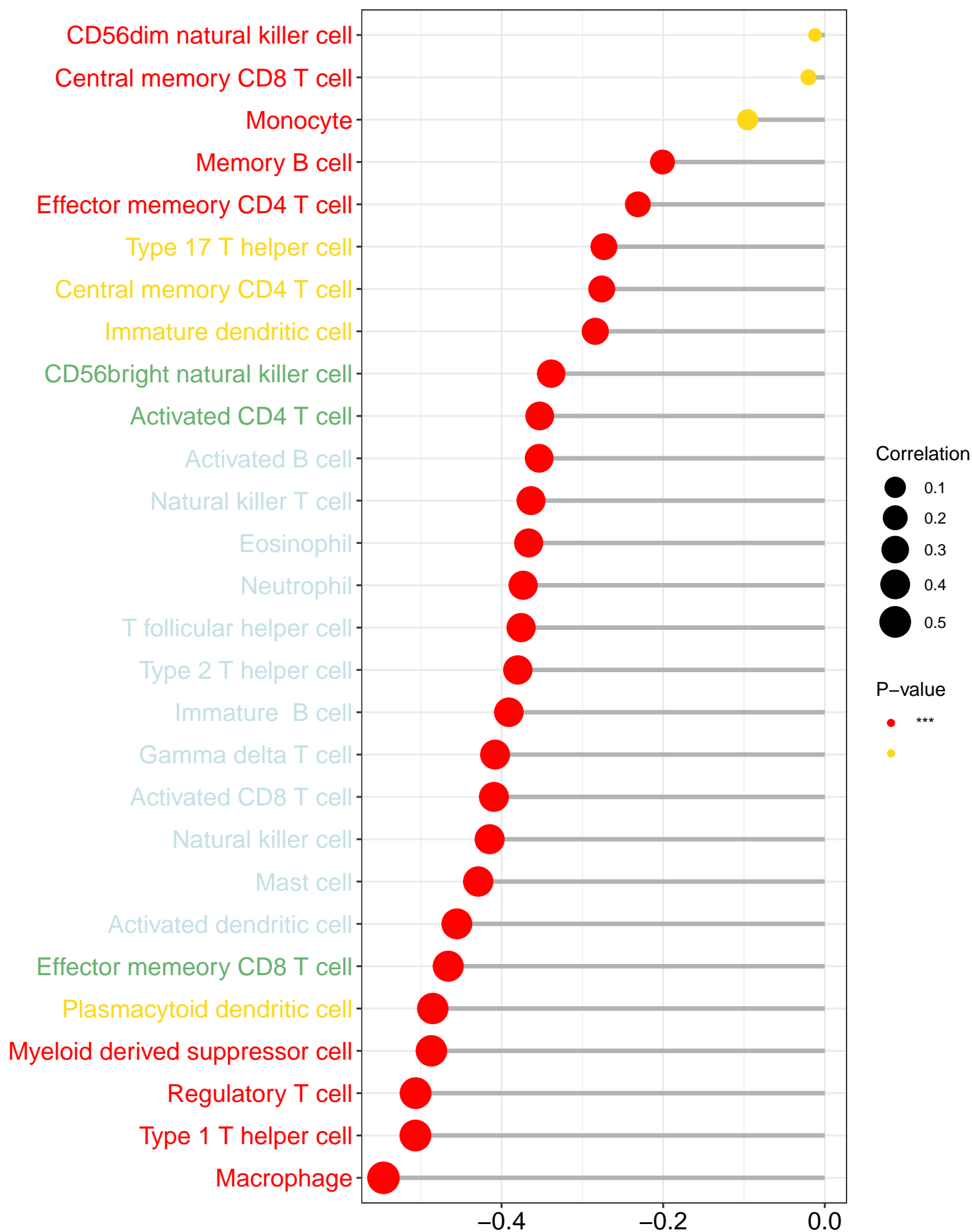

Supplement: Supplementary file 1 [file Supplementaryfile1.zip › Supplementary Material/04_Immune/ABO_Correlation_with_Immune_All.pdf]

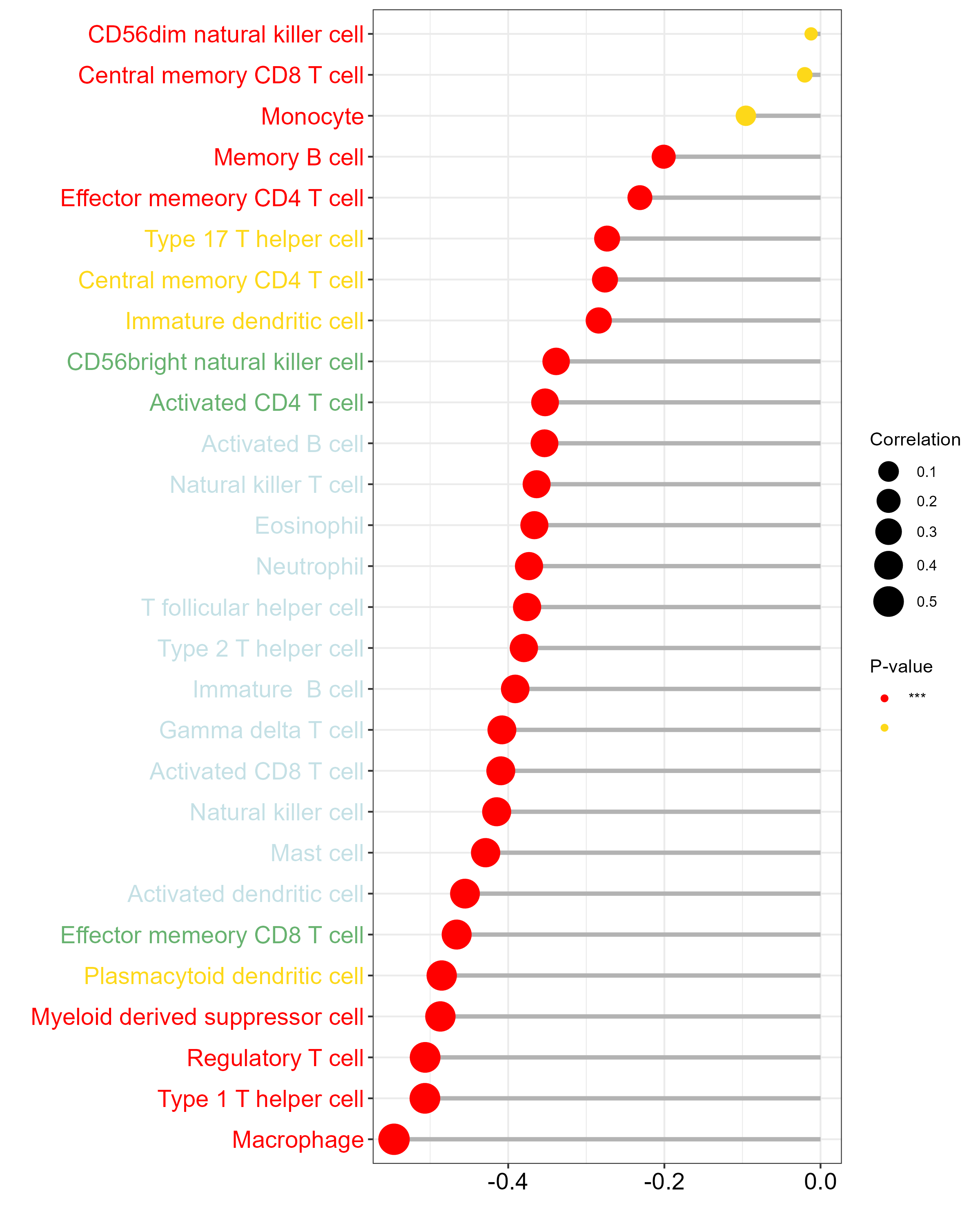

Supplement: Supplementary file 1 [file Supplementaryfile1.zip › Supplementary Material/04_Immune/ABO_Correlation_with_Immune_All.png]

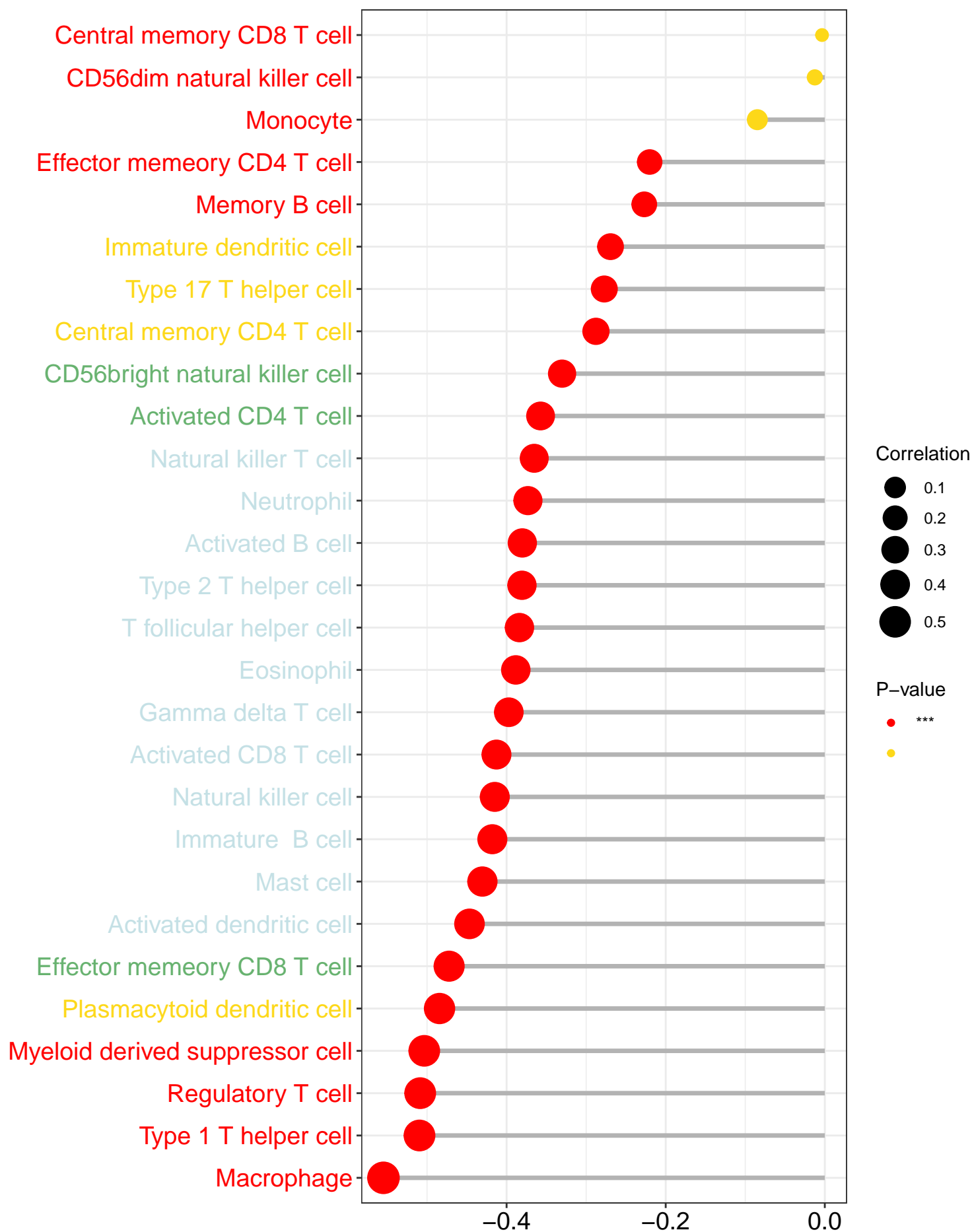

Supplement: Supplementary file 1 [file Supplementaryfile1.zip › Supplementary Material/04_Immune/ABO_Correlation_with_Immune_CD.pdf]

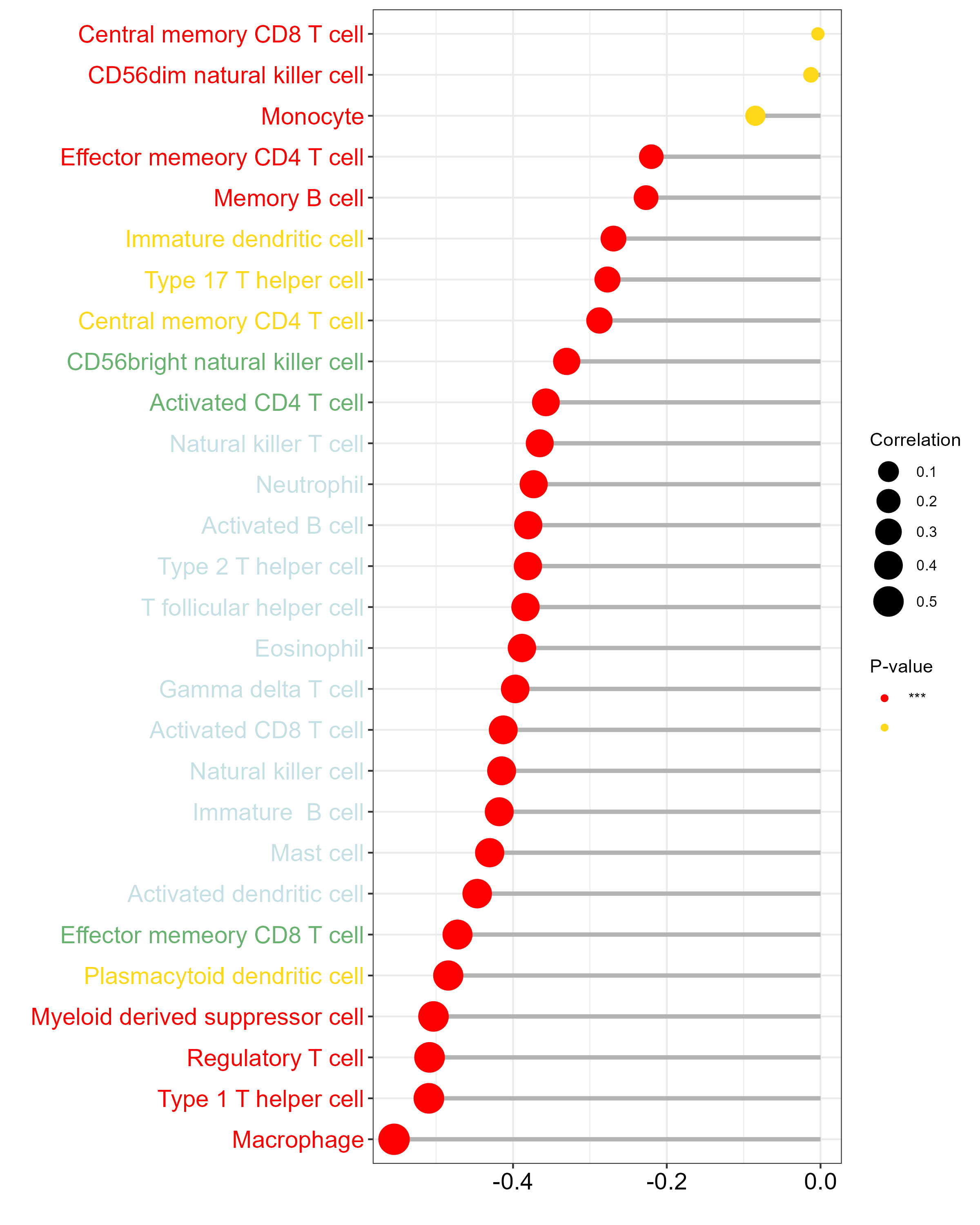

Supplement: Supplementary file 1 [file Supplementaryfile1.zip › Supplementary Material/04_Immune/ABO_Correlation_with_Immune_CD.png]

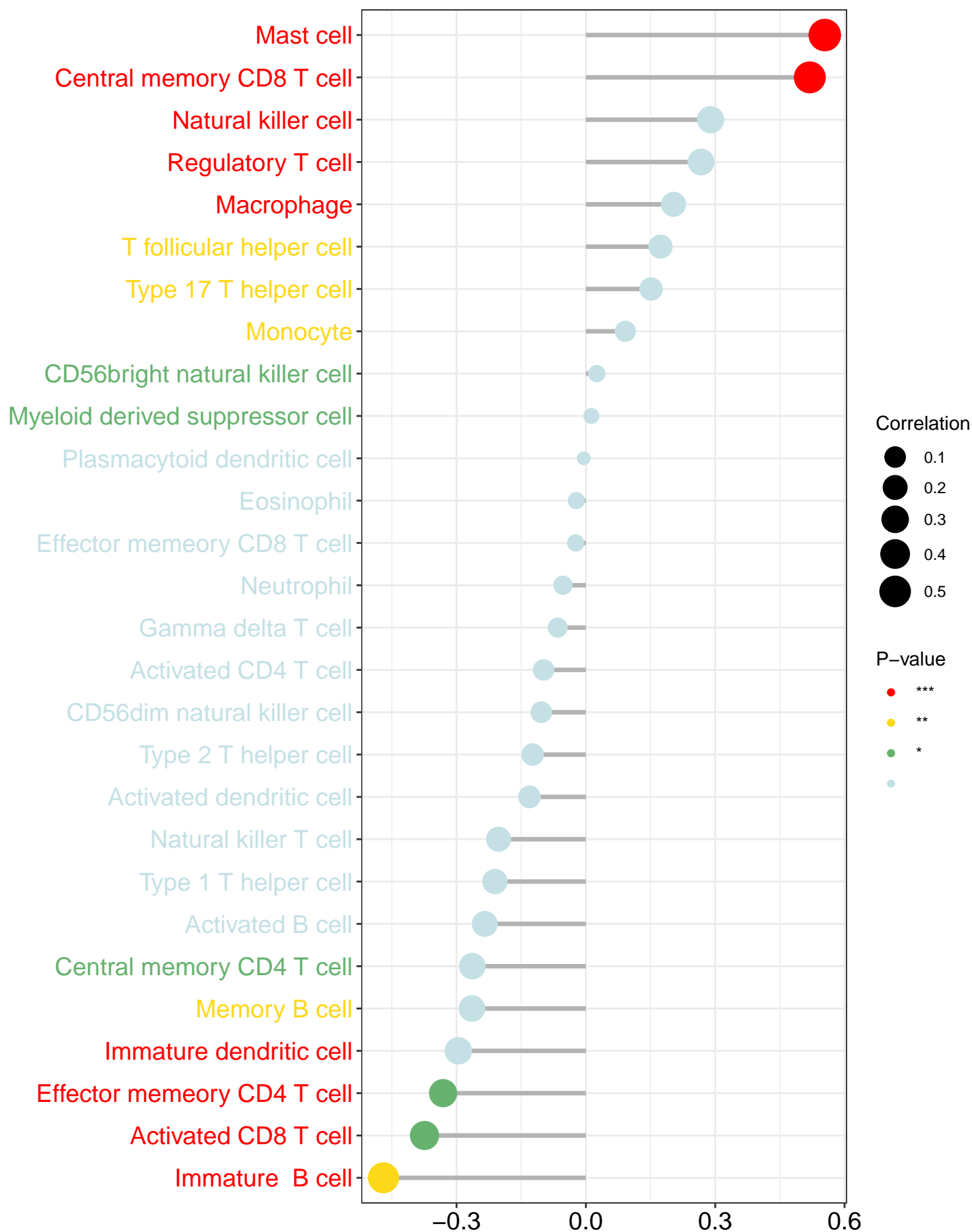

Supplement: Supplementary file 1 [file Supplementaryfile1.zip › Supplementary Material/04_Immune/ABO_Correlation_with_Immune_Low_BMD.pdf]

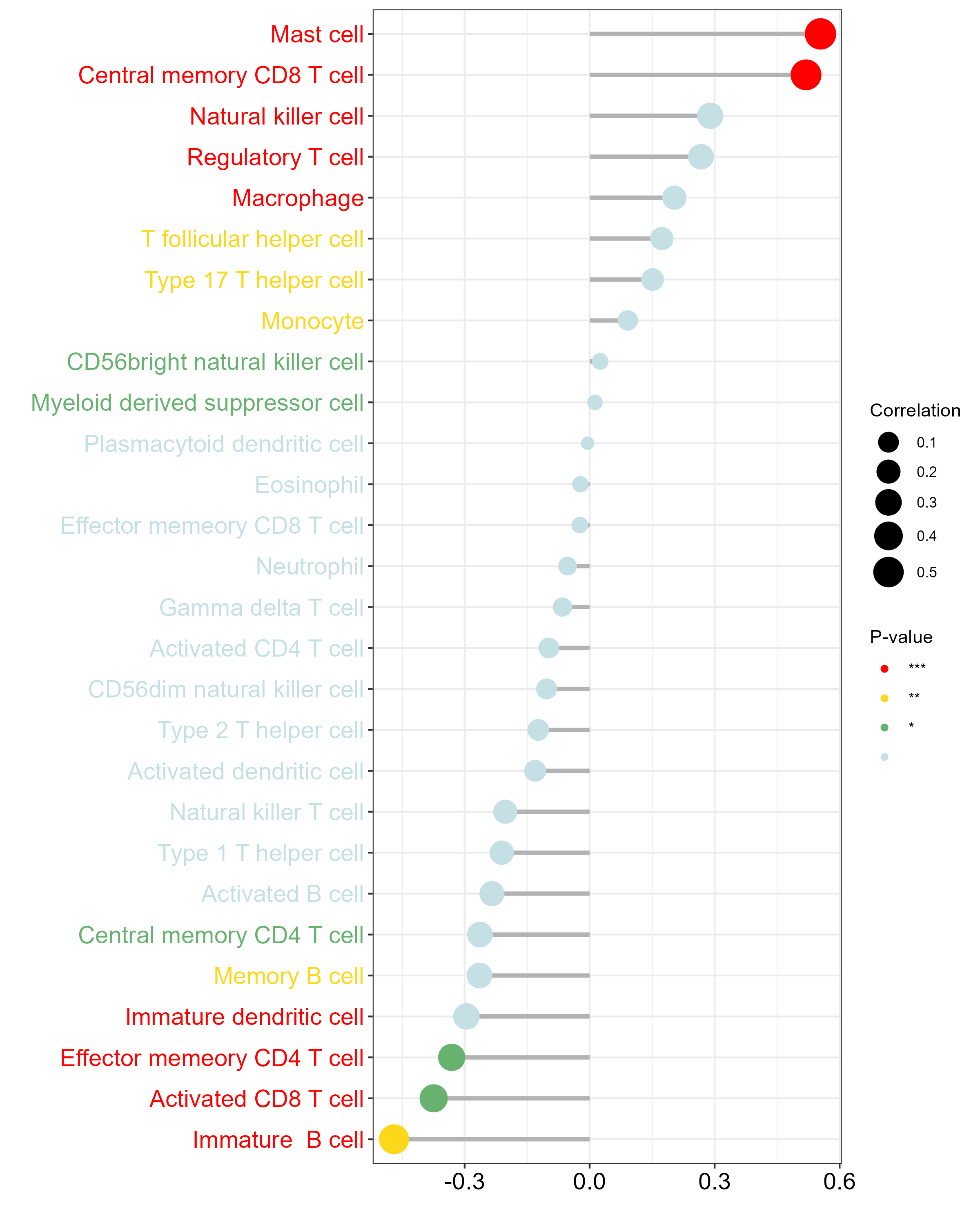

Supplement: Supplementary file 1 [file Supplementaryfile1.zip › Supplementary Material/04_Immune/ABO_Correlation_with_Immune_Low_BMD.png]

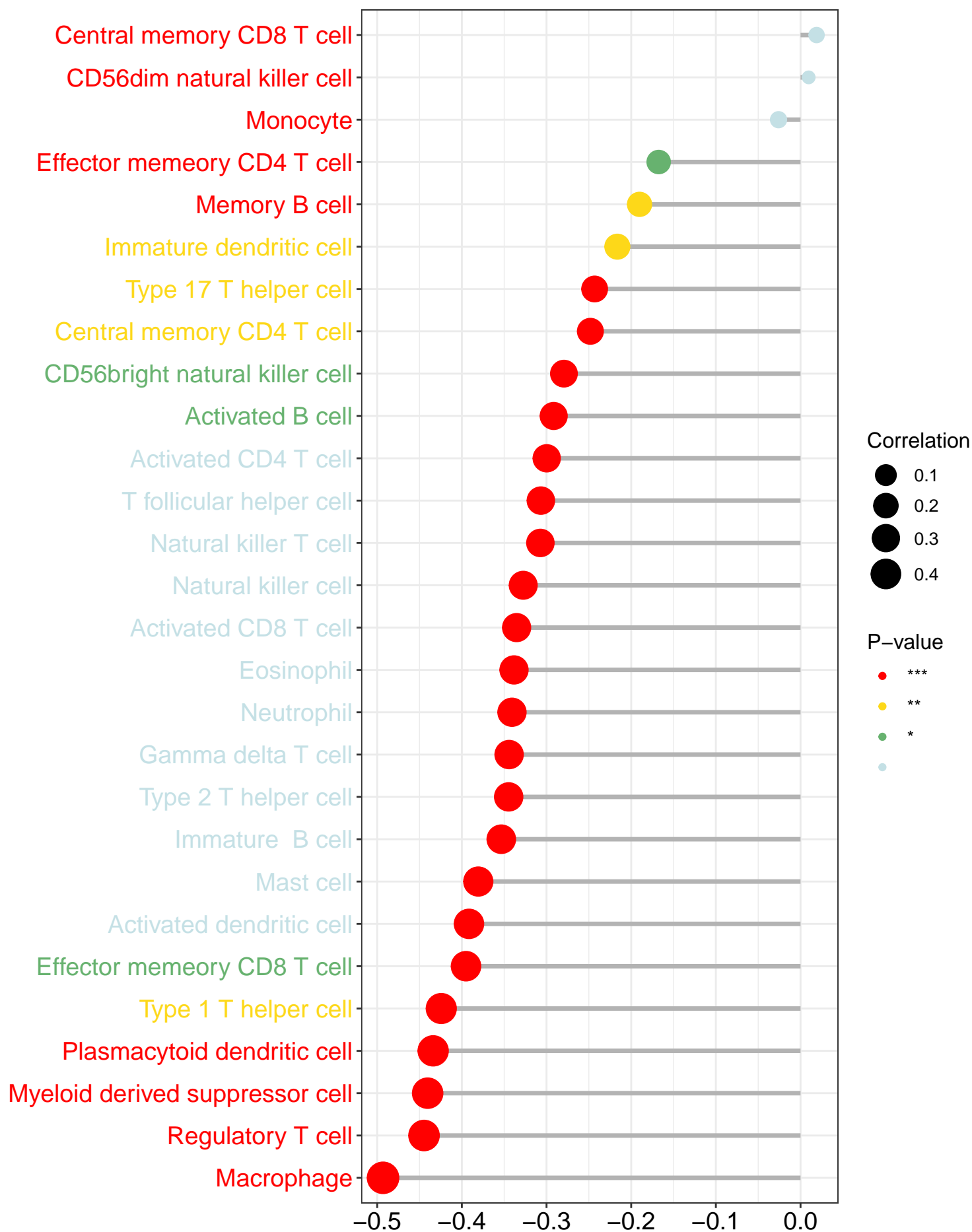

Supplement: Supplementary file 1 [file Supplementaryfile1.zip › Supplementary Material/04_Immune/ABO_Correlation_with_Immune_Treatment.pdf]

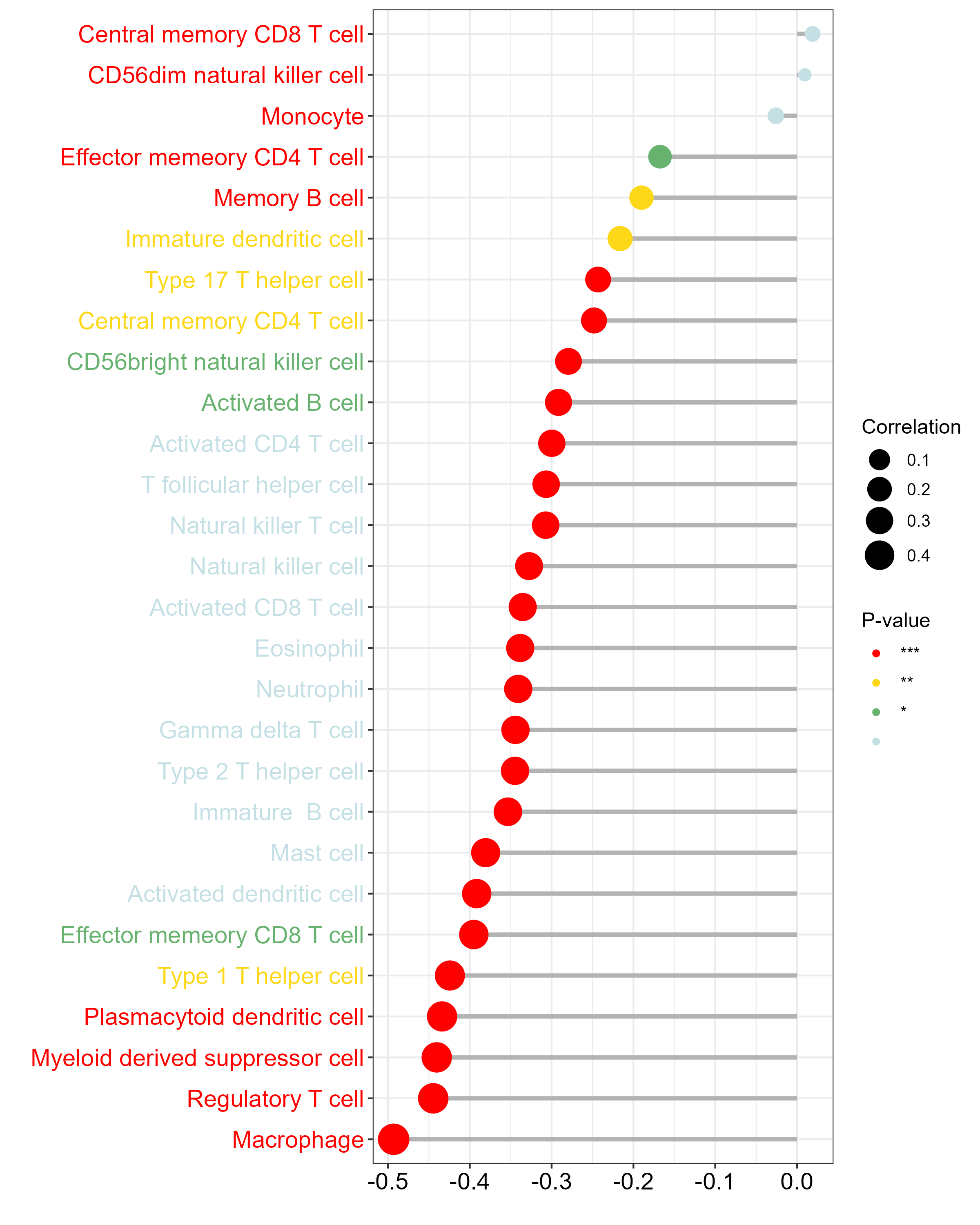

Supplement: Supplementary file 1 [file Supplementaryfile1.zip › Supplementary Material/04_Immune/ABO_Correlation_with_Immune_Treatment.png]

**Subgroup** ■ Normal ■ Treatment

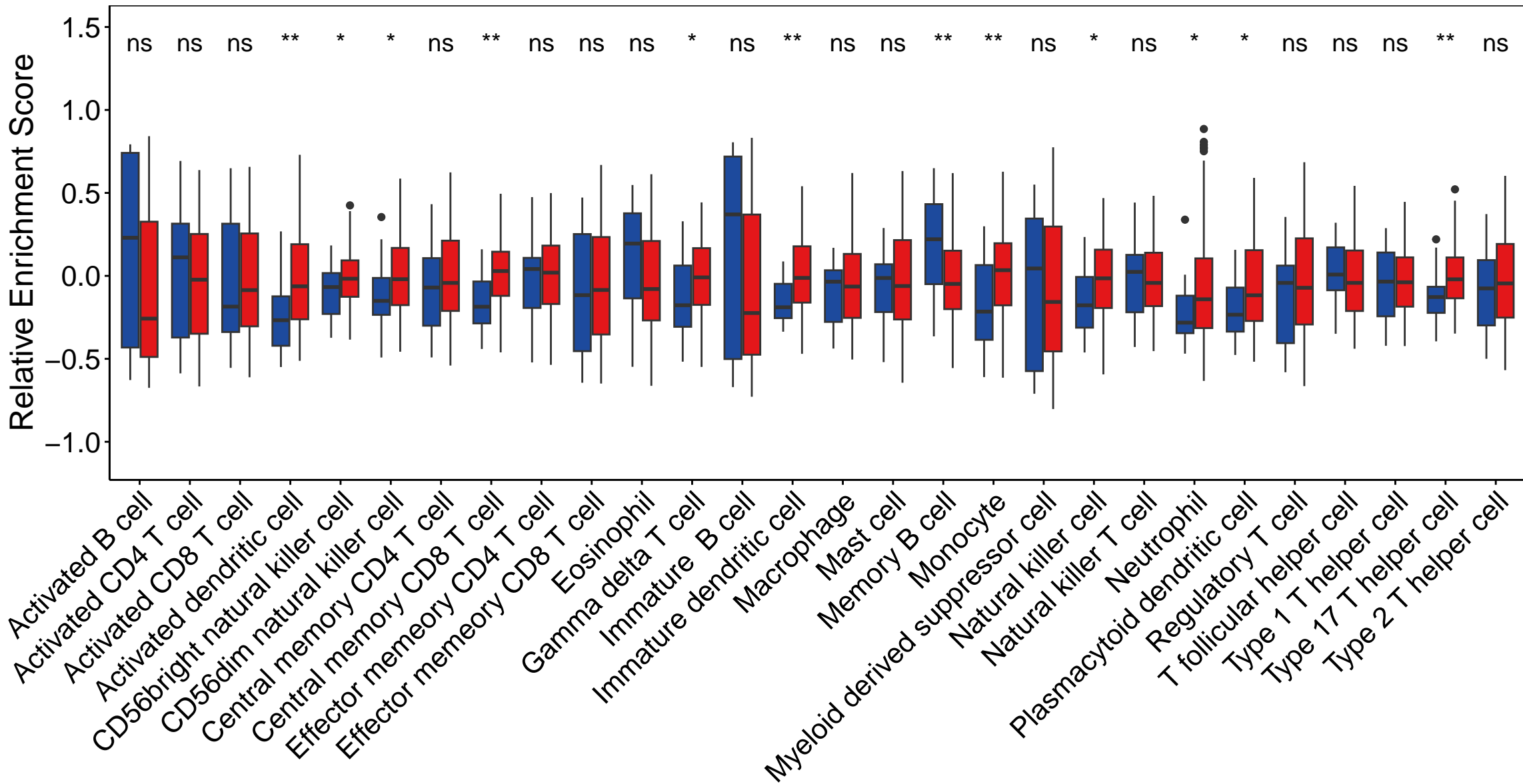

Supplement: Supplementary file 1 [file Supplementaryfile1.zip › Supplementary Material/04_Immune/CD-teratment-boxplot.pdf]

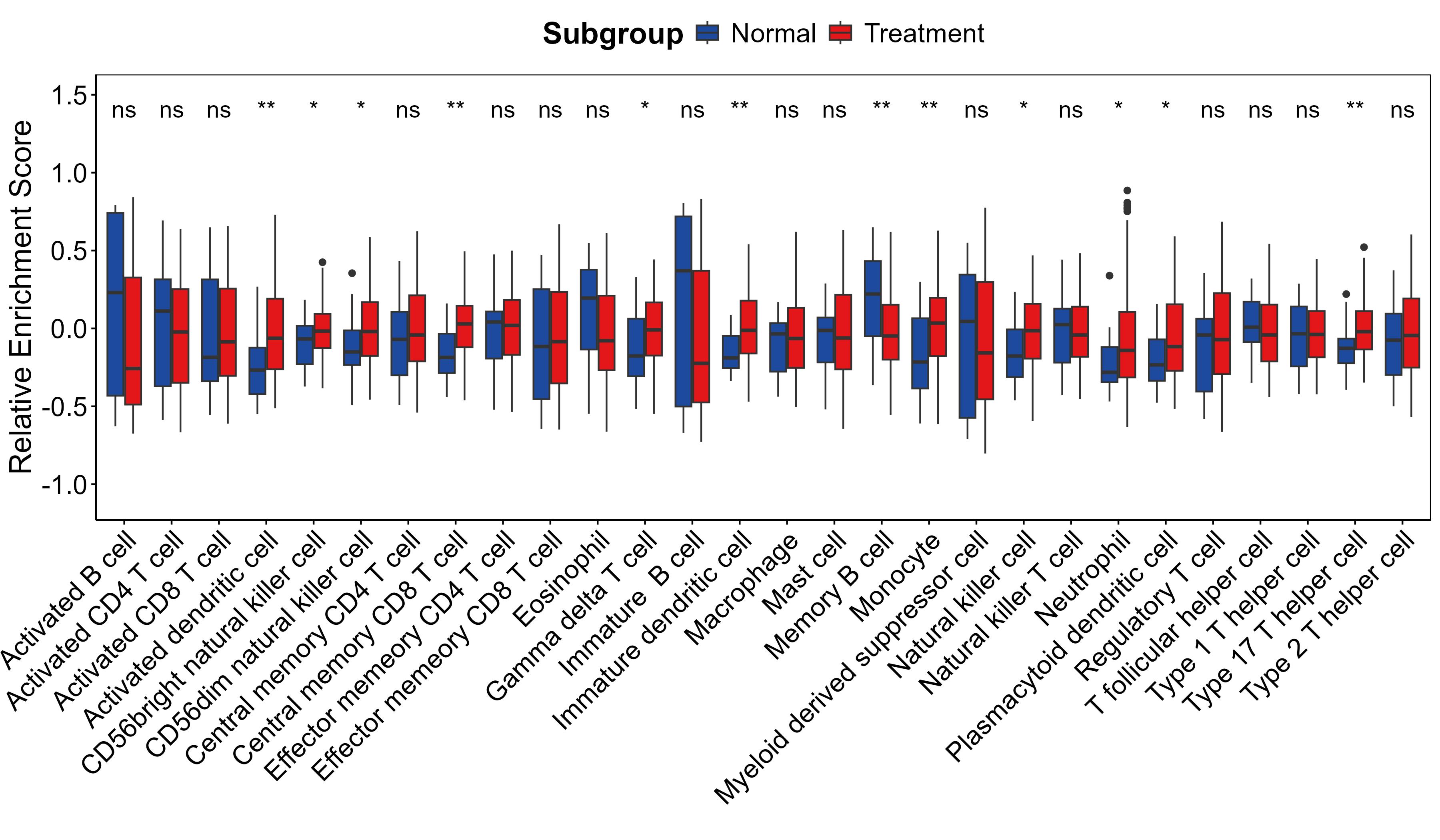

Supplement: Supplementary file 1 [file Supplementaryfile1.zip › Supplementary Material/04_Immune/CD-teratment-boxplot.png]

## ABO Train

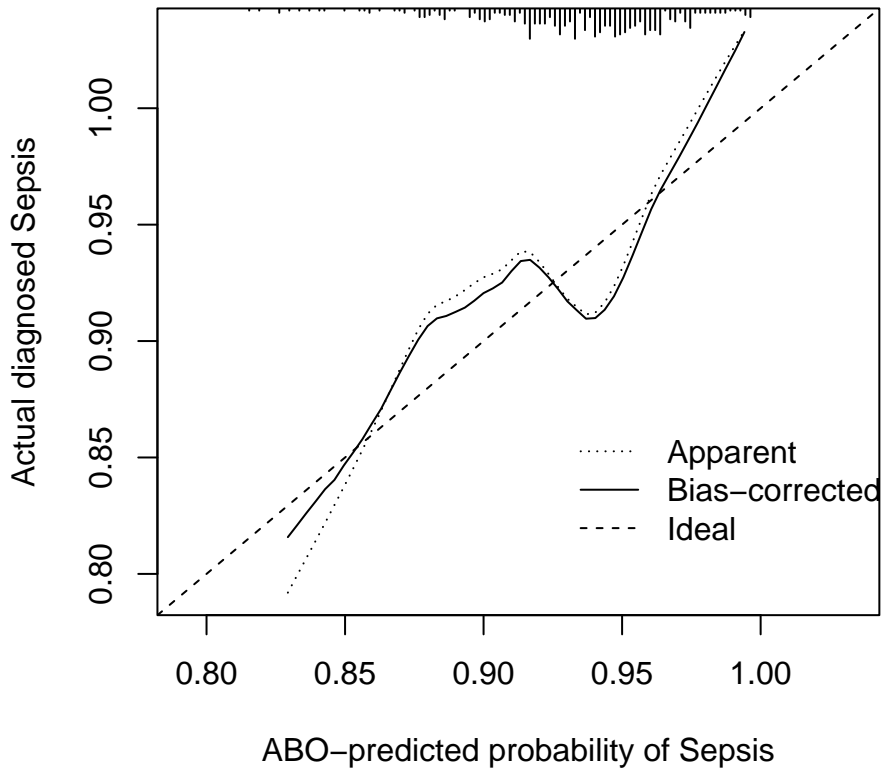

Supplement: Supplementary file 1 [file Supplementaryfile1.zip › Supplementary Material/ABO/CD/Calibration_CD_ABO_Train.pdf]

## ABO Valid

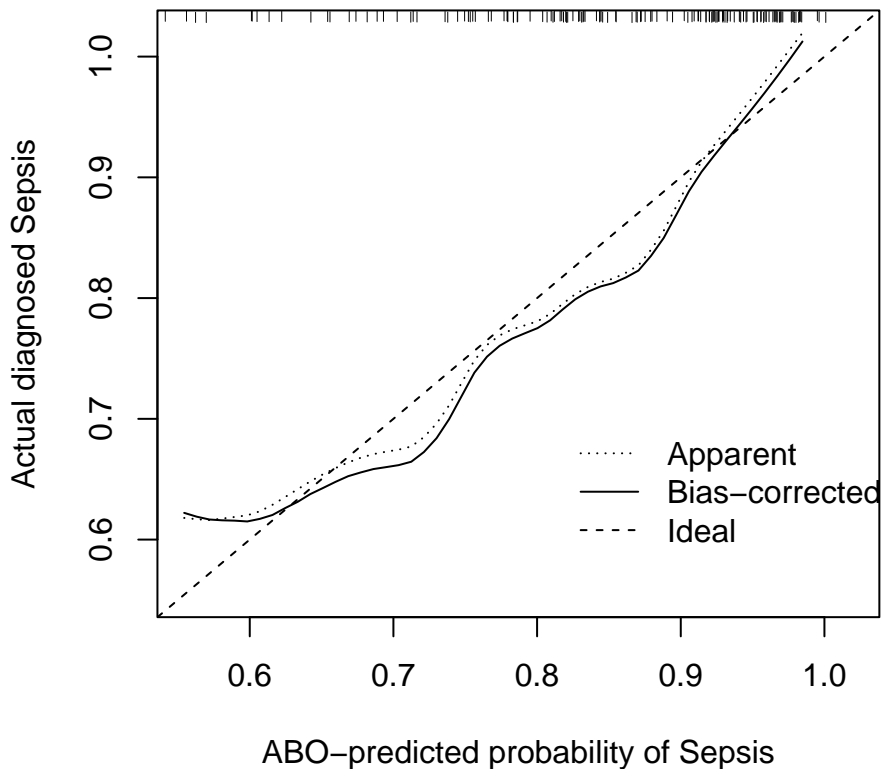

Supplement: Supplementary file 1 [file Supplementaryfile1.zip › Supplementary Material/ABO/CD/Calibration_CD_ABO_Valid.pdf]

# ABO Train

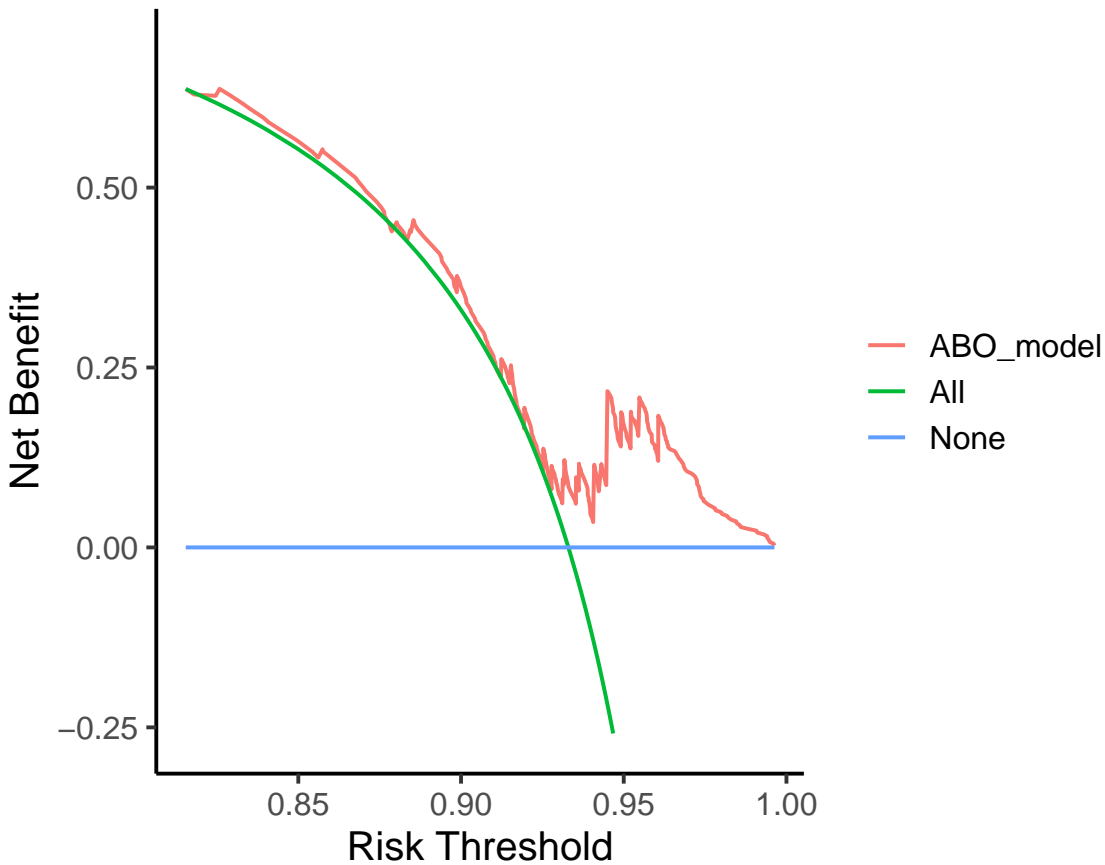

Supplement: Supplementary file 1 [file Supplementaryfile1.zip › Supplementary Material/ABO/CD/DCA_CD_ABO_Train.pdf]

# ABO Valid

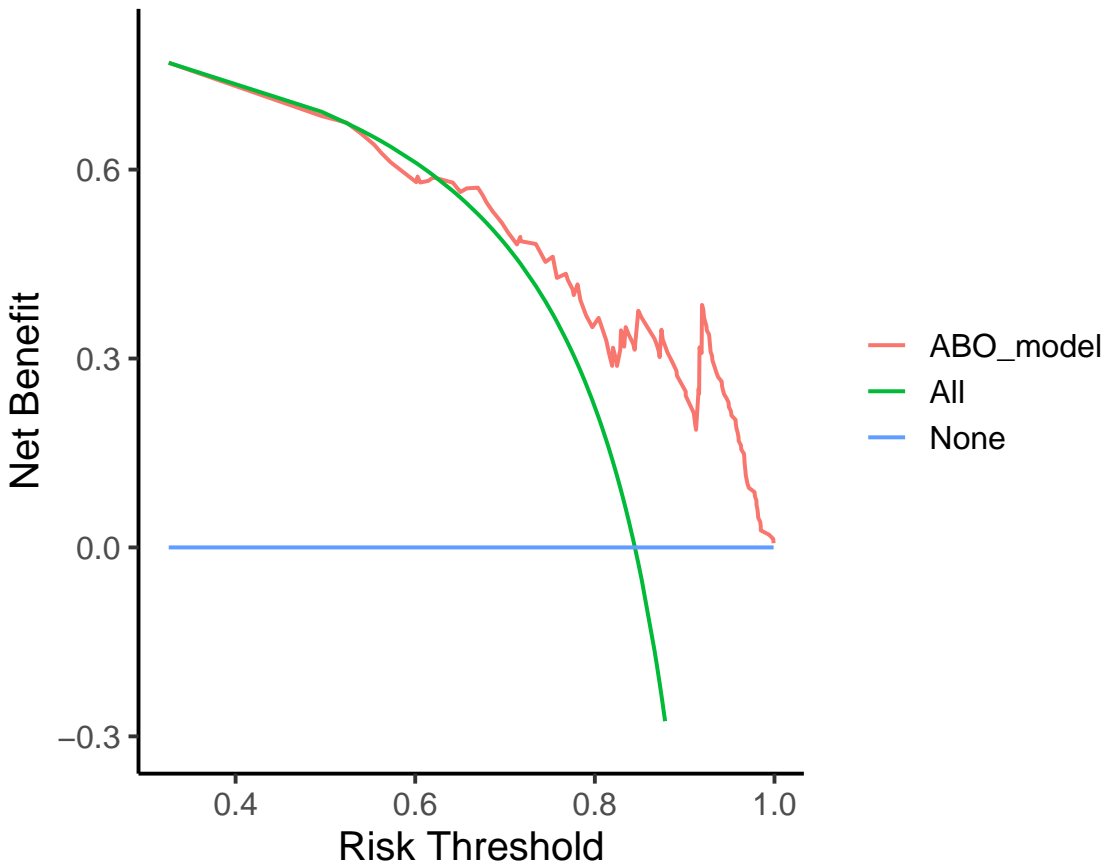

Supplement: Supplementary file 1 [file Supplementaryfile1.zip › Supplementary Material/ABO/CD/DCA_CD_ABO_Valid.pdf]

# ABO Train

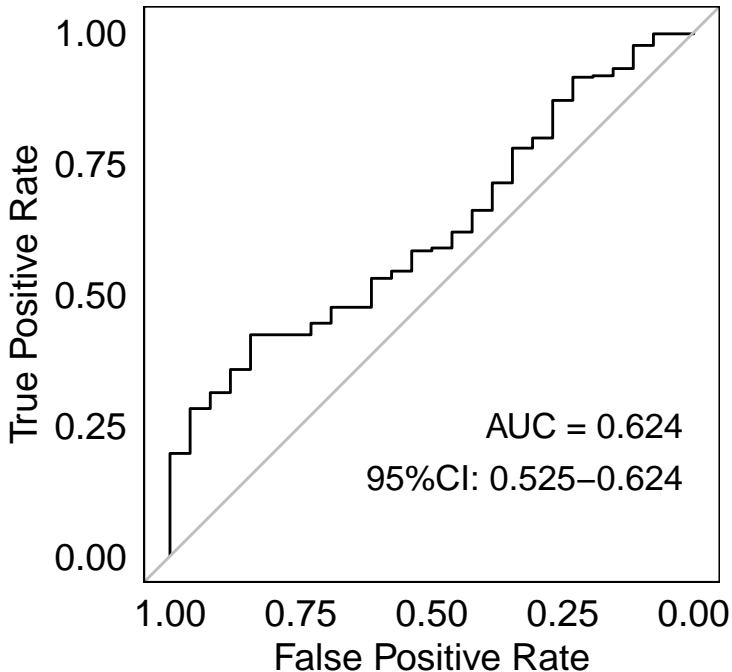

Supplement: Supplementary file 1 [file Supplementaryfile1.zip › Supplementary Material/ABO/CD/ROC_CD_ABO_Train.pdf]

# ABO Valid

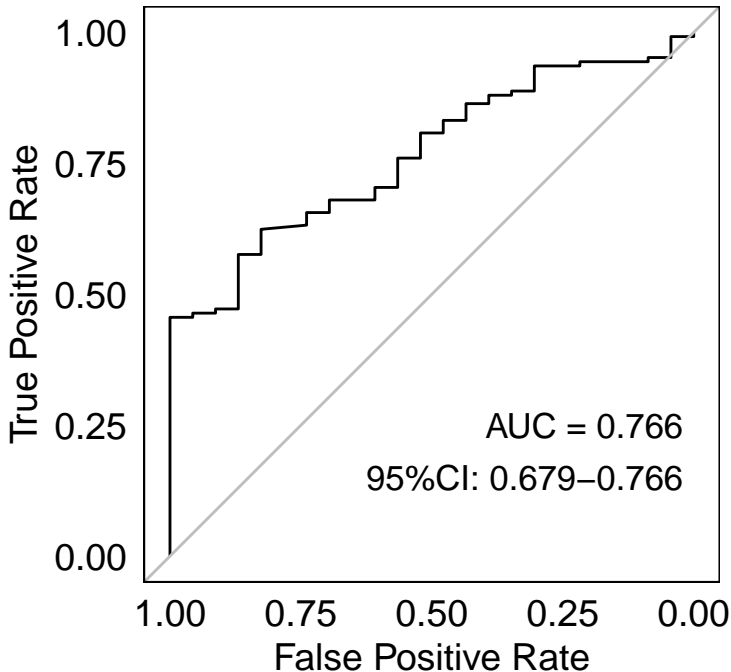

Supplement: Supplementary file 1 [file Supplementaryfile1.zip › Supplementary Material/ABO/CD/ROC_CD_ABO_Valid.pdf]

## ABO Train

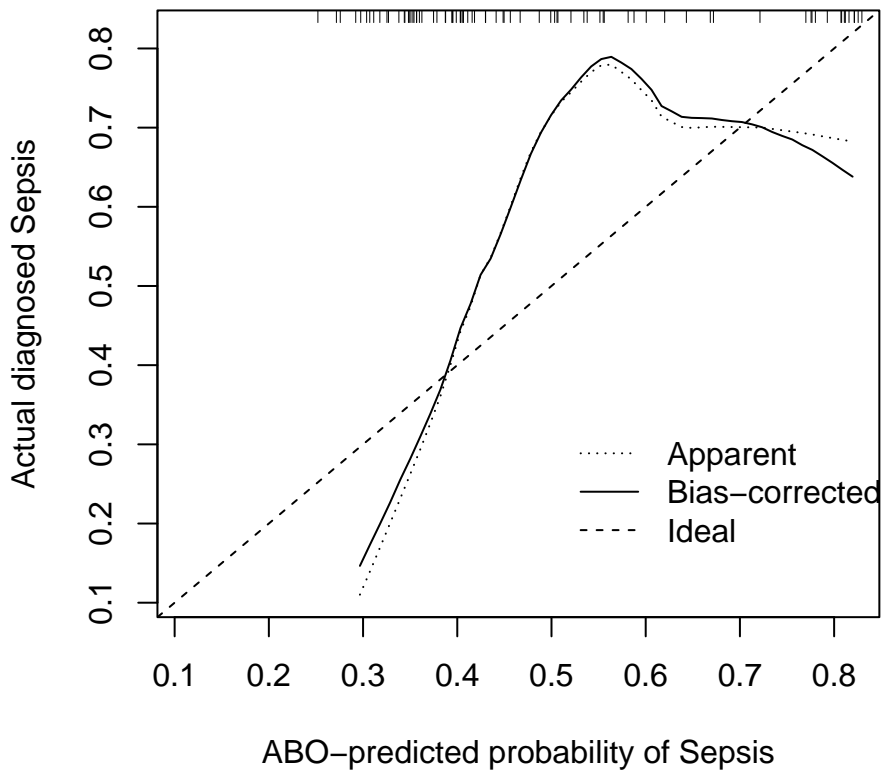

Supplement: Supplementary file 1 [file Supplementaryfile1.zip › Supplementary Material/ABO/OP/Calibration_OP_ABO_Train.pdf]

## ABO Valid

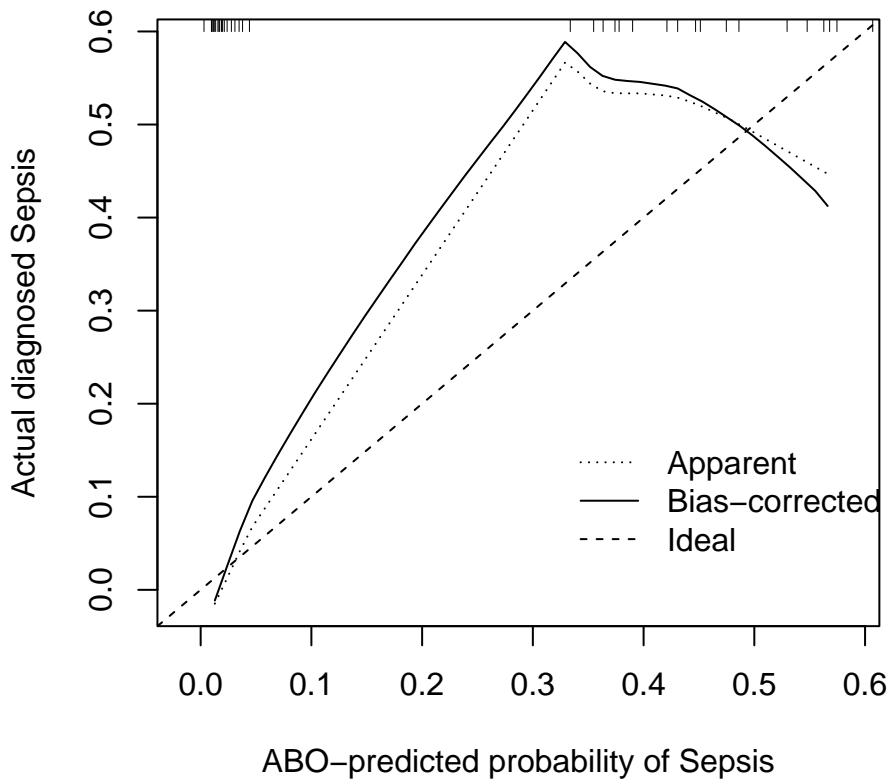

Supplement: Supplementary file 1 [file Supplementaryfile1.zip › Supplementary Material/ABO/OP/Calibration_OP_ABO_Valid.pdf]

# ABO Train

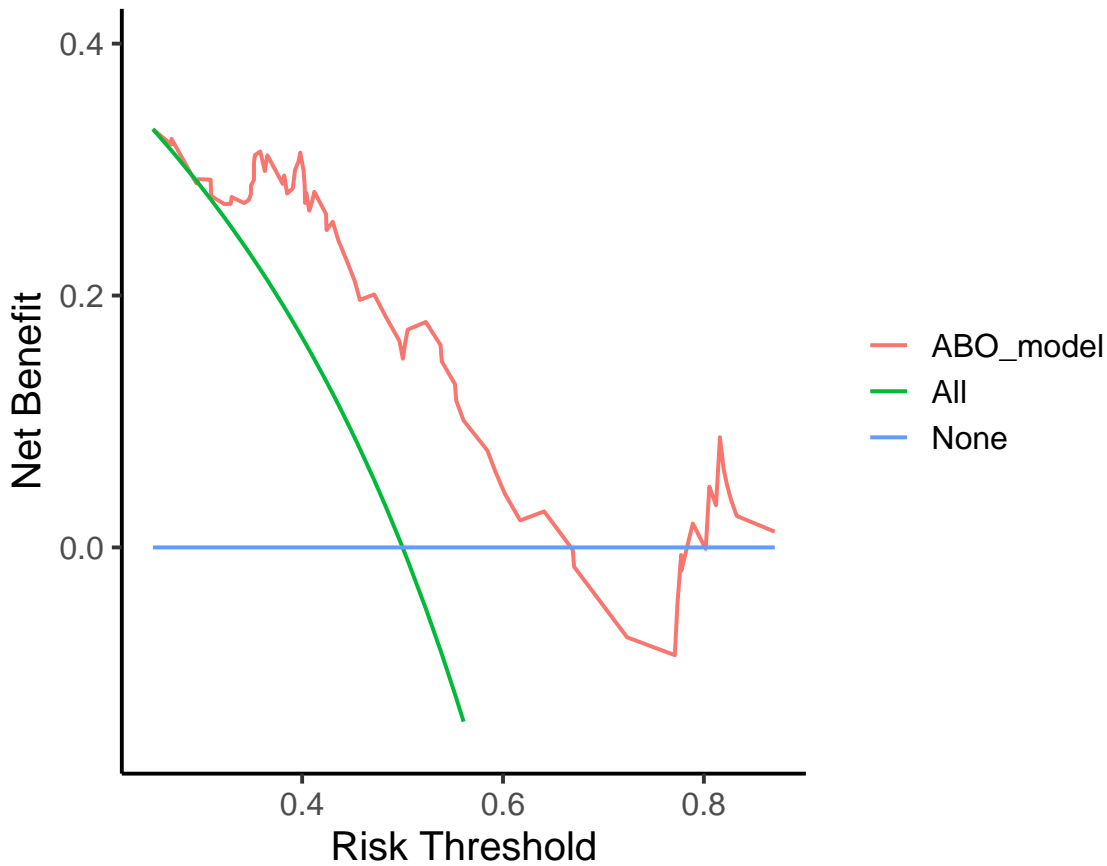

Supplement: Supplementary file 1 [file Supplementaryfile1.zip › Supplementary Material/ABO/OP/DCA_OP_ABO_Train.pdf]

# ABO Valid

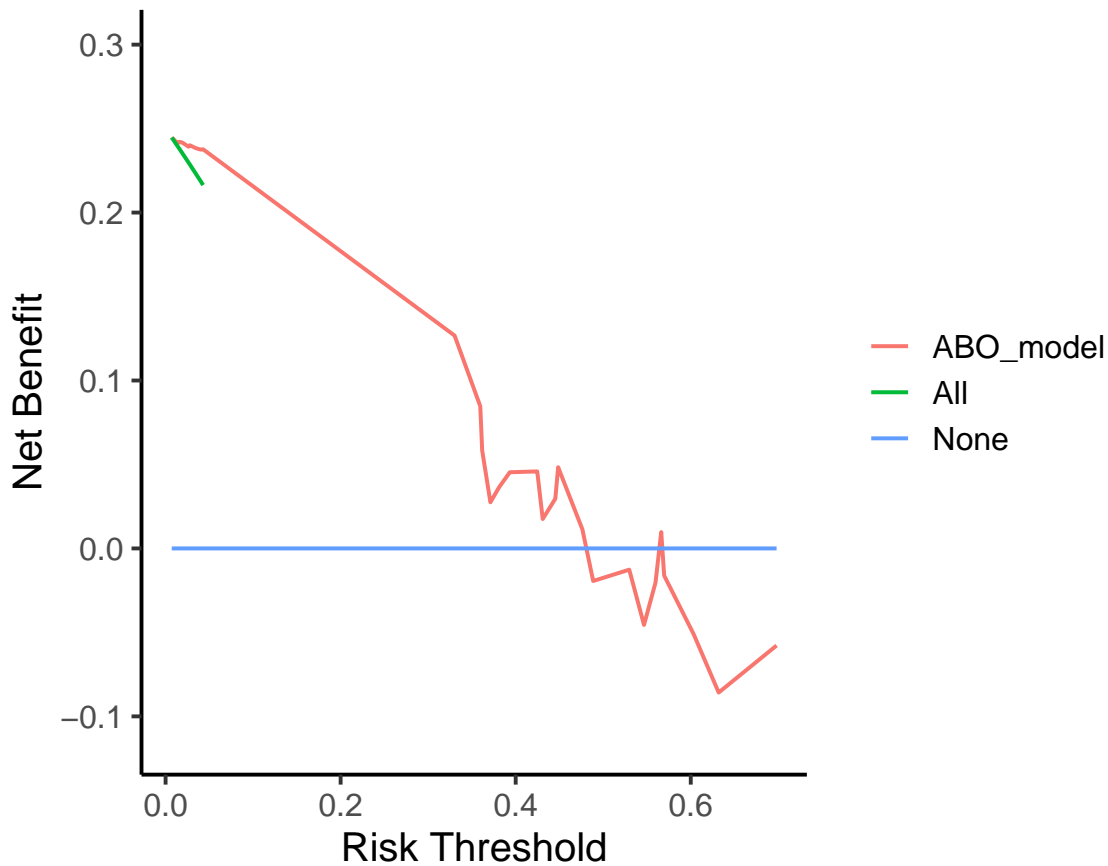

Supplement: Supplementary file 1 [file Supplementaryfile1.zip › Supplementary Material/ABO/OP/DCA_OP_ABO_Valid.pdf]

# ABO Train

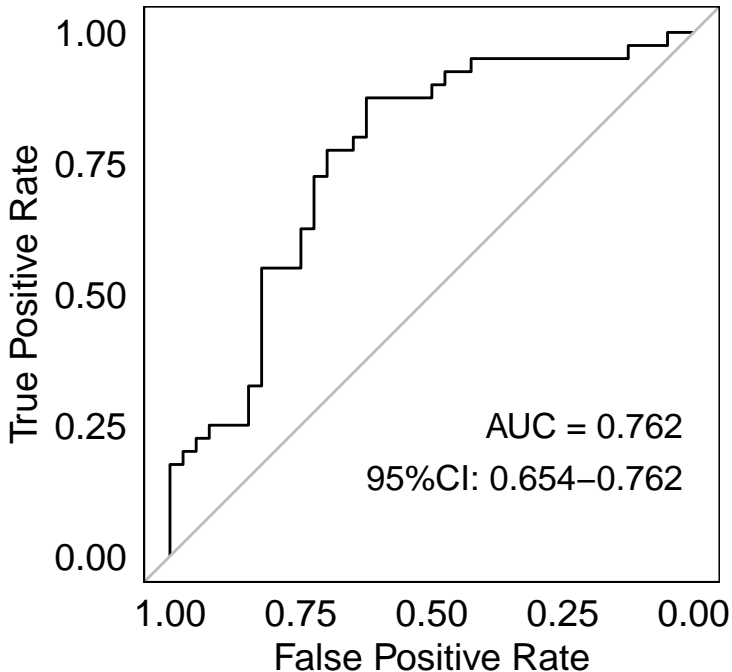

Supplement: Supplementary file 1 [file Supplementaryfile1.zip › Supplementary Material/ABO/OP/ROC_OP_ABO_Train.pdf]

# ABO Valid

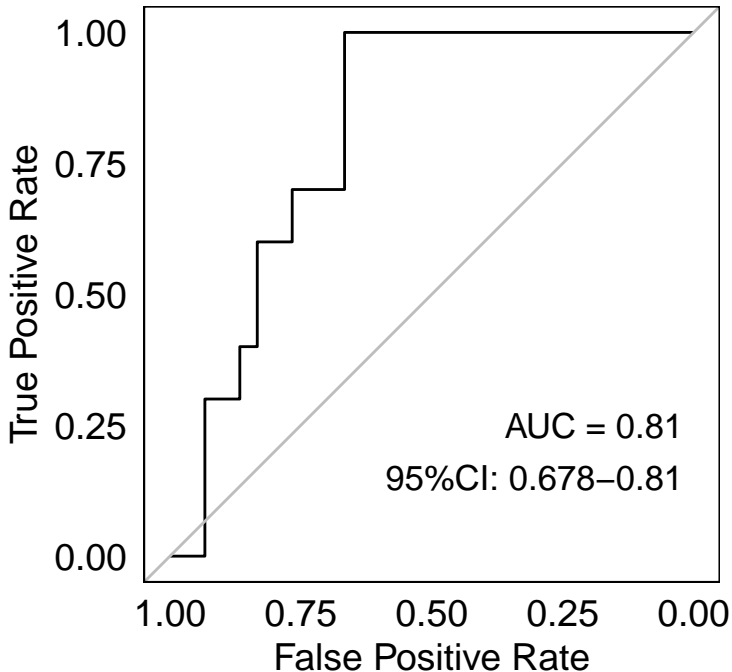

Supplement: Supplementary file 1 [file Supplementaryfile1.zip › Supplementary Material/ABO/OP/ROC_OP_ABO_Valid.pdf]

## CD Nomogram Train

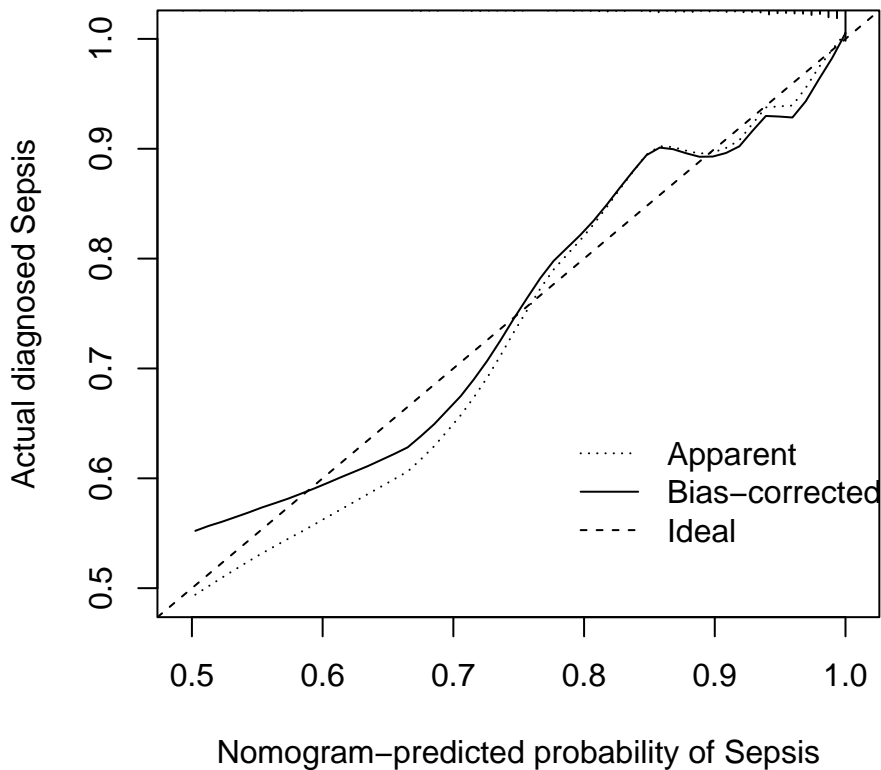

Supplement: Supplementary file 1 [file Supplementaryfile1.zip › Supplementary Material/Nomogram/CD/Calibration_CD_Nomogram_Train.pdf]

## CD Nomogram Valid

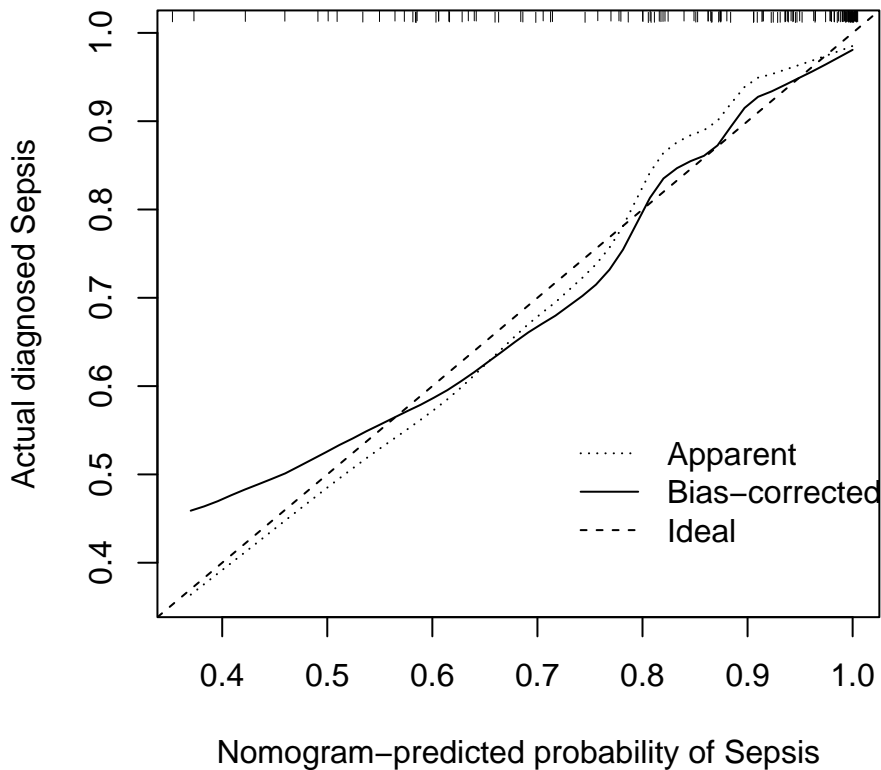

Supplement: Supplementary file 1 [file Supplementaryfile1.zip › Supplementary Material/Nomogram/CD/Calibration_CD_Nomogram_Valid.pdf]

# CD Nomogram Train

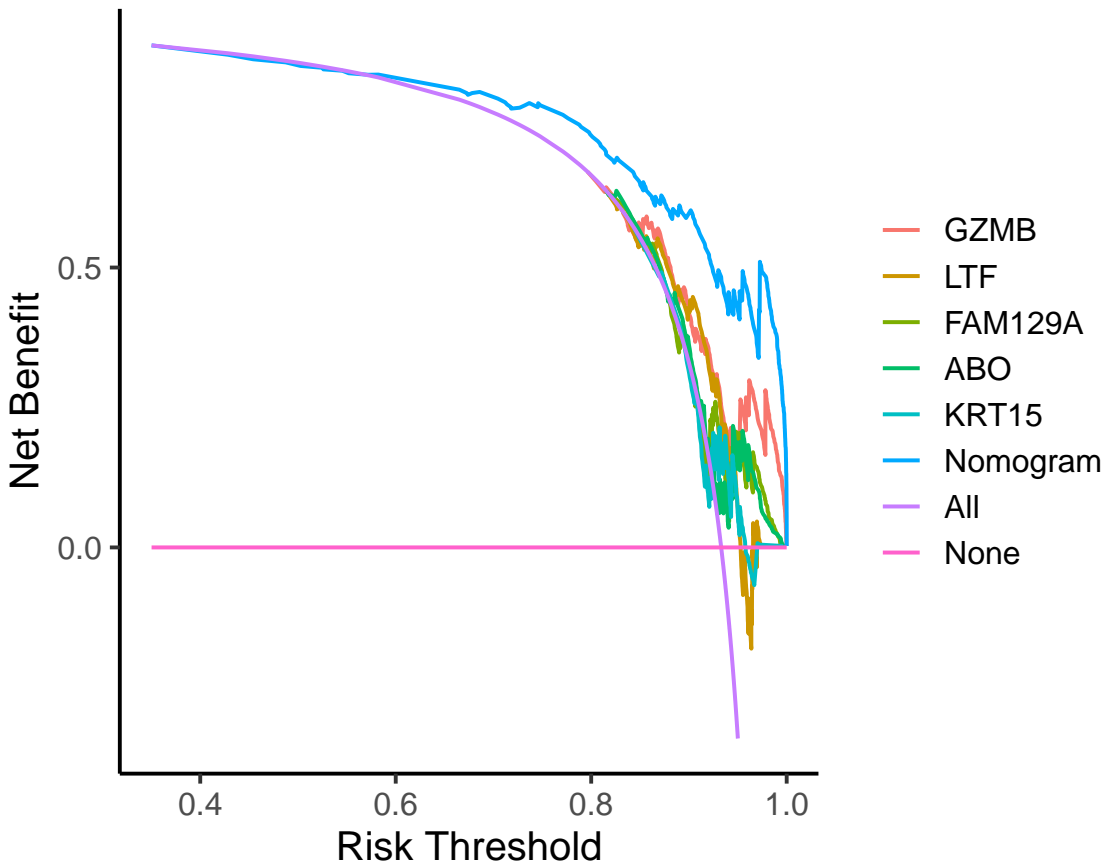

Supplement: Supplementary file 1 [file Supplementaryfile1.zip › Supplementary Material/Nomogram/CD/DCA_CD_Nomogram_Train.pdf]

## CD Nomogram Valid

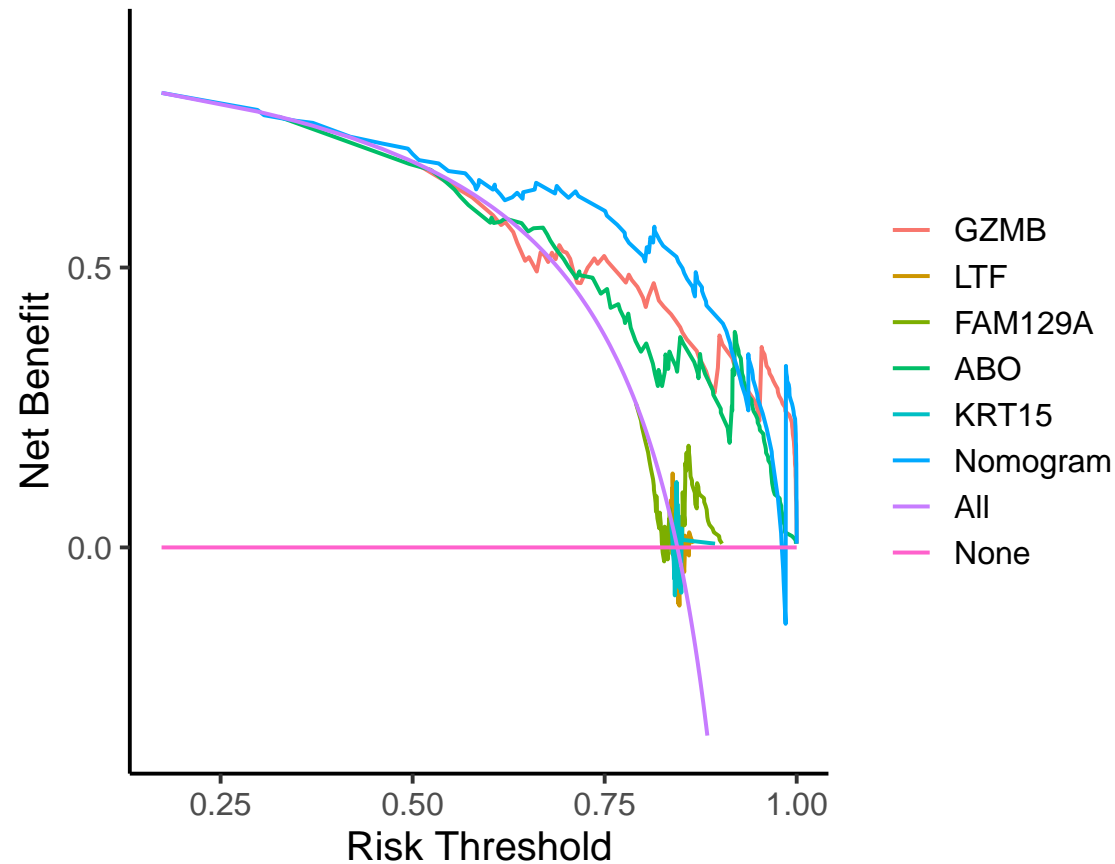

Supplement: Supplementary file 1 [file Supplementaryfile1.zip › Supplementary Material/Nomogram/CD/DCA_CD_Nomogram_Valid.pdf]

# CD Nomogram Train

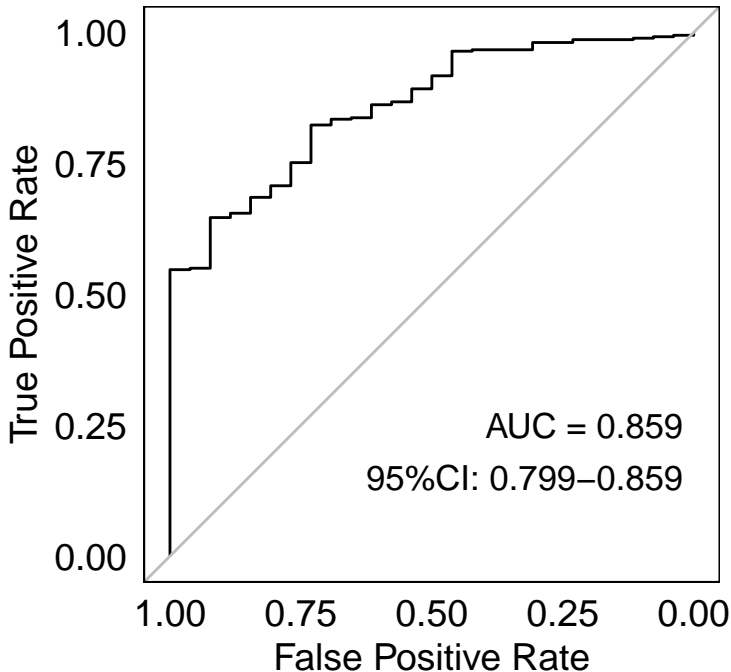

Supplement: Supplementary file 1 [file Supplementaryfile1.zip › Supplementary Material/Nomogram/CD/ROC_CD_Nomogram_Train.pdf]

# CD Nomogram Valid

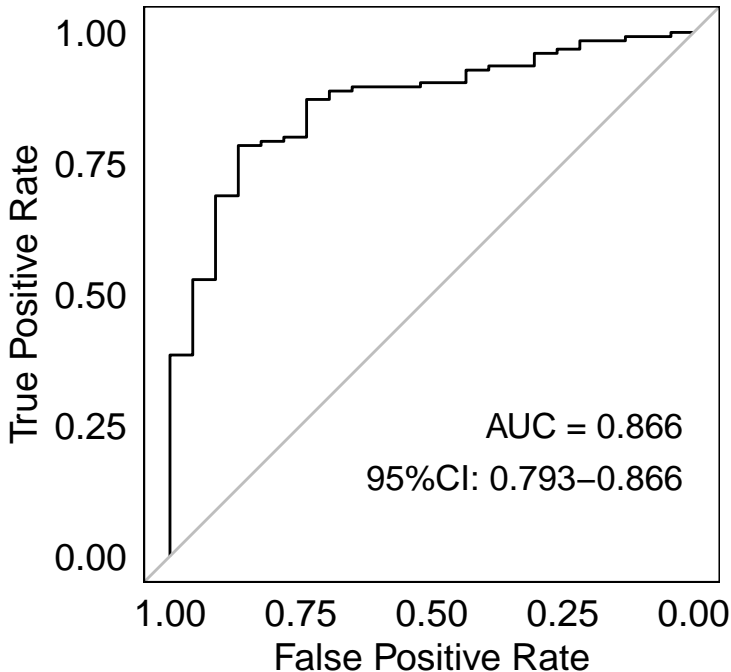

Supplement: Supplementary file 1 [file Supplementaryfile1.zip › Supplementary Material/Nomogram/CD/ROC_CD_Nomogram_Valid.pdf]

## OP Nomogram Train

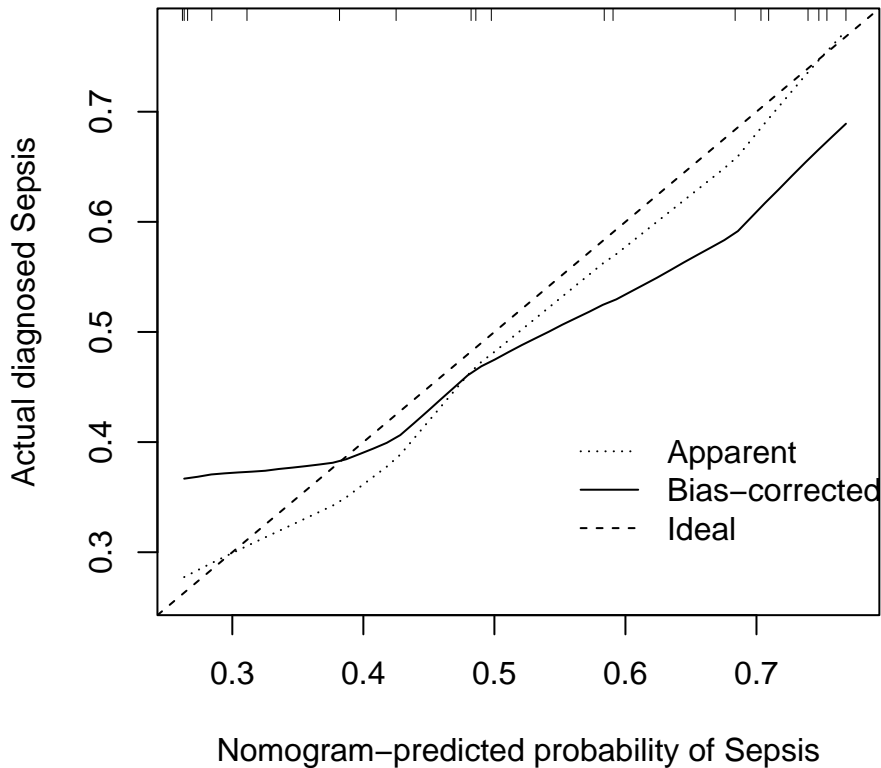

Supplement: Supplementary file 1 [file Supplementaryfile1.zip › Supplementary Material/Nomogram/OP/Calibration_OP_Nomogram_Train.pdf]

## OP Nomogram Valid

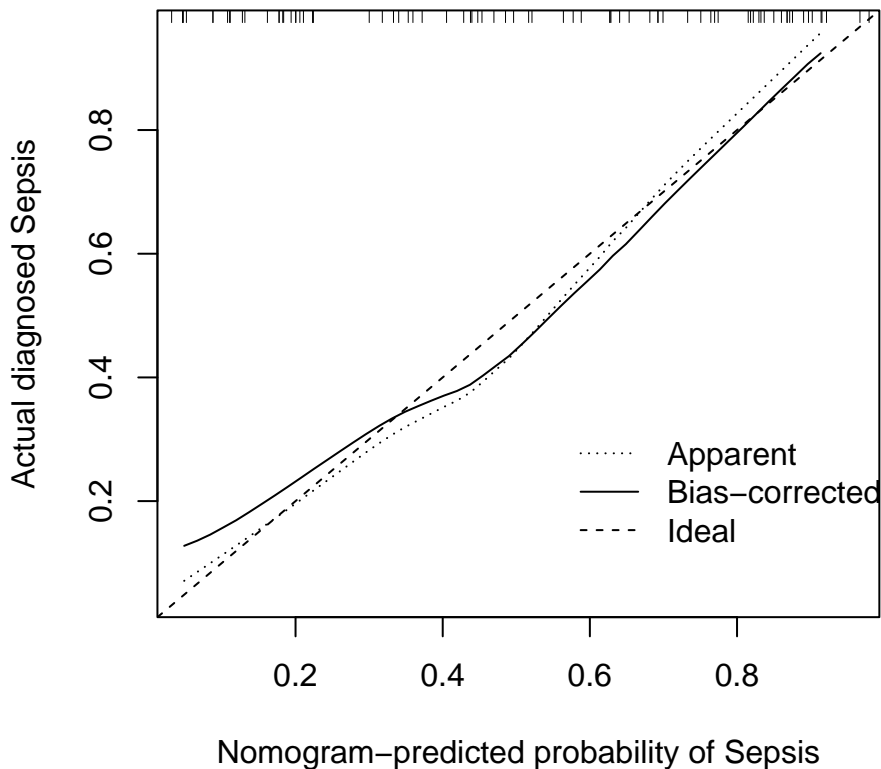

Supplement: Supplementary file 1 [file Supplementaryfile1.zip › Supplementary Material/Nomogram/OP/Calibration_OP_Nomogram_Valid.pdf]

# OP Nomogram Train

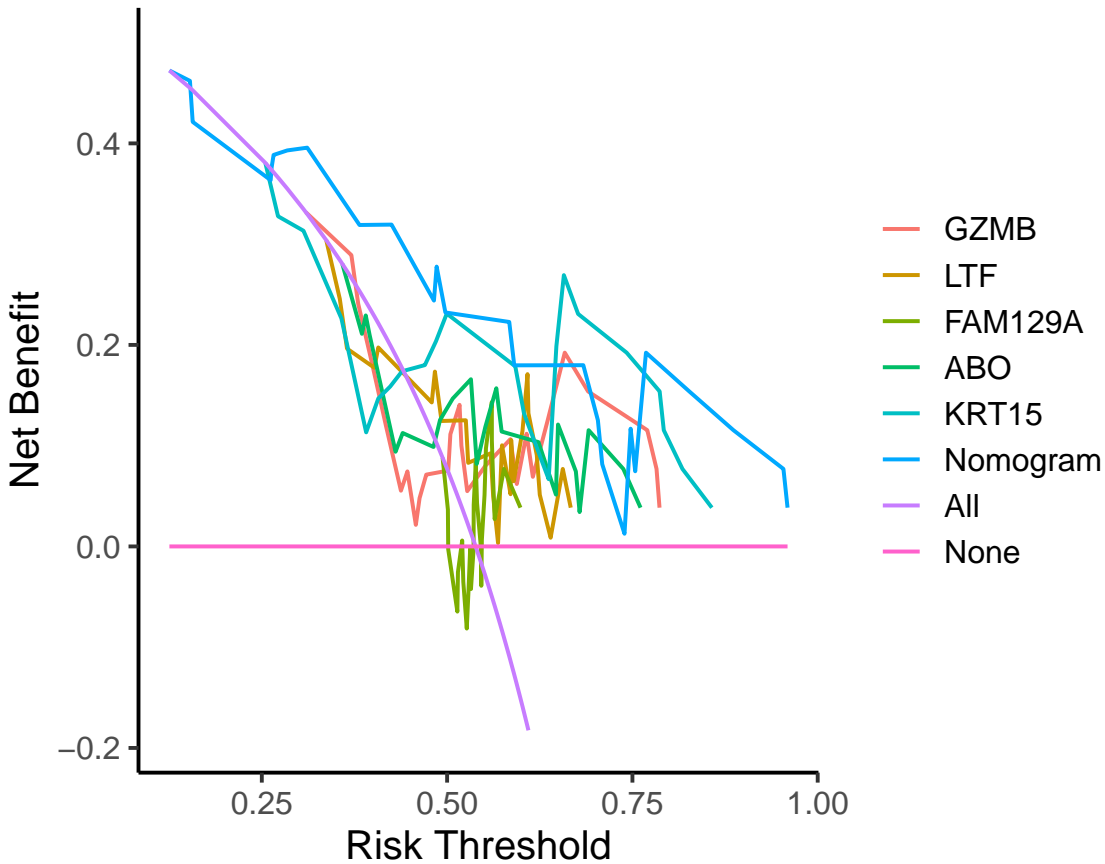

Supplement: Supplementary file 1 [file Supplementaryfile1.zip › Supplementary Material/Nomogram/OP/DCA_OP_Nomogram_Train.pdf]

## OP Nomogram Valid

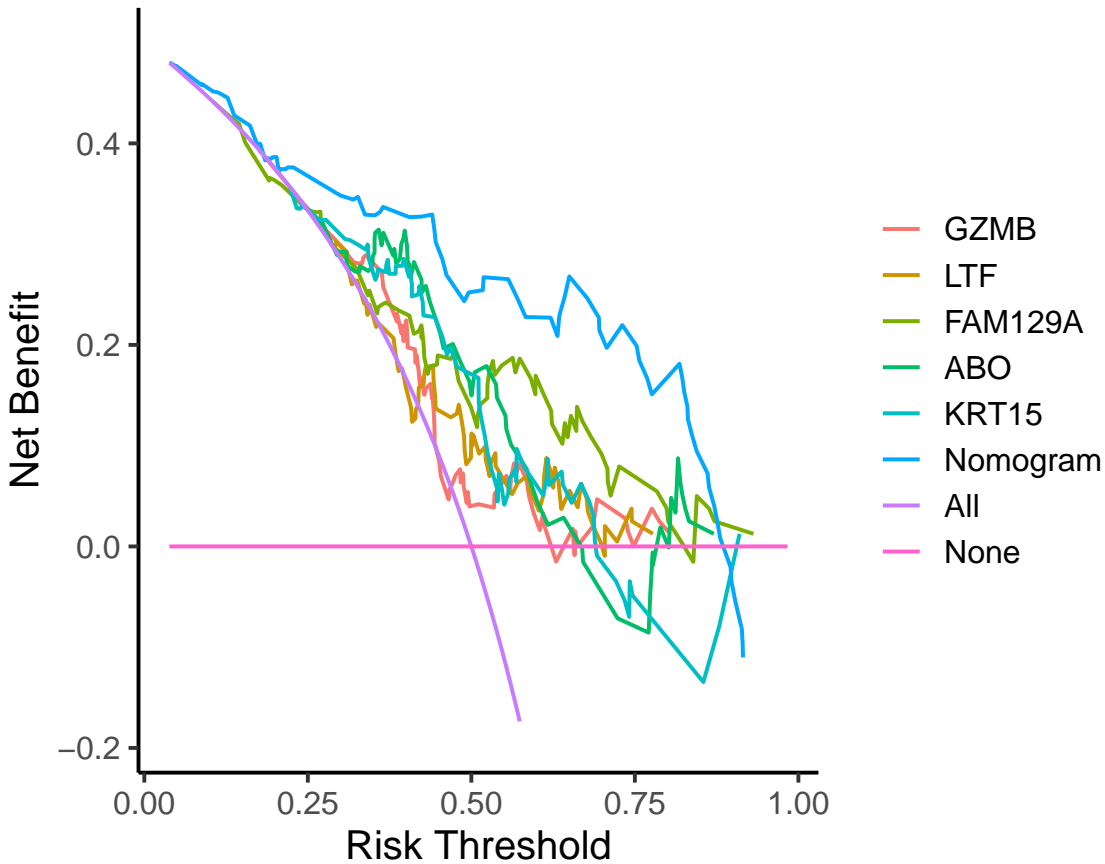

Supplement: Supplementary file 1 [file Supplementaryfile1.zip › Supplementary Material/Nomogram/OP/DCA_OP_Nomogram_Valid.pdf]

# OP Nomogram Train

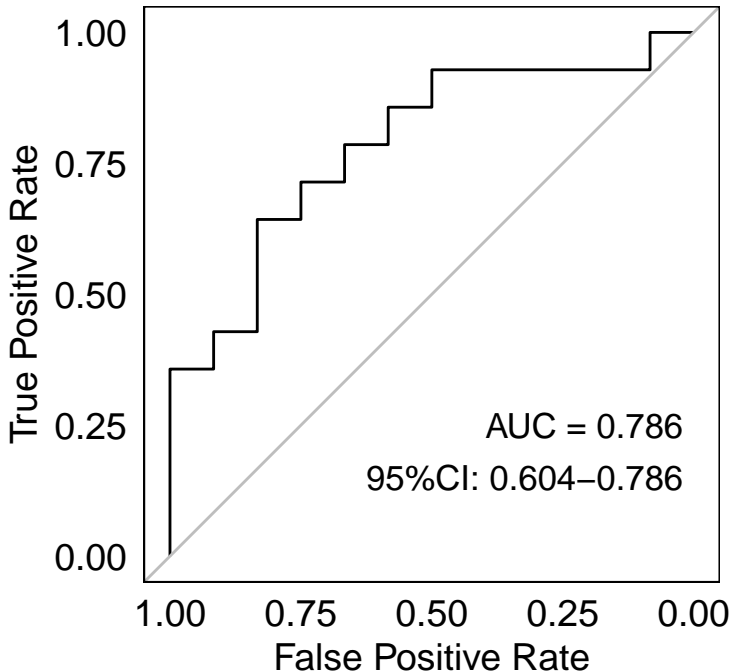

Supplement: Supplementary file 1 [file Supplementaryfile1.zip › Supplementary Material/Nomogram/OP/ROC_OP_Nomogram_Train.pdf]

# Nomogram Valid

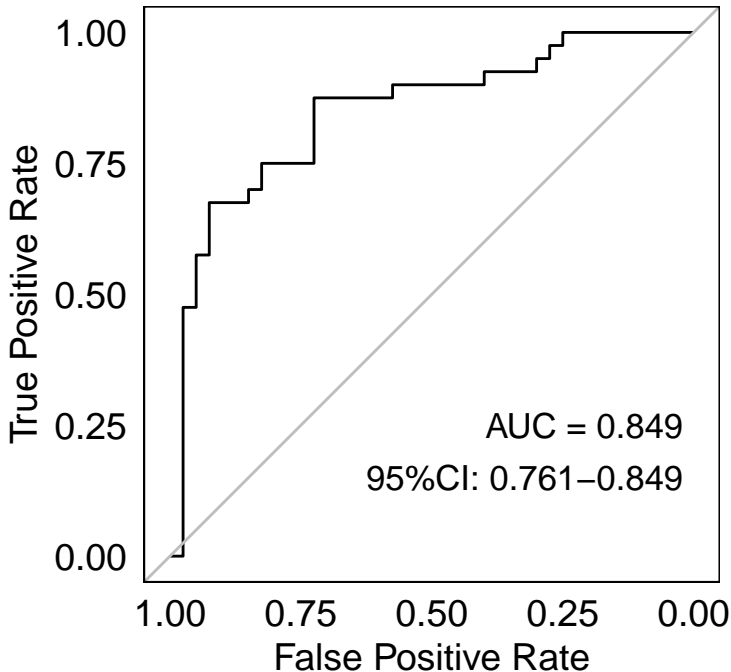

Supplement: Supplementary file 1 [file Supplementaryfile1.zip › Supplementary Material/Nomogram/OP/ROC_OP_Nomogram_Valid.pdf]

**CD**

Mitochondria geneset (log2CPM)

$R = 0.29, p = 3.2e-08$

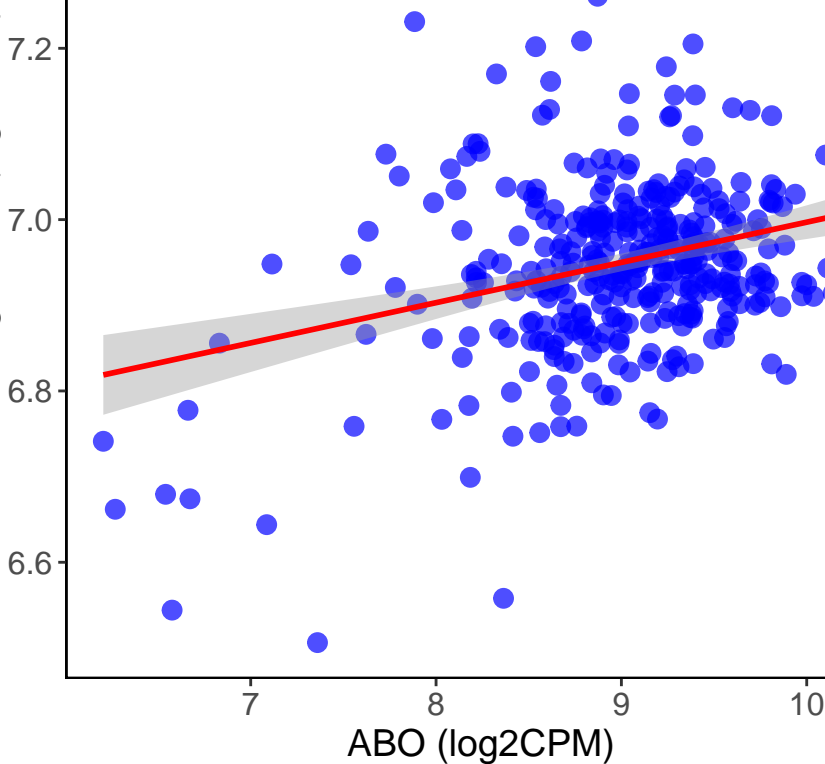

Supplement: Supplementary file 1 [file Supplementaryfile1.zip › Supplementary Material/Pathway/GSE112366/ABO_Mitochondria_Correlation_CD.pdf]

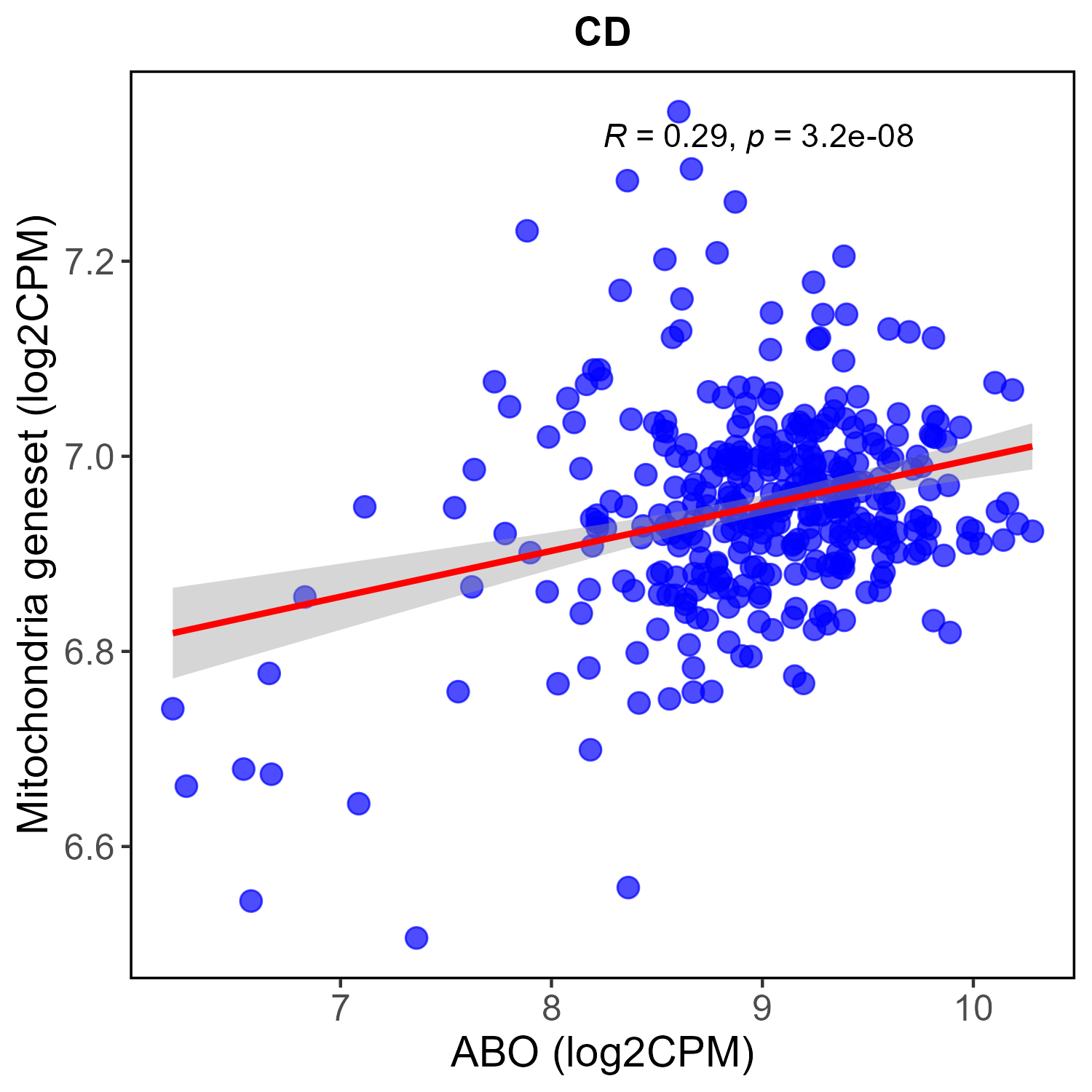

Supplement: Supplementary file 1 [file Supplementaryfile1.zip › Supplementary Material/Pathway/GSE112366/ABO_Mitochondria_Correlation_CD.png]

**CD**

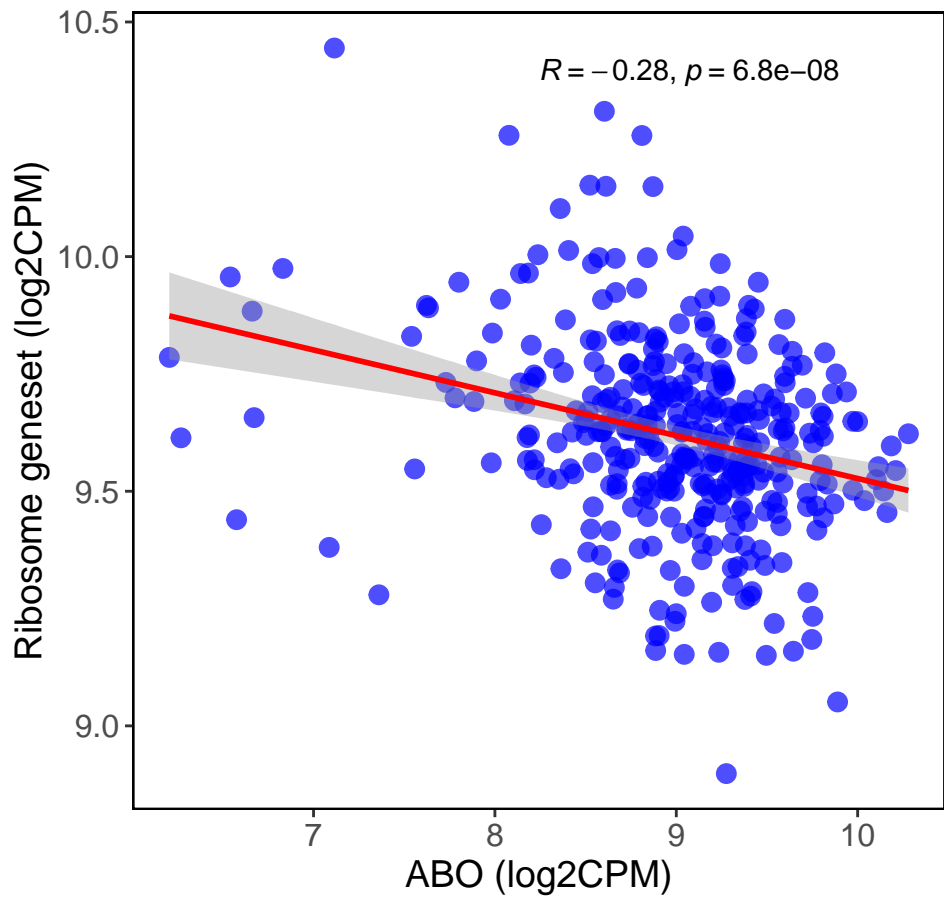

Supplement: Supplementary file 1 [file Supplementaryfile1.zip › Supplementary Material/Pathway/GSE112366/ABO_Ribosome_Correlation_CD.pdf]

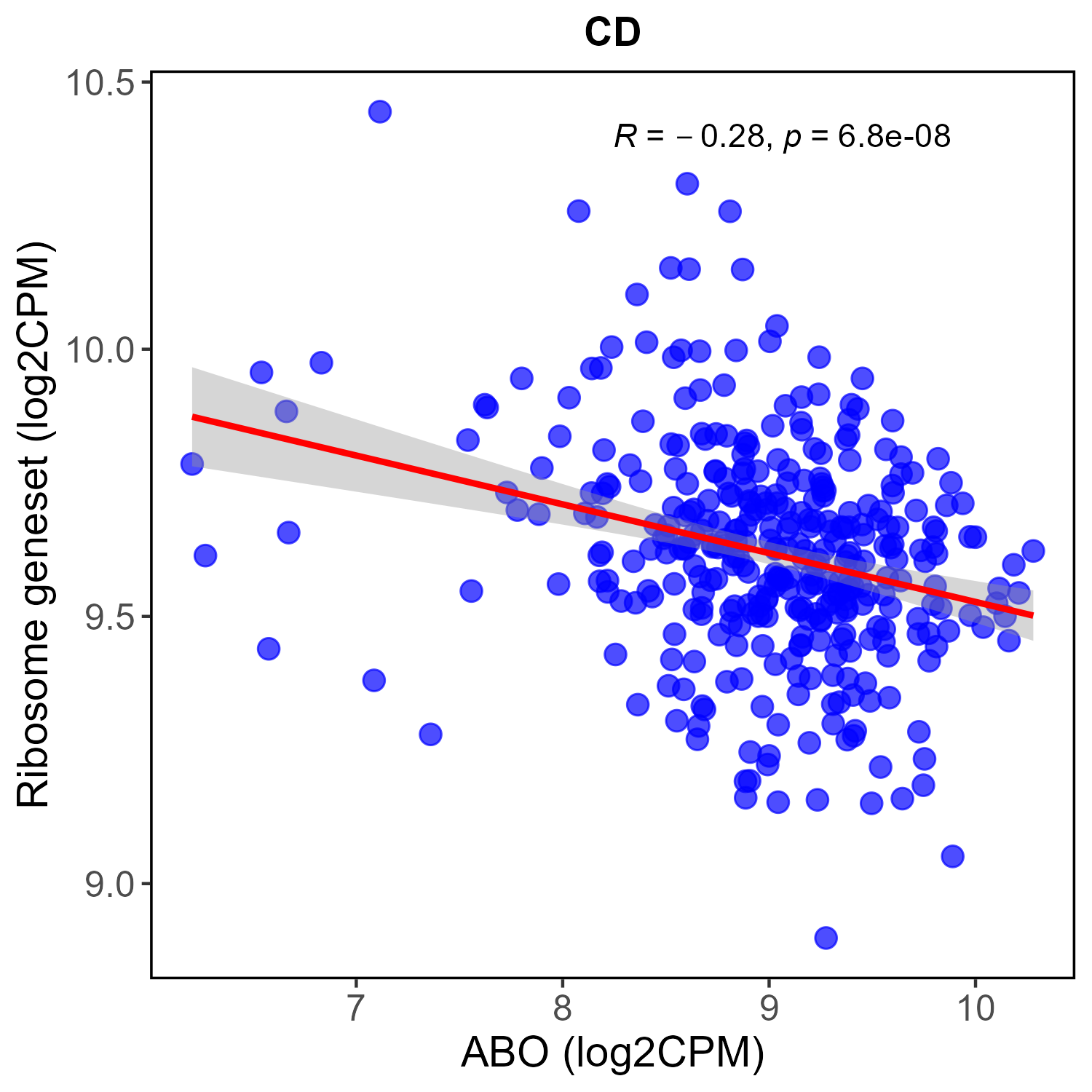

Supplement: Supplementary file 1 [file Supplementaryfile1.zip › Supplementary Material/Pathway/GSE112366/ABO_Ribosome_Correlation_CD.png]

OP

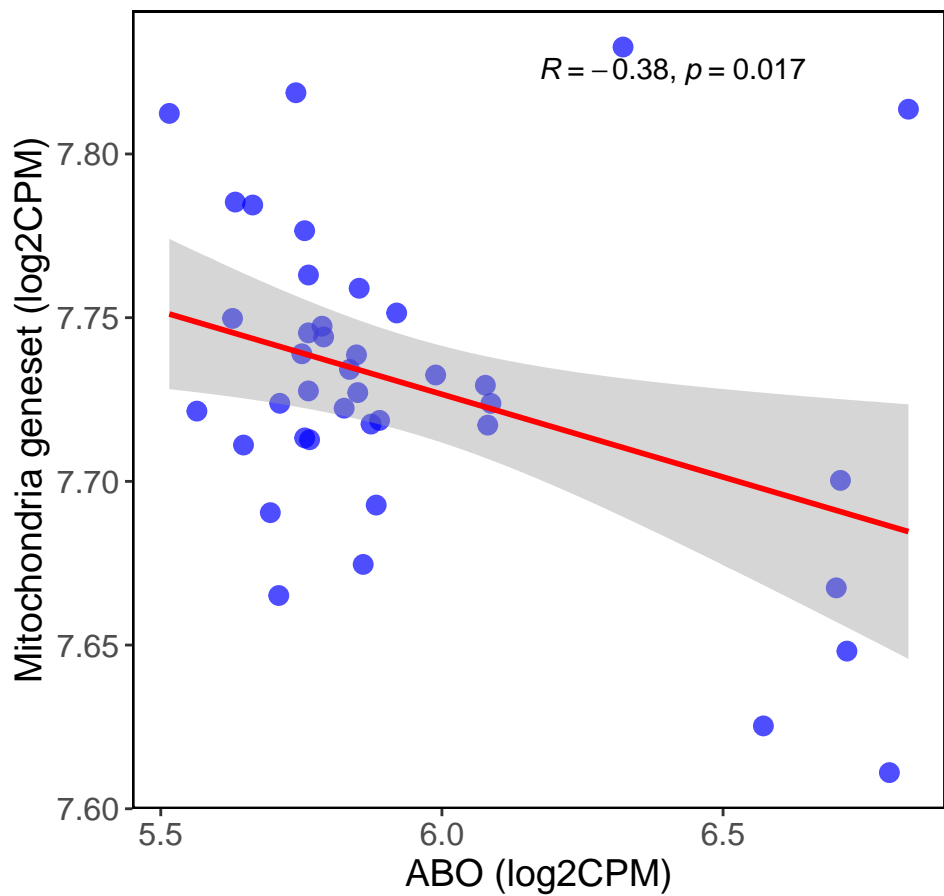

Supplement: Supplementary file 1 [file Supplementaryfile1.zip › Supplementary Material/Pathway/GSE56815/ABO_Mitochondria_Correlation_Low_BMD.pdf]

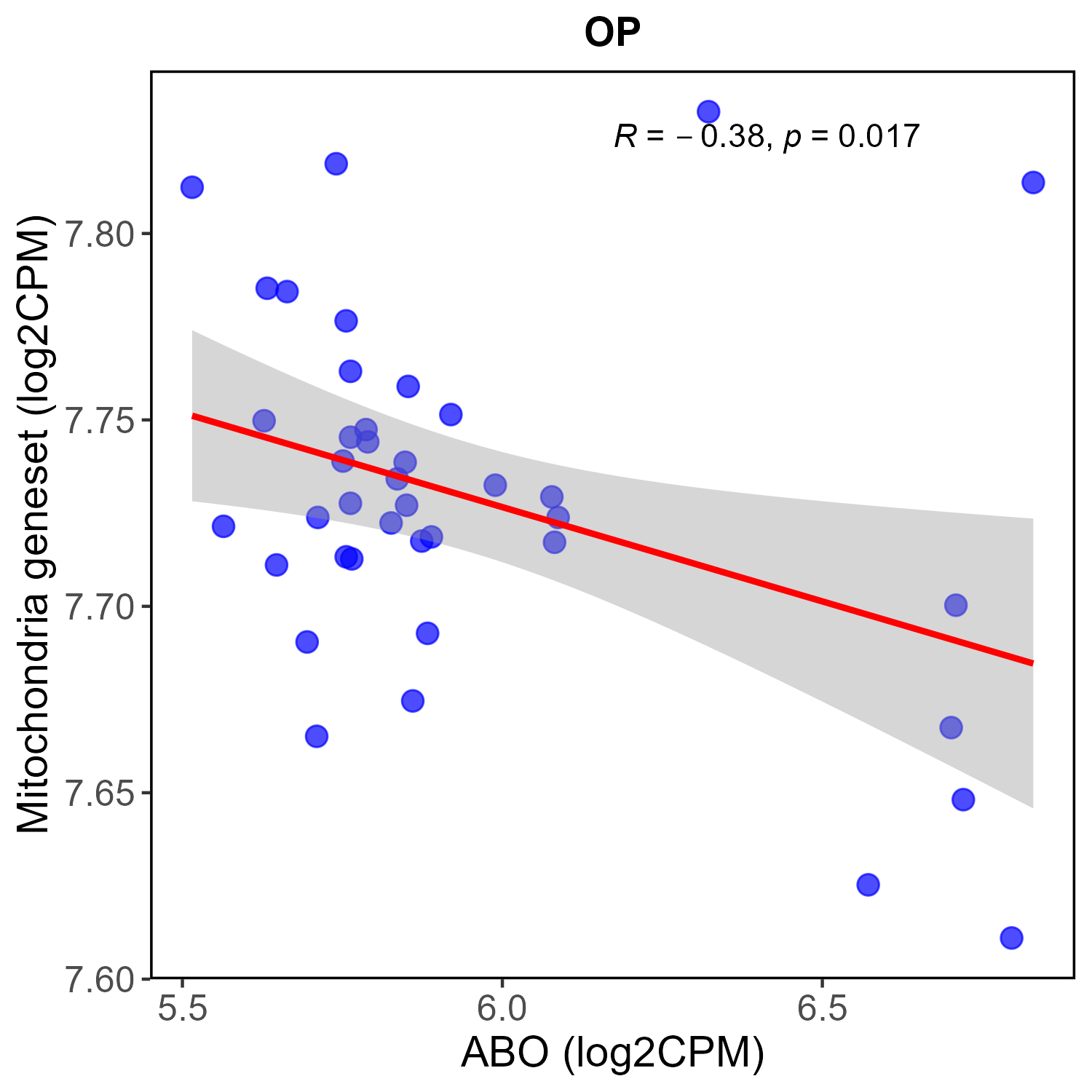

Supplement: Supplementary file 1 [file Supplementaryfile1.zip › Supplementary Material/Pathway/GSE56815/ABO_Mitochondria_Correlation_Low_BMD.png]

OP

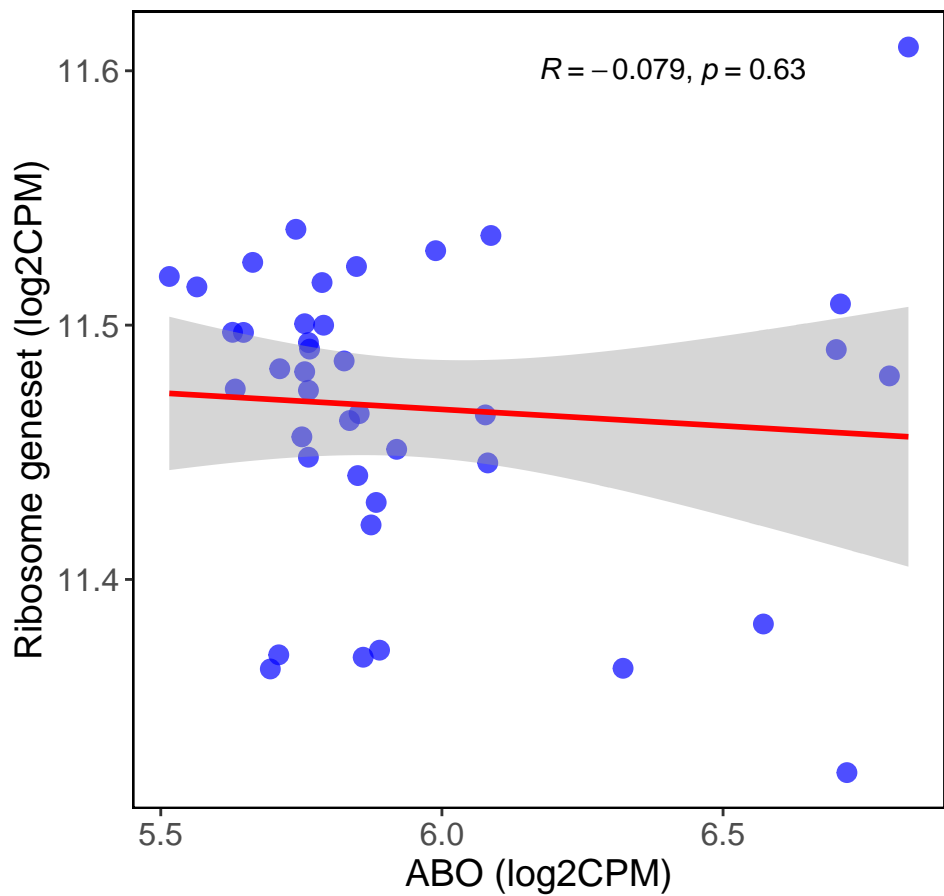

Supplement: Supplementary file 1 [file Supplementaryfile1.zip › Supplementary Material/Pathway/GSE56815/ABO_Ribosome_Correlation_Low_BMD.pdf]

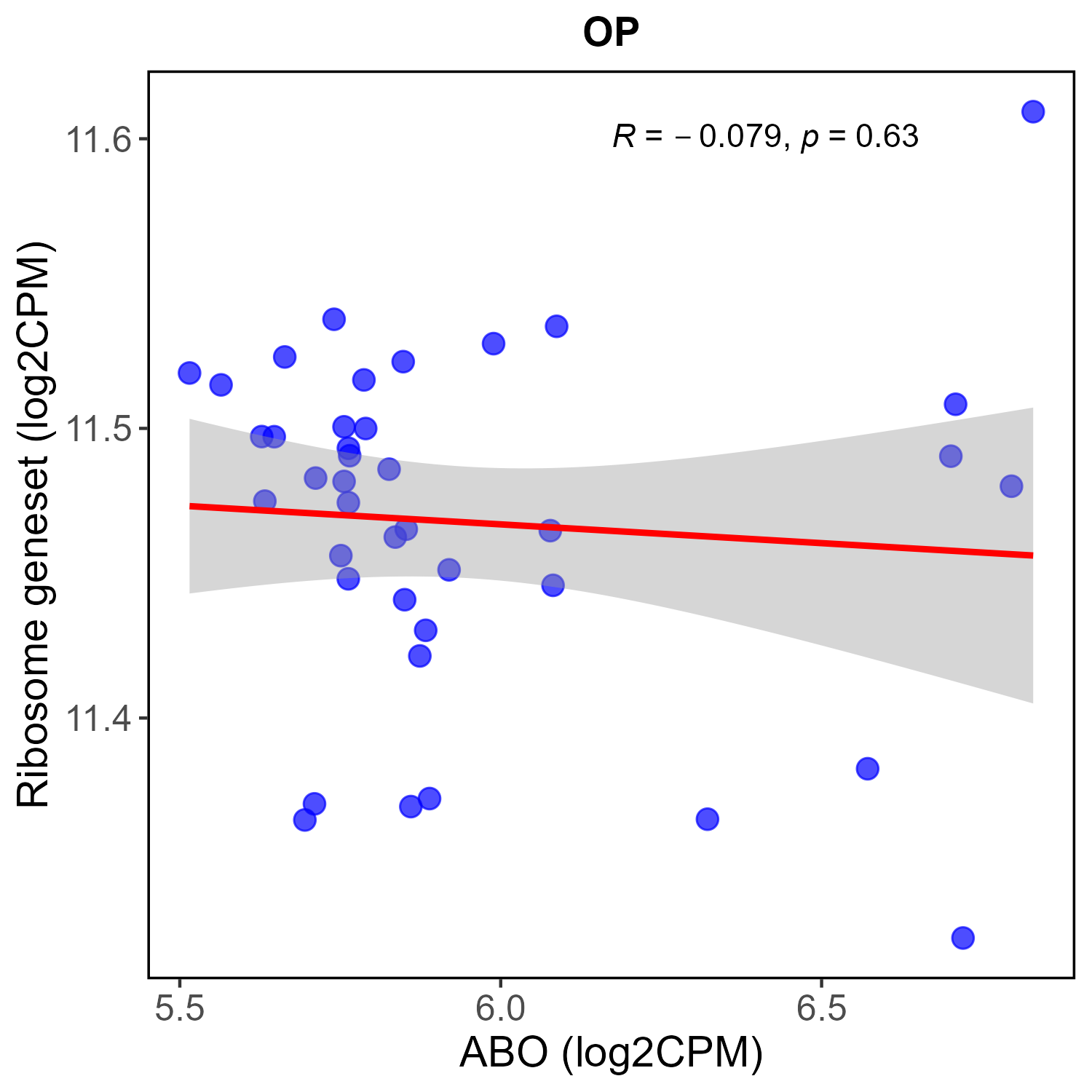

Supplement: Supplementary file 1 [file Supplementaryfile1.zip › Supplementary Material/Pathway/GSE56815/ABO_Ribosome_Correlation_Low_BMD.png]

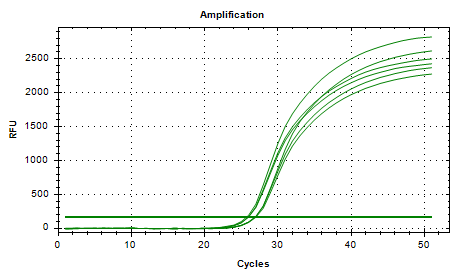

Supplement: Supplementary file 1 [file Supplementaryfile1.zip › Supplementary Material/RT-qPCR/RT-qPCR1/ABO Amplification.png]

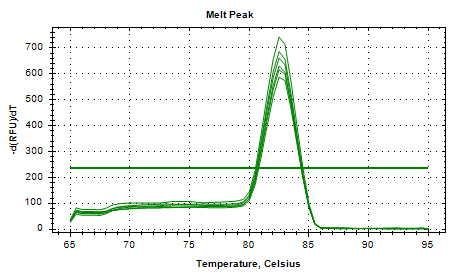

Supplement: Supplementary file 1 [file Supplementaryfile1.zip › Supplementary Material/RT-qPCR/RT-qPCR1/ABO Melt Peak.png]

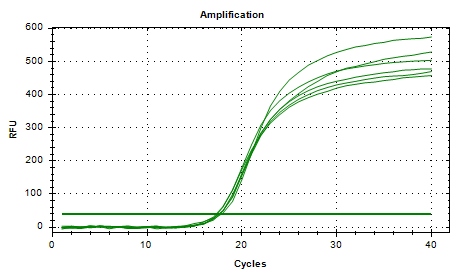

Supplement: Supplementary file 1 [file Supplementaryfile1.zip › Supplementary Material/RT-qPCR/RT-qPCR1/GAPDH Amplification.png]

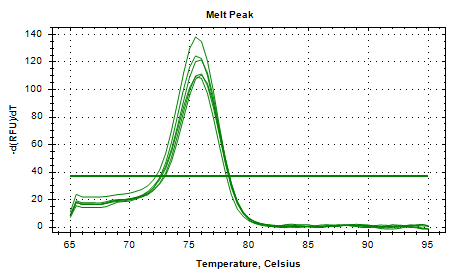

Supplement: Supplementary file 1 [file Supplementaryfile1.zip › Supplementary Material/RT-qPCR/RT-qPCR1/GAPDH Melt Peak.png]

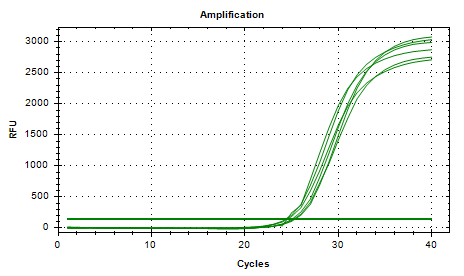

Supplement: Supplementary file 1 [file Supplementaryfile1.zip › Supplementary Material/RT-qPCR/RT-qPCR2/Ctsk Amplification.png]

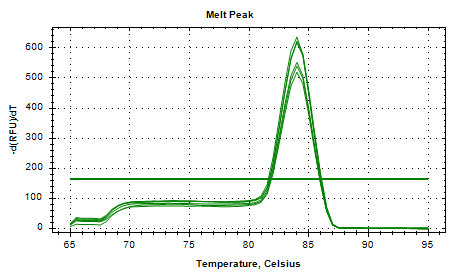

Supplement: Supplementary file 1 [file Supplementaryfile1.zip › Supplementary Material/RT-qPCR/RT-qPCR2/Ctsk Melt Peak.png]

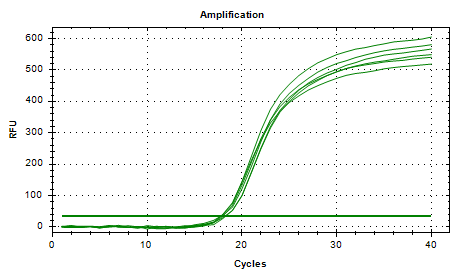

Supplement: Supplementary file 1 [file Supplementaryfile1.zip › Supplementary Material/RT-qPCR/RT-qPCR2/GAPDH Amplification.png]

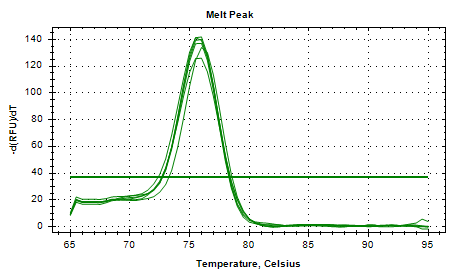

Supplement: Supplementary file 1 [file Supplementaryfile1.zip › Supplementary Material/RT-qPCR/RT-qPCR2/GAPDH Melt Peak.png]

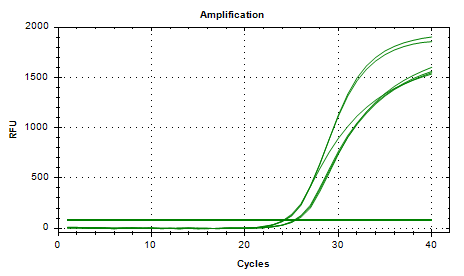

Supplement: Supplementary file 1 [file Supplementaryfile1.zip › Supplementary Material/RT-qPCR/RT-qPCR2/Mmp9 Amplification.png]

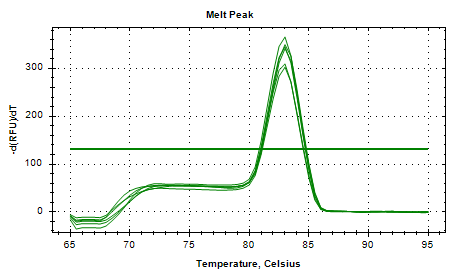

Supplement: Supplementary file 1 [file Supplementaryfile1.zip › Supplementary Material/RT-qPCR/RT-qPCR2/Mmp9 Melt Peak.png]

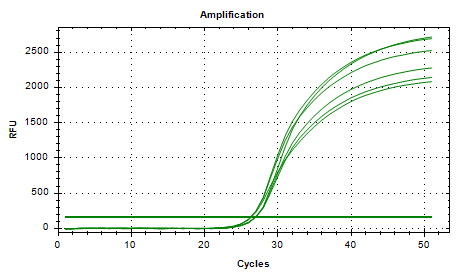

Supplement: Supplementary file 1 [file Supplementaryfile1.zip › Supplementary Material/RT-qPCR/RT-qPCR3/ABO Amplification.png]

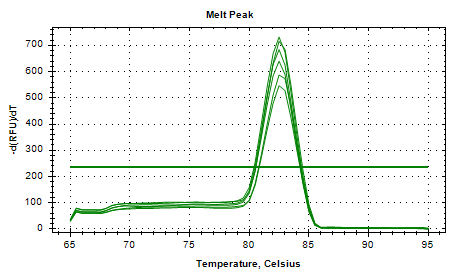

Supplement: Supplementary file 1 [file Supplementaryfile1.zip › Supplementary Material/RT-qPCR/RT-qPCR3/ABO Melt Peak.png]

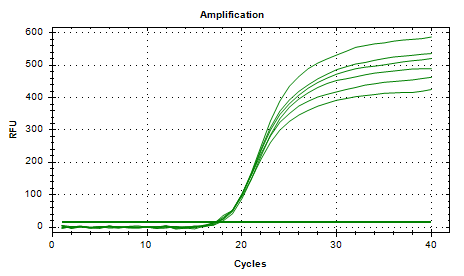

Supplement: Supplementary file 1 [file Supplementaryfile1.zip › Supplementary Material/RT-qPCR/RT-qPCR3/GAPDH Amplification.png]

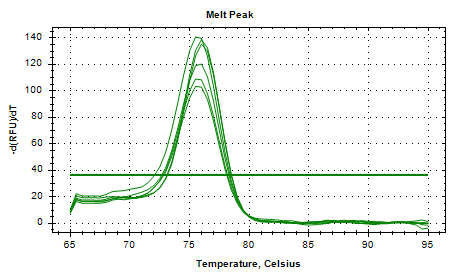

Supplement: Supplementary file 1 [file Supplementaryfile1.zip › Supplementary Material/RT-qPCR/RT-qPCR3/GAPDH Melt Peak.png]

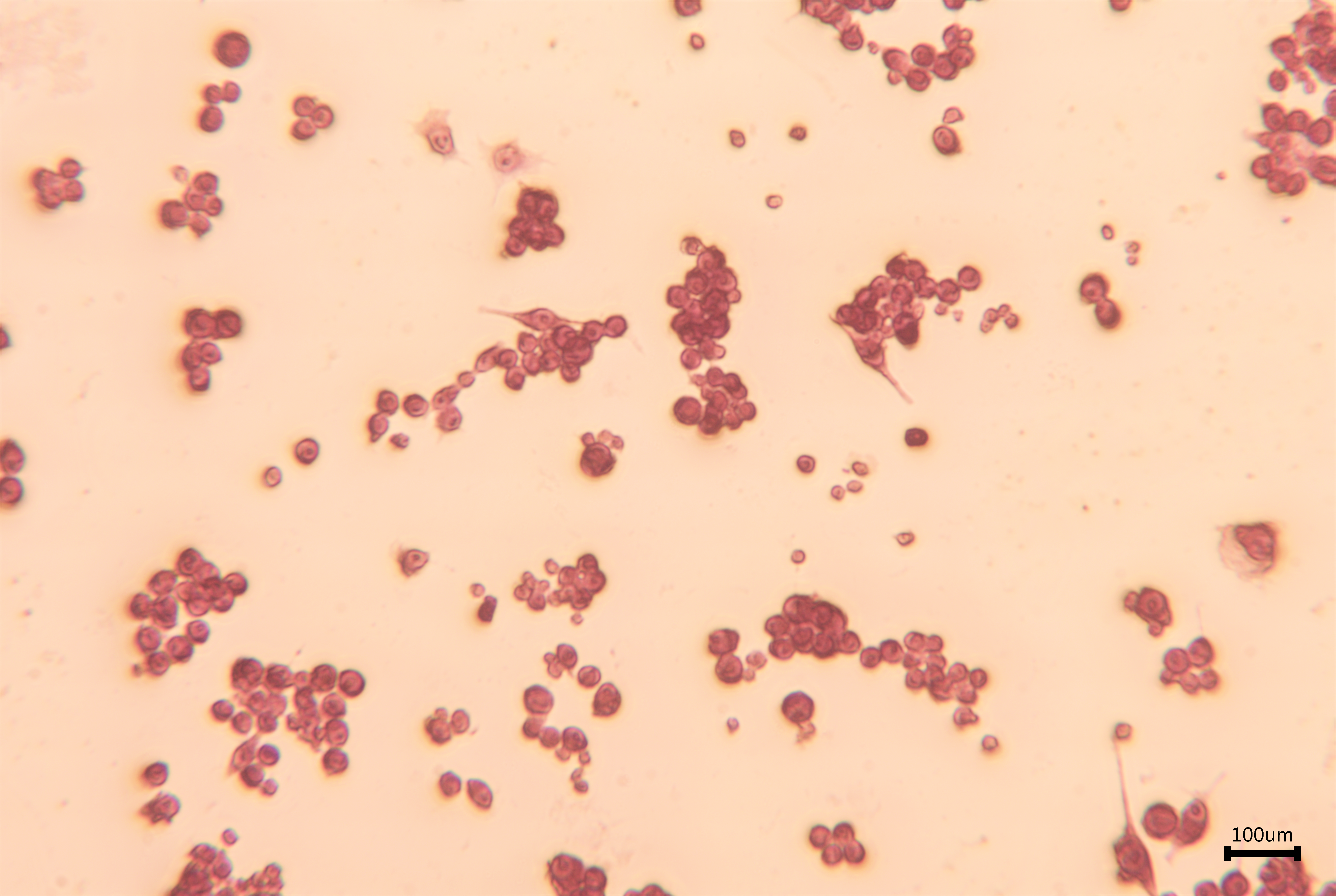

Supplement: Supplementary file 1 [file Supplementaryfile1.zip › Supplementary Material/TRAP staining/A (1)上图.tif]

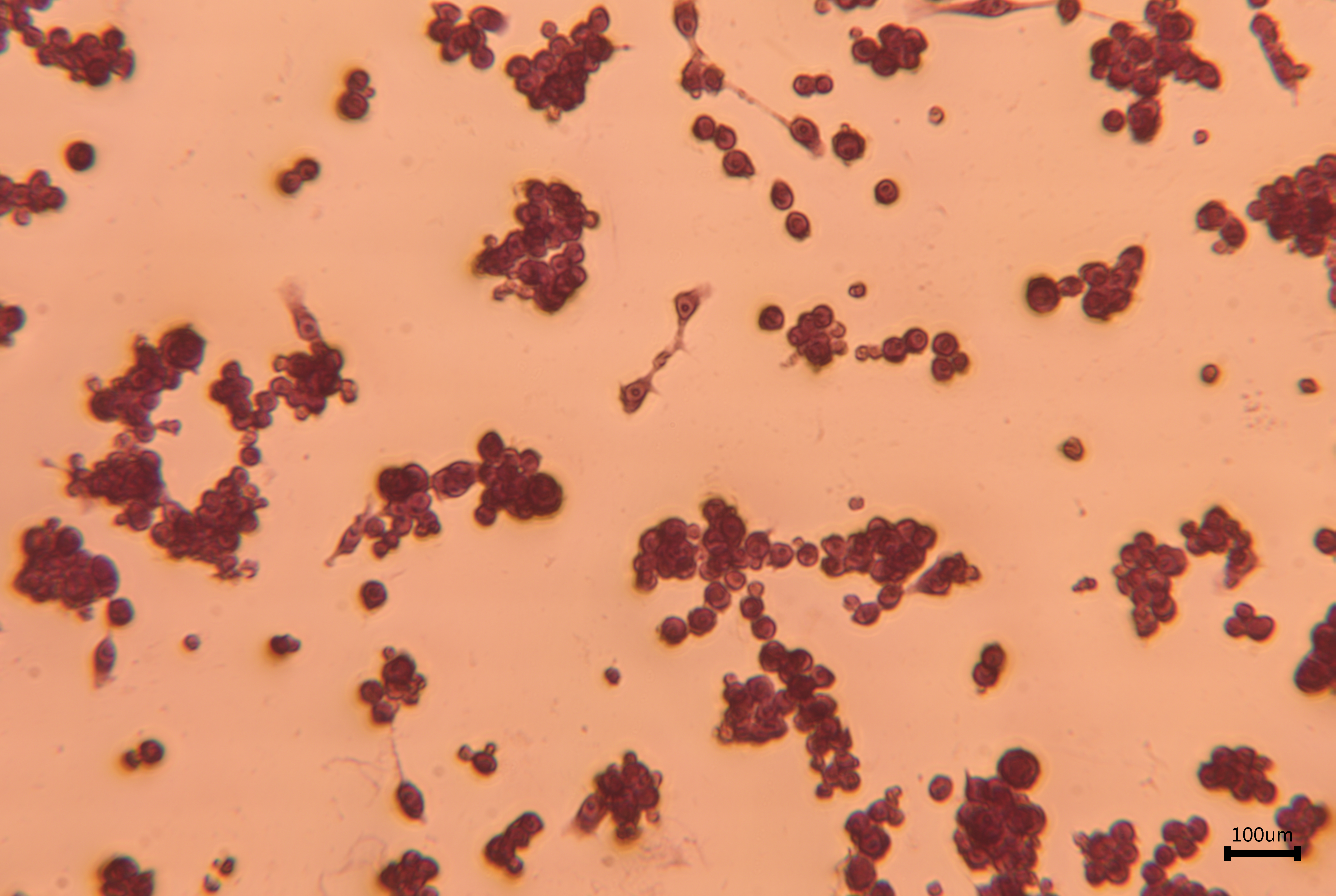

Supplement: Supplementary file 1 [file Supplementaryfile1.zip › Supplementary Material/TRAP staining/A (2).tif]

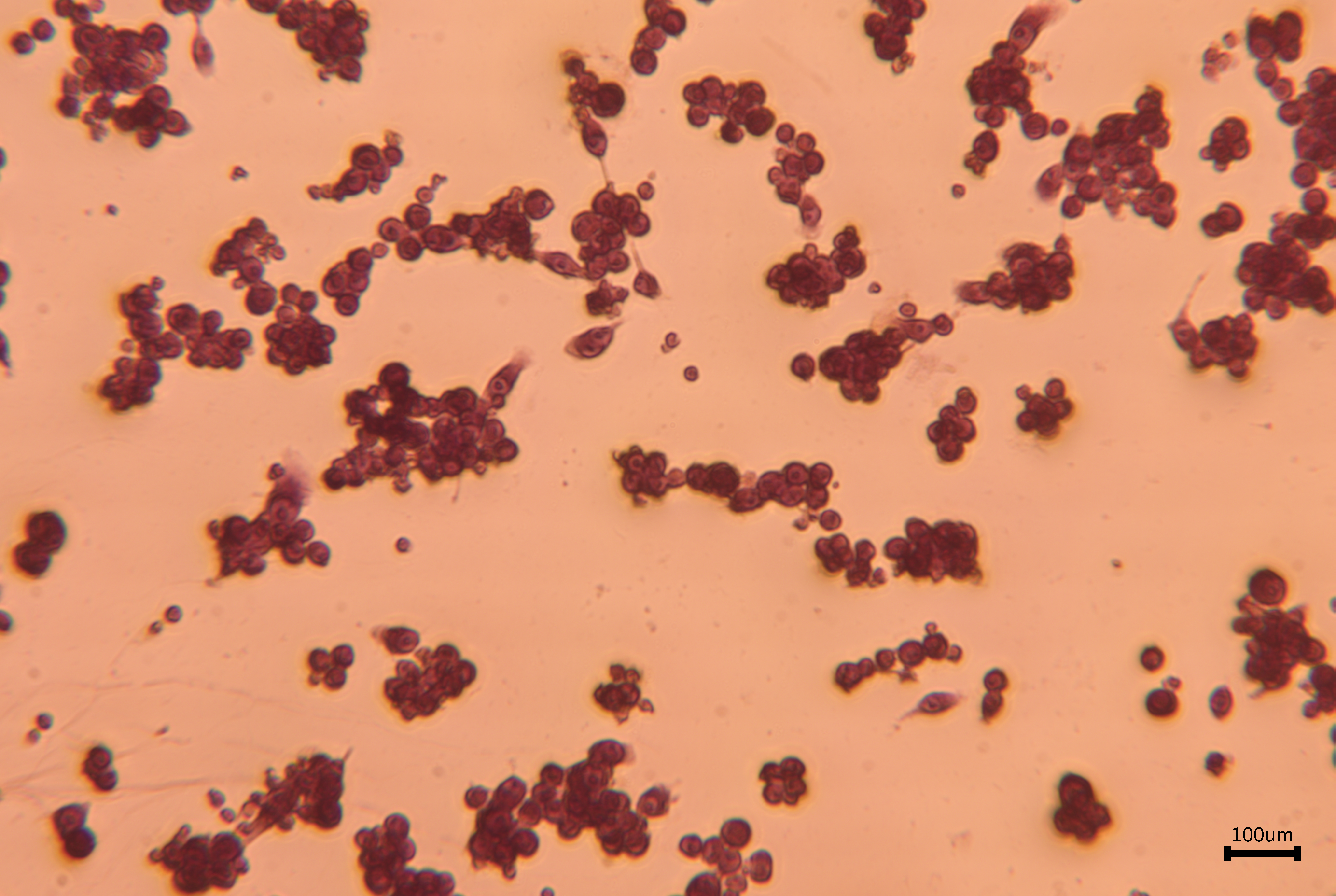

Supplement: Supplementary file 1 [file Supplementaryfile1.zip › Supplementary Material/TRAP staining/A (3).tif]

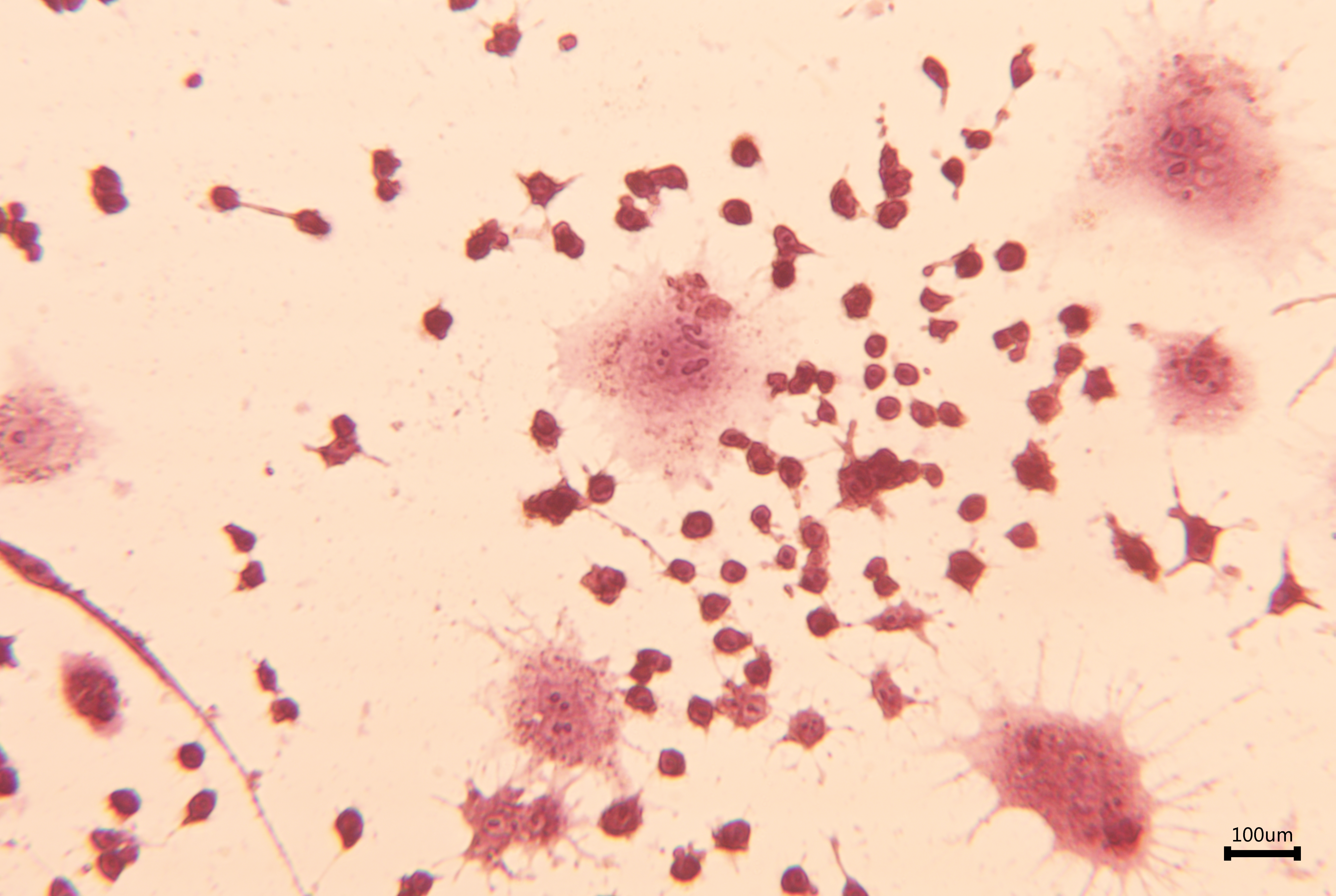

Supplement: Supplementary file 1 [file Supplementaryfile1.zip › Supplementary Material/TRAP staining/B (1)上图.tif]

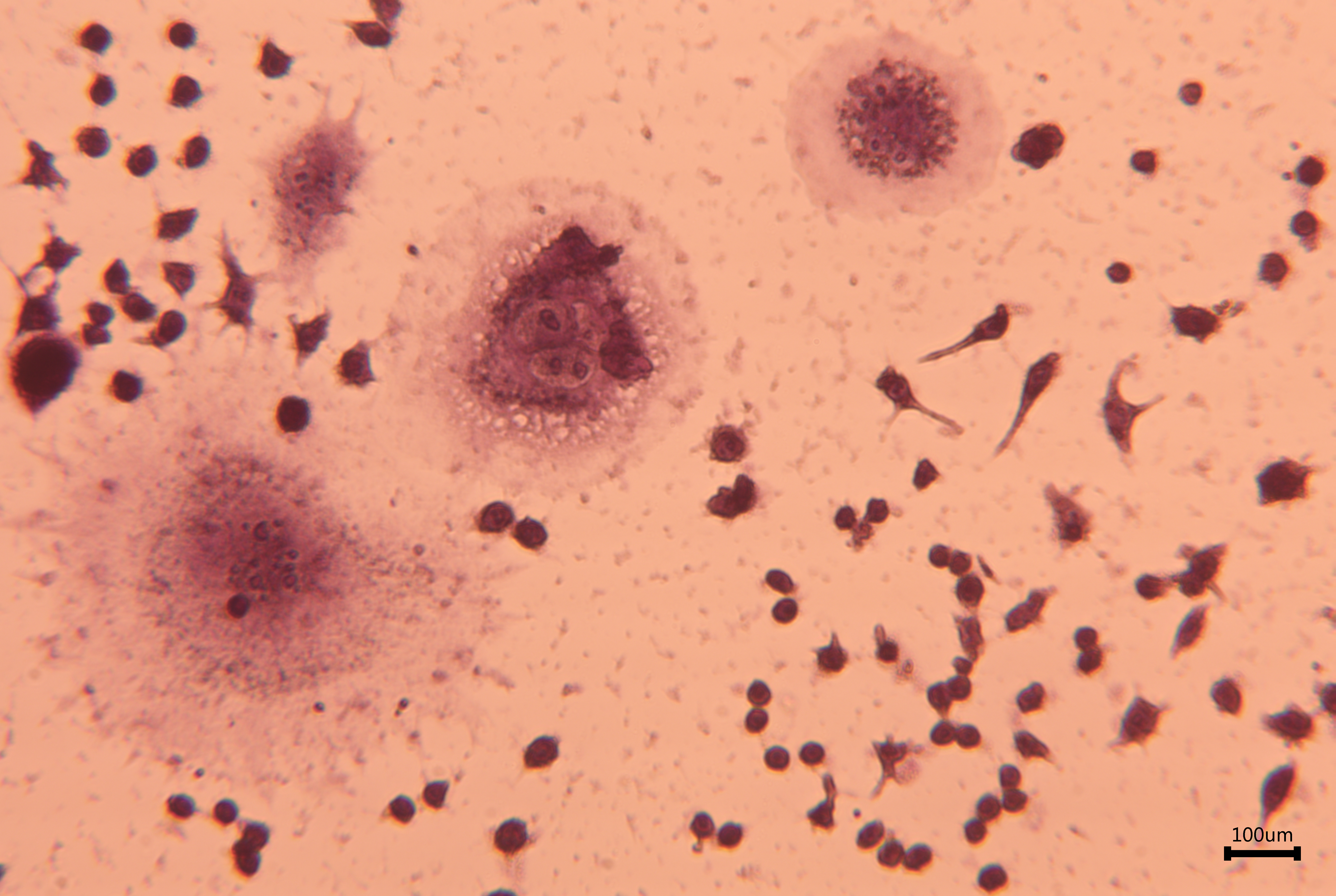

Supplement: Supplementary file 1 [file Supplementaryfile1.zip › Supplementary Material/TRAP staining/B (2).tif]
