# Supplementary figures and images for: Identification of shared diagnostic genes between osteoporosis and Crohn’s disease through integrated transcriptomic analysis and machine learning (part 1 of 2)
Source: Front Genet. 2025 Oct 7;16:1609915. doi: 10.3389/fgene.2025.1609915 (PMC12538133; doi:10.3389/fgene.2025.1609915)

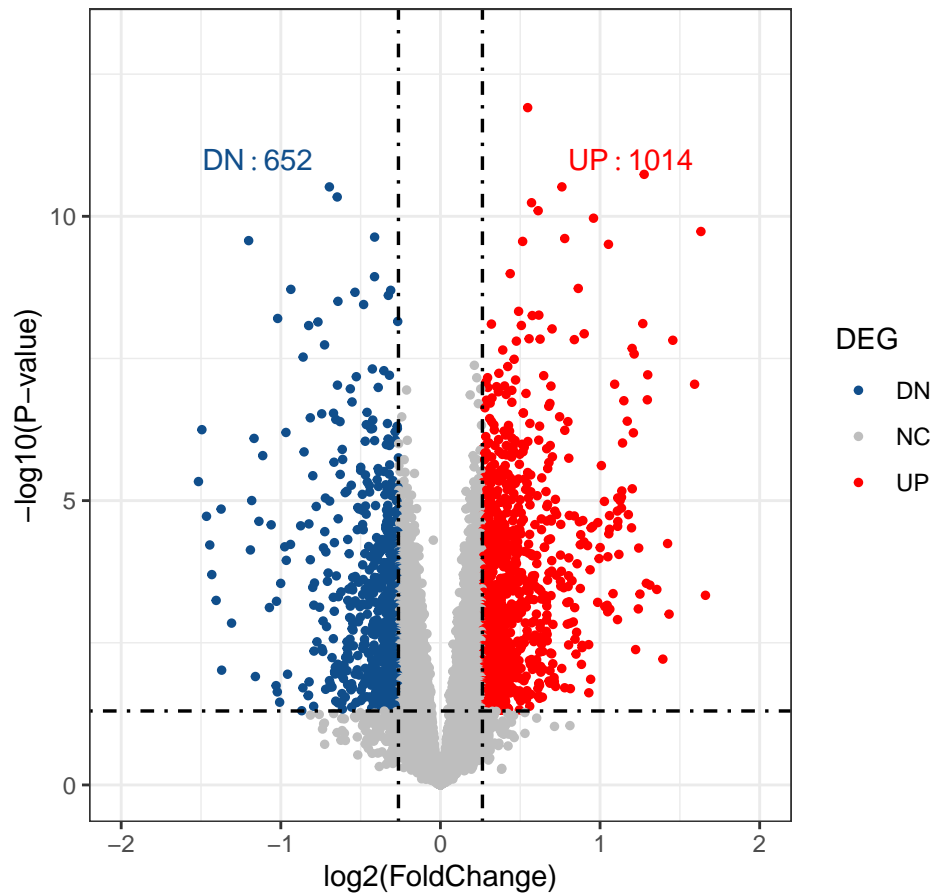

Supplement: Supplementary file 1 [file Supplementaryfile1.zip › Supplementary Material/01_DEG/1.3_Volcano_plot.pdf]

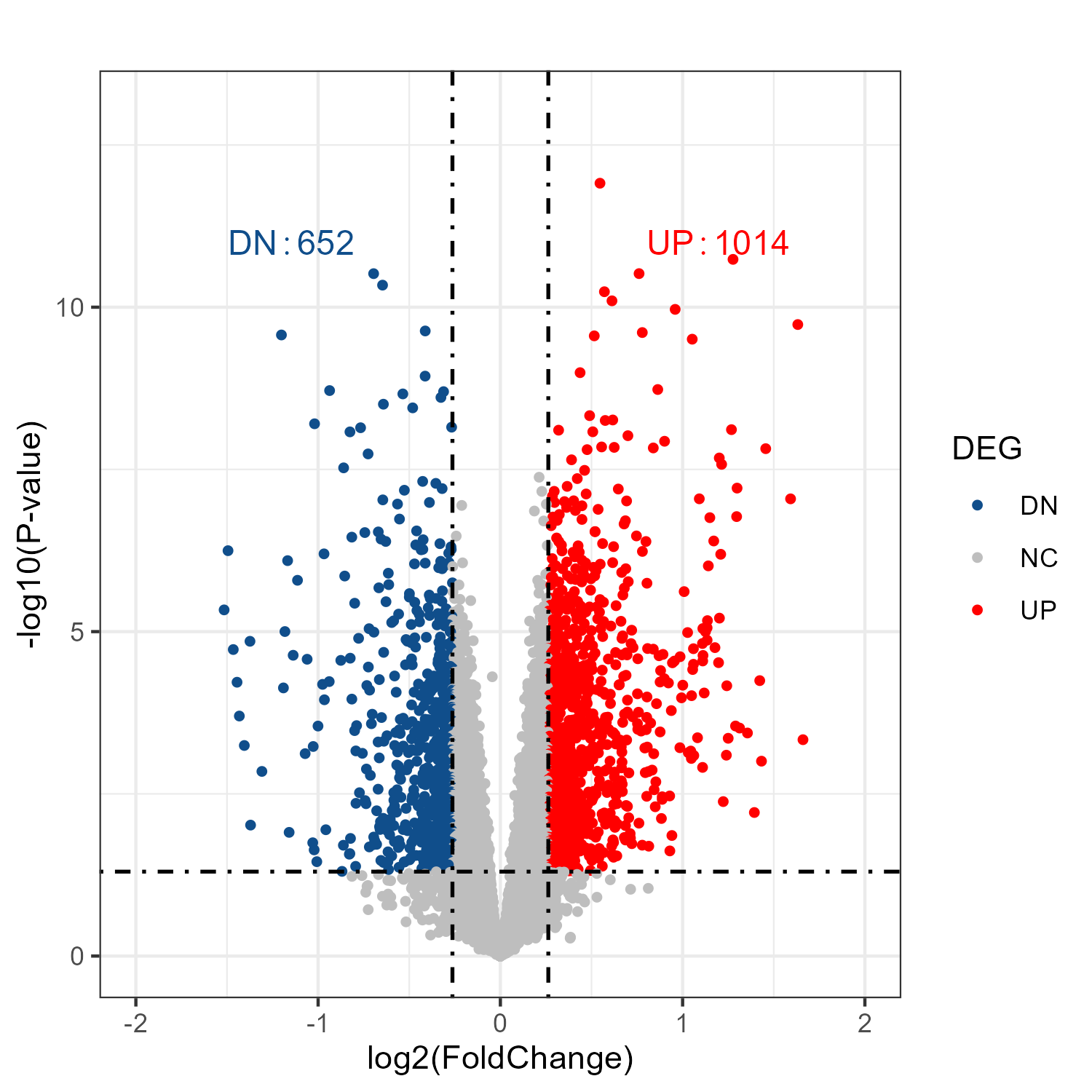

Supplement: Supplementary file 1 [file Supplementaryfile1.zip › Supplementary Material/01_DEG/1.3_Volcano_plot.png]

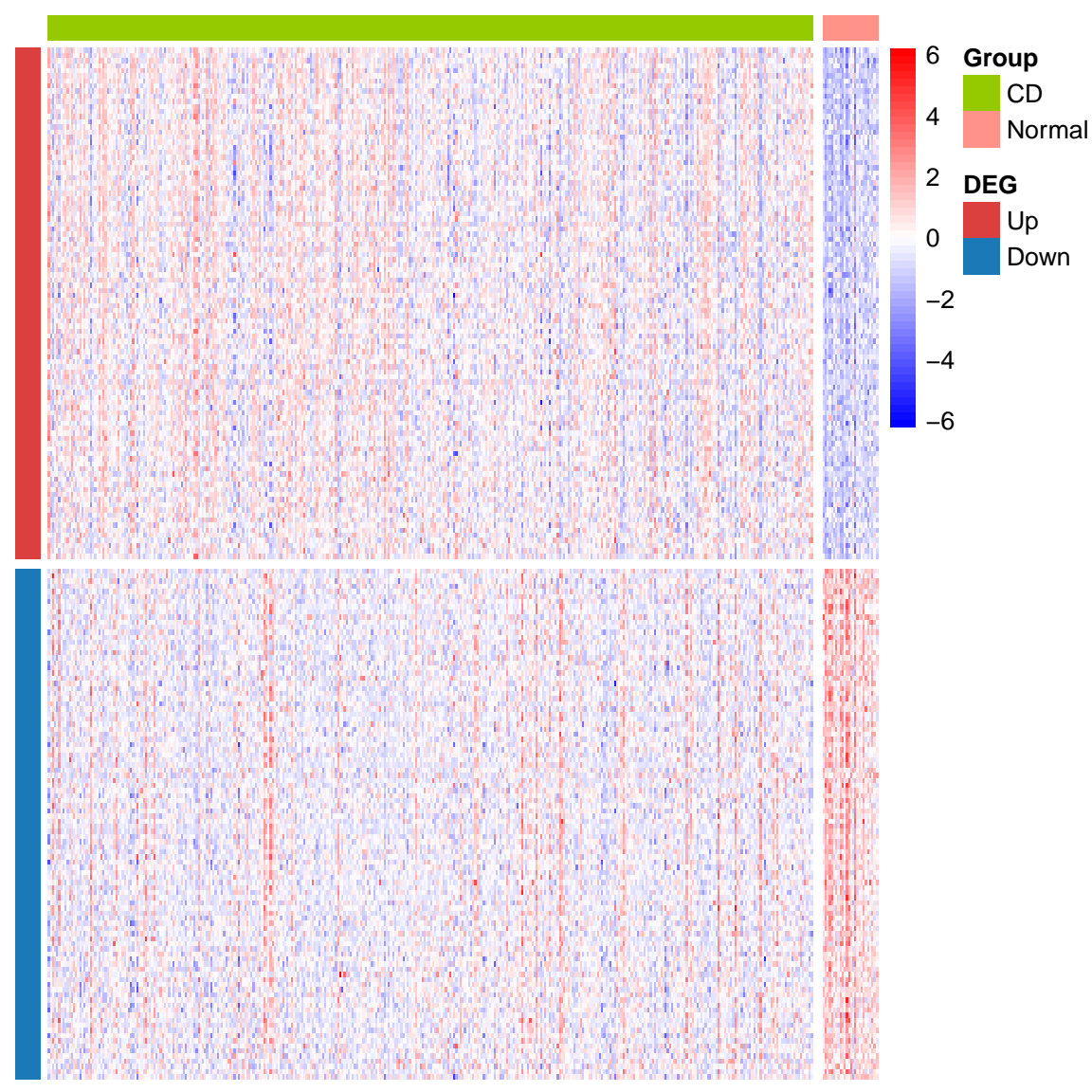

Supplement: Supplementary file 1 [file Supplementaryfile1.zip › Supplementary Material/01_DEG/1.4_Heatmap_DEG.pdf]

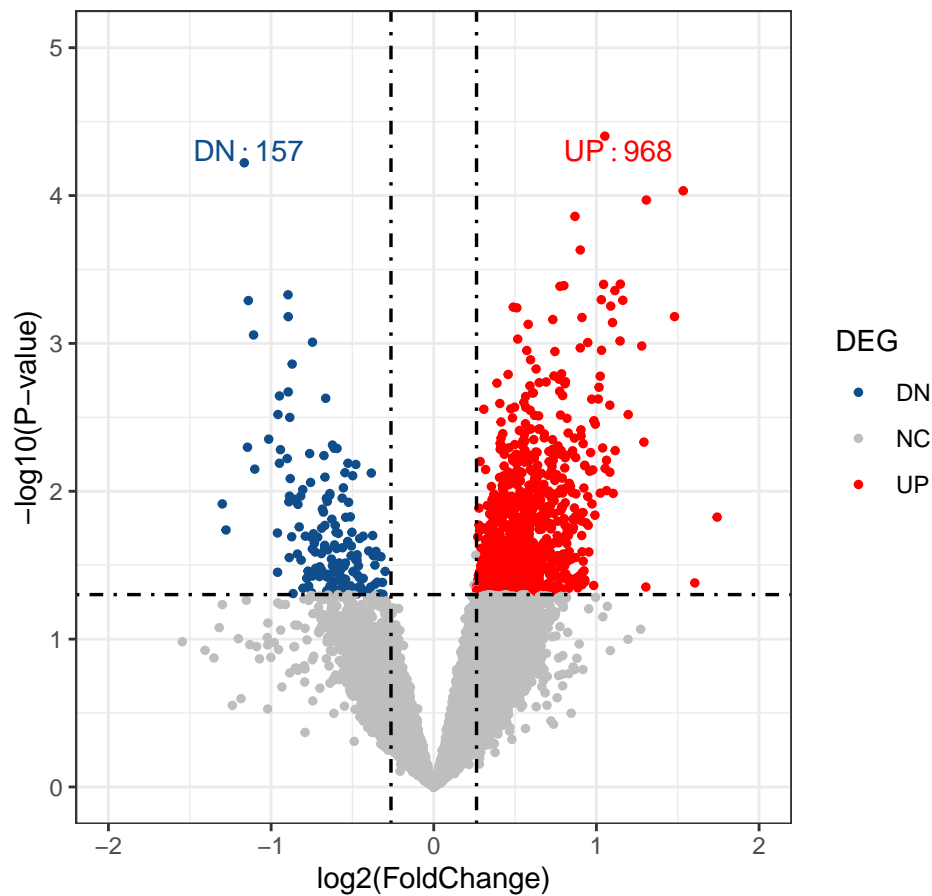

Supplement: Supplementary file 1 [file Supplementaryfile1.zip › Supplementary Material/01_DEG/2.3_Volcano_plot.pdf]

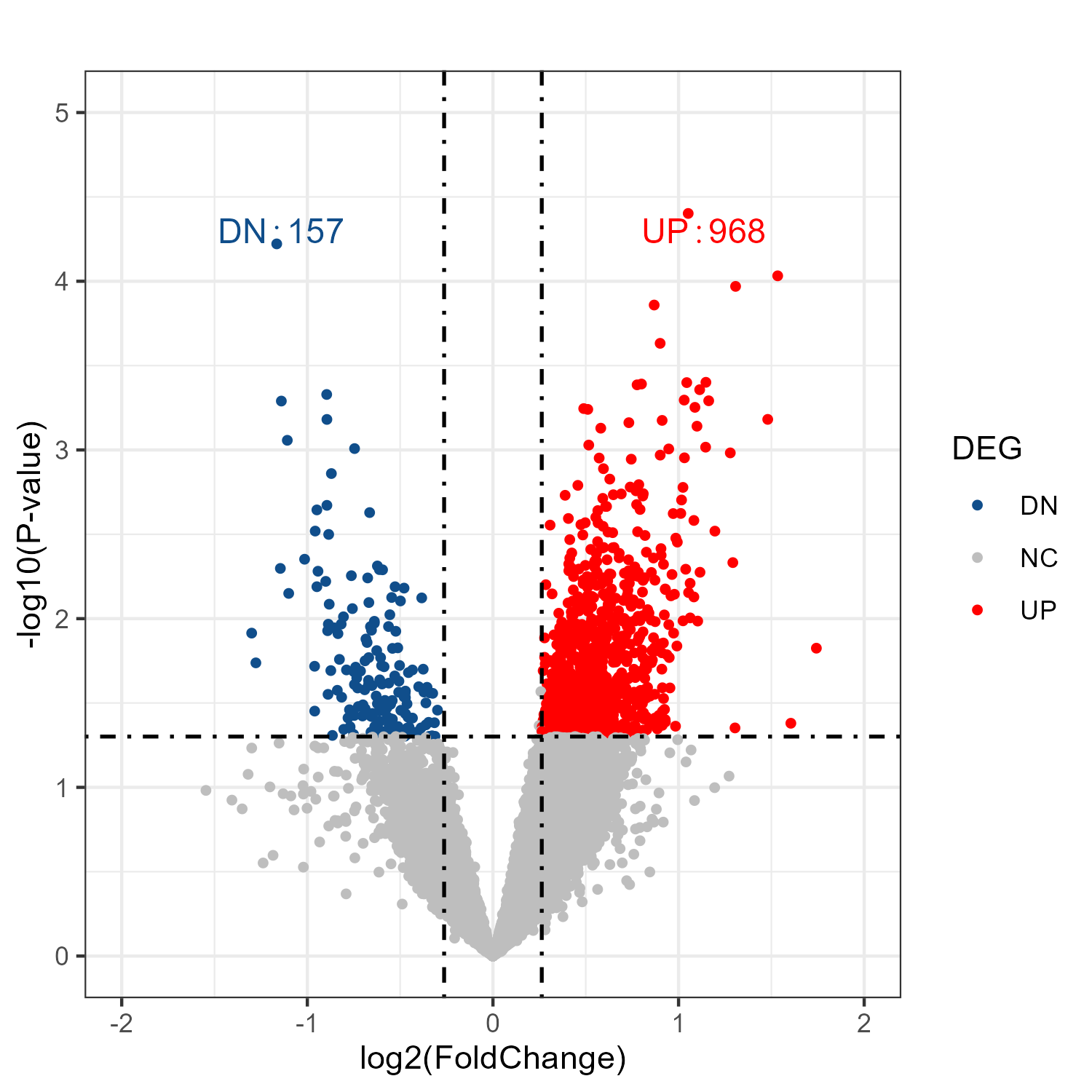

Supplement: Supplementary file 1 [file Supplementaryfile1.zip › Supplementary Material/01_DEG/2.3_Volcano_plot.png]

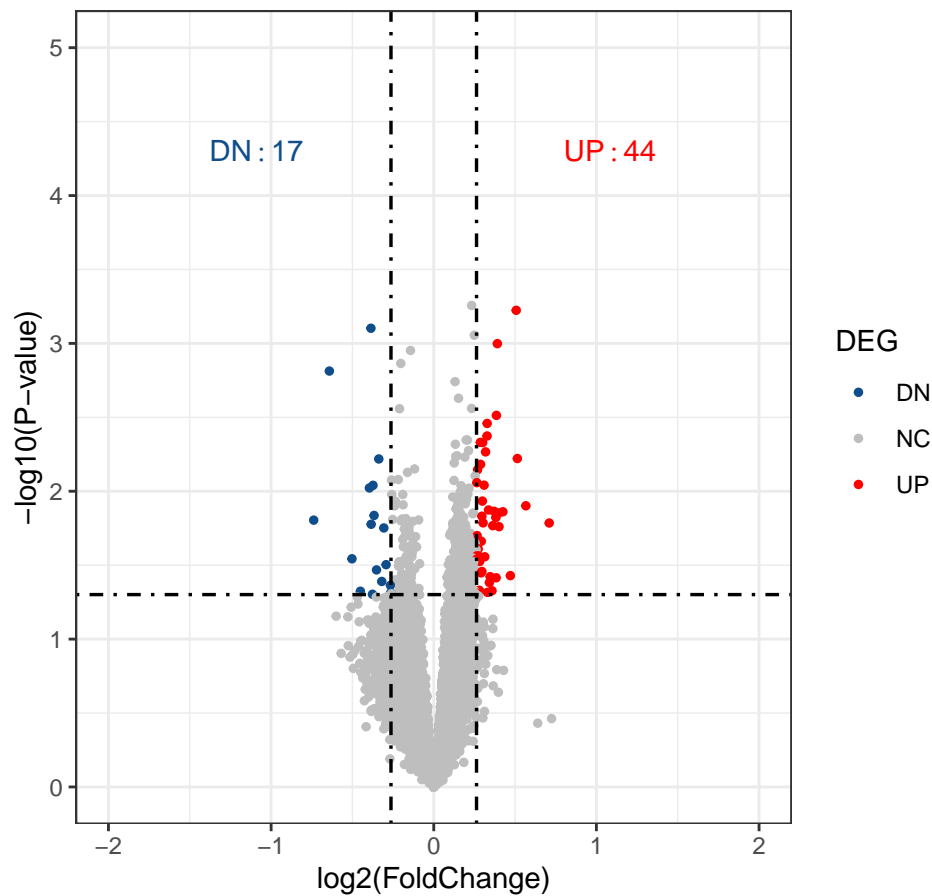

Supplement: Supplementary file 1 [file Supplementaryfile1.zip › Supplementary Material/01_DEG/3.3_Volcano_plot.pdf]

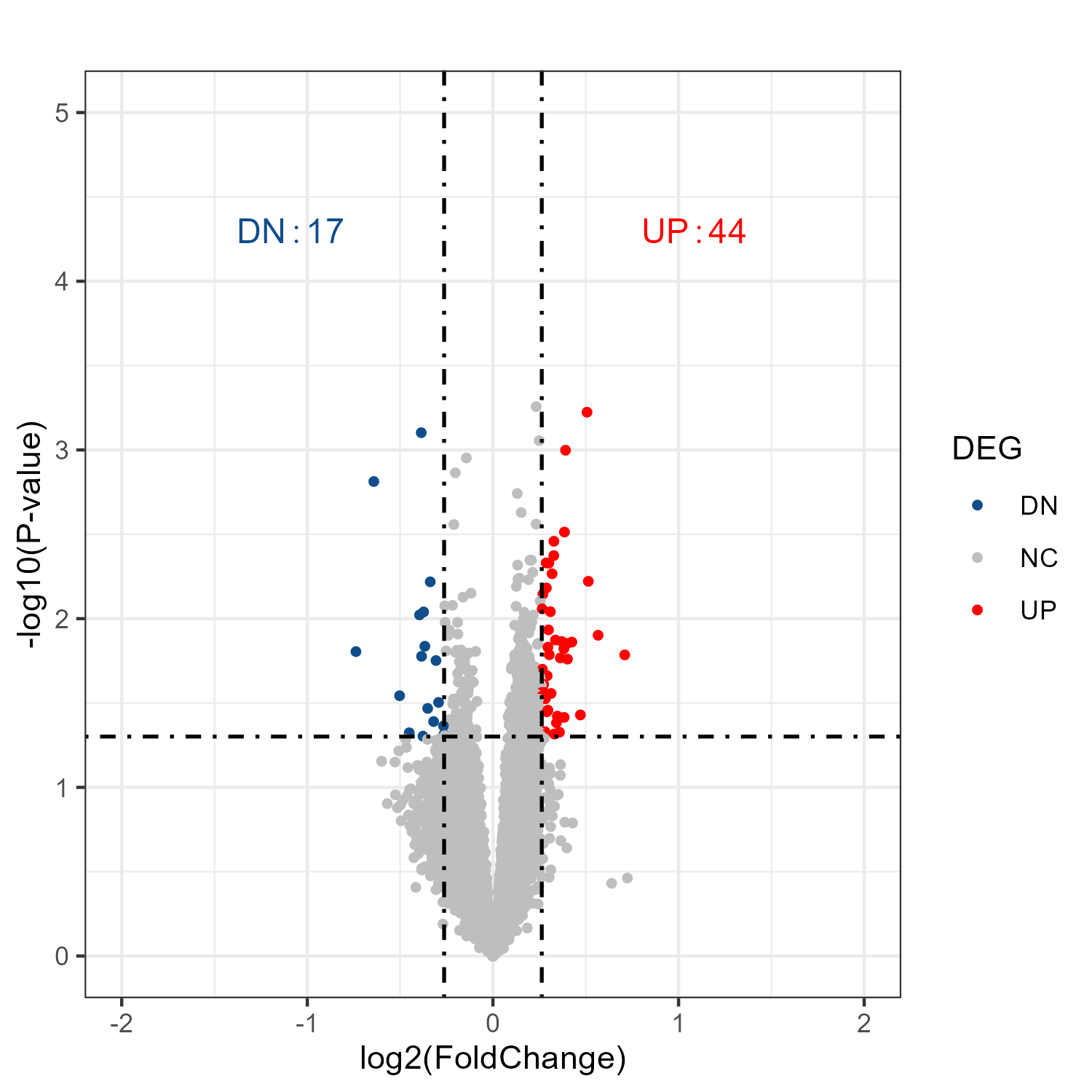

Supplement: Supplementary file 1 [file Supplementaryfile1.zip › Supplementary Material/01_DEG/3.3_Volcano_plot.png]

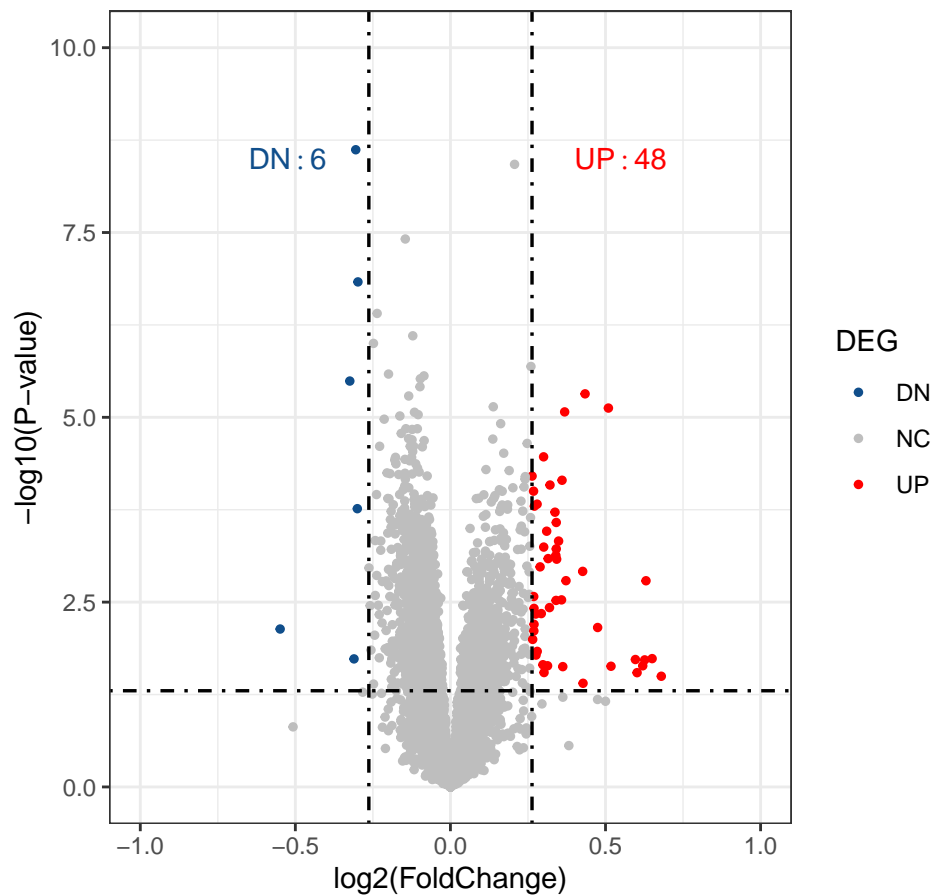

Supplement: Supplementary file 1 [file Supplementaryfile1.zip › Supplementary Material/01_DEG/4.3_Volcano_plot.pdf]

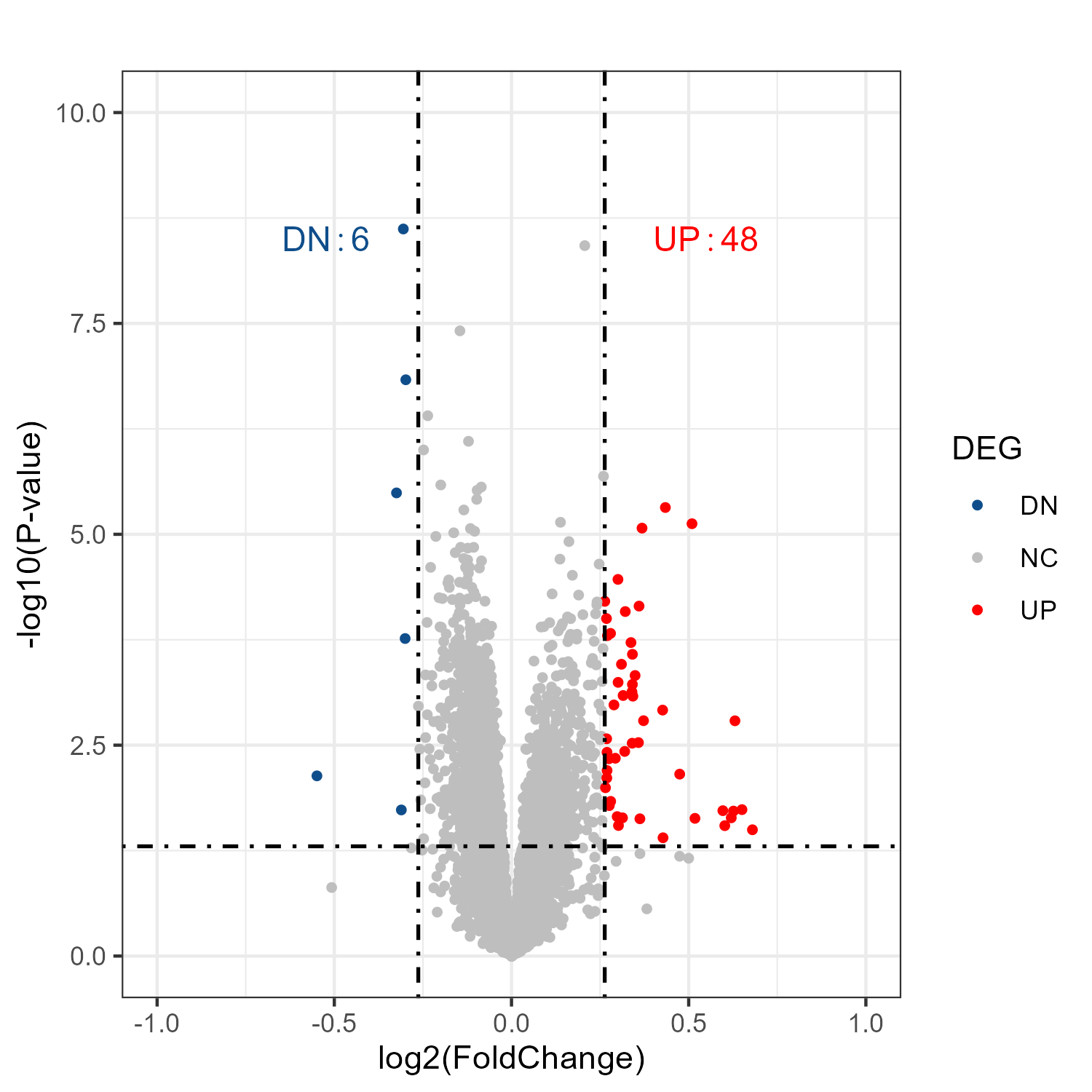

Supplement: Supplementary file 1 [file Supplementaryfile1.zip › Supplementary Material/01_DEG/4.3_Volcano_plot.png]

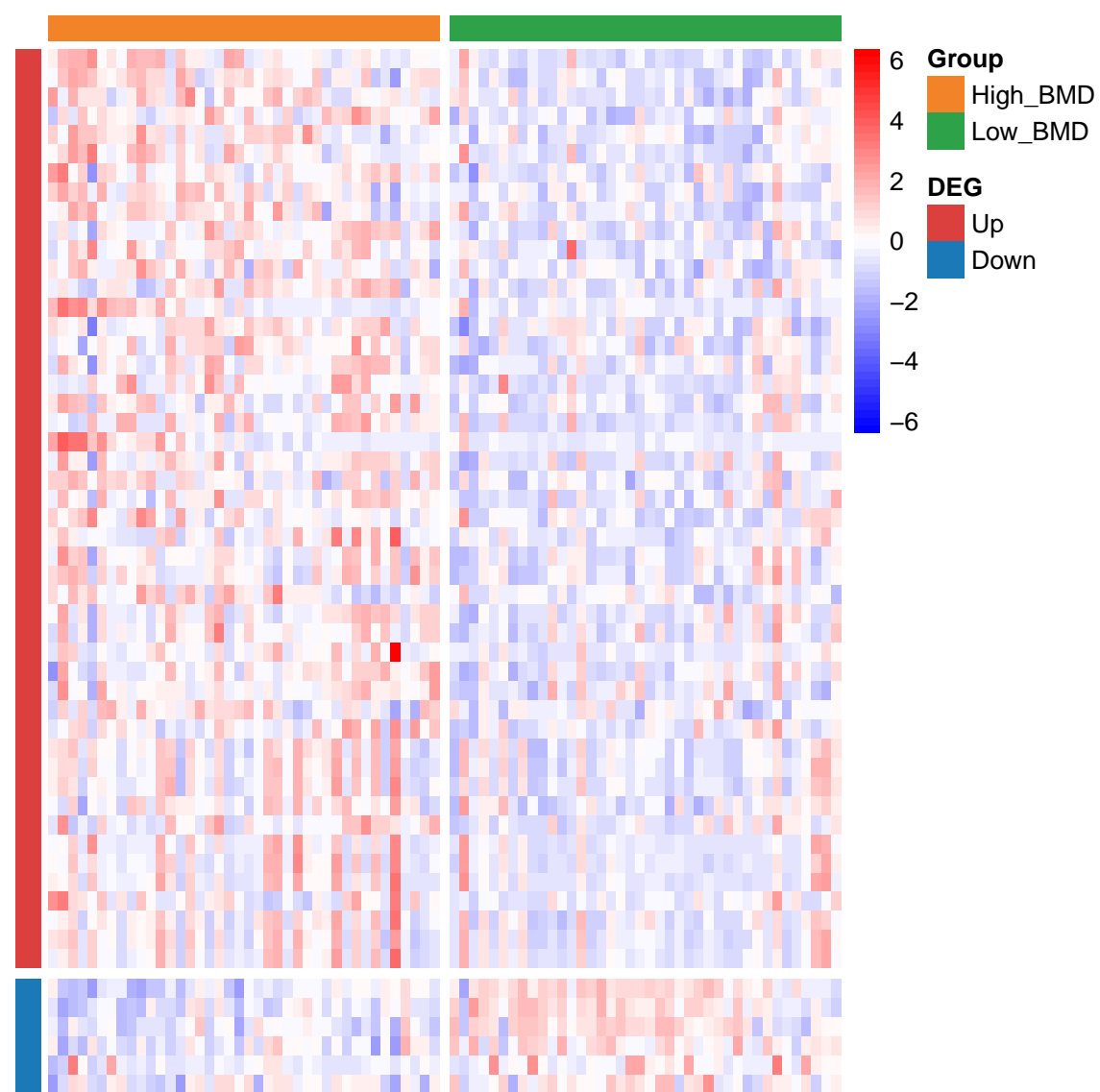

Supplement: Supplementary file 1 [file Supplementaryfile1.zip › Supplementary Material/01_DEG/4.4_Heatmap_DEG.pdf]

DEG\_CD

DEG\_OP

1658  
(96.8%)

8  
(0.5%)

46  
(2.7%)

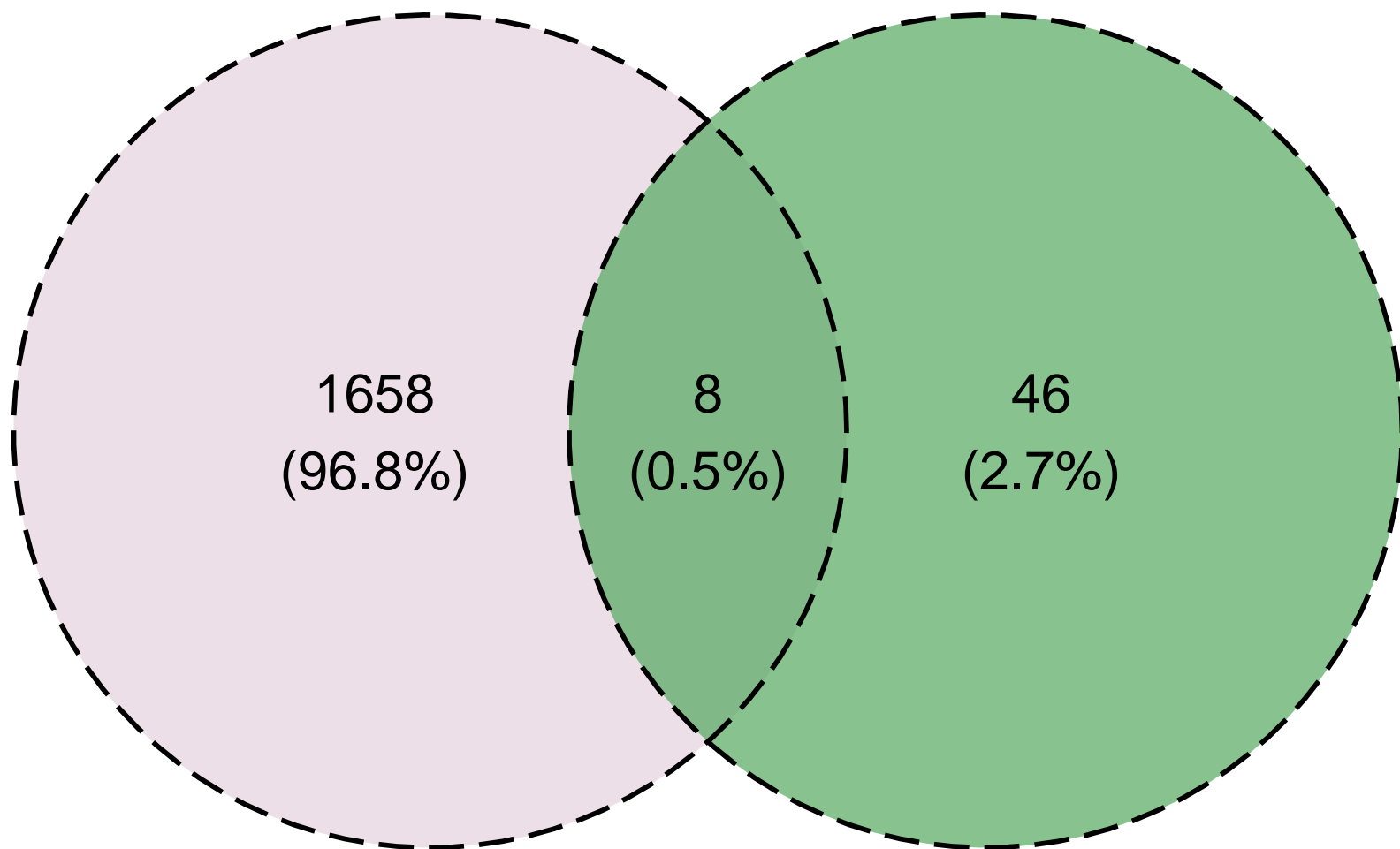

Supplement: Supplementary file 1 [file Supplementaryfile1.zip › Supplementary Material/01_DEG/5.2_Common_DEG_venn.pdf]

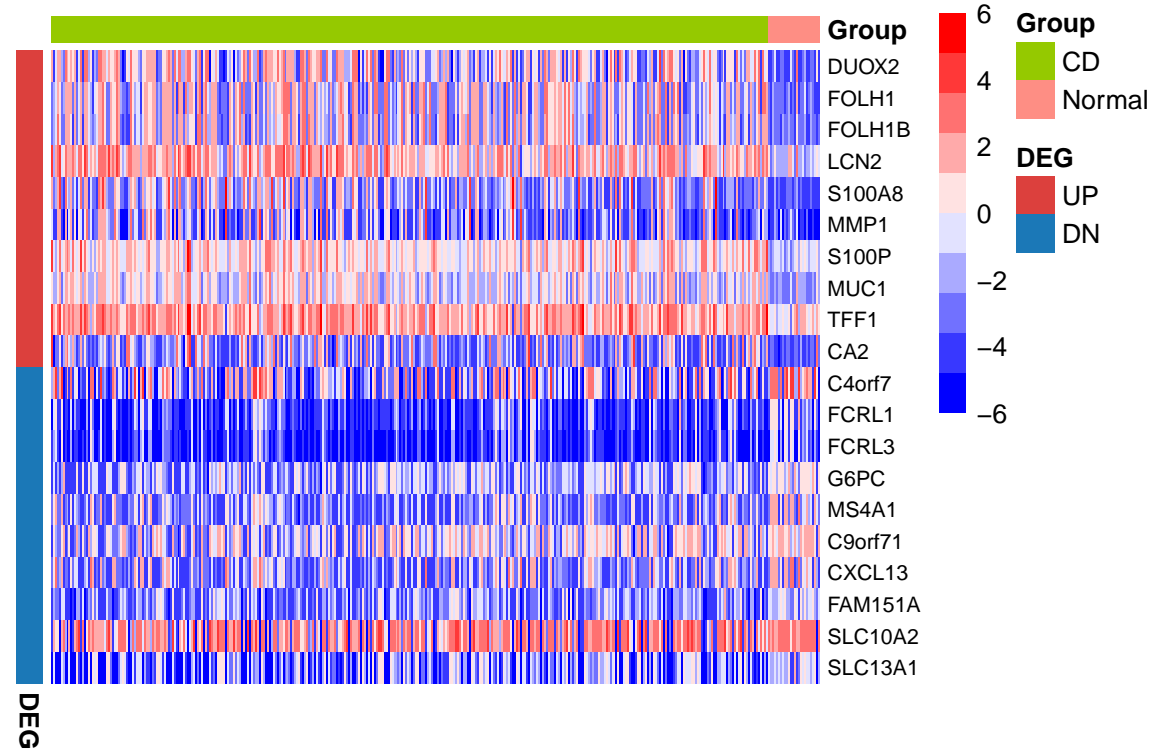

Supplement: Supplementary file 1 [file Supplementaryfile1.zip › Supplementary Material/01_DEG/Heatmap/CD-Heatmap-Top10.pdf]

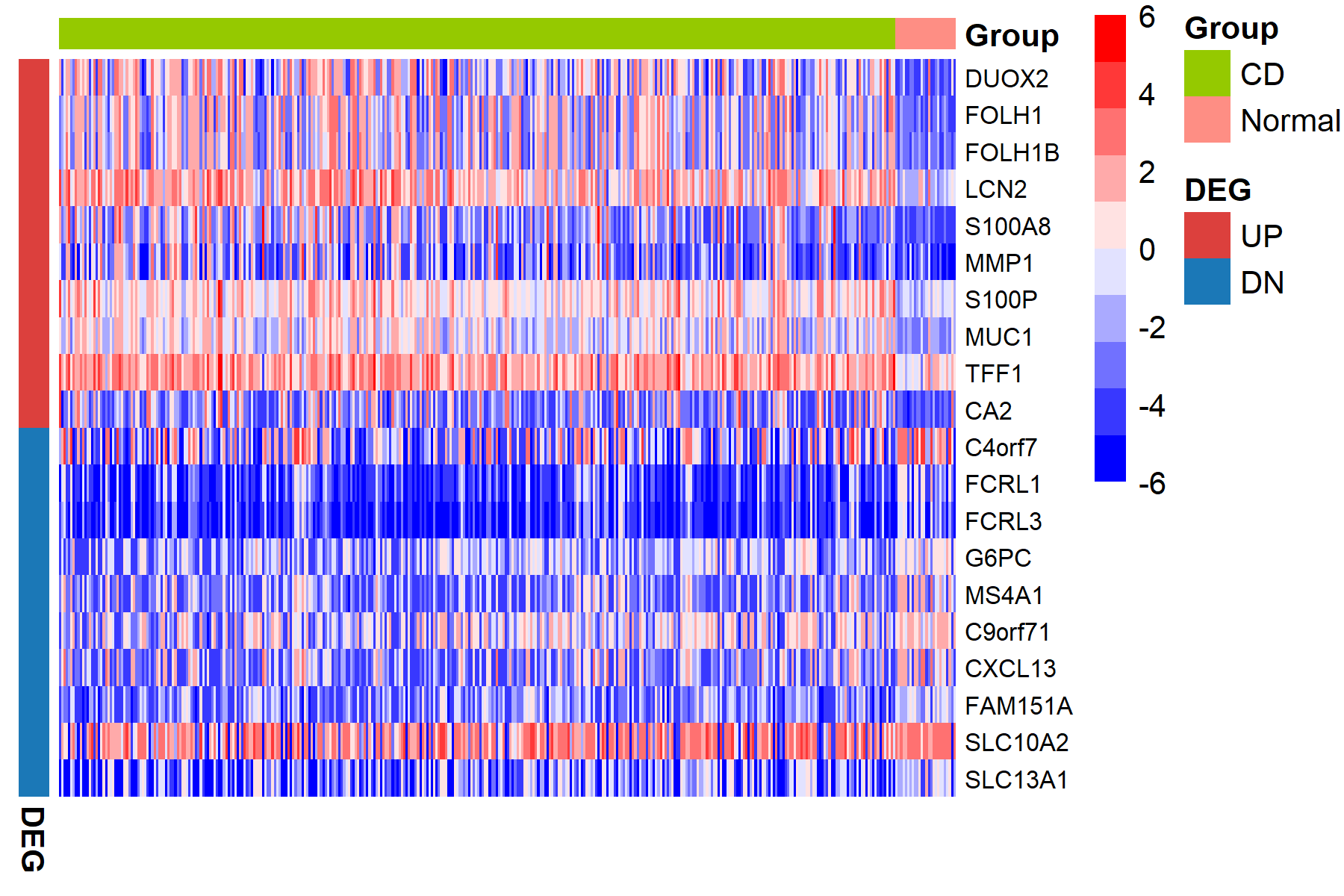

Supplement: Supplementary file 1 [file Supplementaryfile1.zip › Supplementary Material/01_DEG/Heatmap/CD-Heatmap-Top10.png]

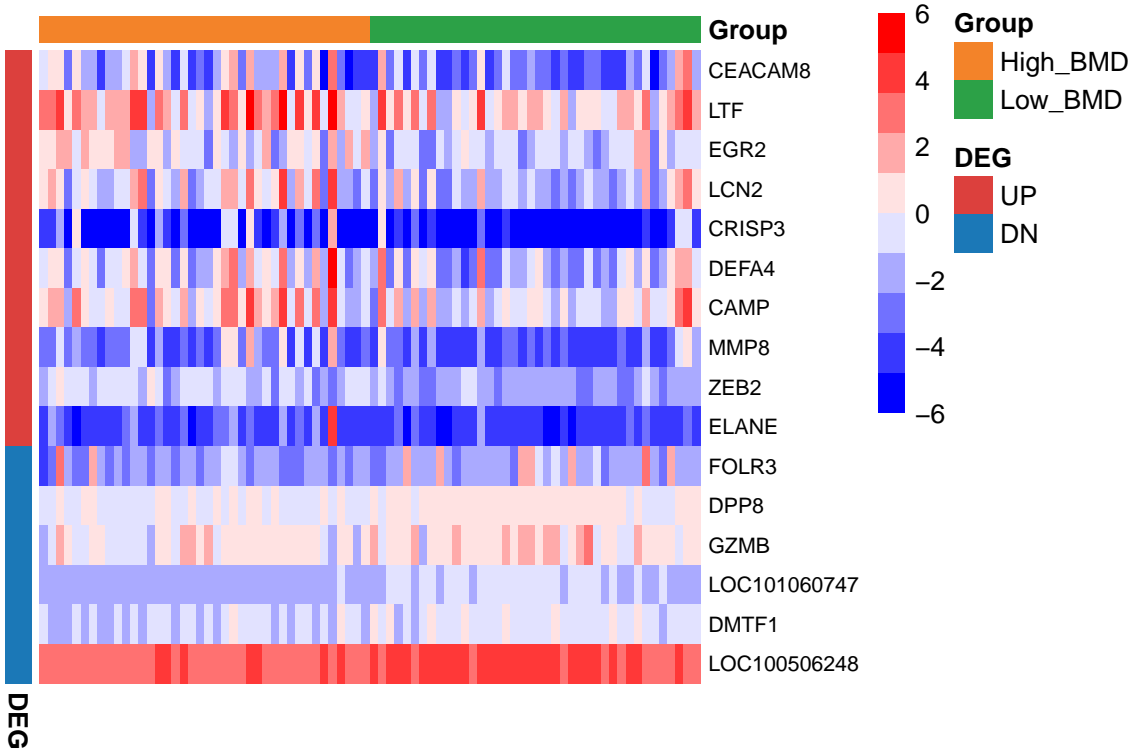

Supplement: Supplementary file 1 [file Supplementaryfile1.zip › Supplementary Material/01_DEG/Heatmap/OP-Heatmap-Top10.pdf]

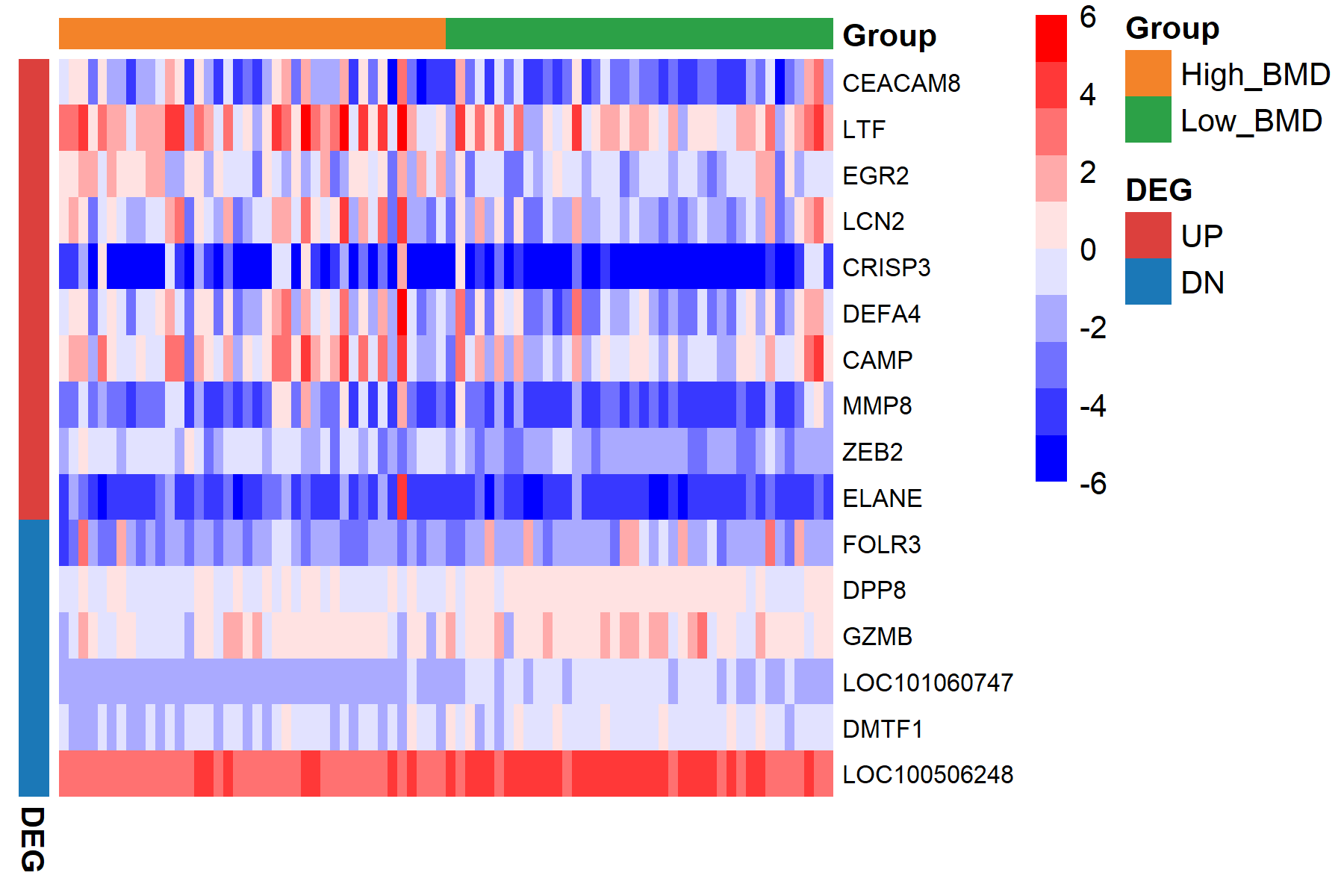

Supplement: Supplementary file 1 [file Supplementaryfile1.zip › Supplementary Material/01_DEG/Heatmap/OP-Heatmap-Top10.png]

# Sample clustering to detect outliers

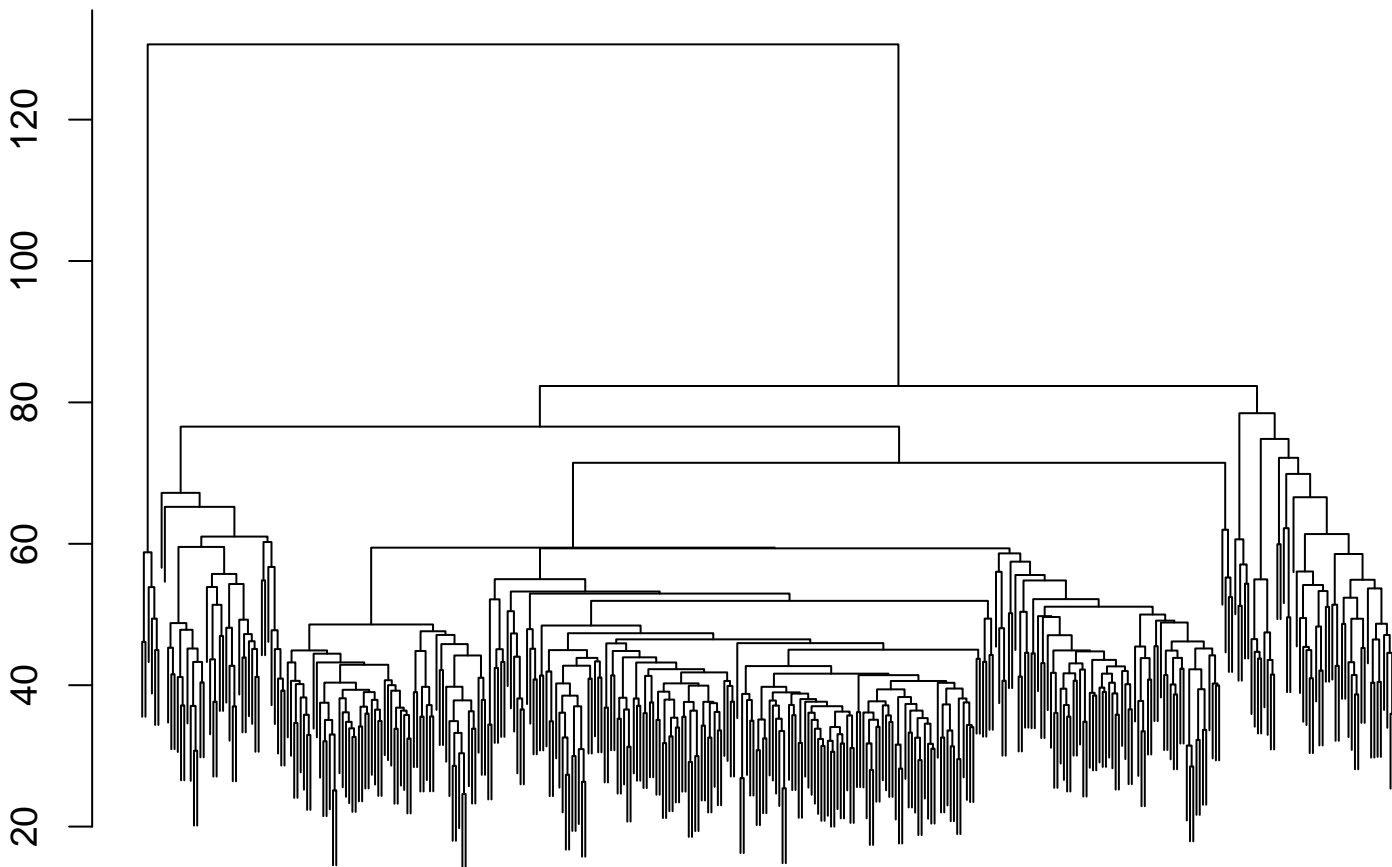

Supplement: Supplementary file 1 [file Supplementaryfile1.zip › Supplementary Material/02_WGCNA/1.1_WGCNA_CD/00_Clustering_Detect_outlier.pdf]

# Sample clustering to detect outliers

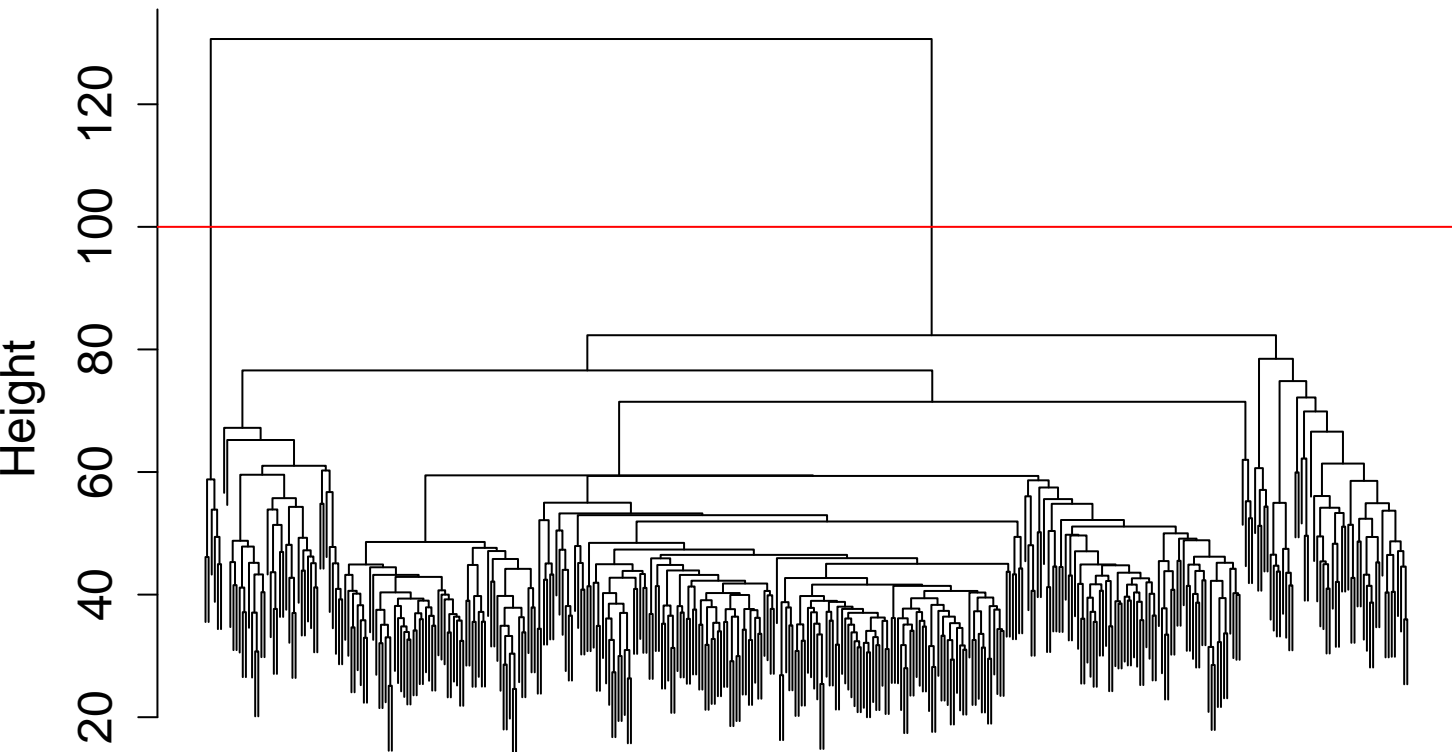

Supplement: Supplementary file 1 [file Supplementaryfile1.zip › Supplementary Material/02_WGCNA/1.1_WGCNA_CD/01_Clustering_show_outlier.pdf]

# Sample dendrogram and trait heatmap

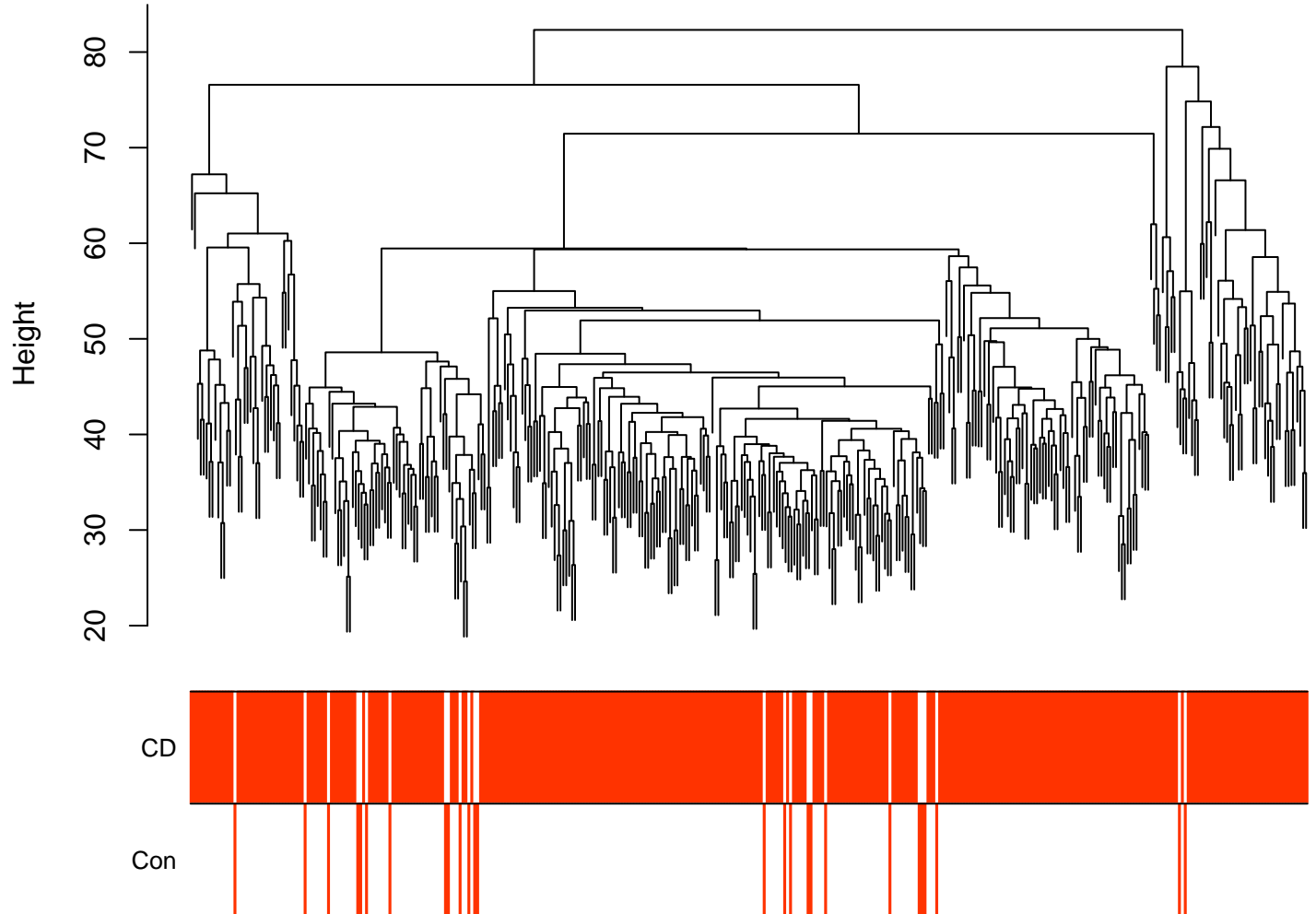

Supplement: Supplementary file 1 [file Supplementaryfile1.zip › Supplementary Material/02_WGCNA/1.1_WGCNA_CD/02_Clustering_with_pheno.pdf]

# Mean connectivity

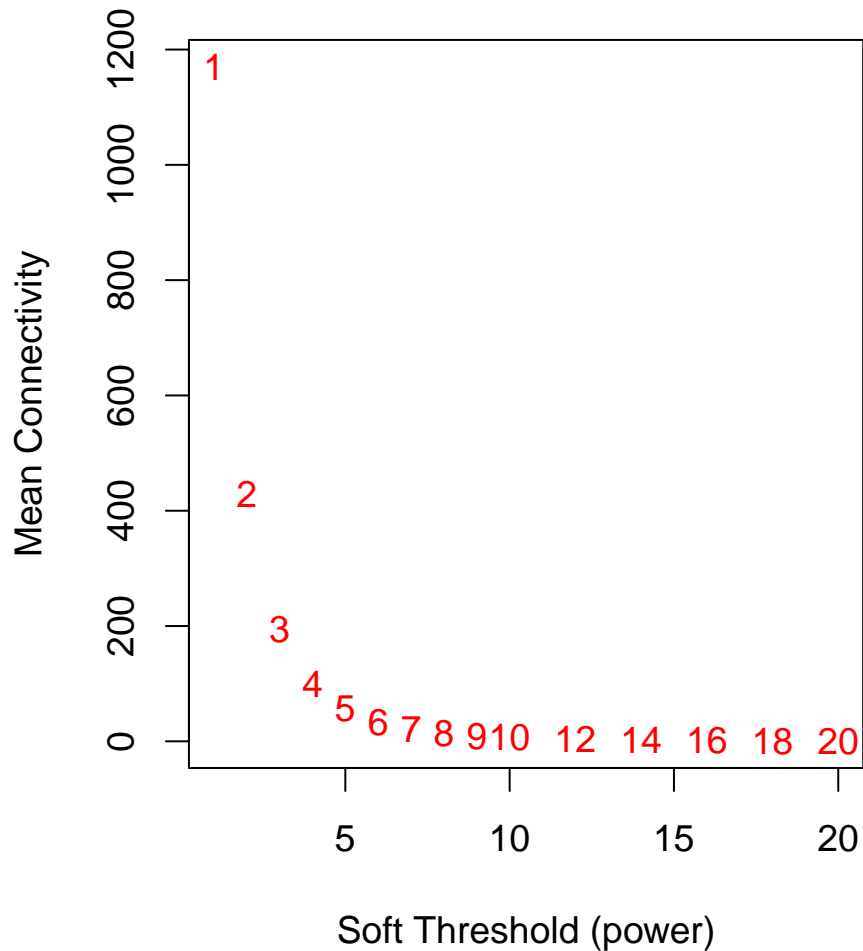

Supplement: Supplementary file 1 [file Supplementaryfile1.zip › Supplementary Material/02_WGCNA/1.1_WGCNA_CD/03_Mean_Connectivity.pdf]

## Scale independence

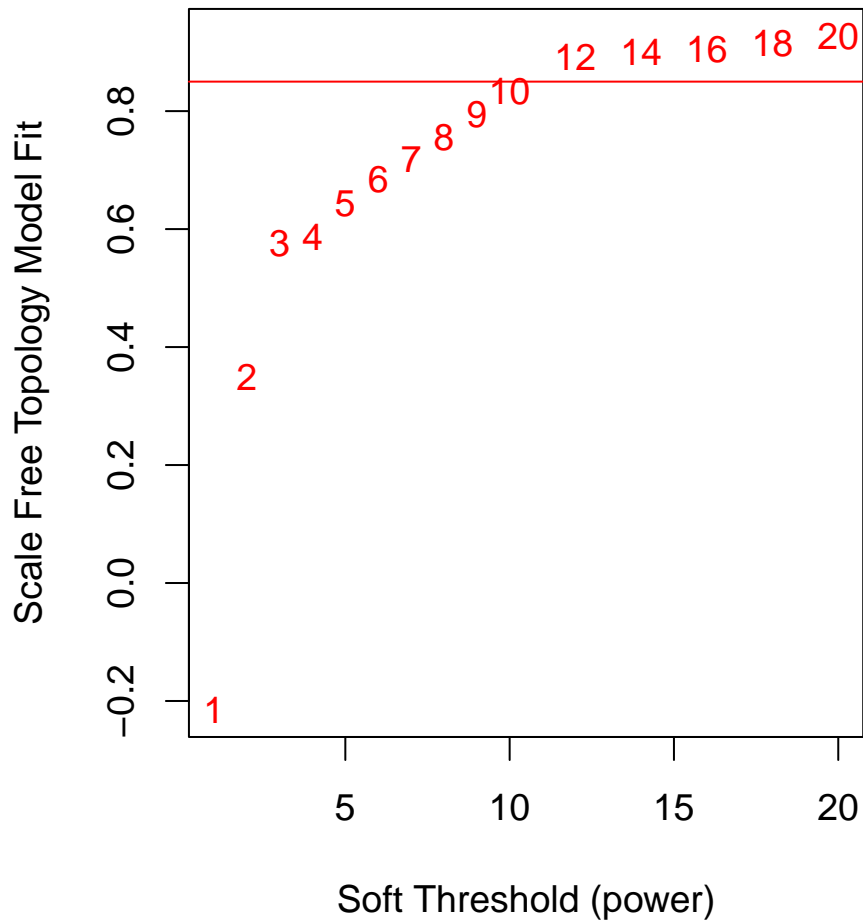

Supplement: Supplementary file 1 [file Supplementaryfile1.zip › Supplementary Material/02_WGCNA/1.1_WGCNA_CD/03_Scale_independence.pdf]

**Cluster Dendrogram**

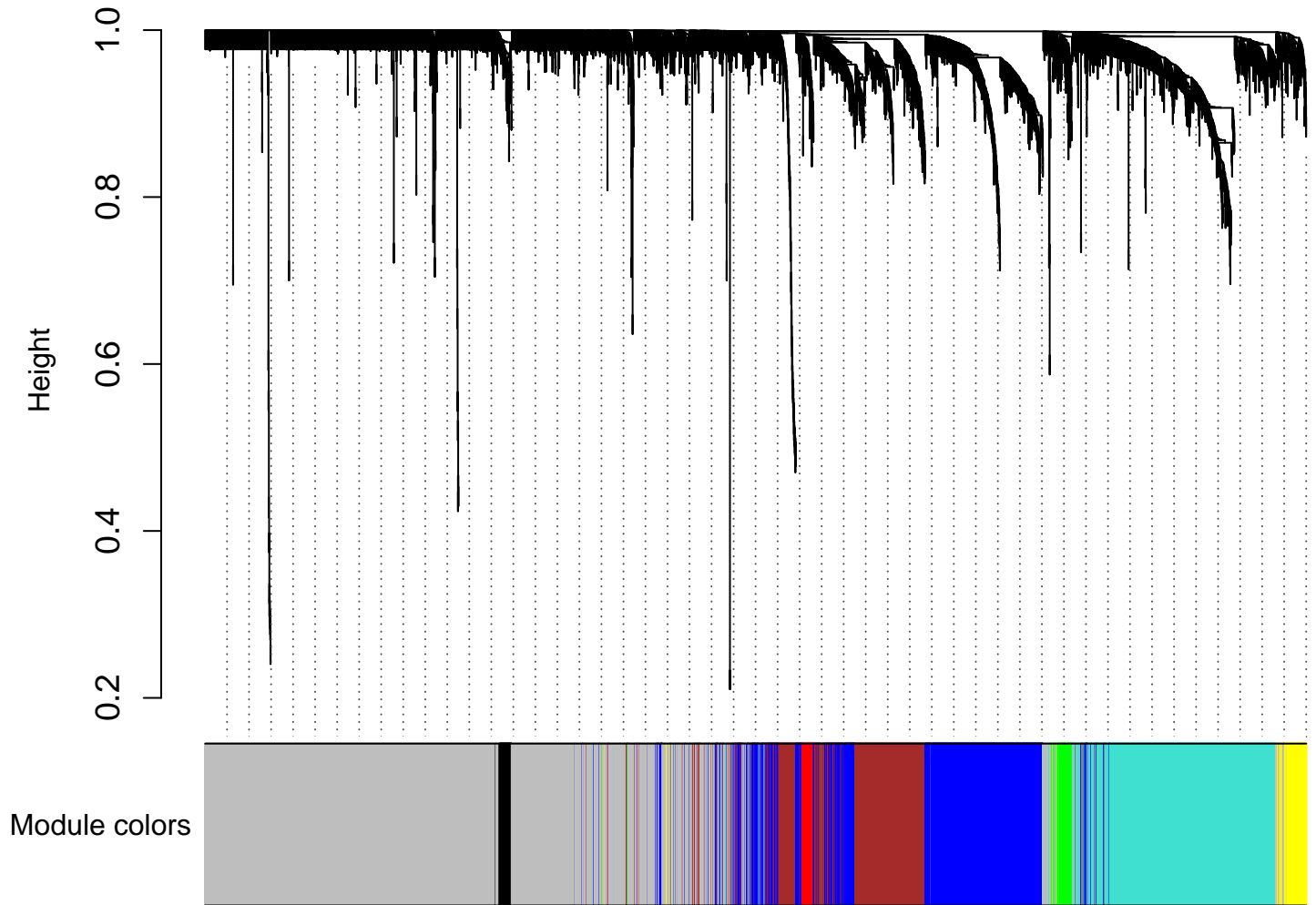

Supplement: Supplementary file 1 [file Supplementaryfile1.zip › Supplementary Material/02_WGCNA/1.1_WGCNA_CD/04_Cluster_dendrogram.pdf]

# Eigengene dendrogram

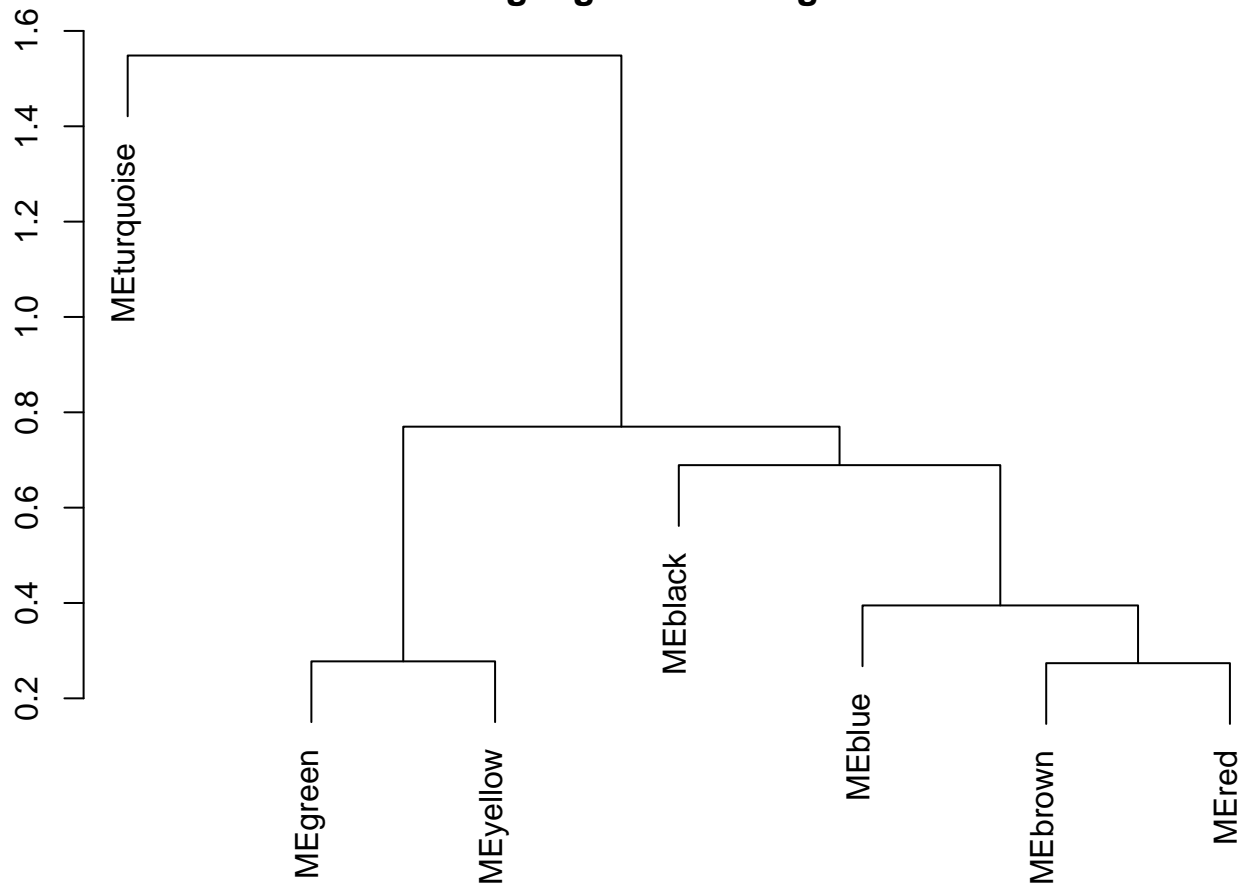

Supplement: Supplementary file 1 [file Supplementaryfile1.zip › Supplementary Material/02_WGCNA/1.1_WGCNA_CD/04_Eigengene dendrogram.pdf]

Module–trait relationships

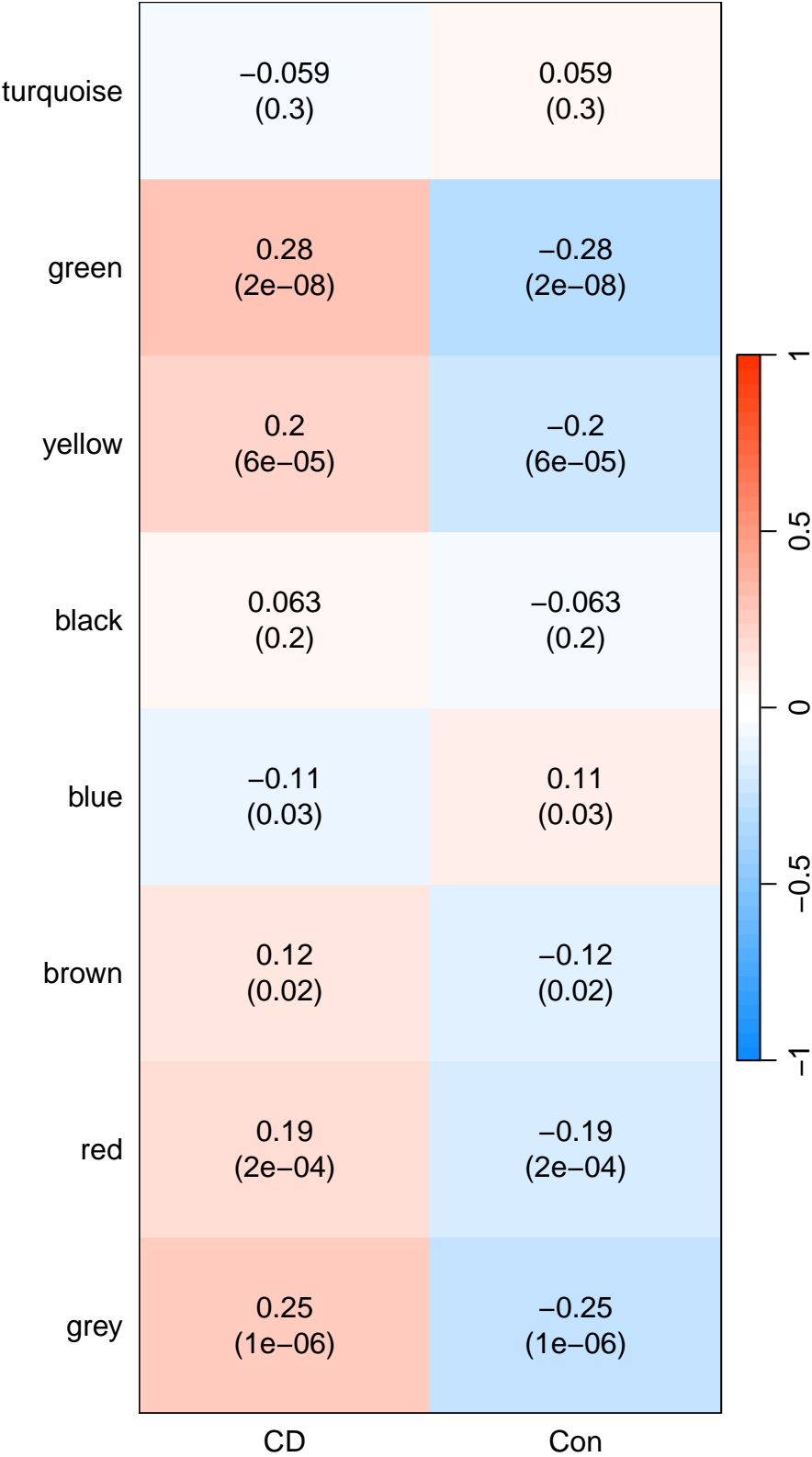

Supplement: Supplementary file 1 [file Supplementaryfile1.zip › Supplementary Material/02_WGCNA/1.1_WGCNA_CD/05_Module-trait_relationships.pdf]

# Sample clustering to detect outliers

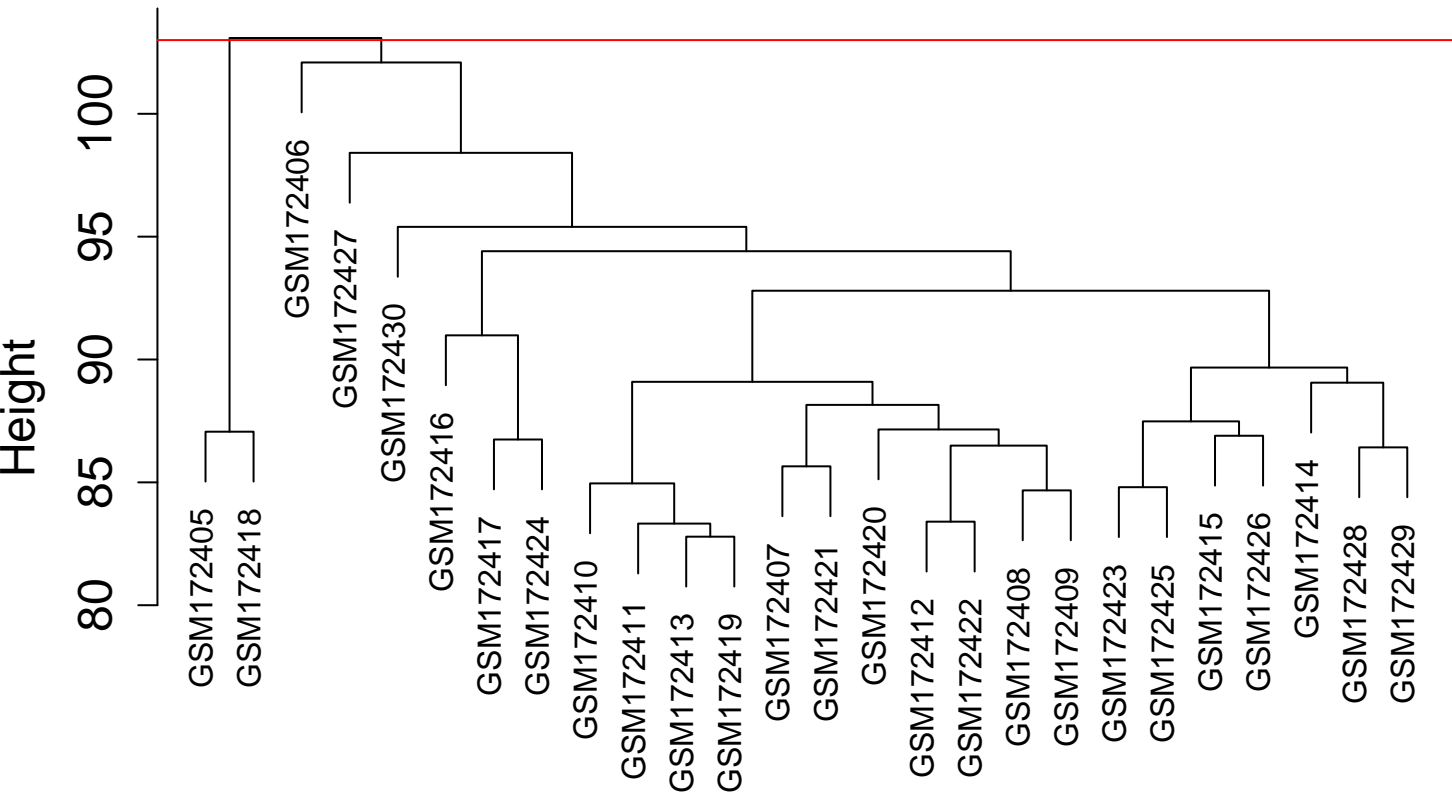

Supplement: Supplementary file 1 [file Supplementaryfile1.zip › Supplementary Material/02_WGCNA/2.1_WGCNA_OP/01_Clustering_show_outlier.pdf]

# Sample dendrogram and trait heatmap

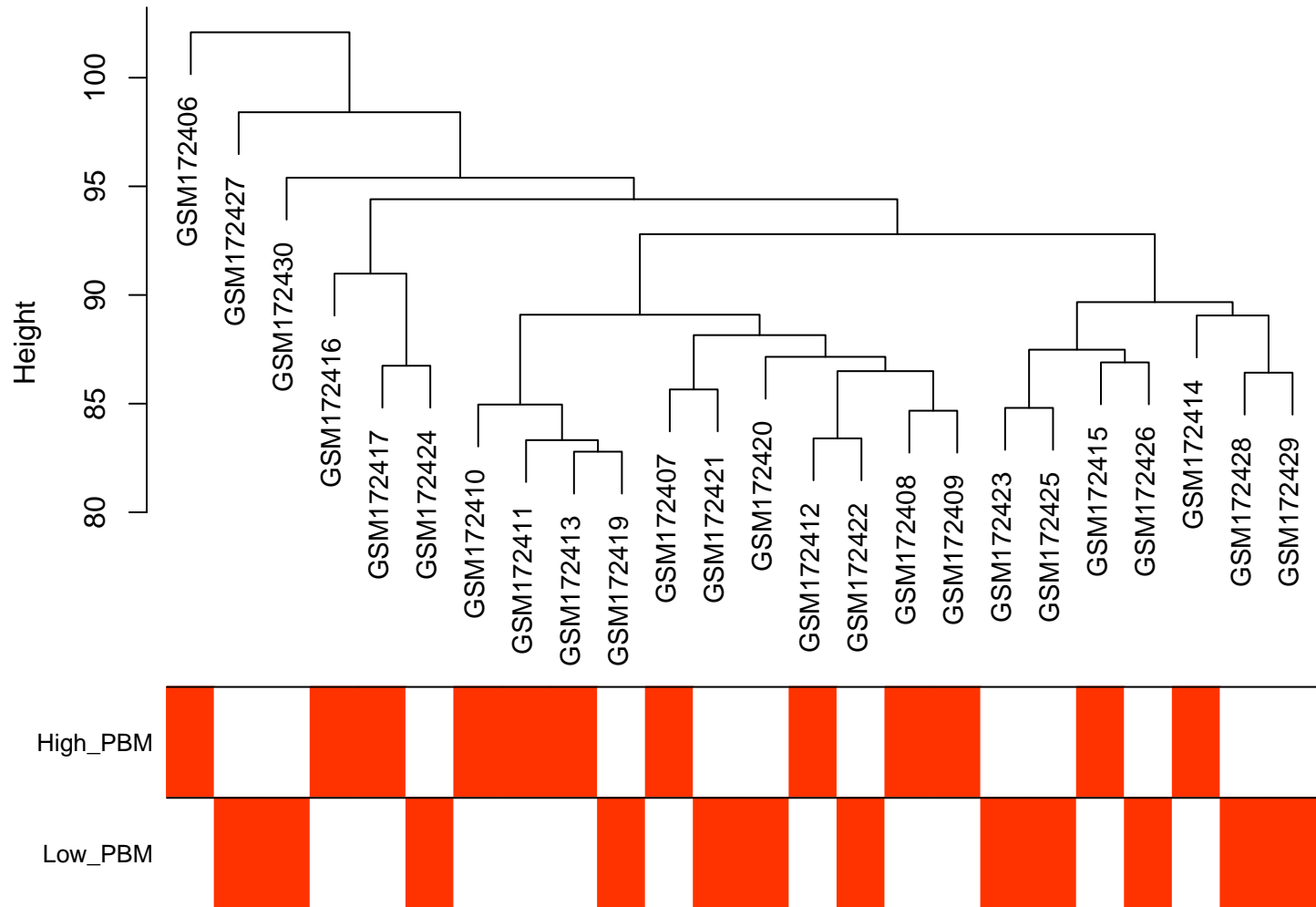

Supplement: Supplementary file 1 [file Supplementaryfile1.zip › Supplementary Material/02_WGCNA/2.1_WGCNA_OP/02_Clustering_with_pheno.pdf]

# Mean connectivity

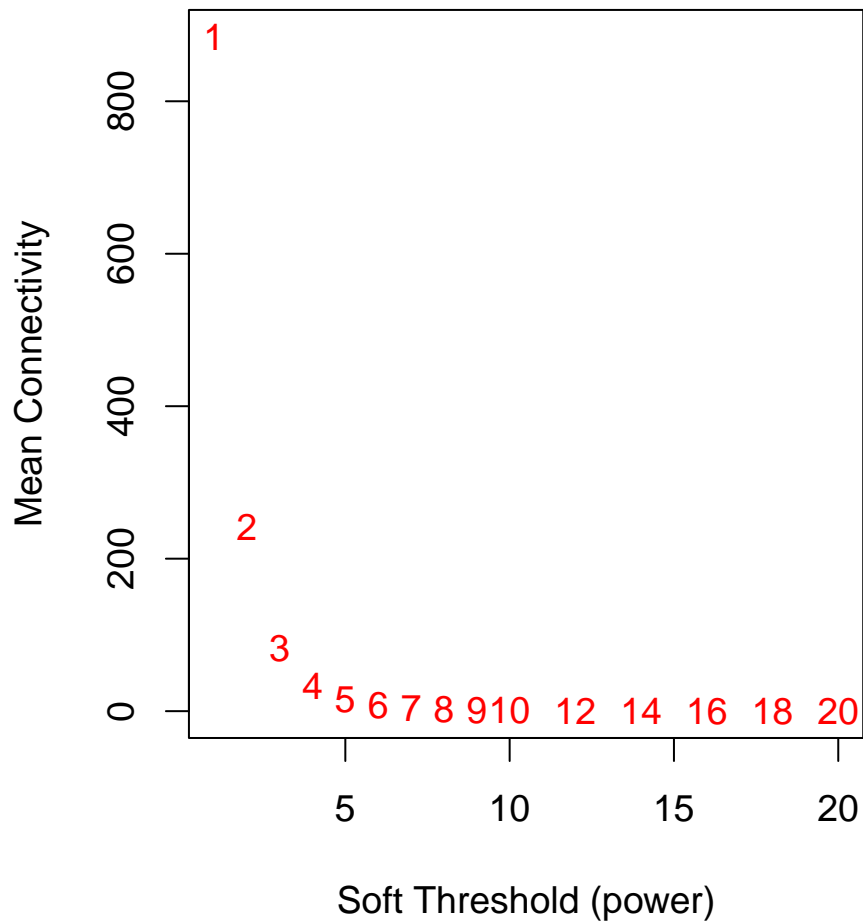

Supplement: Supplementary file 1 [file Supplementaryfile1.zip › Supplementary Material/02_WGCNA/2.1_WGCNA_OP/03_Mean_Connectivity.pdf]

## Scale independence

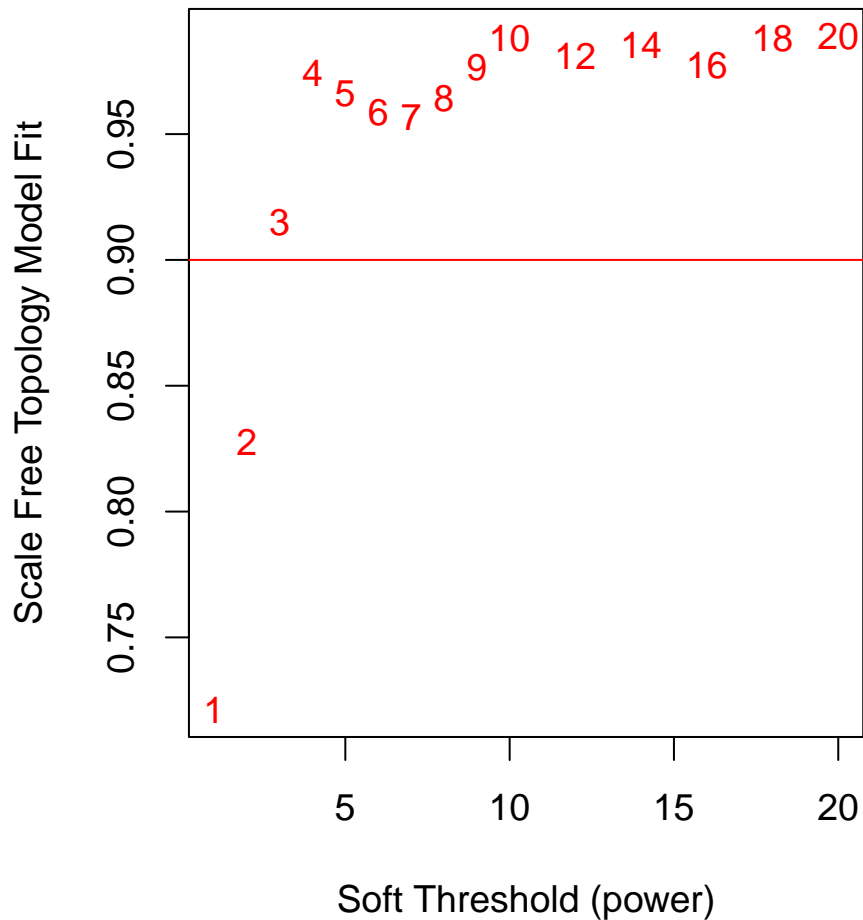

Supplement: Supplementary file 1 [file Supplementaryfile1.zip › Supplementary Material/02_WGCNA/2.1_WGCNA_OP/03_Scale_independence.pdf]

# Cluster Dendrogram

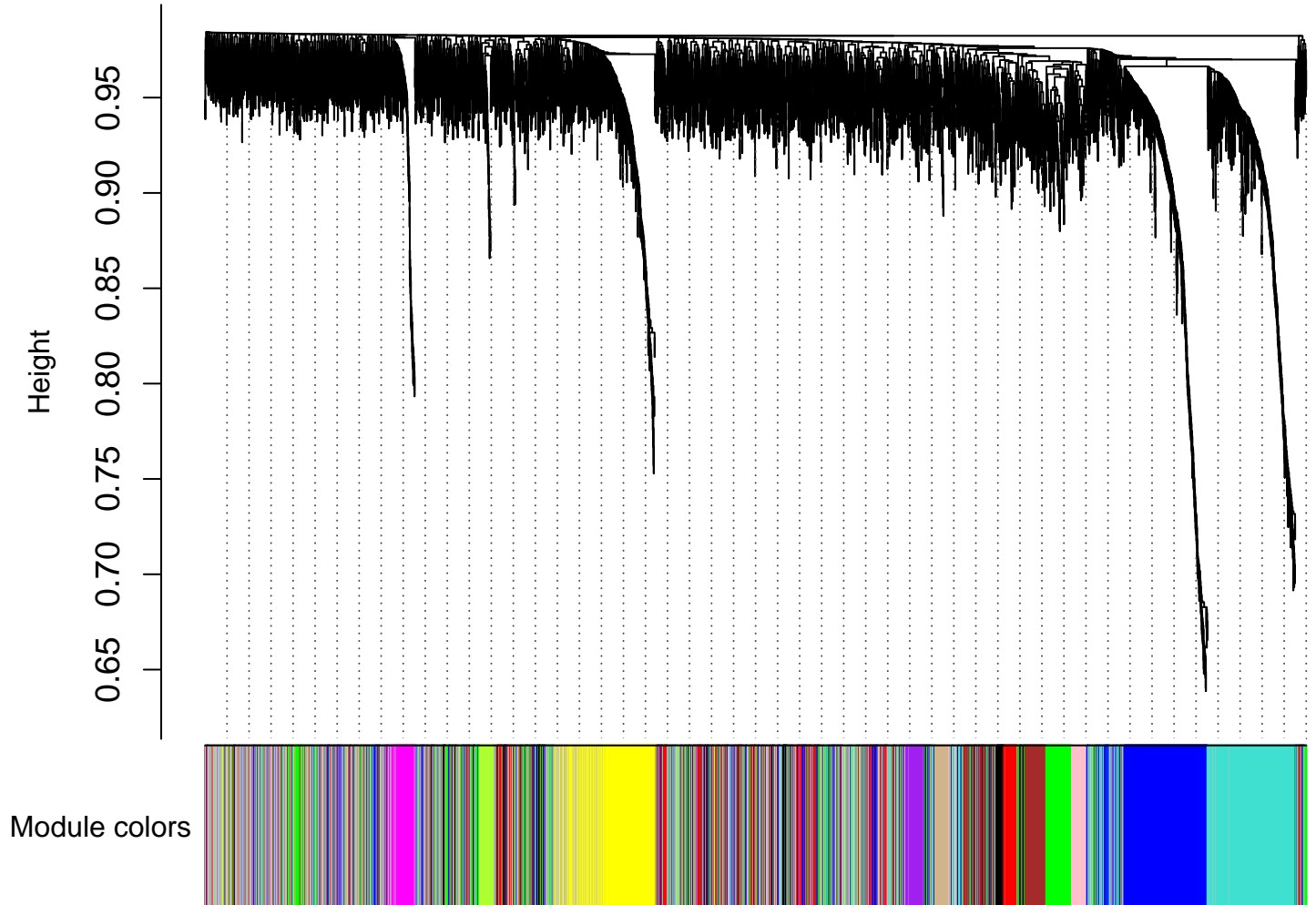

Supplement: Supplementary file 1 [file Supplementaryfile1.zip › Supplementary Material/02_WGCNA/2.1_WGCNA_OP/04_Cluster_dendrogram.pdf]

## Eigengene dendrogram

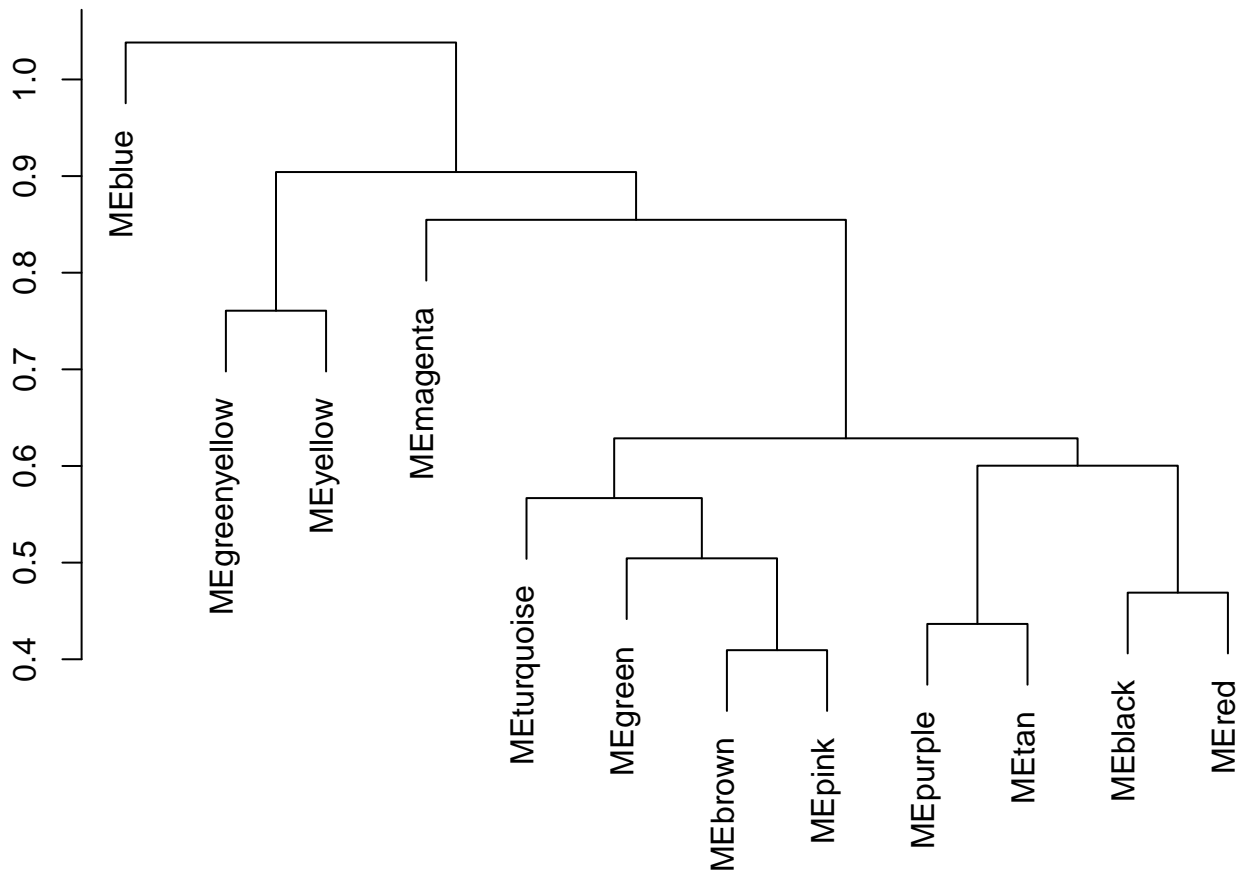

Supplement: Supplementary file 1 [file Supplementaryfile1.zip › Supplementary Material/02_WGCNA/2.1_WGCNA_OP/04_Eigengene dendrogram.pdf]

Module–trait relationships

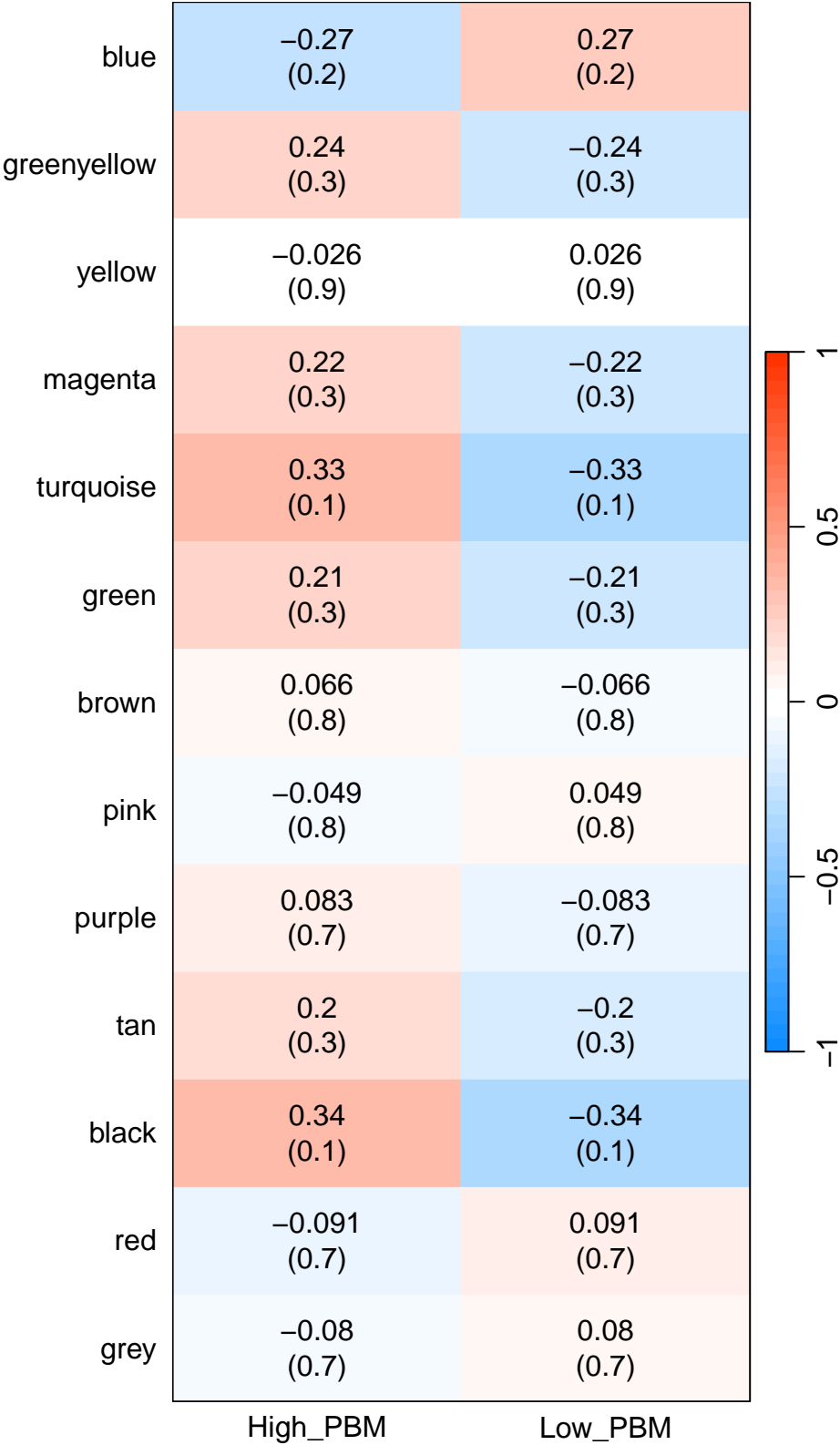

Supplement: Supplementary file 1 [file Supplementaryfile1.zip › Supplementary Material/02_WGCNA/2.1_WGCNA_OP/05_Module-trait_relationships.pdf]

# Sample clustering to detect outliers

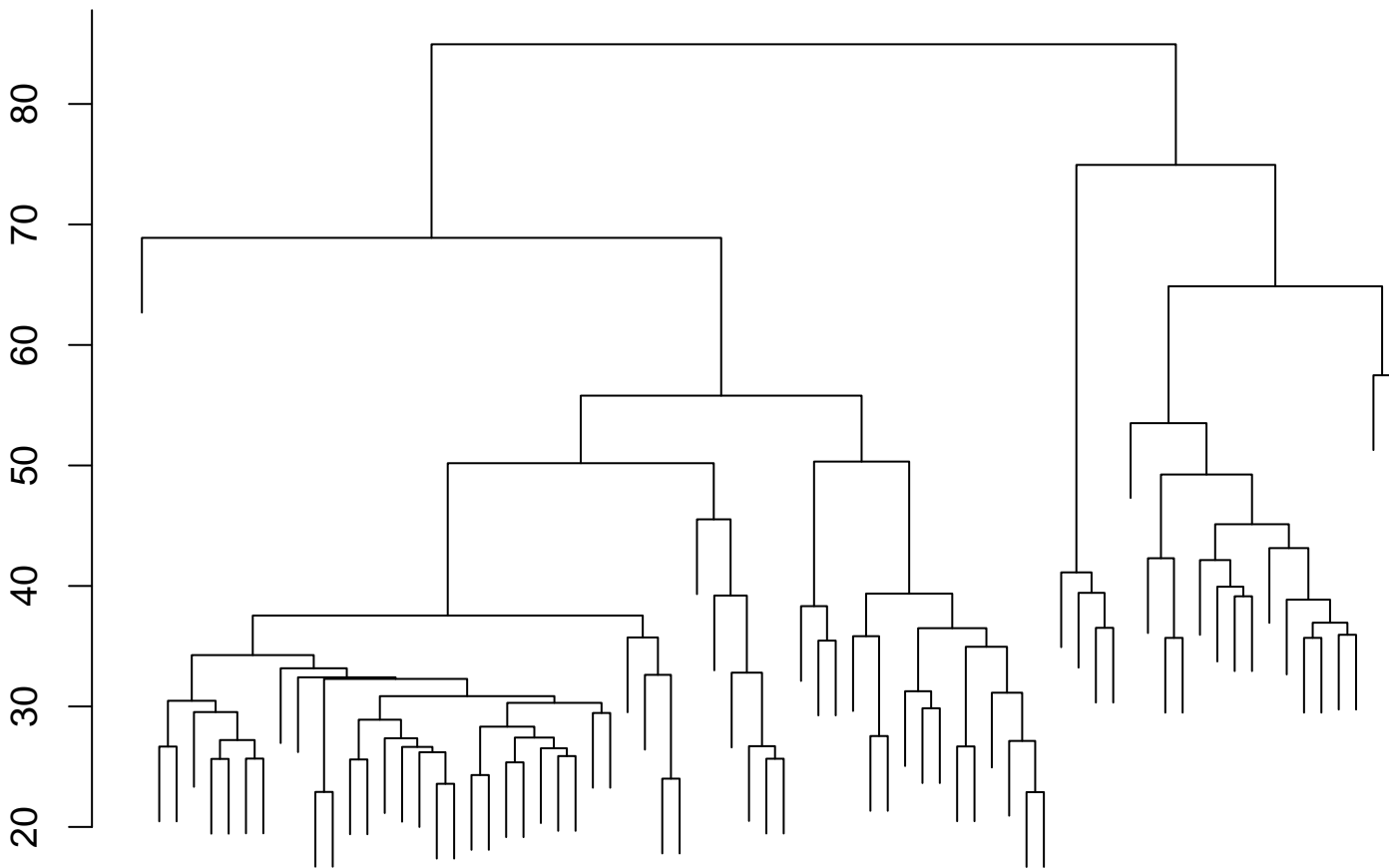

Supplement: Supplementary file 1 [file Supplementaryfile1.zip › Supplementary Material/02_WGCNA/3.1_WGCNA_OP_GSE56814/00_Clustering_Detect_outlier.pdf]

# Sample clustering to detect outliers

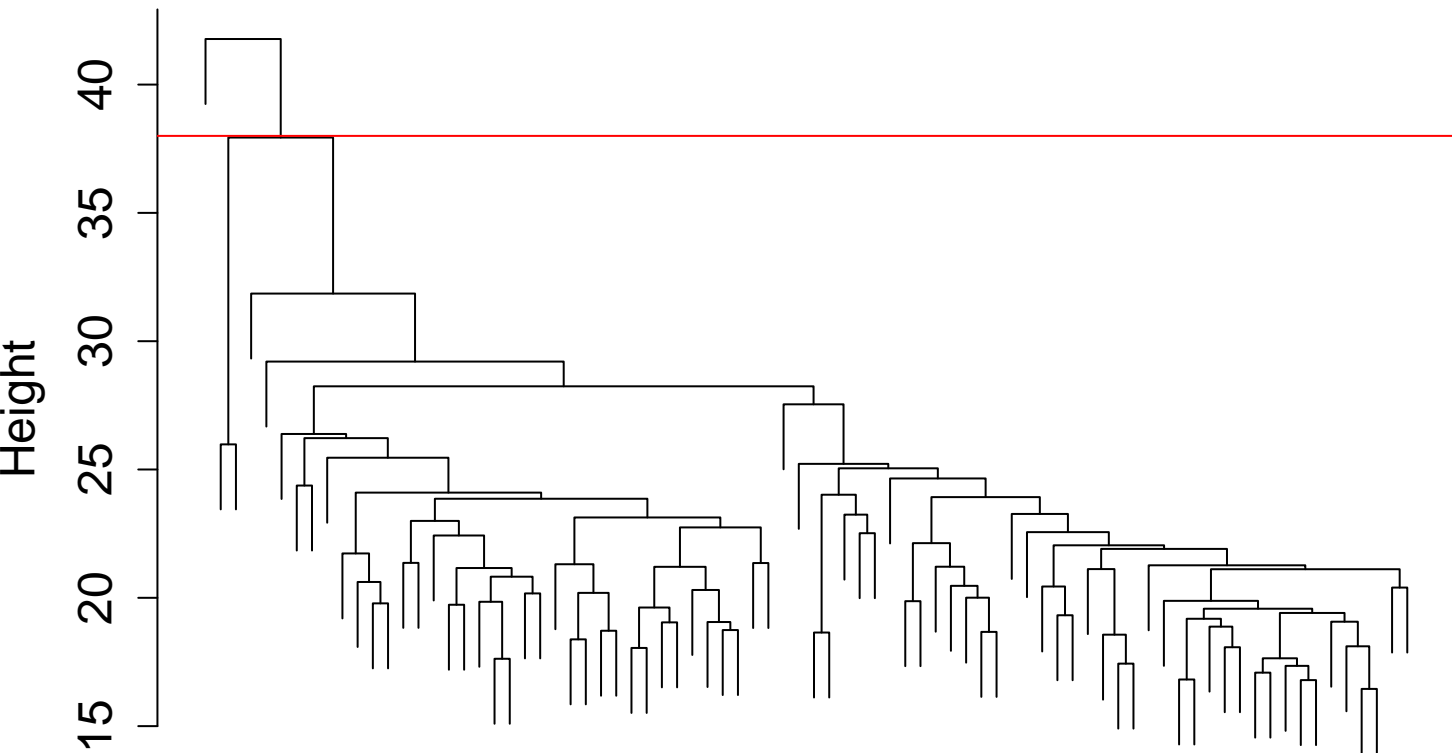

Supplement: Supplementary file 1 [file Supplementaryfile1.zip › Supplementary Material/02_WGCNA/3.1_WGCNA_OP_GSE56814/01_Clustering_show_outlier.pdf]

**Sample dendrogram and trait heatmap**

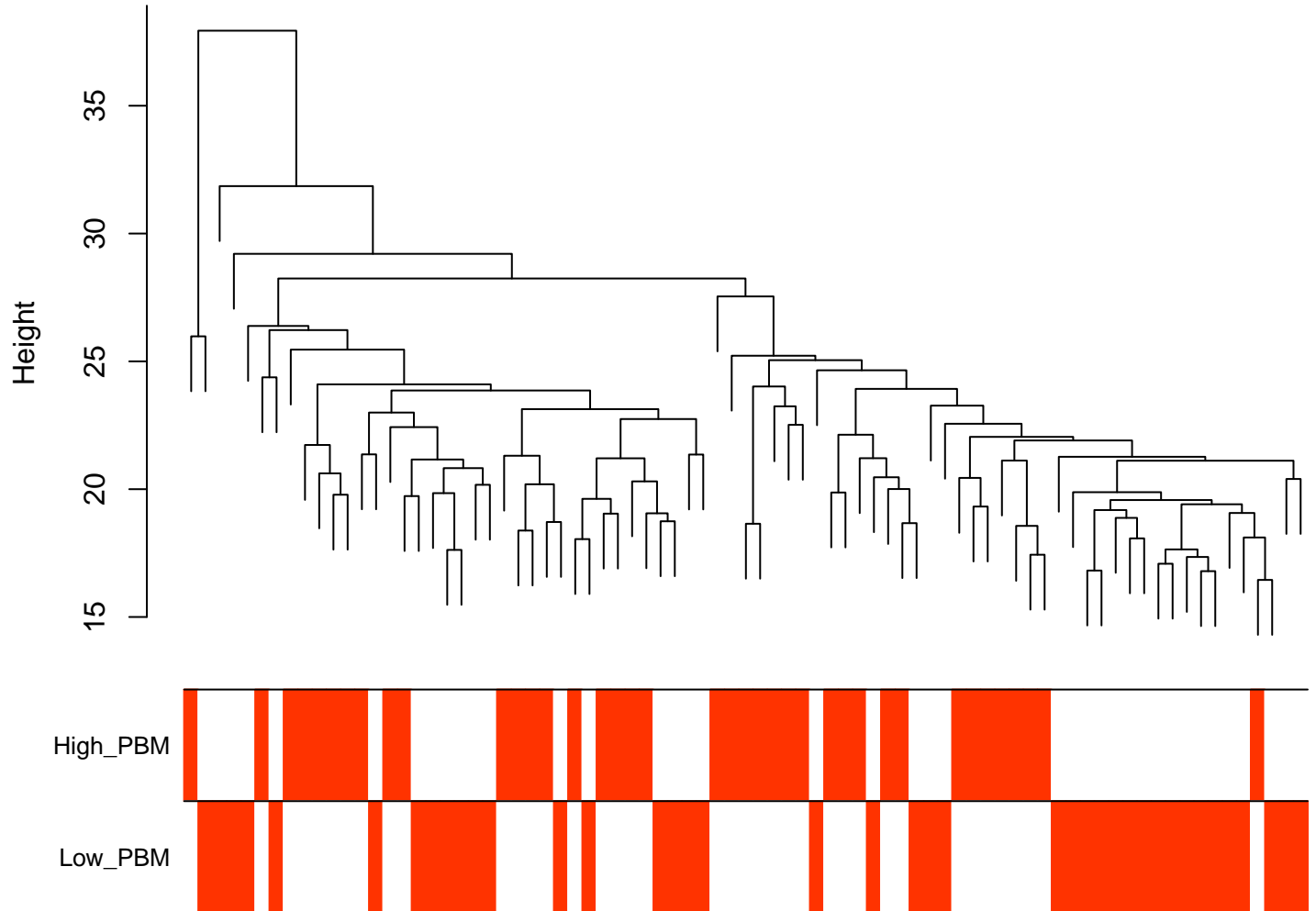

Supplement: Supplementary file 1 [file Supplementaryfile1.zip › Supplementary Material/02_WGCNA/3.1_WGCNA_OP_GSE56814/02_Clustering_with_pheno.pdf]

# Mean connectivity

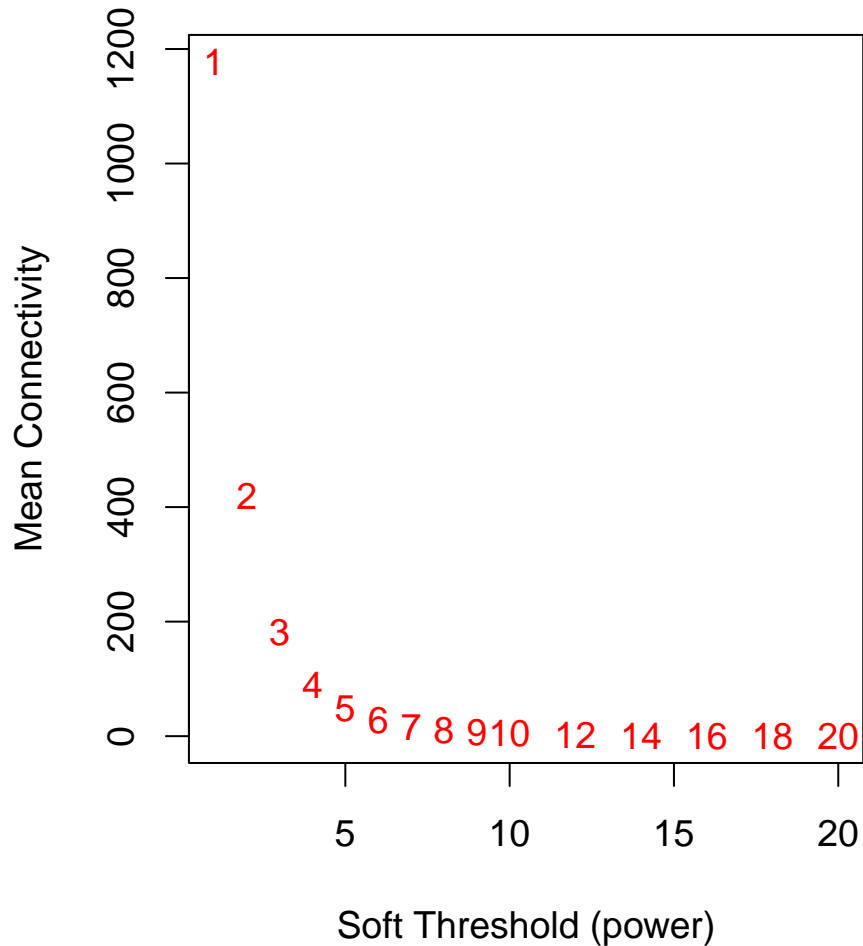

Supplement: Supplementary file 1 [file Supplementaryfile1.zip › Supplementary Material/02_WGCNA/3.1_WGCNA_OP_GSE56814/03_Mean_Connectivity.pdf]

## Scale independence

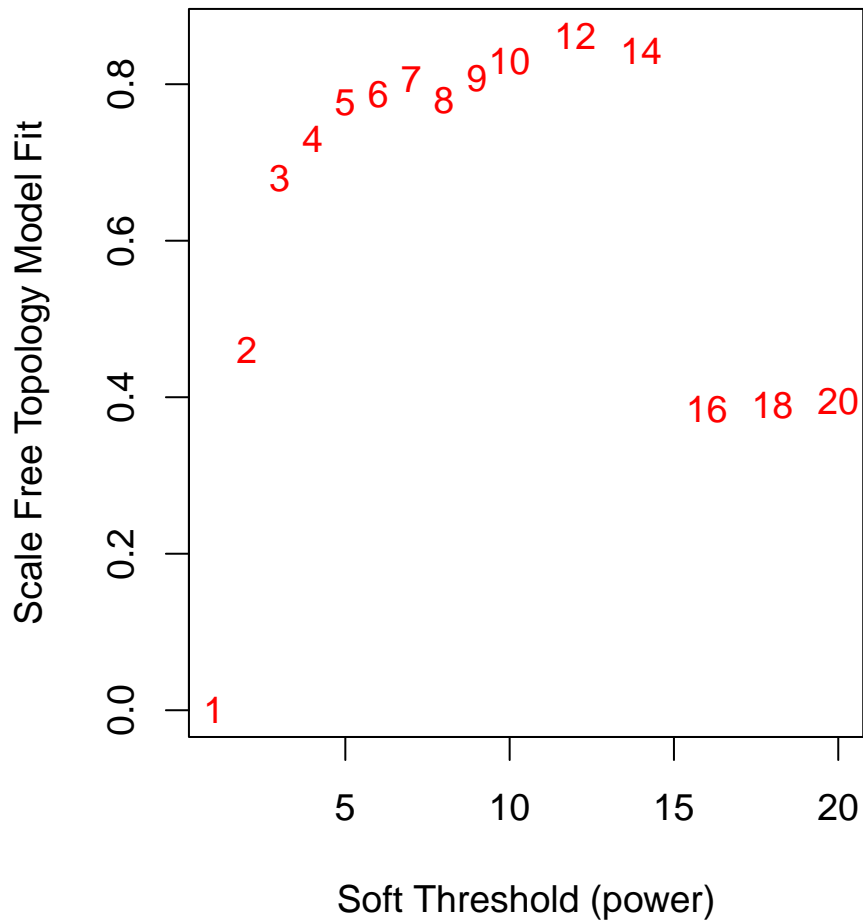

Supplement: Supplementary file 1 [file Supplementaryfile1.zip › Supplementary Material/02_WGCNA/3.1_WGCNA_OP_GSE56814/03_Scale_independence.pdf]

# Cluster Dendrogram

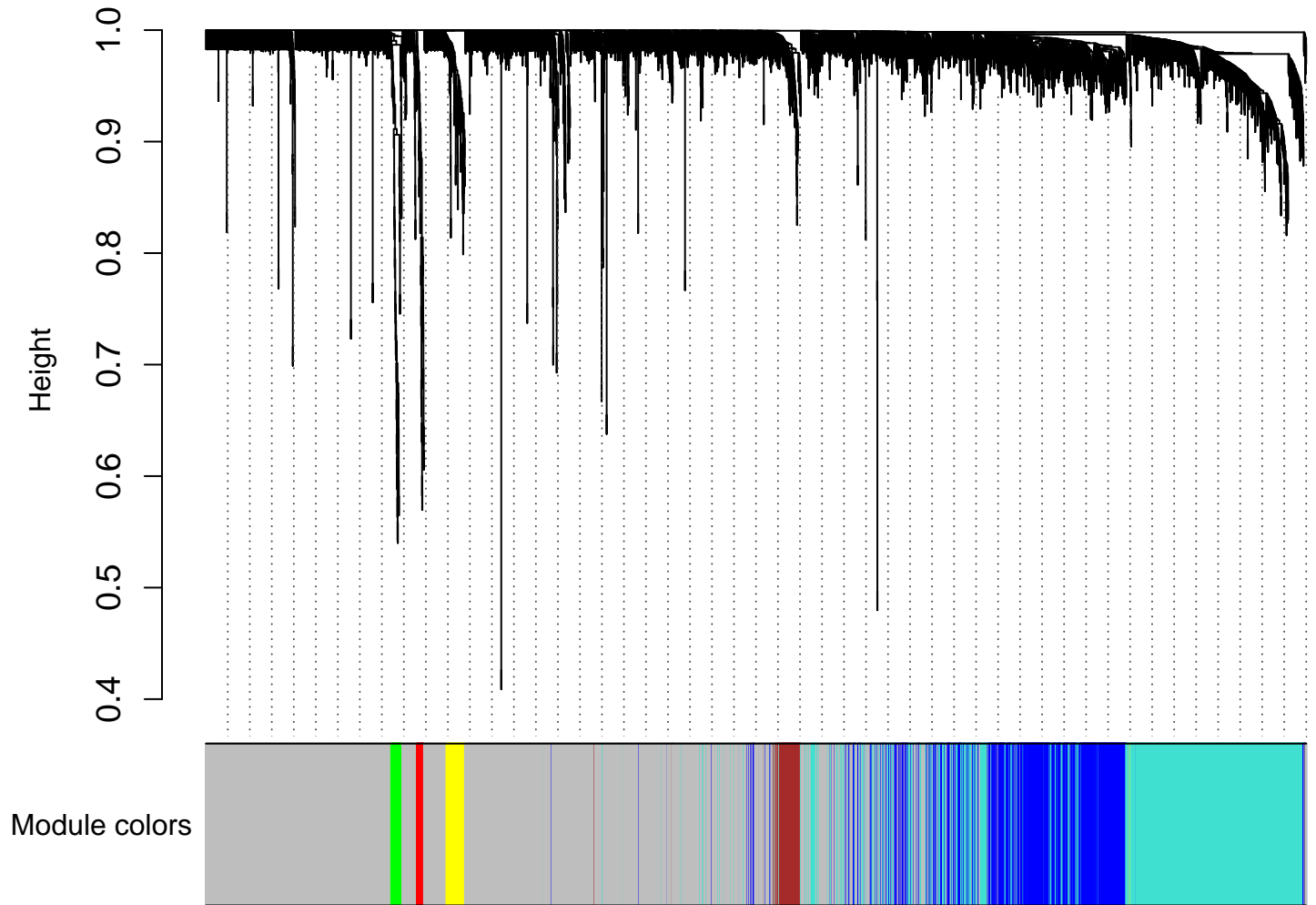

Supplement: Supplementary file 1 [file Supplementaryfile1.zip › Supplementary Material/02_WGCNA/3.1_WGCNA_OP_GSE56814/04_Cluster_dendrogram.pdf]

# Eigengene dendrogram

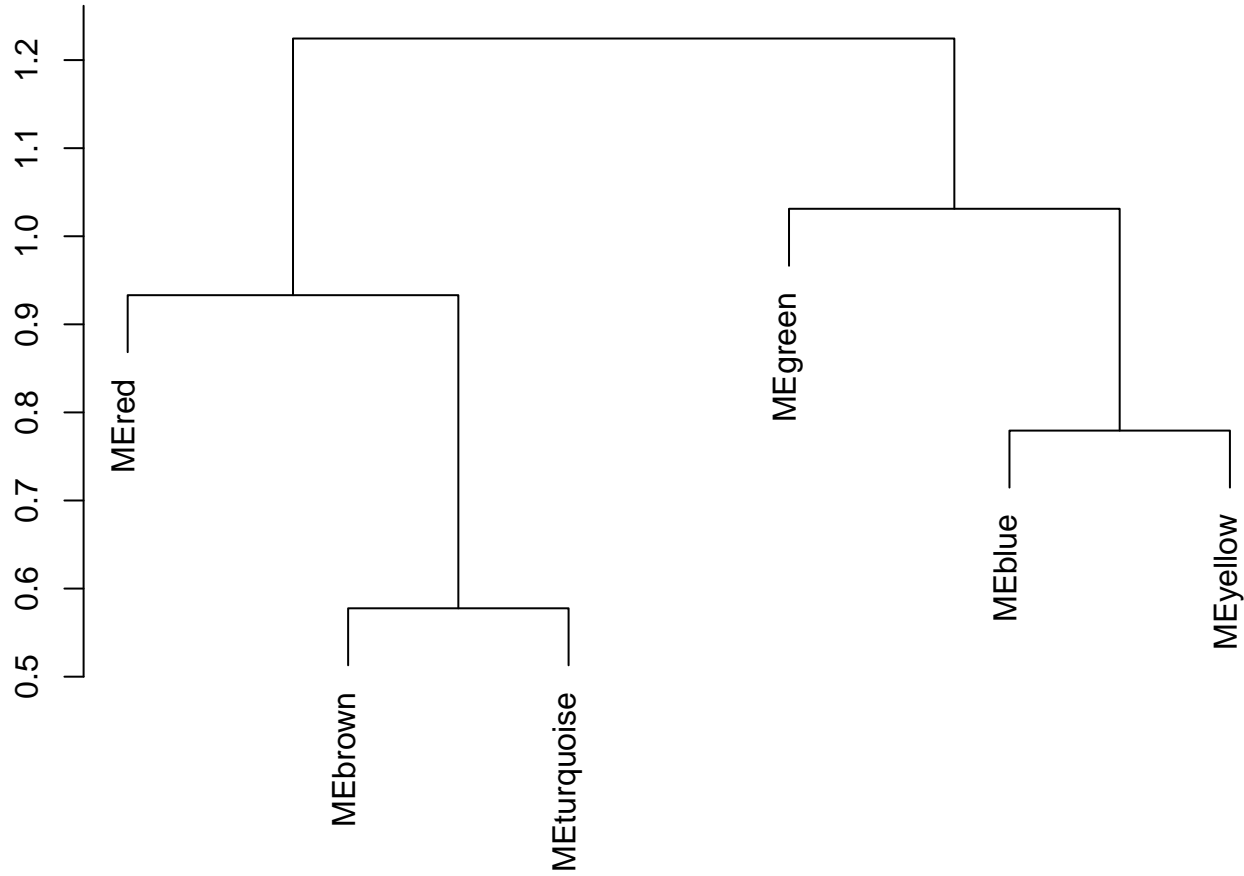

Supplement: Supplementary file 1 [file Supplementaryfile1.zip › Supplementary Material/02_WGCNA/3.1_WGCNA_OP_GSE56814/04_Eigengene dendrogram.pdf]

Module–trait relationships

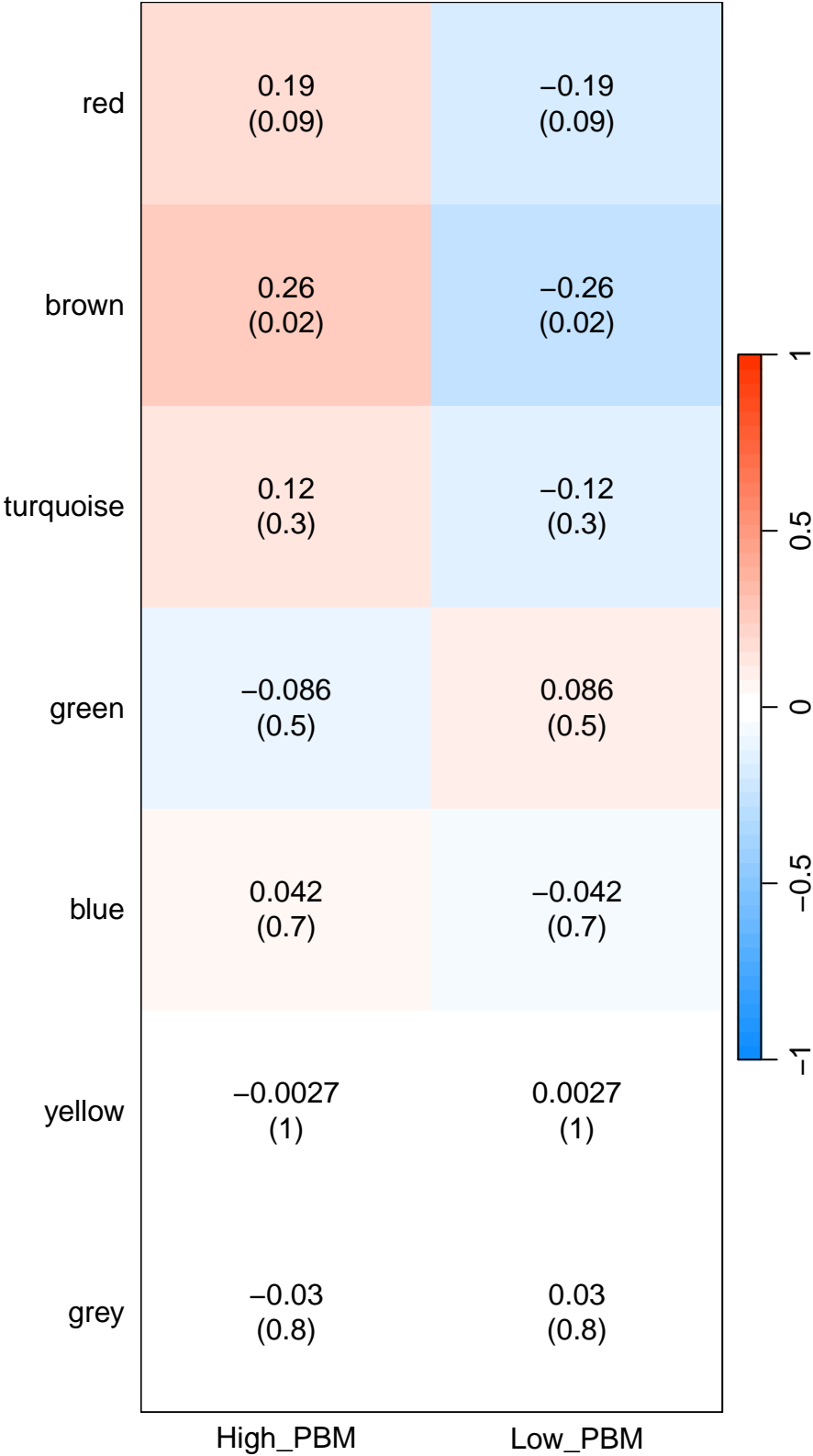

Supplement: Supplementary file 1 [file Supplementaryfile1.zip › Supplementary Material/02_WGCNA/3.1_WGCNA_OP_GSE56814/05_Module-trait_relationships.pdf]

## Sample clustering to detect outliers

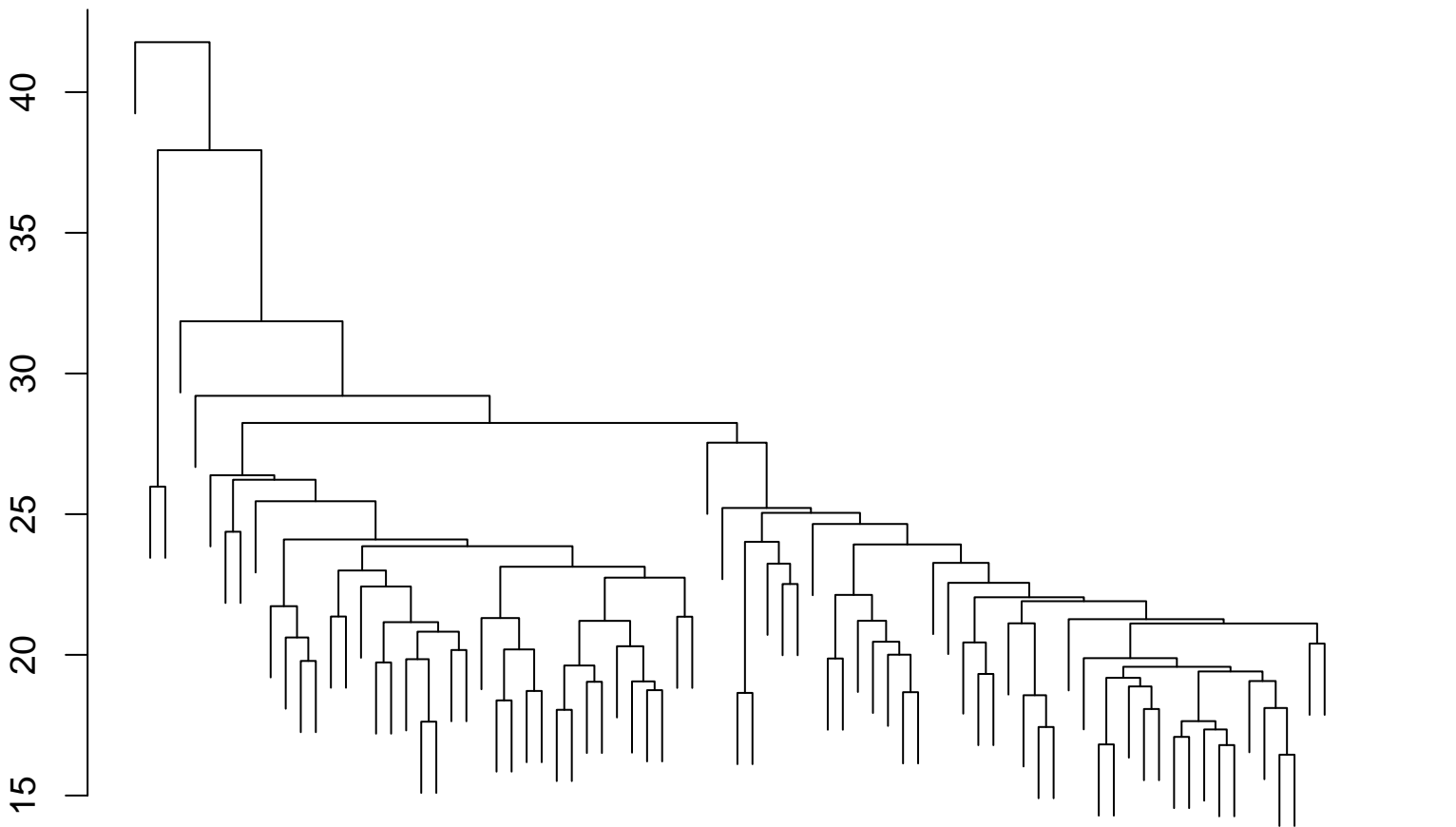

Supplement: Supplementary file 1 [file Supplementaryfile1.zip › Supplementary Material/02_WGCNA/4.1_WGCNA_OP_GSE56815/00_Clustering_Detect_outlier.pdf]

# Sample clustering to detect outliers

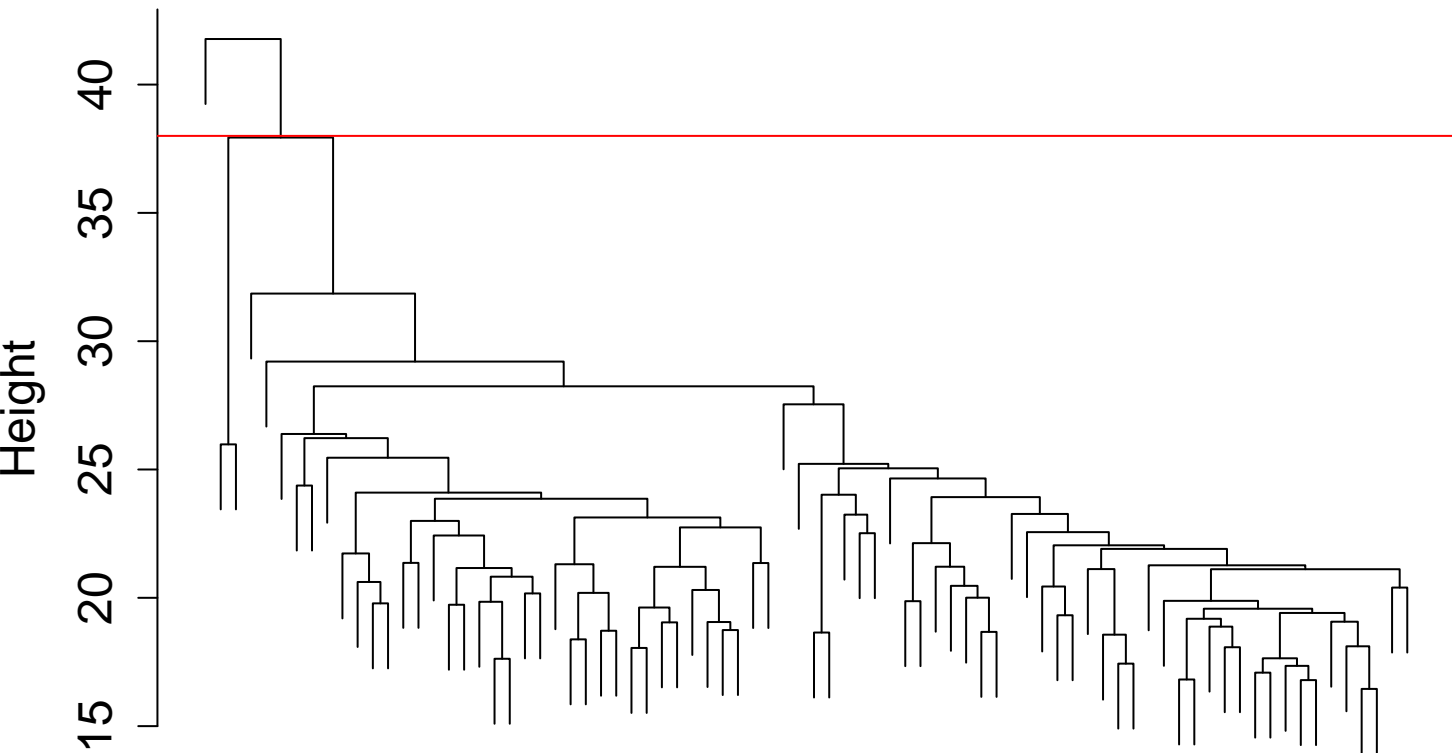

Supplement: Supplementary file 1 [file Supplementaryfile1.zip › Supplementary Material/02_WGCNA/4.1_WGCNA_OP_GSE56815/01_Clustering_show_outlier.pdf]

## Mean connectivity

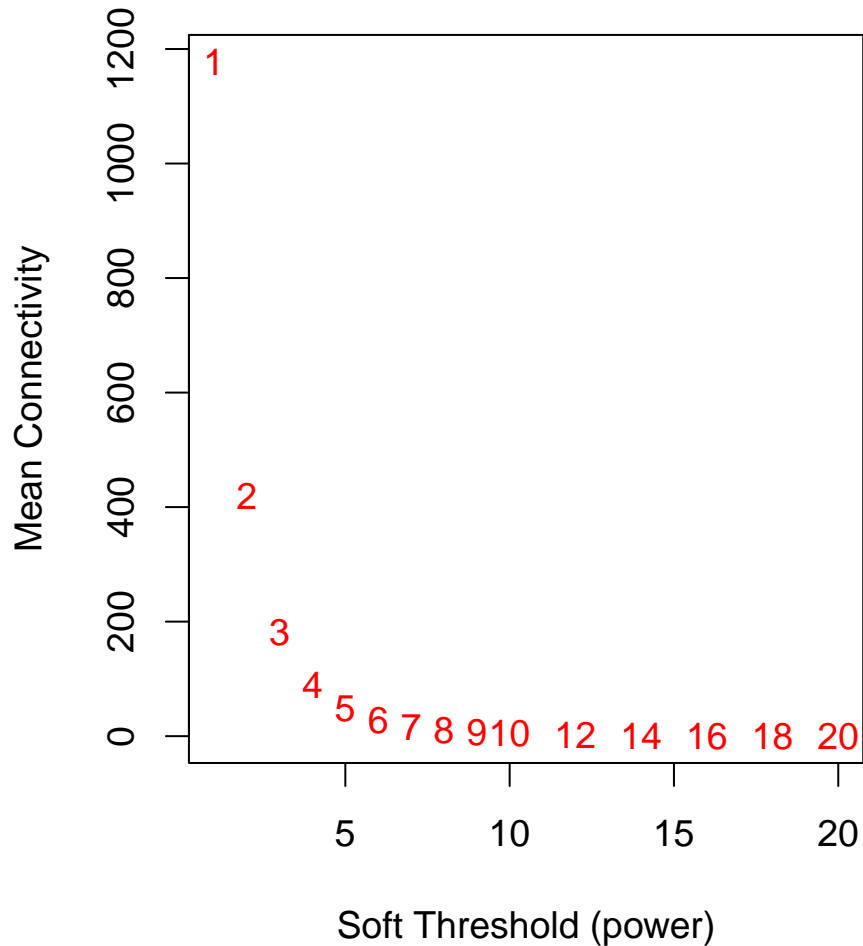

Supplement: Supplementary file 1 [file Supplementaryfile1.zip › Supplementary Material/02_WGCNA/4.1_WGCNA_OP_GSE56815/03_Mean_Connectivity.pdf]

## Scale independence

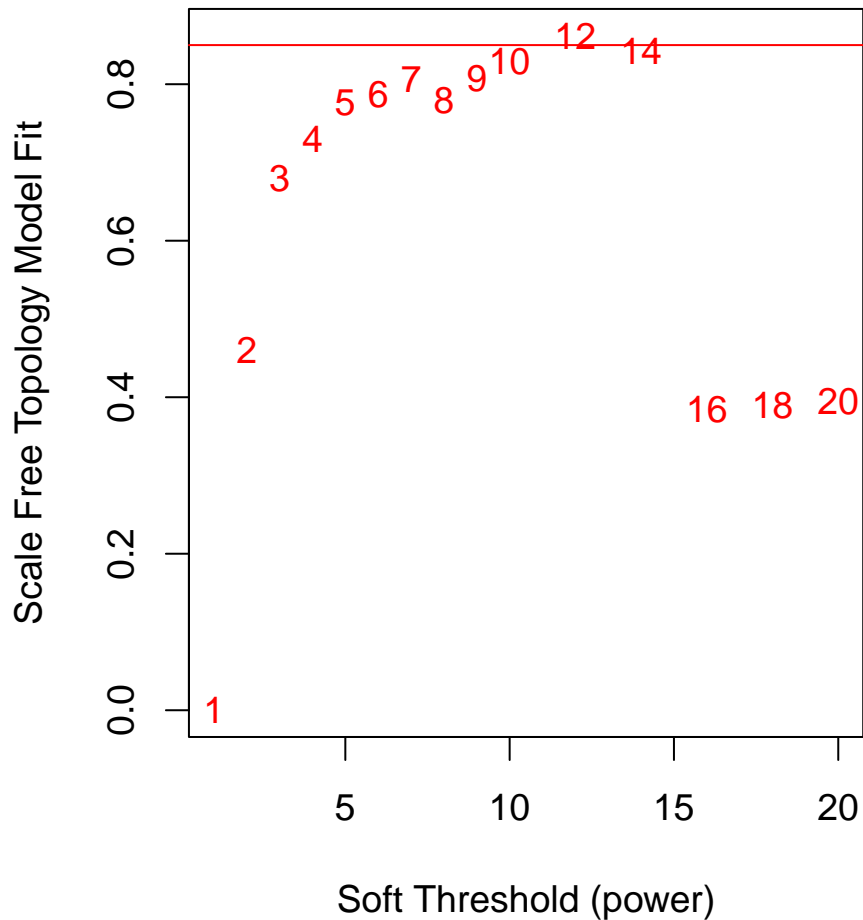

Supplement: Supplementary file 1 [file Supplementaryfile1.zip › Supplementary Material/02_WGCNA/4.1_WGCNA_OP_GSE56815/03_Scale_independence.pdf]

# Cluster Dendrogram

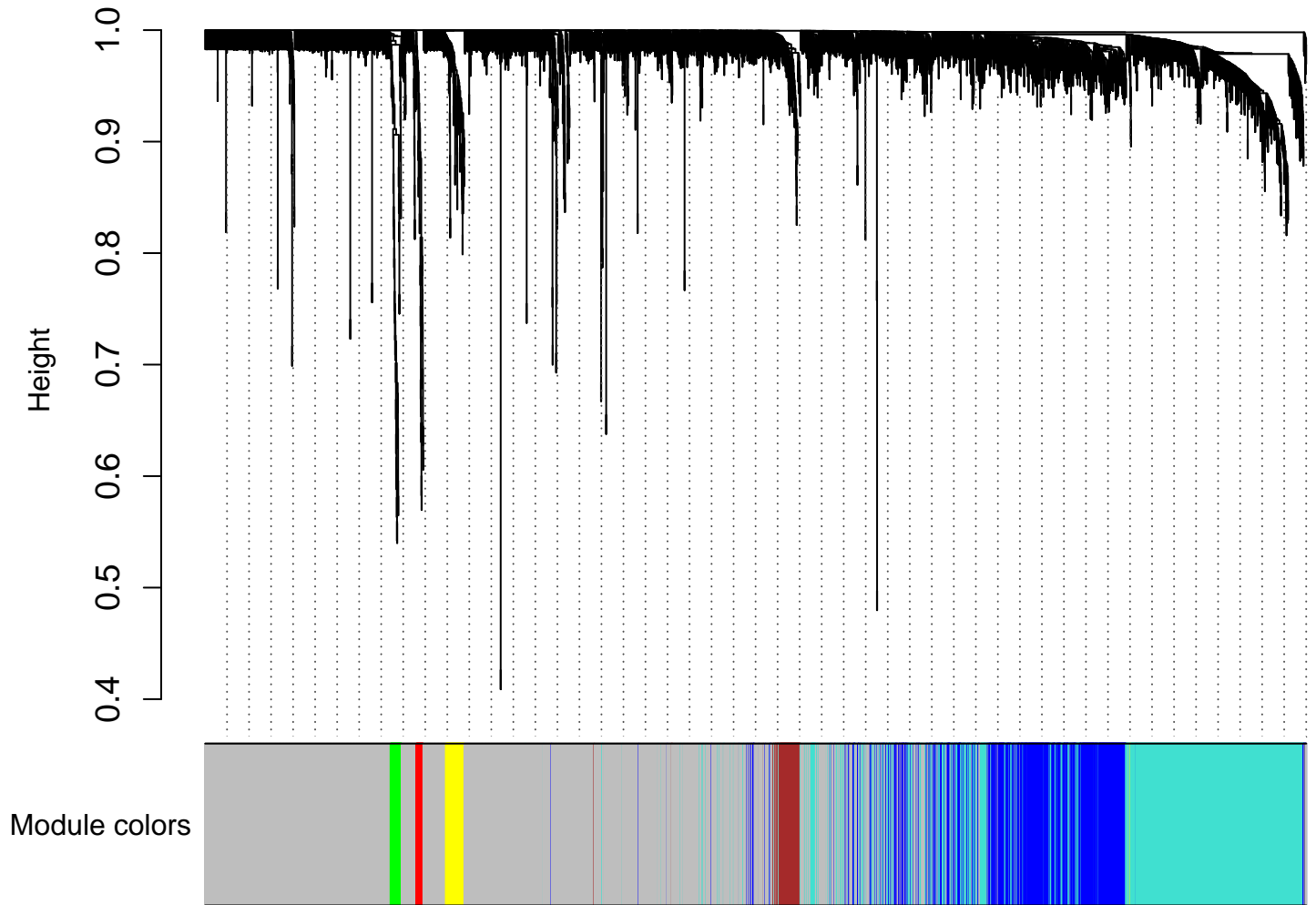

Supplement: Supplementary file 1 [file Supplementaryfile1.zip › Supplementary Material/02_WGCNA/4.1_WGCNA_OP_GSE56815/04_Cluster_dendrogram.pdf]

# Eigengene dendrogram

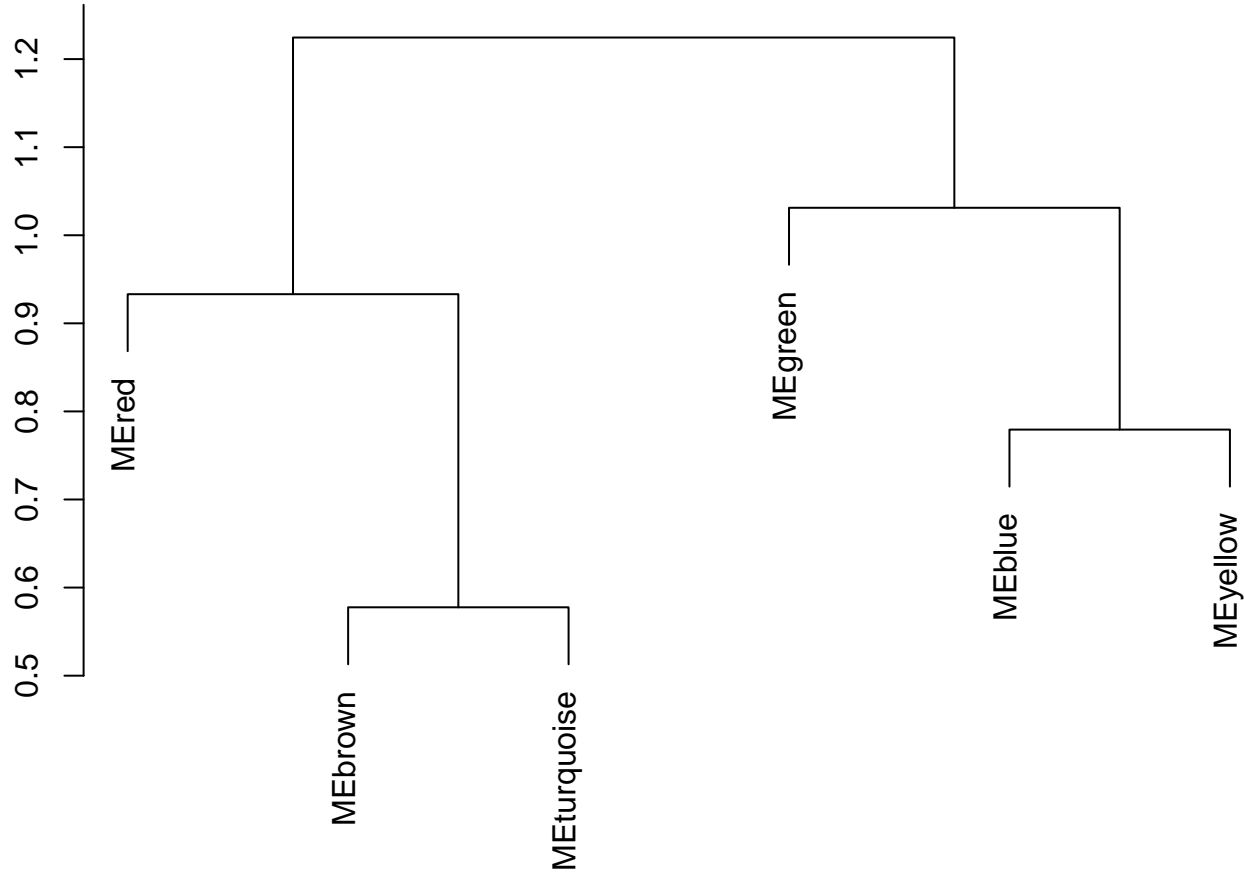

Supplement: Supplementary file 1 [file Supplementaryfile1.zip › Supplementary Material/02_WGCNA/4.1_WGCNA_OP_GSE56815/04_Eigengene dendrogram.pdf]

Module–trait relationships

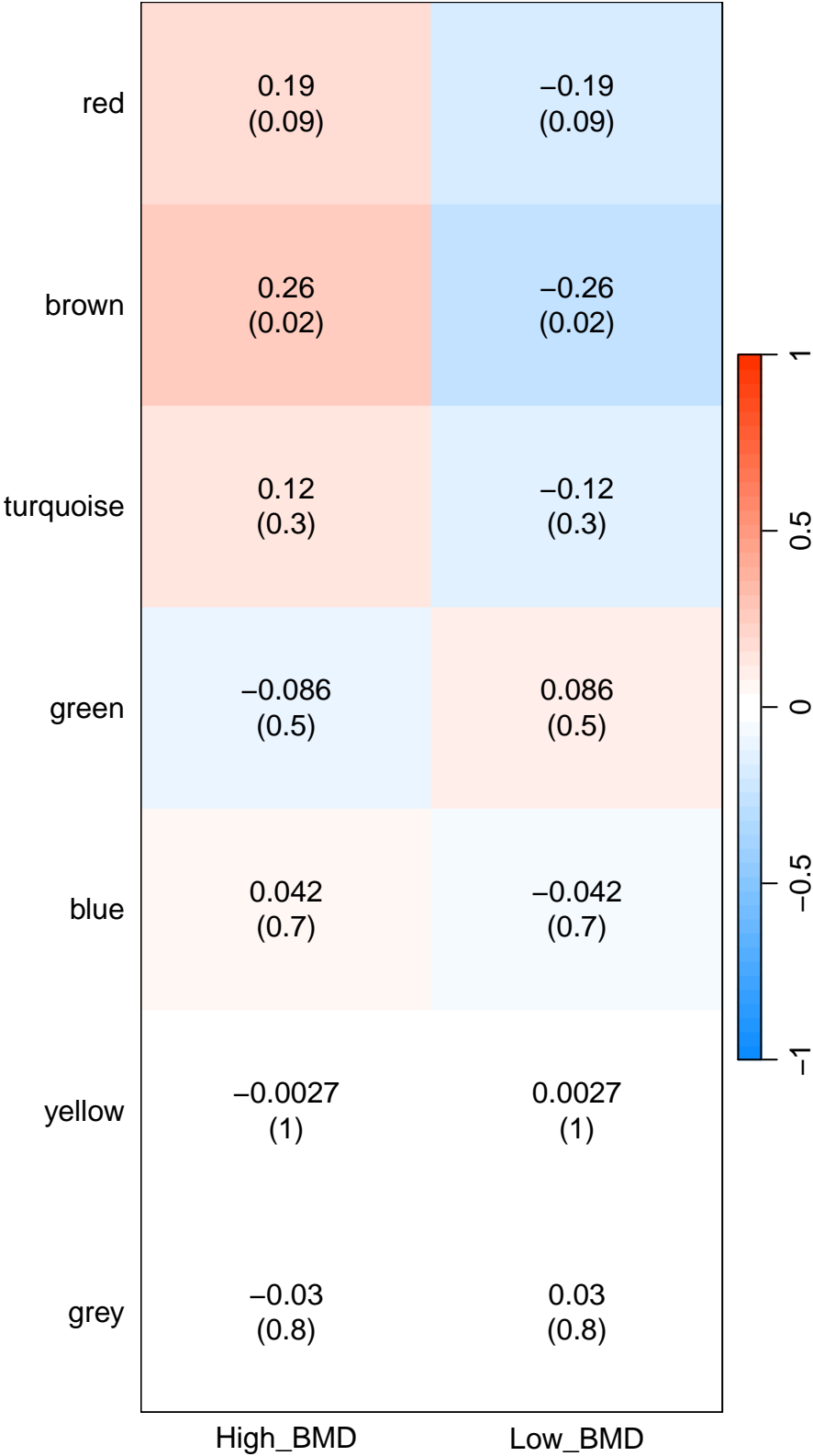

Supplement: Supplementary file 1 [file Supplementaryfile1.zip › Supplementary Material/02_WGCNA/4.1_WGCNA_OP_GSE56815/05_Module-trait_relationships.pdf]

## Sample clustering to detect outliers

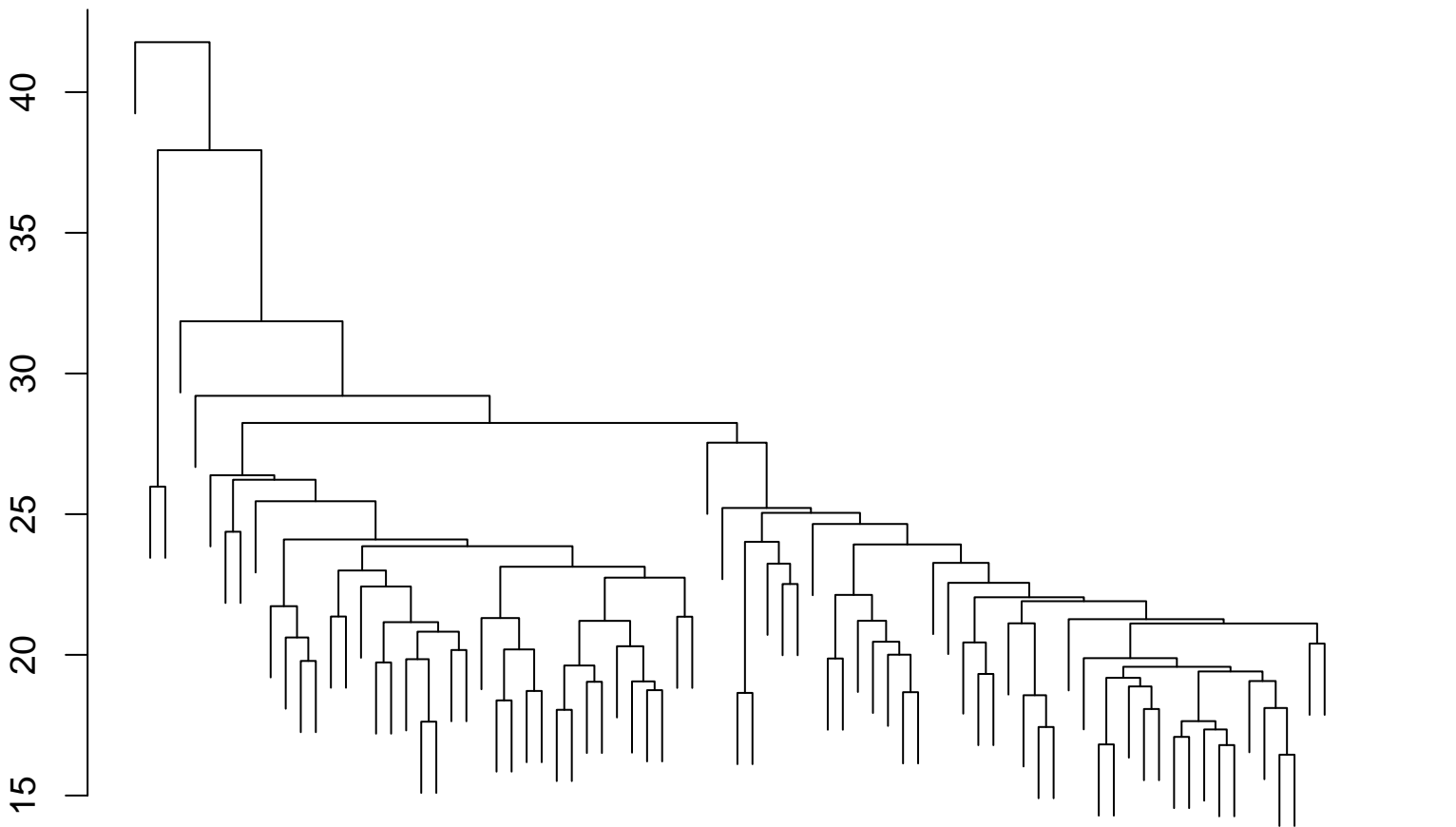

Supplement: Supplementary file 1 [file Supplementaryfile1.zip › Supplementary Material/02_WGCNA/4.1_WGCNA_OP_GSE56815_bp/00_Clustering_Detect_outlier.pdf]

# Sample clustering to detect outliers

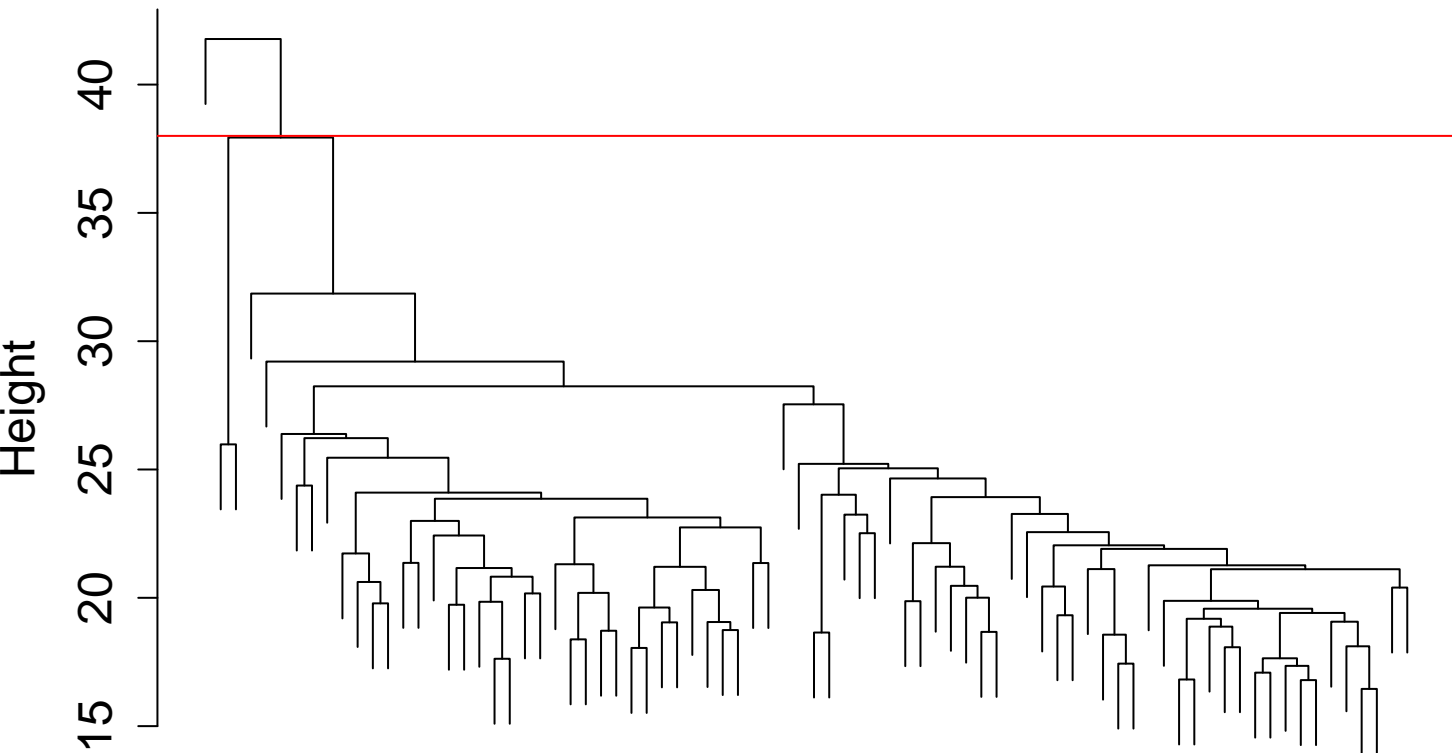

Supplement: Supplementary file 1 [file Supplementaryfile1.zip › Supplementary Material/02_WGCNA/4.1_WGCNA_OP_GSE56815_bp/01_Clustering_show_outlier.pdf]

## Sample dendrogram and trait heatmap

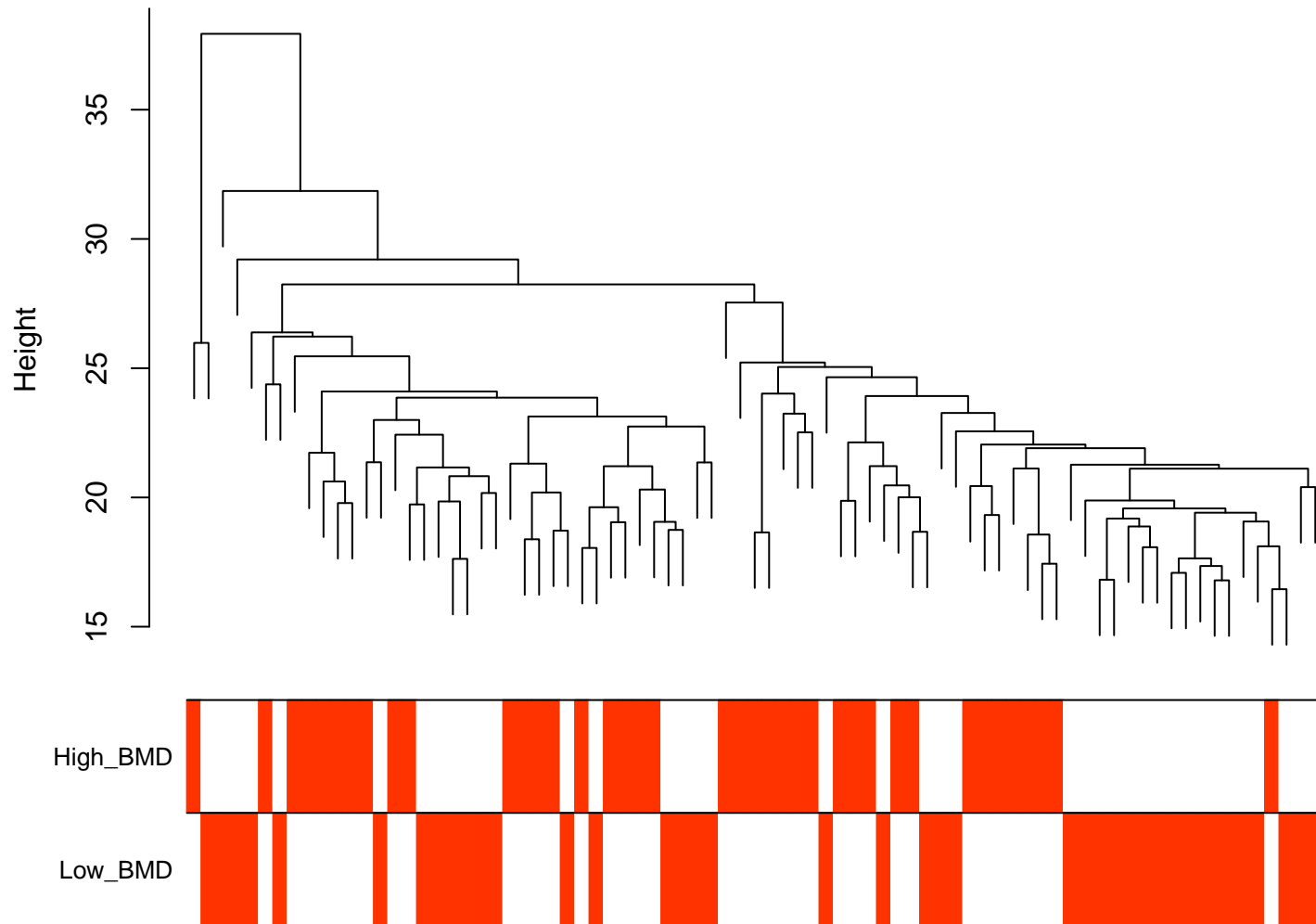

Supplement: Supplementary file 1 [file Supplementaryfile1.zip › Supplementary Material/02_WGCNA/4.1_WGCNA_OP_GSE56815_bp/02_Clustering_with_pheno.pdf]

# Mean connectivity

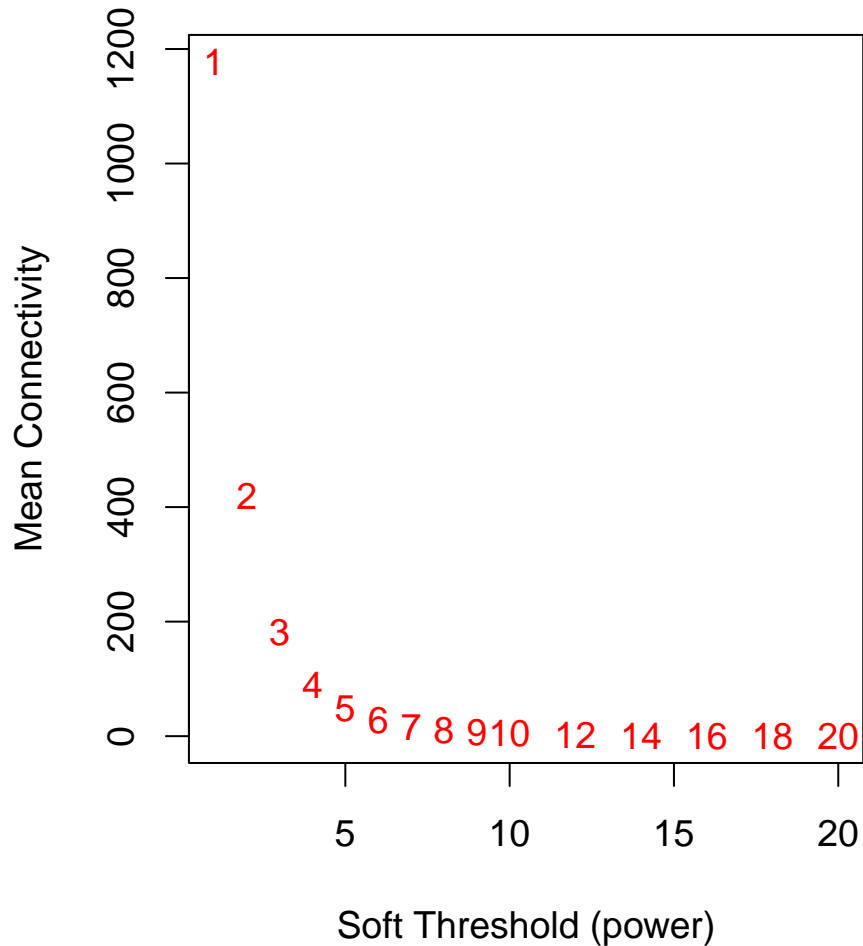

Supplement: Supplementary file 1 [file Supplementaryfile1.zip › Supplementary Material/02_WGCNA/4.1_WGCNA_OP_GSE56815_bp/03_Mean_Connectivity.pdf]

## Scale independence

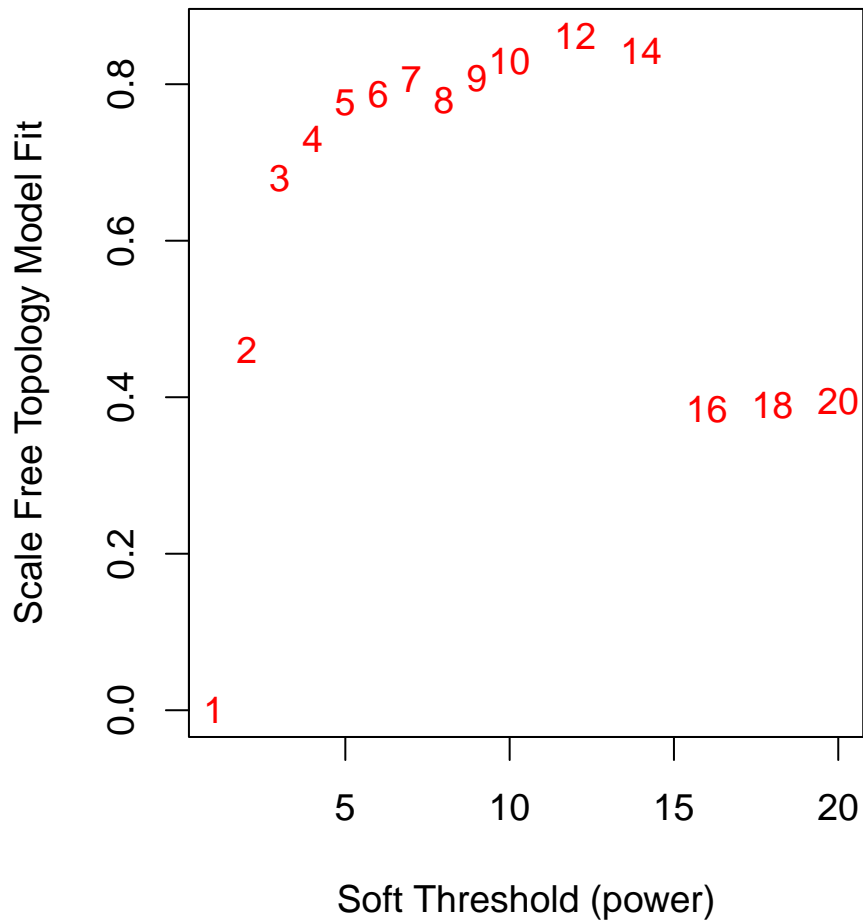

Supplement: Supplementary file 1 [file Supplementaryfile1.zip › Supplementary Material/02_WGCNA/4.1_WGCNA_OP_GSE56815_bp/03_Scale_independence.pdf]

# Eigengene dendrogram

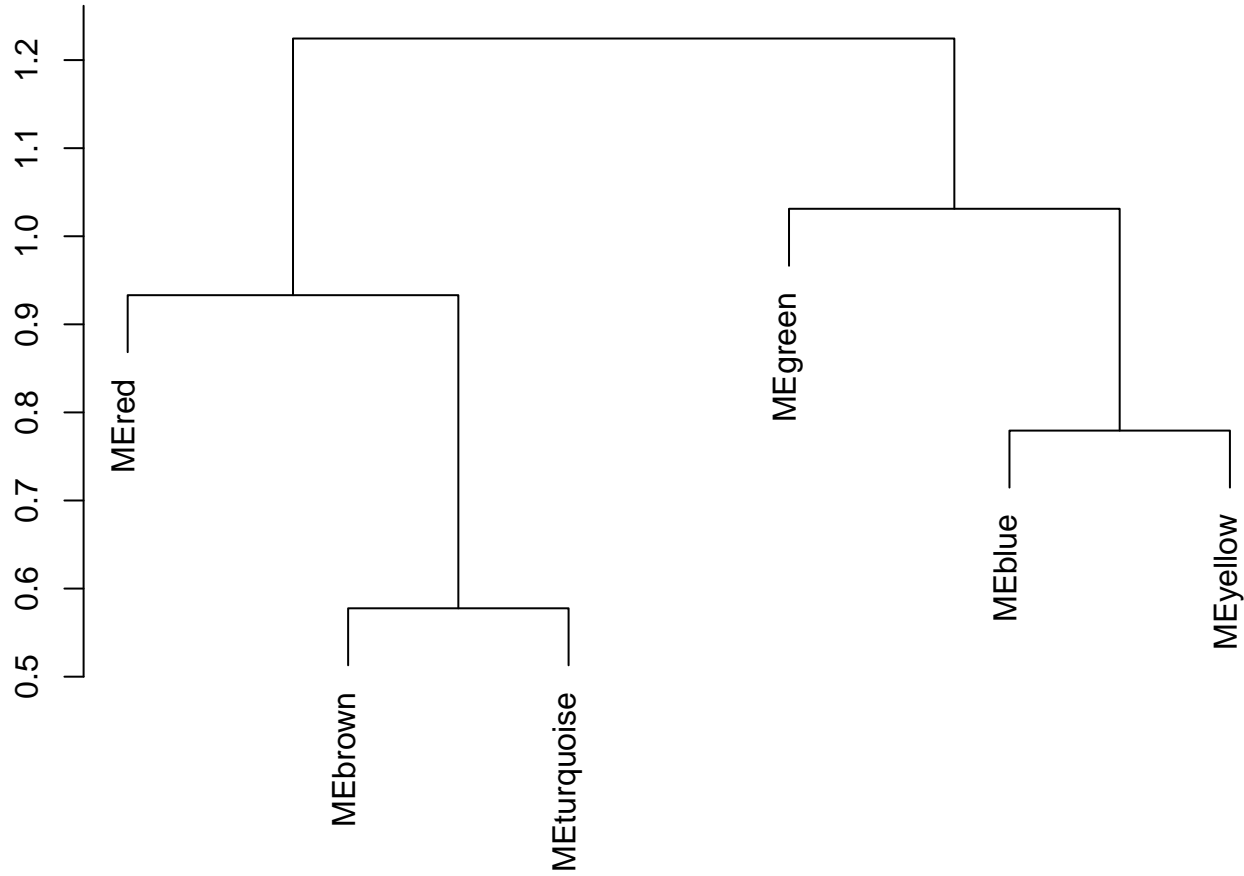

Supplement: Supplementary file 1 [file Supplementaryfile1.zip › Supplementary Material/02_WGCNA/4.1_WGCNA_OP_GSE56815_bp/04_Eigengene dendrogram.pdf]

Module–trait relationships

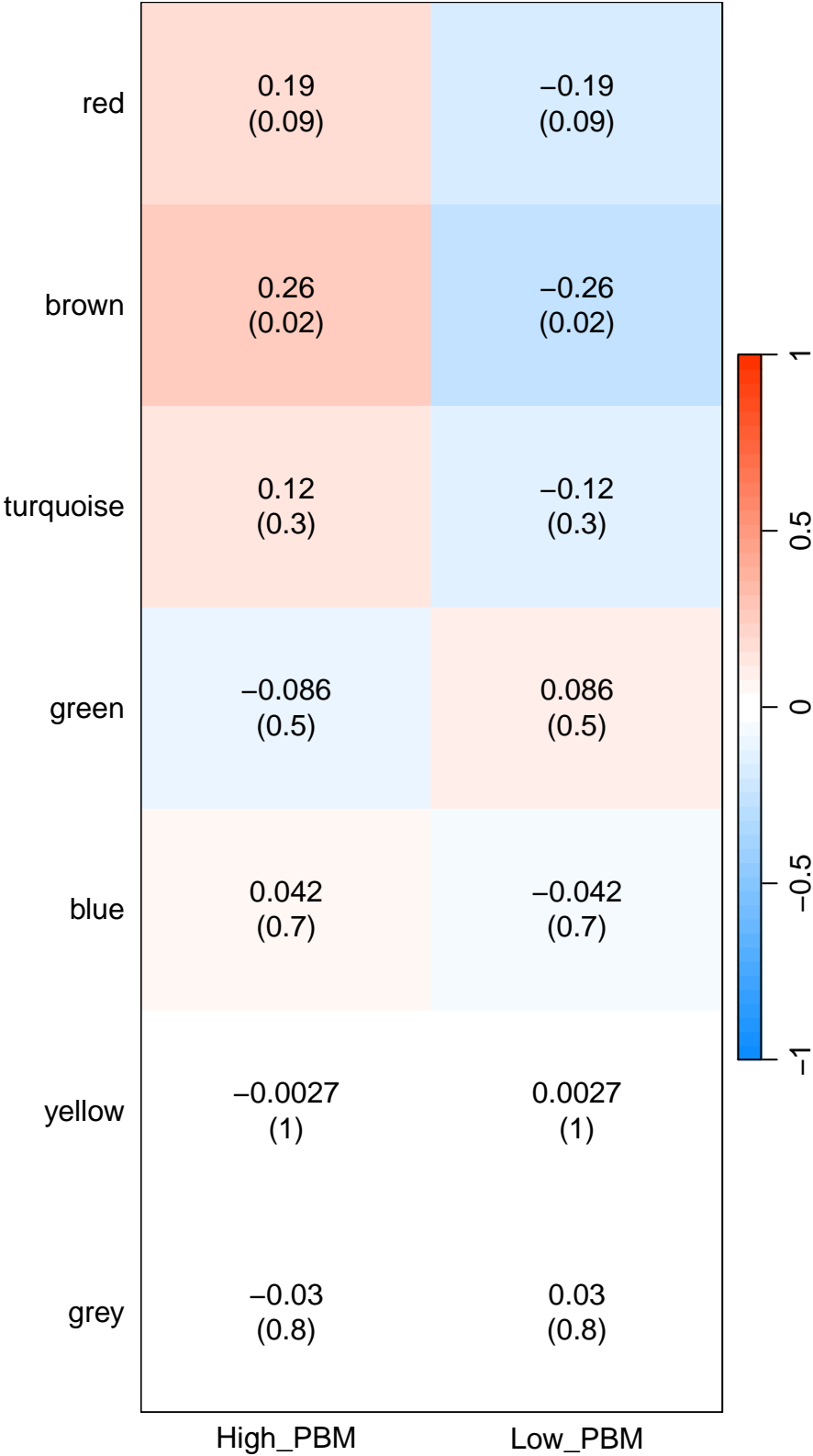

Supplement: Supplementary file 1 [file Supplementaryfile1.zip › Supplementary Material/02_WGCNA/4.1_WGCNA_OP_GSE56815_bp/05_Module-trait_relationships.pdf]

WGCNA\_CD

WGCNA\_OP

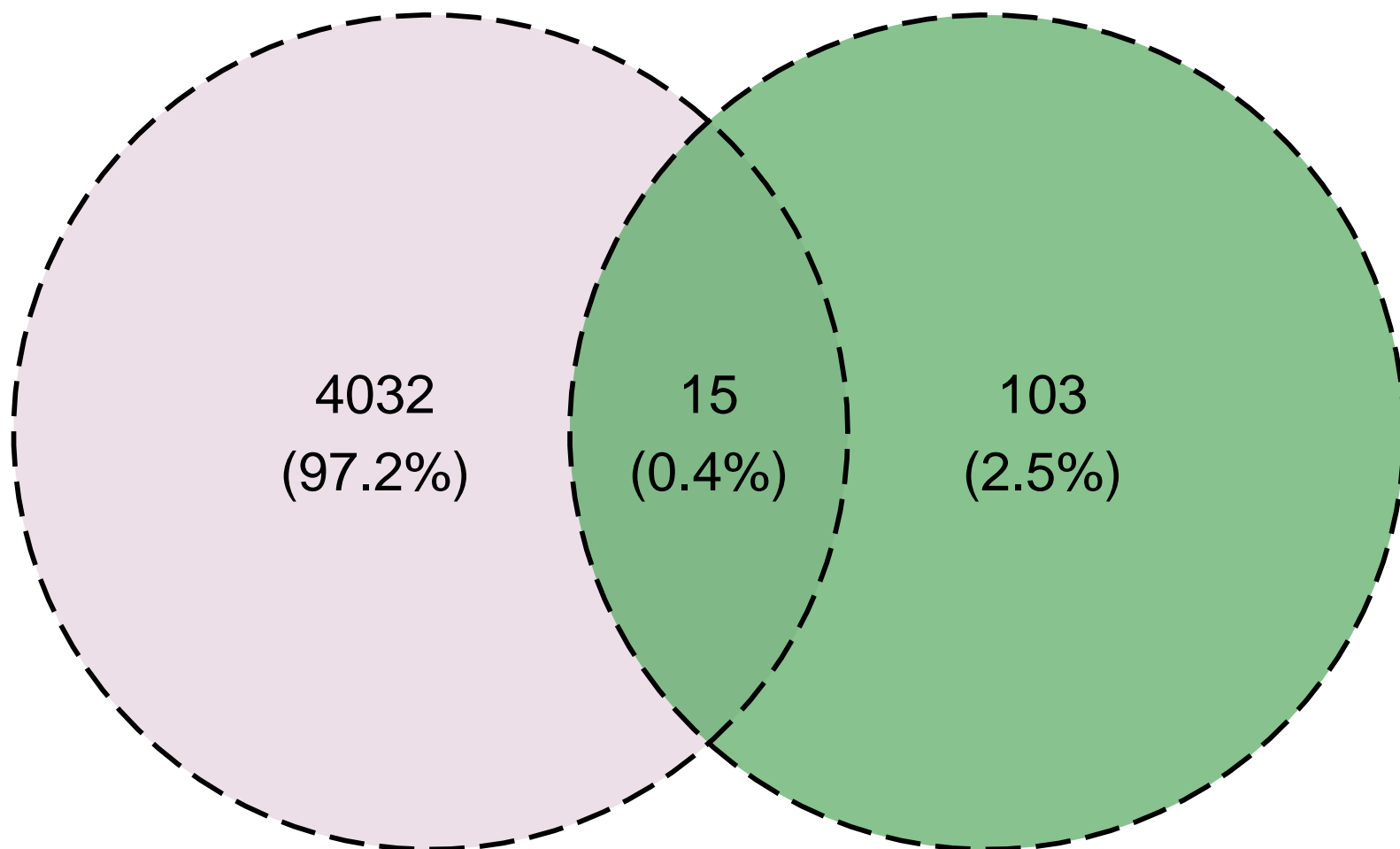

Supplement: Supplementary file 1 [file Supplementaryfile1.zip › Supplementary Material/02_WGCNA/5.1_WGCNA_venn.pdf]

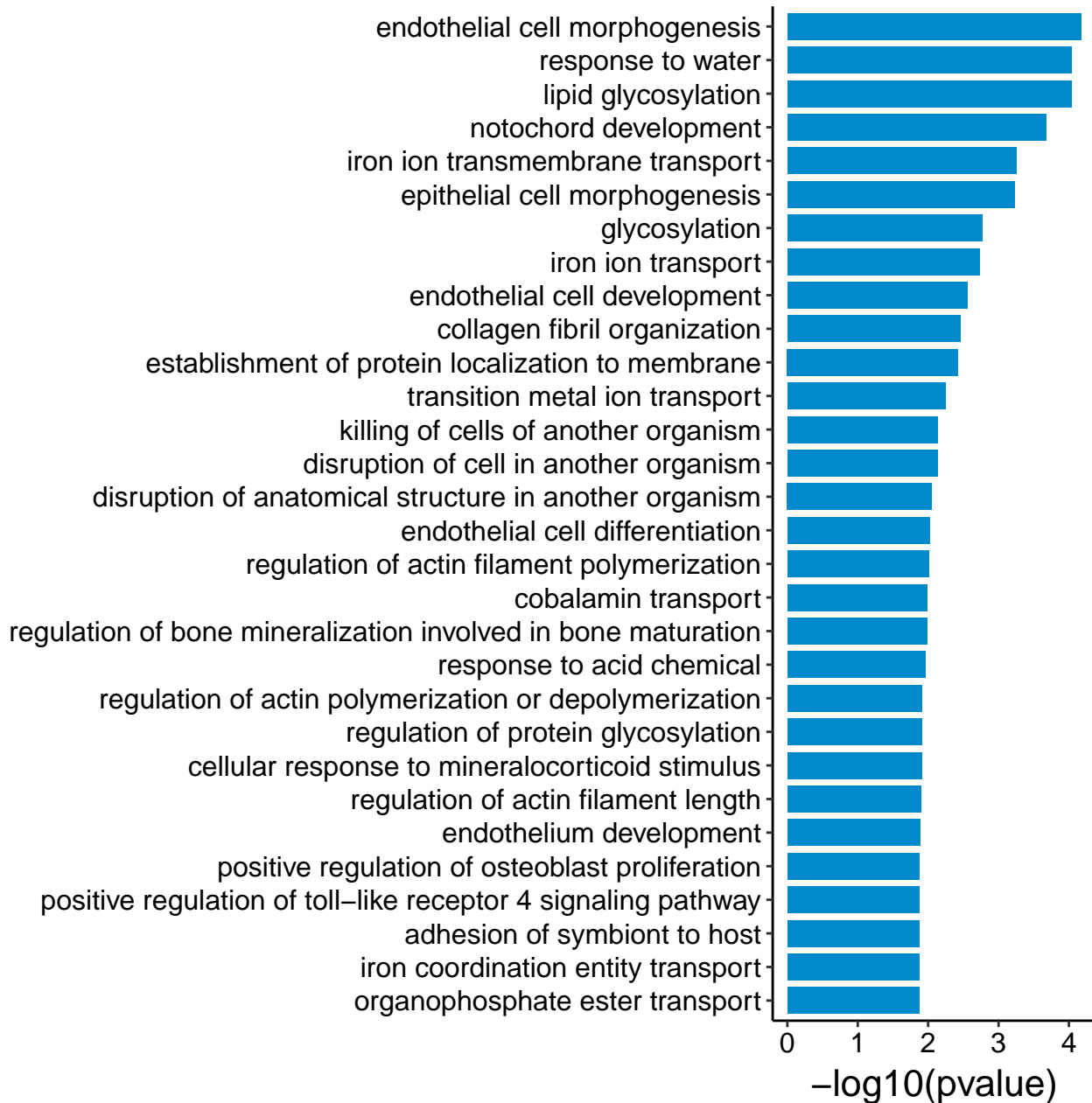

Supplement: Supplementary file 1 [file Supplementaryfile1.zip › Supplementary Material/02_WGCNA/6.2_key_genes_GO.pdf]

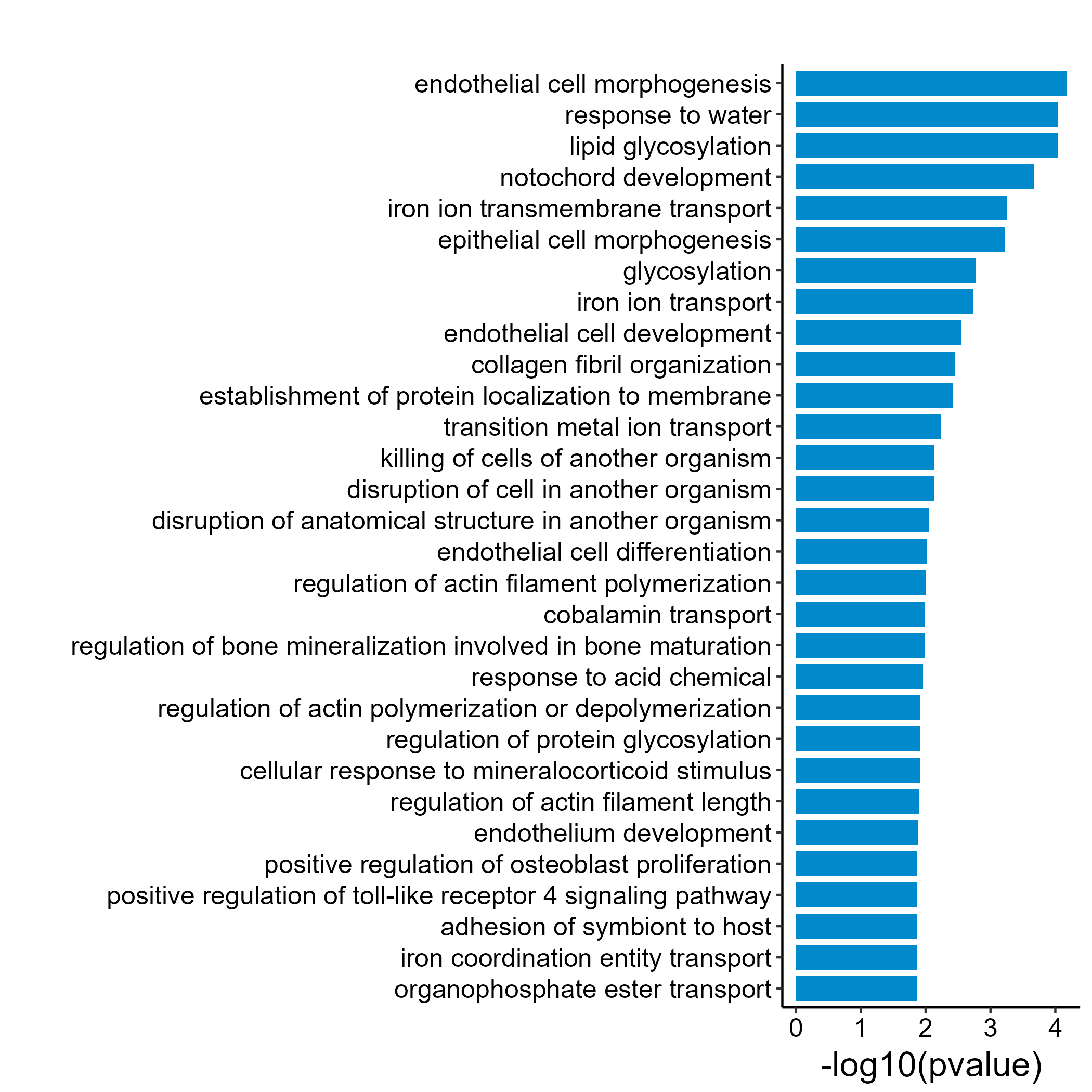

Supplement: Supplementary file 1 [file Supplementaryfile1.zip › Supplementary Material/02_WGCNA/6.2_key_genes_GO.png]

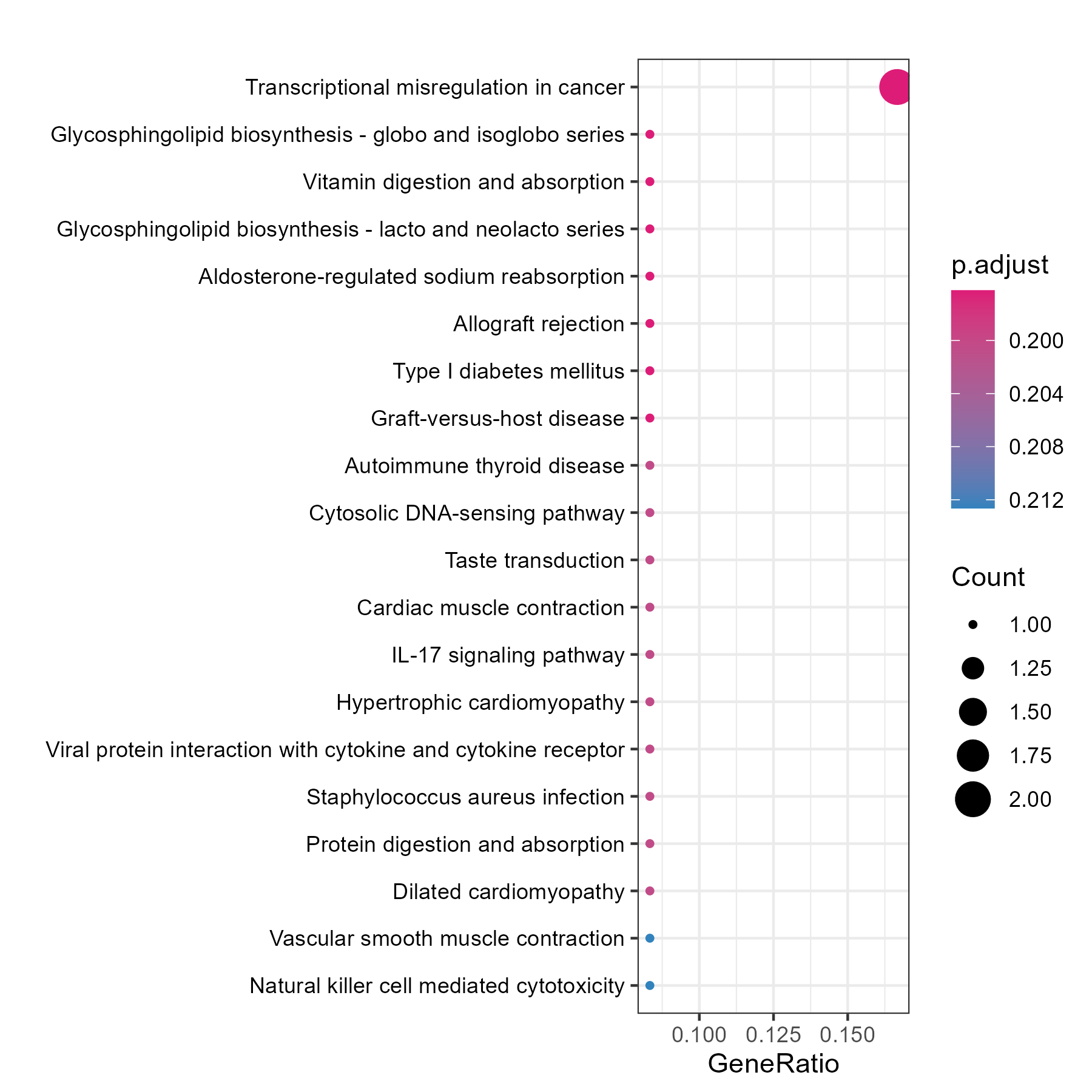

Supplement: Supplementary file 1 [file Supplementaryfile1.zip › Supplementary Material/02_WGCNA/6.2_key_genes_KEGG.png]

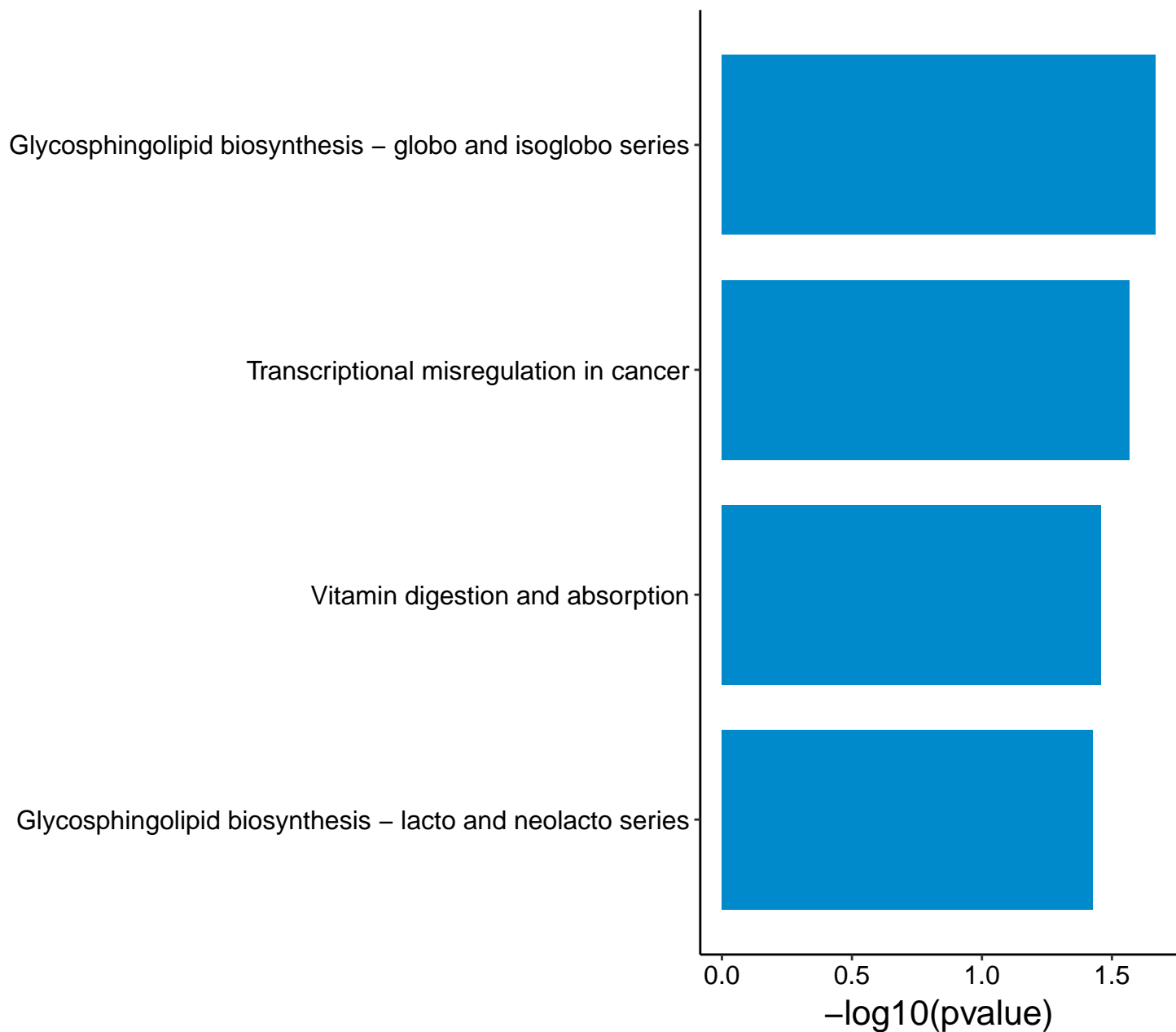

Supplement: Supplementary file 1 [file Supplementaryfile1.zip › Supplementary Material/02_WGCNA/6.3_key_genes_KEGG.pdf]

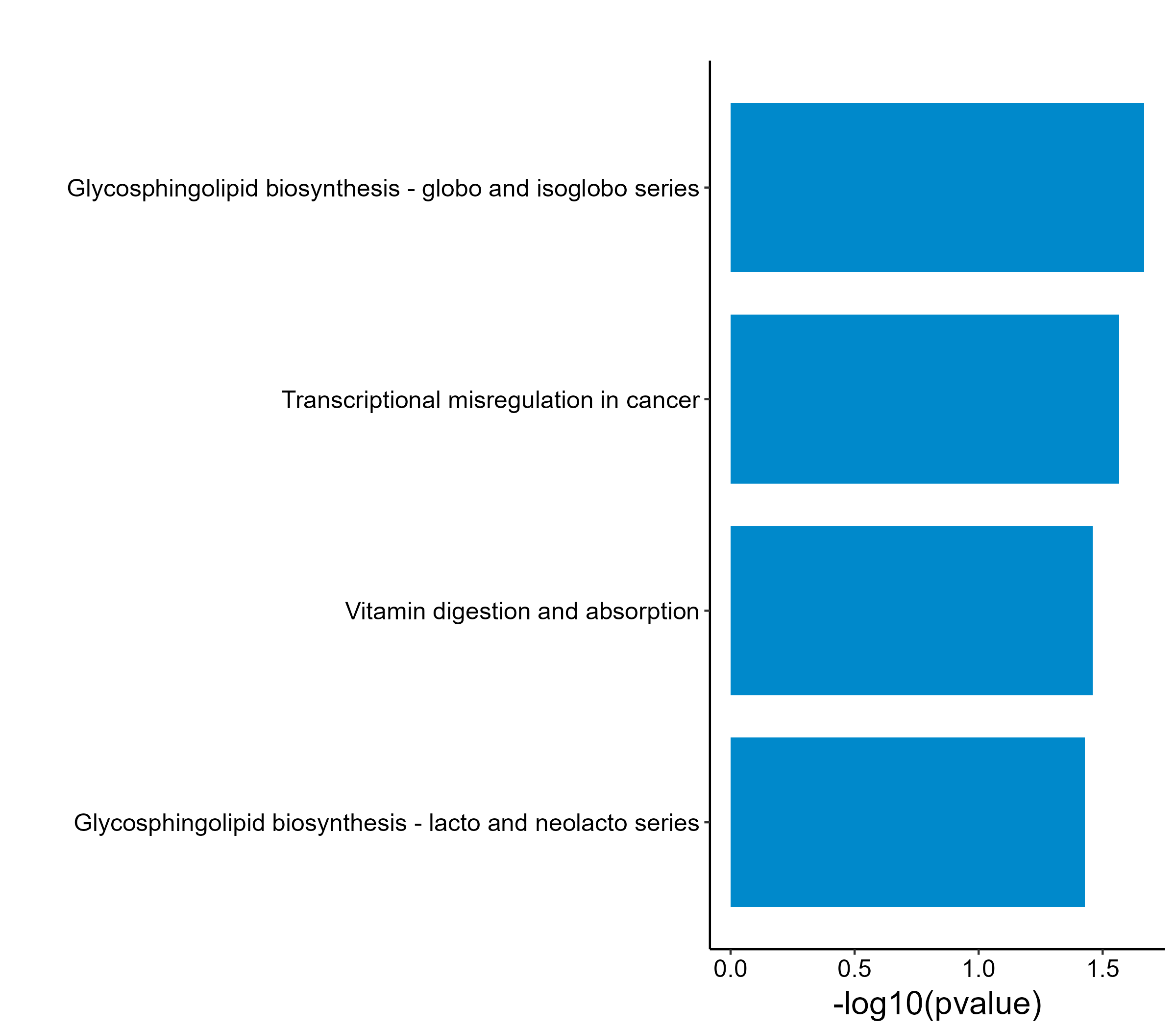

Supplement: Supplementary file 1 [file Supplementaryfile1.zip › Supplementary Material/02_WGCNA/6.3_key_genes_KEGG.png]

Coefficients

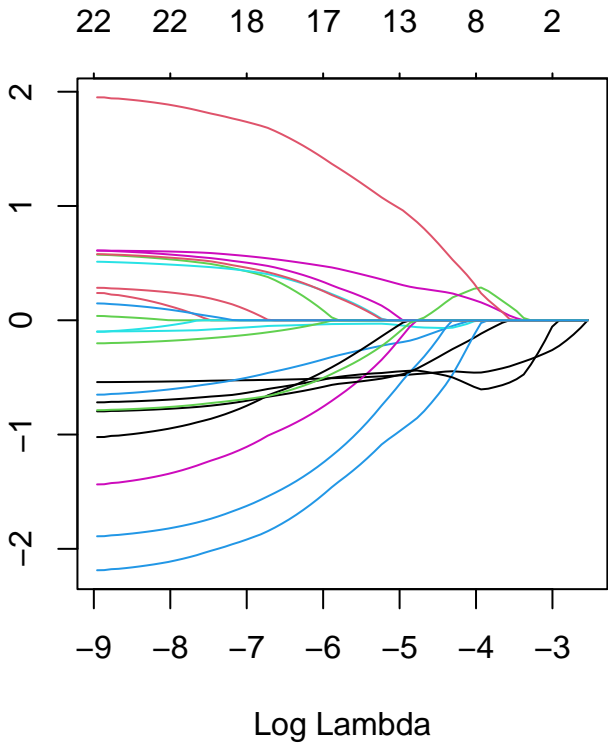

Supplement: Supplementary file 1 [file Supplementaryfile1.zip › Supplementary Material/03_ML/1.1_CD_LASSSO.lambda.pdf]

Binomial Deviance

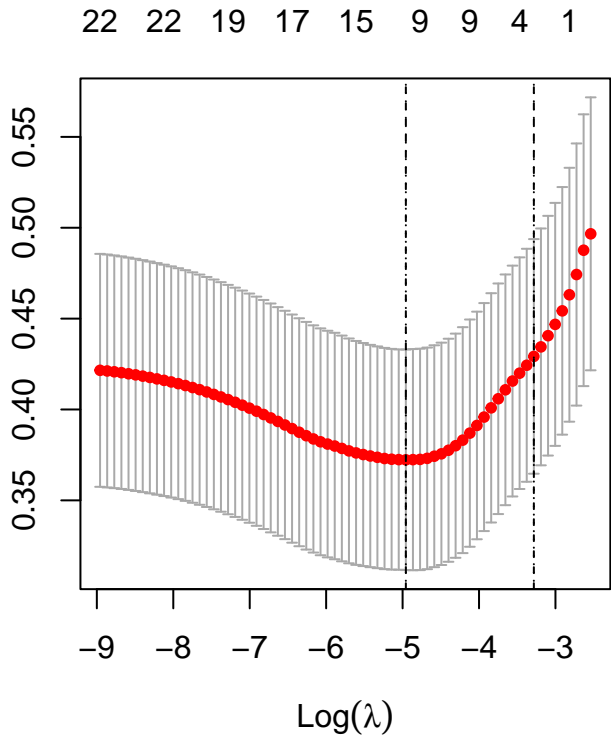

Supplement: Supplementary file 1 [file Supplementaryfile1.zip › Supplementary Material/03_ML/1.2_CD_LASSO.cvfit.pdf]

# Variable importance

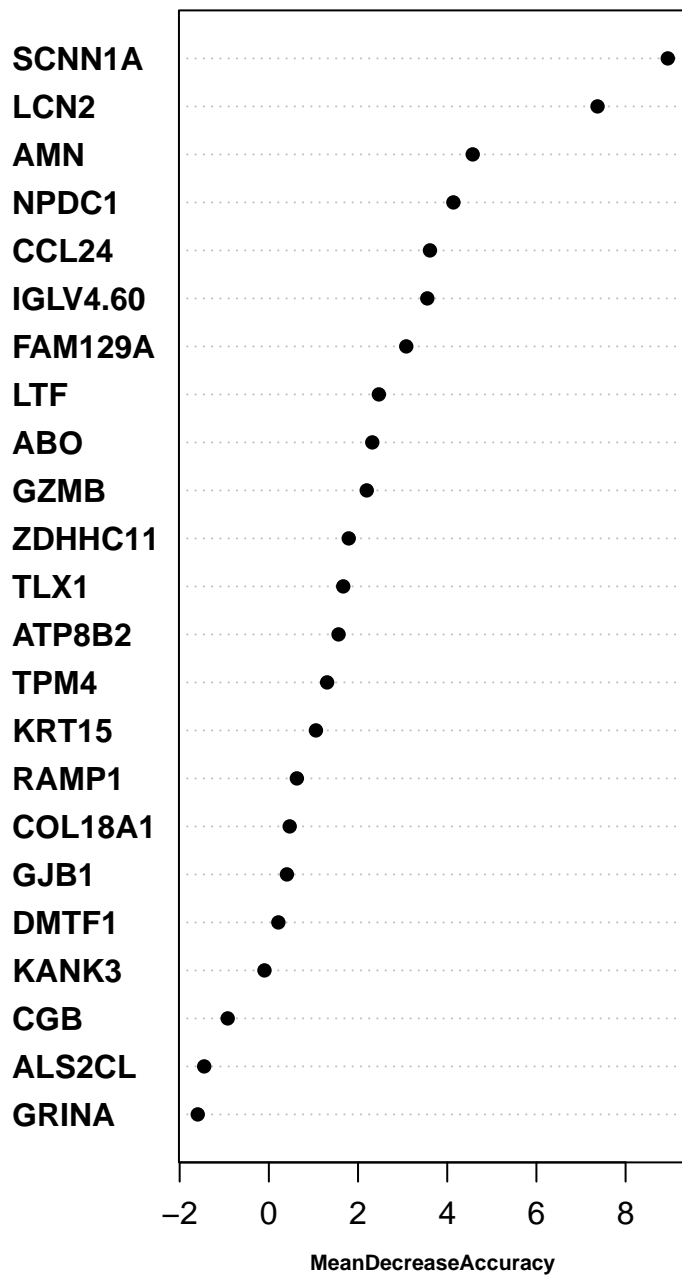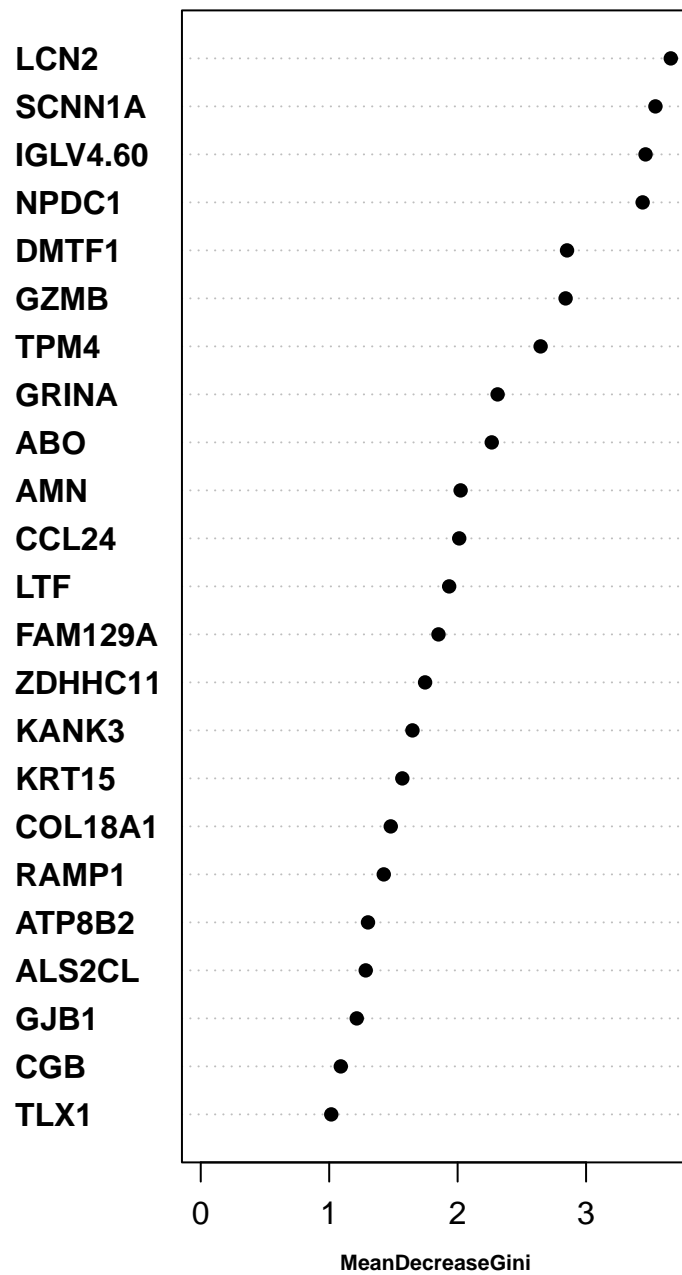

Supplement: Supplementary file 1 [file Supplementaryfile1.zip › Supplementary Material/03_ML/1.4_CD_RF_Variable_importance.pdf]

Coefficients

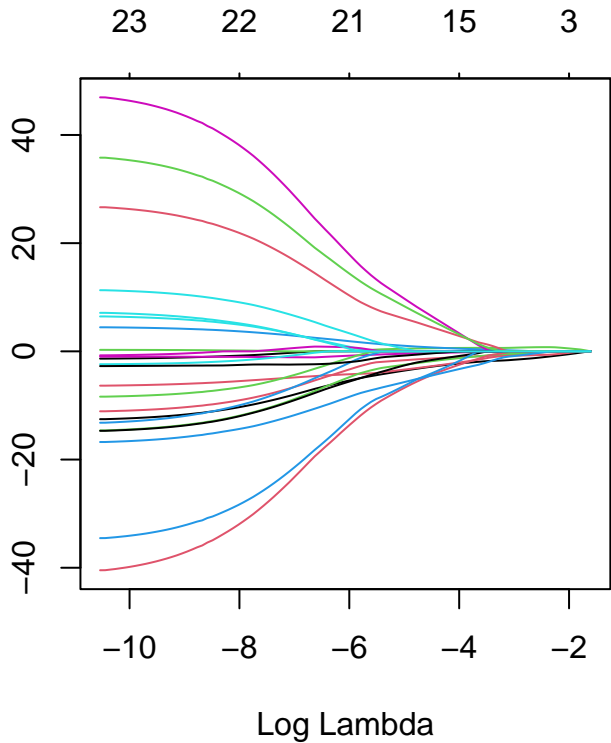

Supplement: Supplementary file 1 [file Supplementaryfile1.zip › Supplementary Material/03_ML/2.1_OP_LASSSO.lambda.pdf]

Binomial Deviance

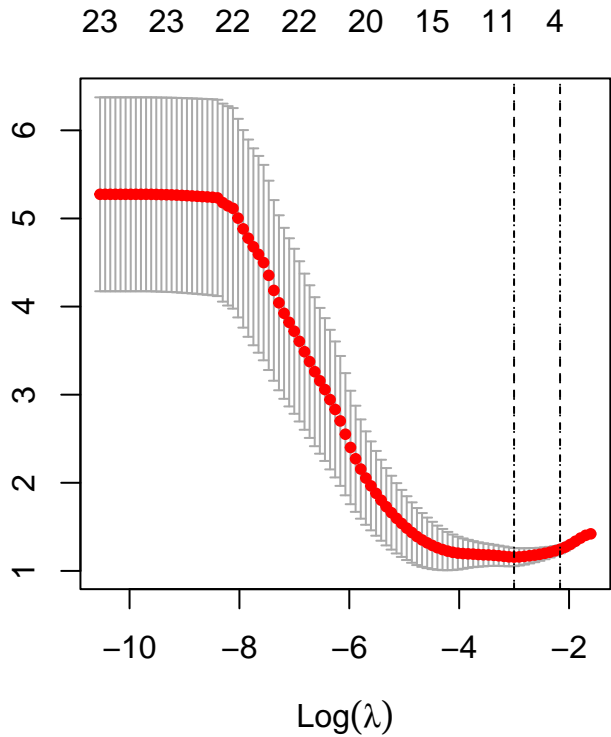

Supplement: Supplementary file 1 [file Supplementaryfile1.zip › Supplementary Material/03_ML/2.2_OP_LASSO.cvfit.pdf]

# Variable importance

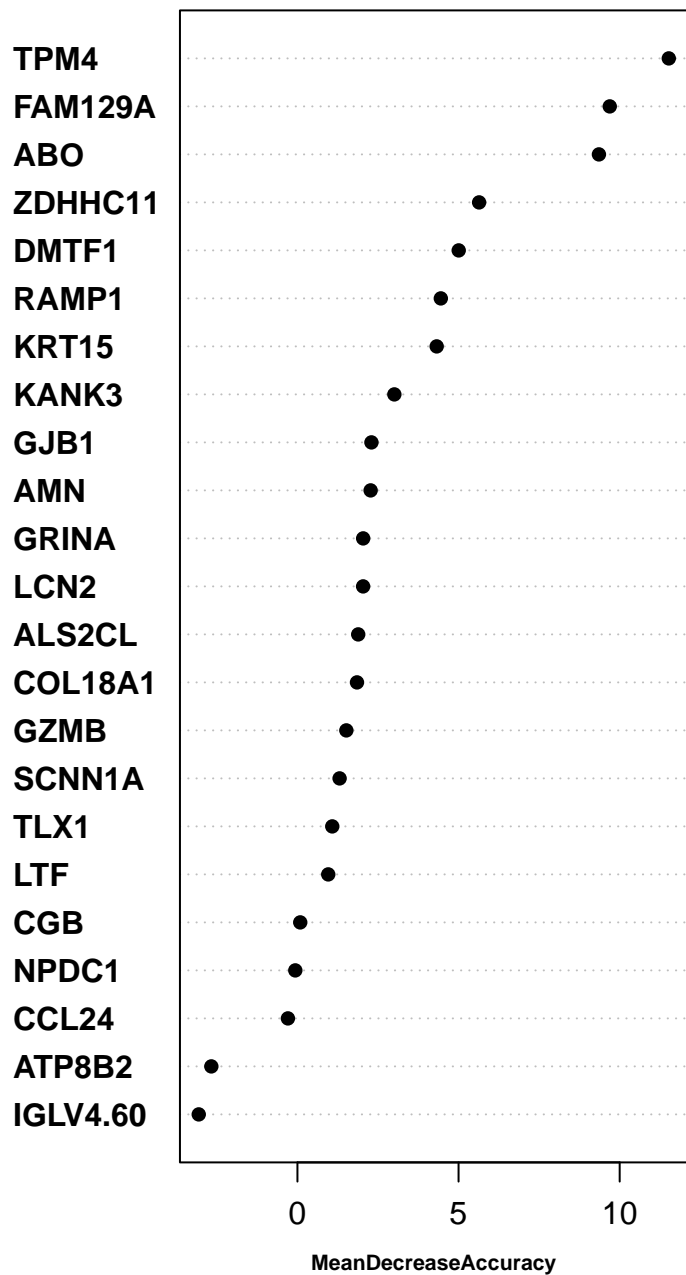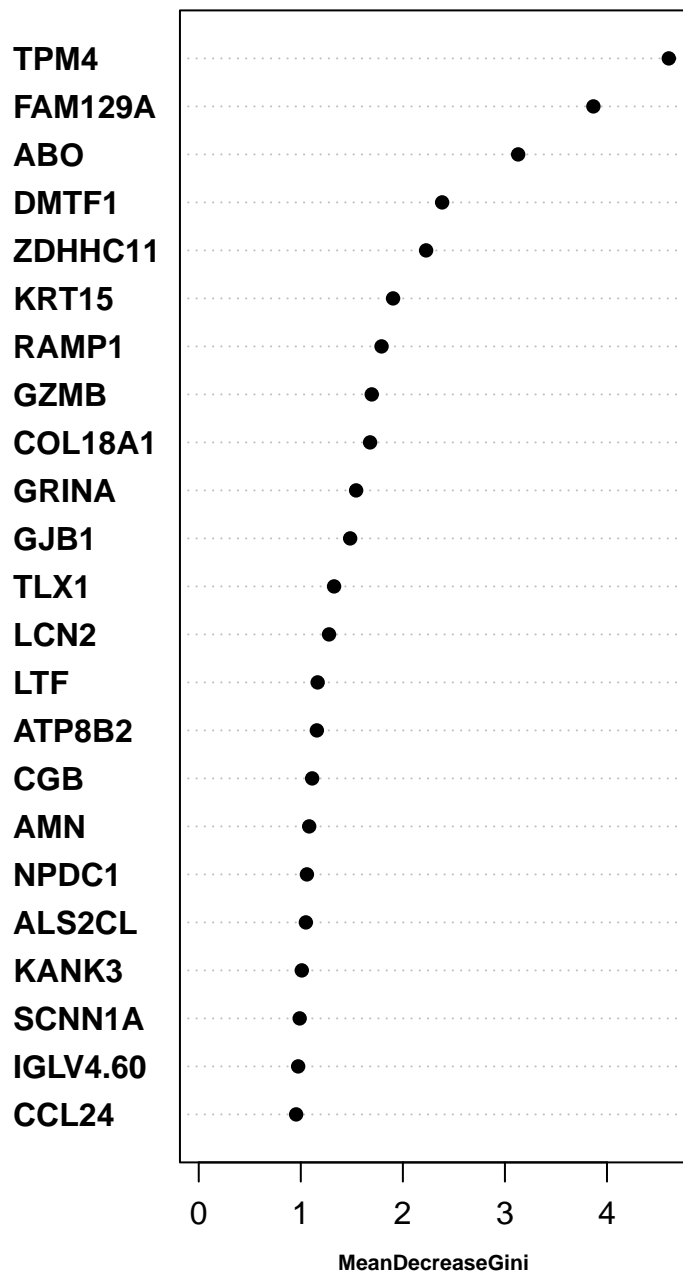

Supplement: Supplementary file 1 [file Supplementaryfile1.zip › Supplementary Material/03_ML/2.4_OP_RF_Variable_importance.pdf]

CD\_LASSO

CD\_RF

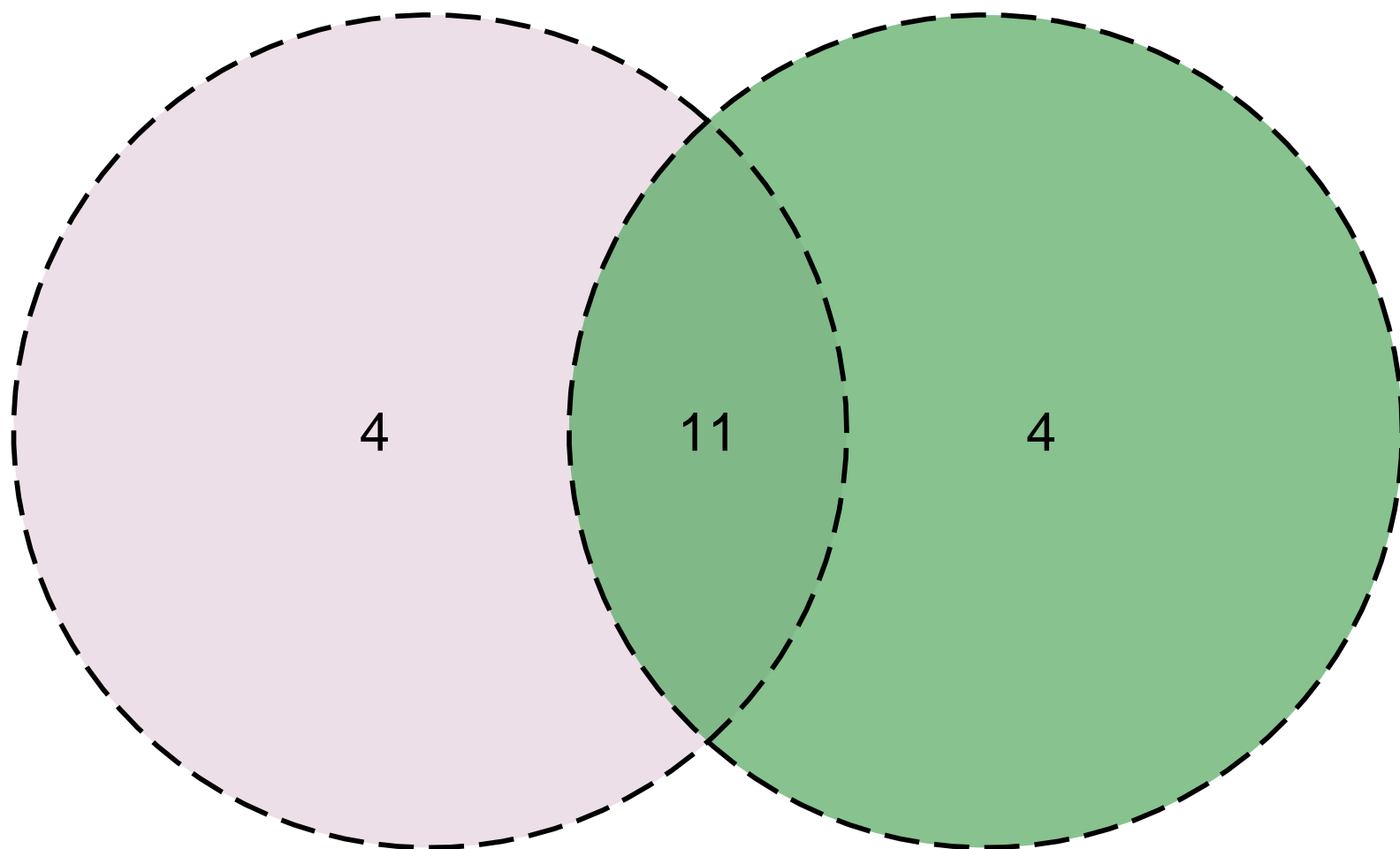

Supplement: Supplementary file 1 [file Supplementaryfile1.zip › Supplementary Material/03_ML/3.1_CD_LASSO_Inter_FR.pdf]

OP\_LASSO

OP\_RF

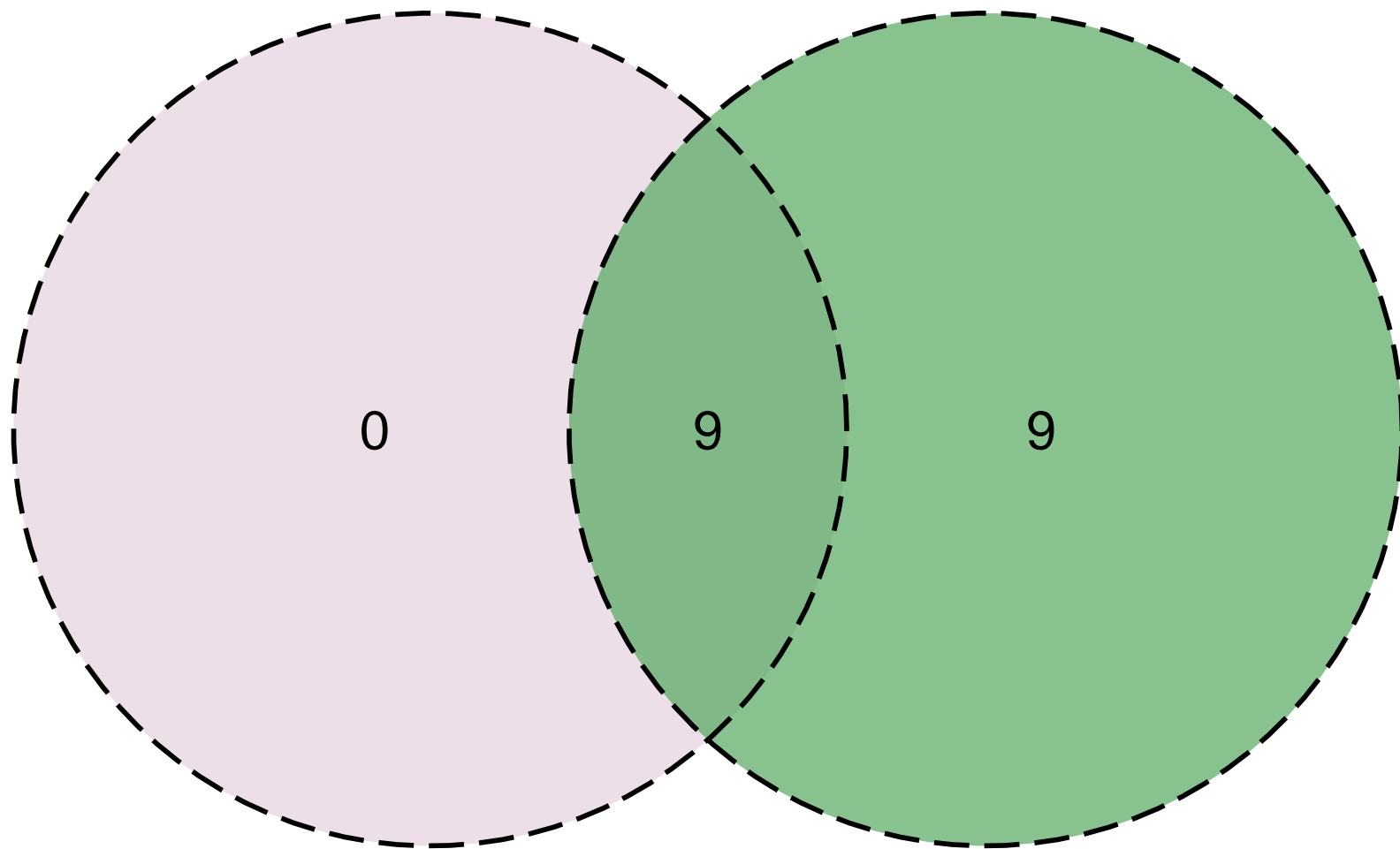

Supplement: Supplementary file 1 [file Supplementaryfile1.zip › Supplementary Material/03_ML/3.2_OP_LASSO_Inter_FR.pdf]

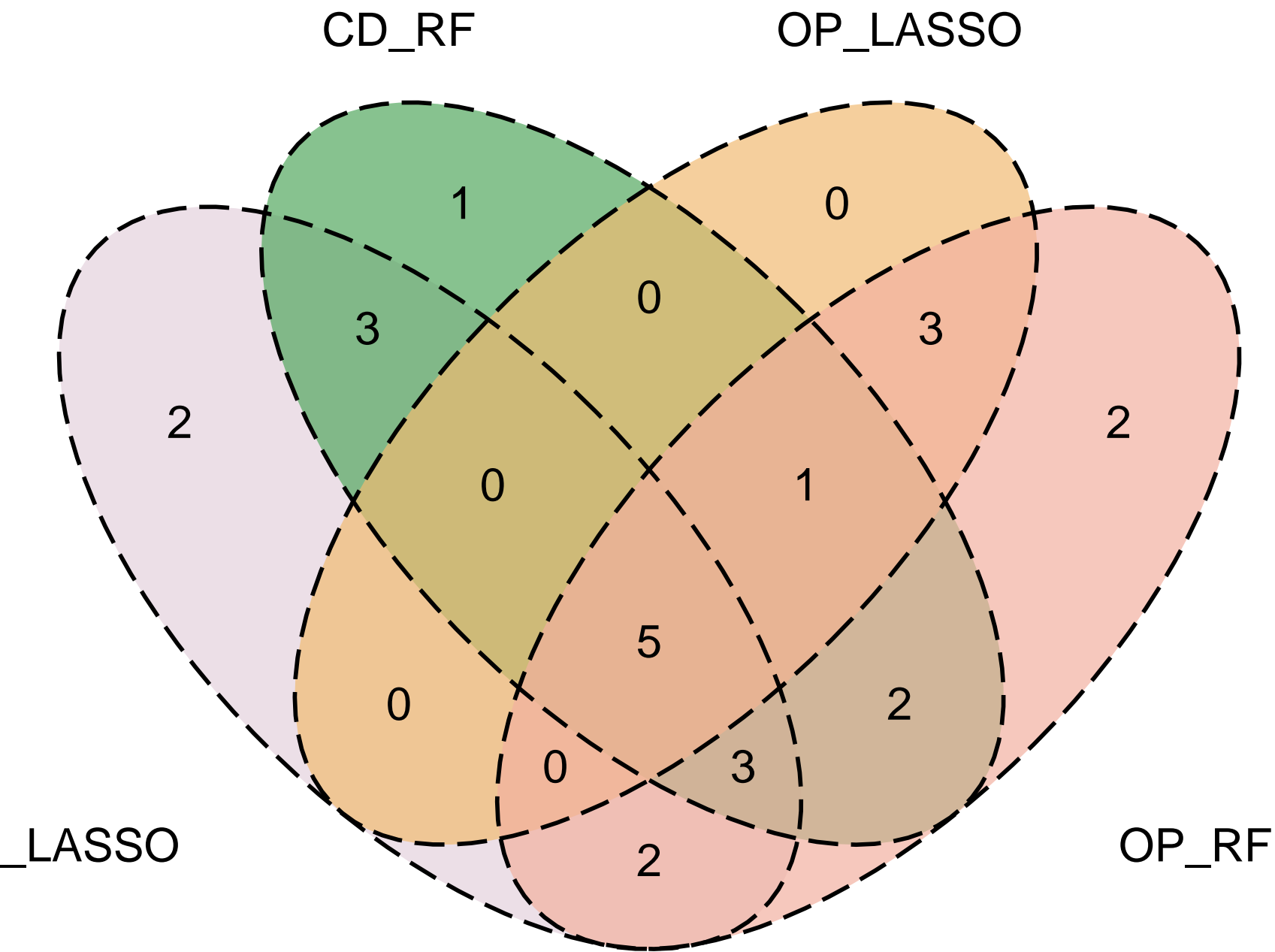

Supplement: Supplementary file 1 [file Supplementaryfile1.zip › Supplementary Material/03_ML/3.3_All_Inter_venn.pdf]

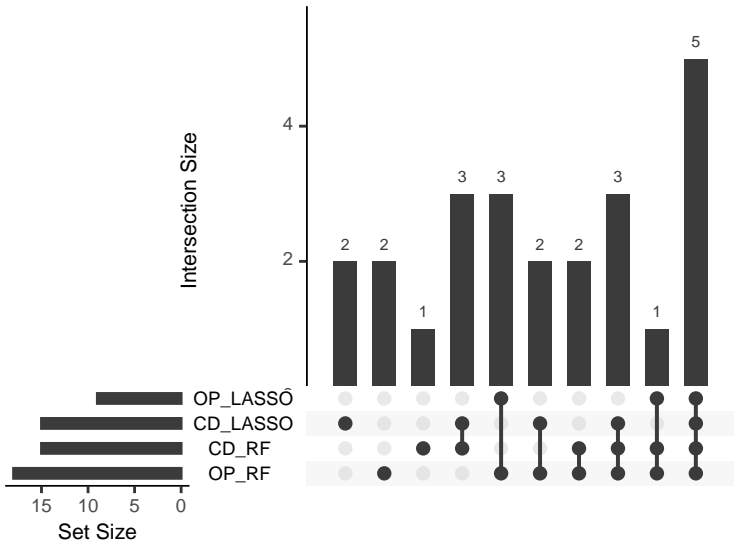

Supplement: Supplementary file 1 [file Supplementaryfile1.zip › Supplementary Material/03_ML/3.3_All_Inter_venn_upset.pdf]

Disease CD Normal

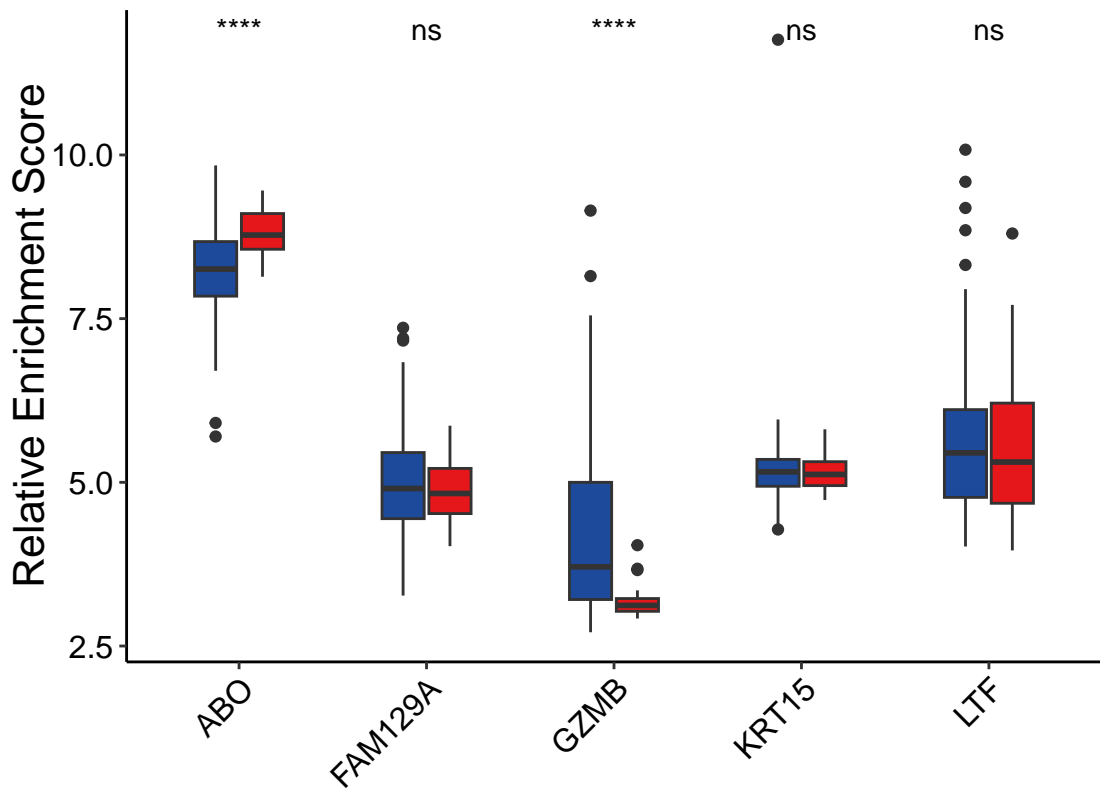

Supplement: Supplementary file 1 [file Supplementaryfile1.zip › Supplementary Material/03_ML/4.2_Common_gene_CD_Valid.pdf]

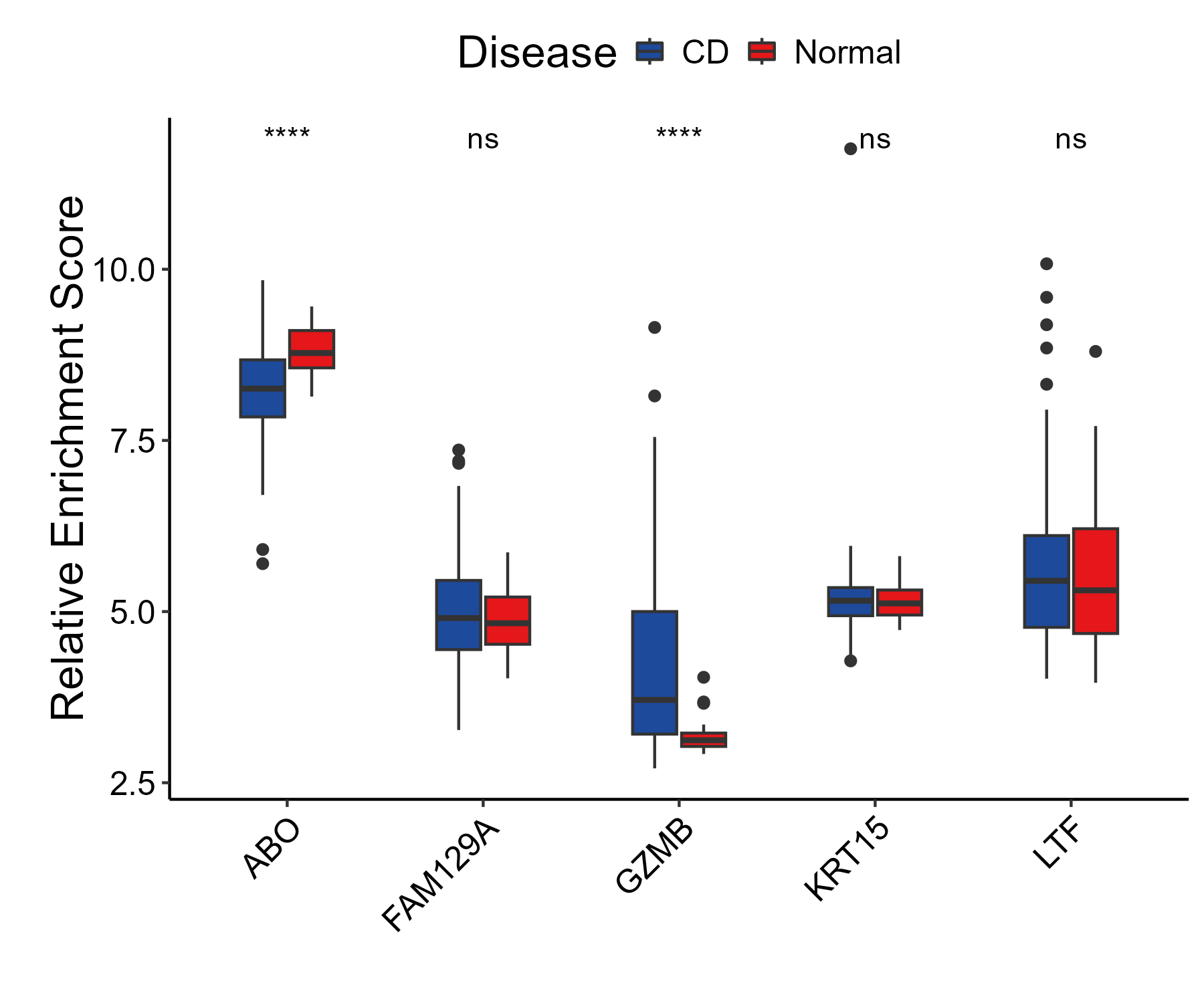

Supplement: Supplementary file 1 [file Supplementaryfile1.zip › Supplementary Material/03_ML/4.2_Common_gene_CD_Valid.png]

Disease

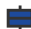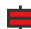

Normal

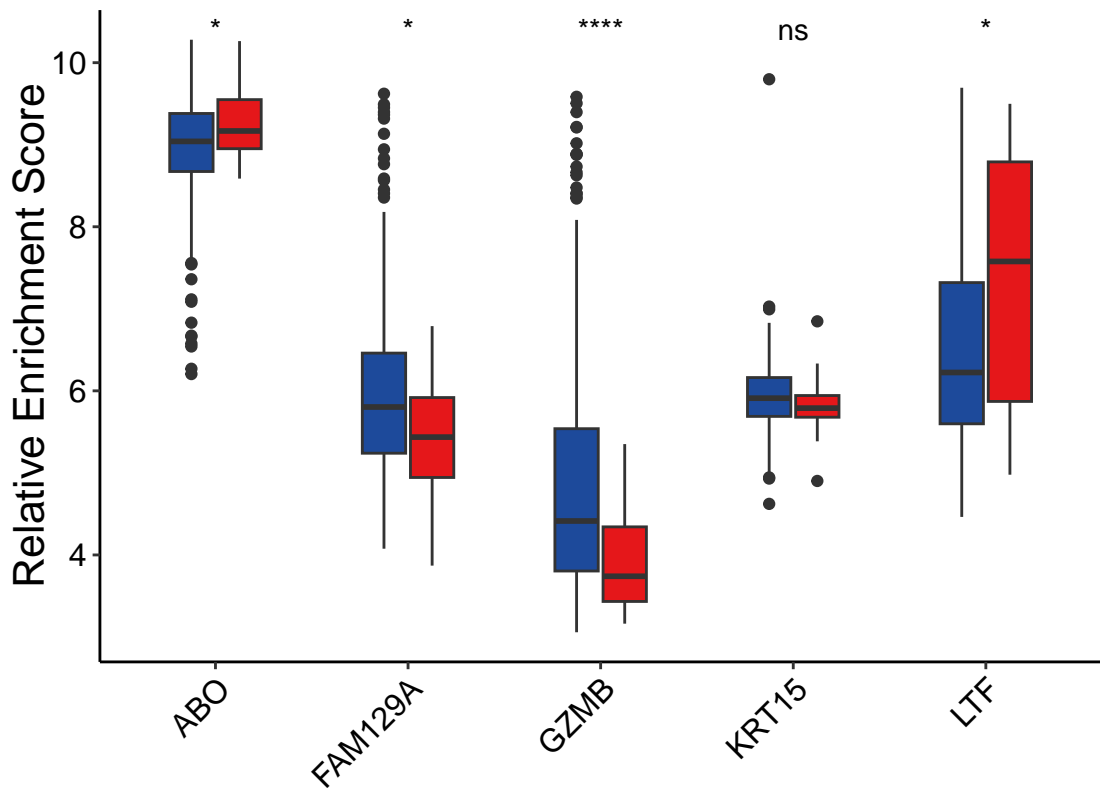

Supplement: Supplementary file 1 [file Supplementaryfile1.zip › Supplementary Material/03_ML/4.3_Common_gene_CD_Train.pdf]

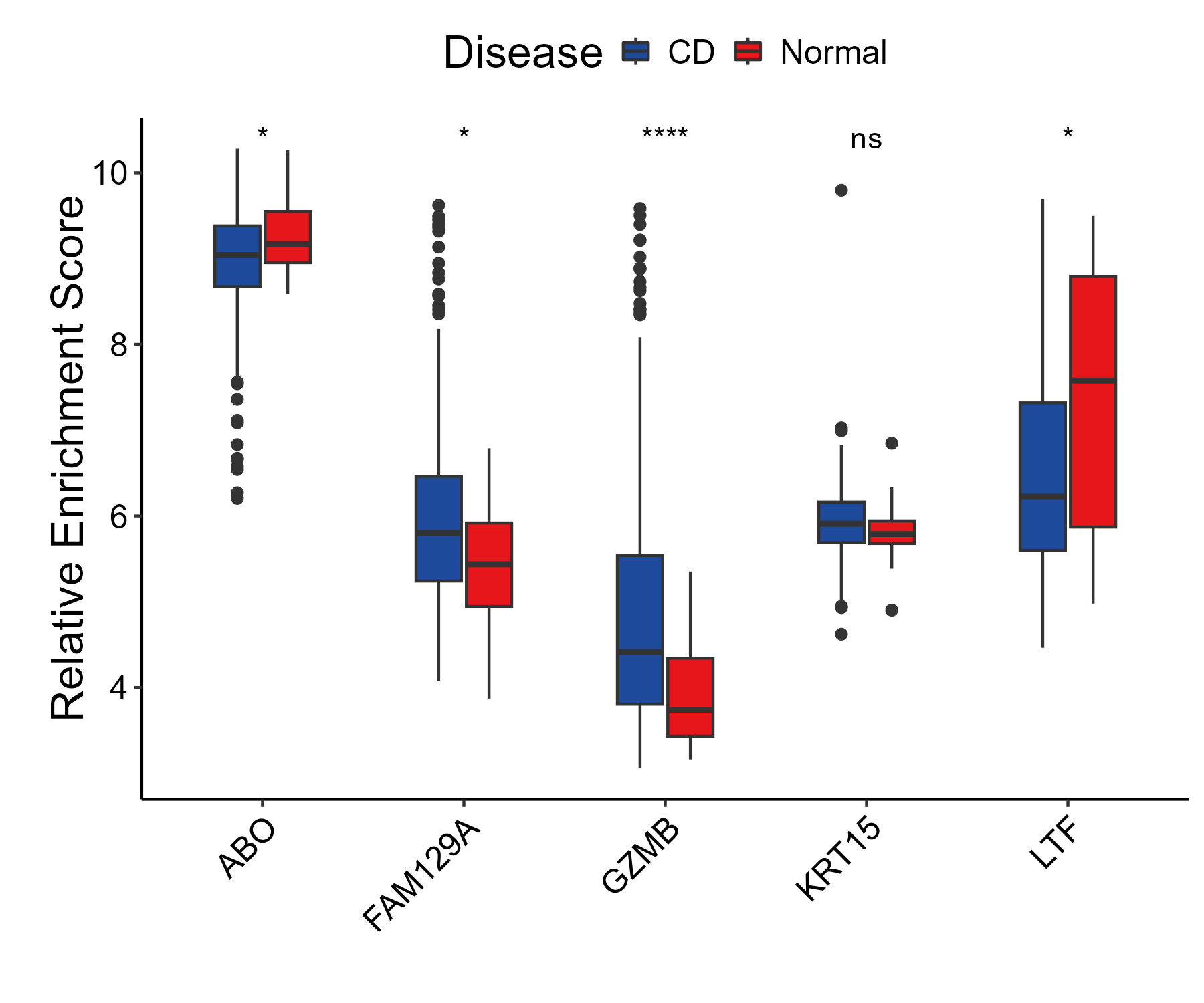

Supplement: Supplementary file 1 [file Supplementaryfile1.zip › Supplementary Material/03_ML/4.3_Common_gene_CD_Train.png]

Disease High\_BMD Low\_BMD

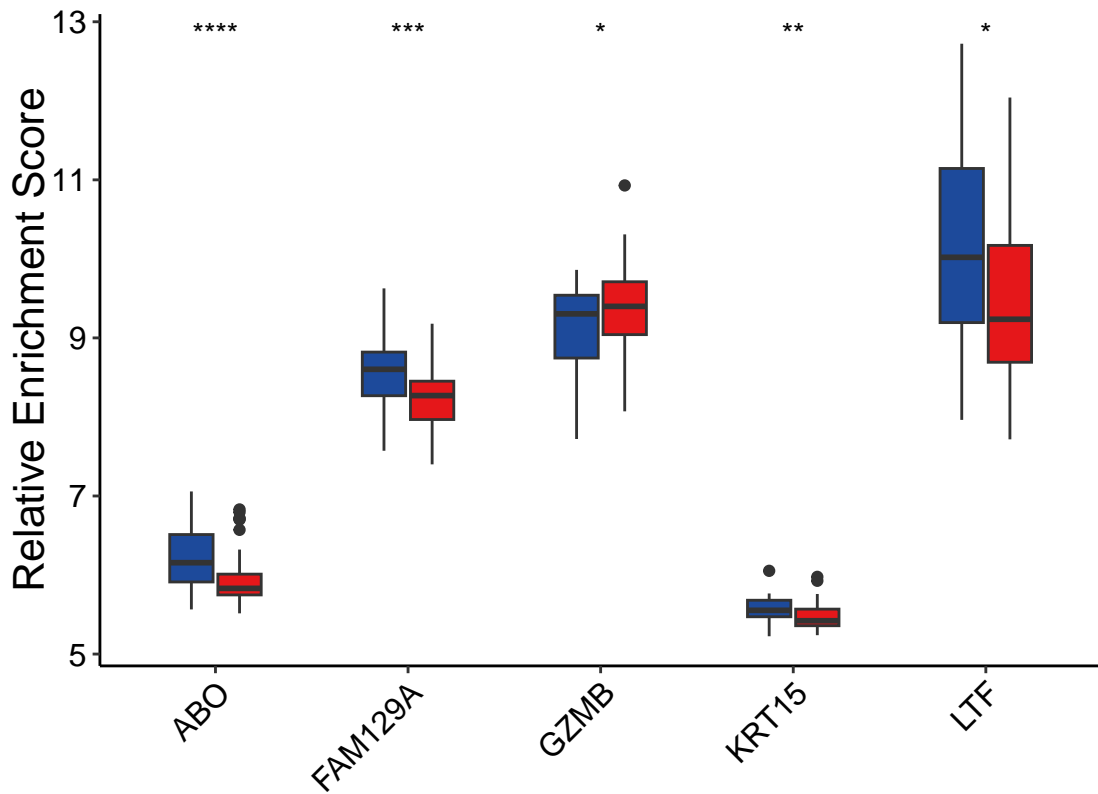

Supplement: Supplementary file 1 [file Supplementaryfile1.zip › Supplementary Material/03_ML/4.8_Common_gene_OP_Train.pdf]

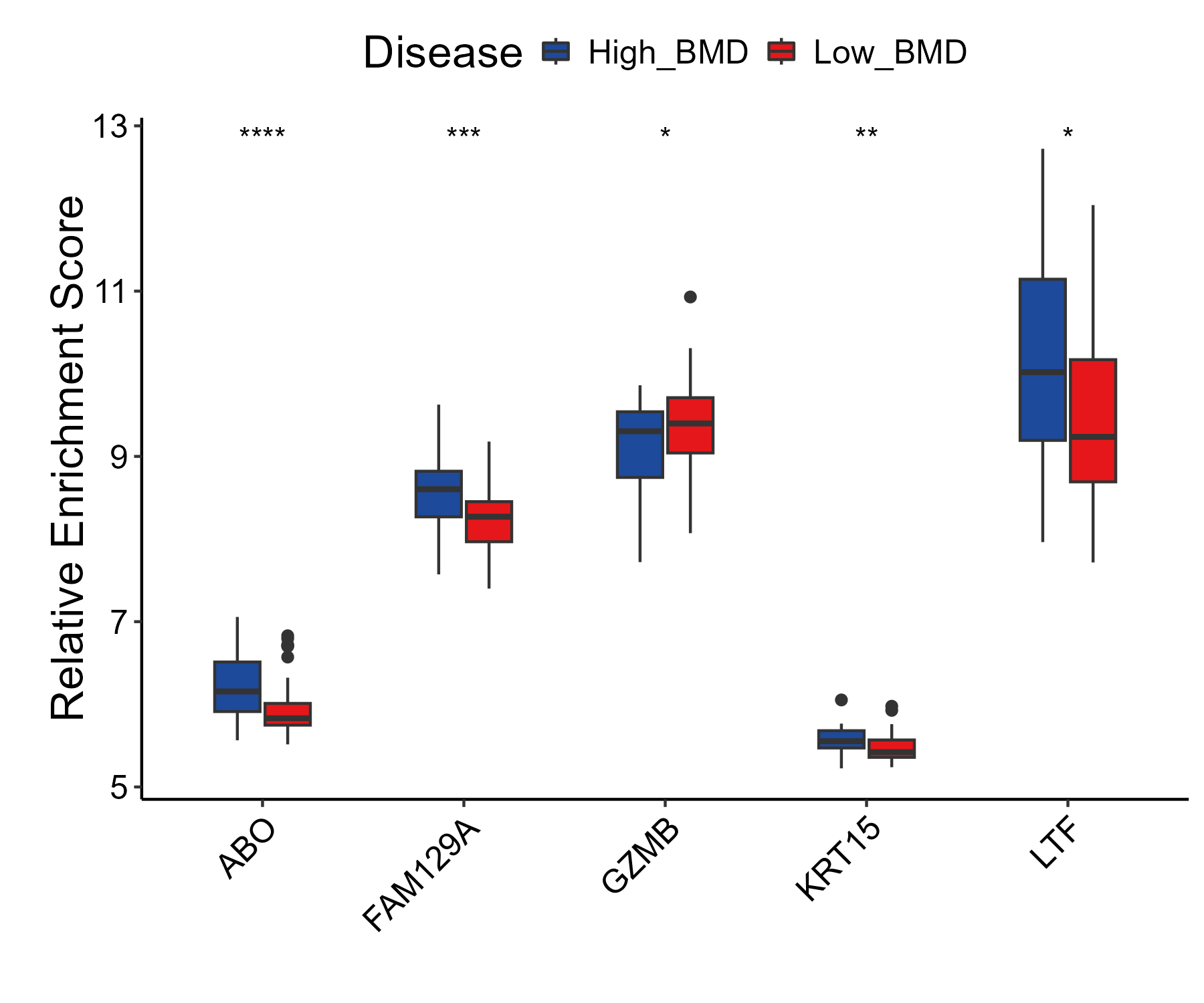

Supplement: Supplementary file 1 [file Supplementaryfile1.zip › Supplementary Material/03_ML/4.8_Common_gene_OP_Train.png]

Disease High\_BMD Low\_BMD

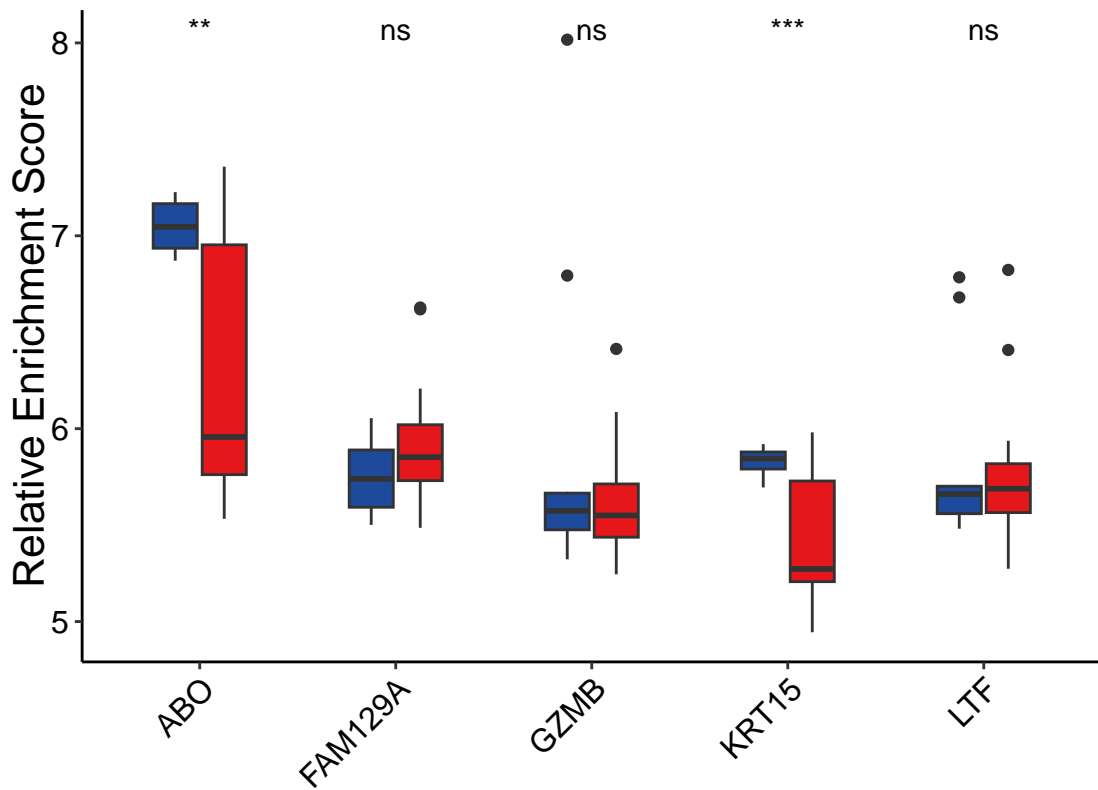

Supplement: Supplementary file 1 [file Supplementaryfile1.zip › Supplementary Material/03_ML/4.9_Common_gene_OP_Valid.pdf.pdf]

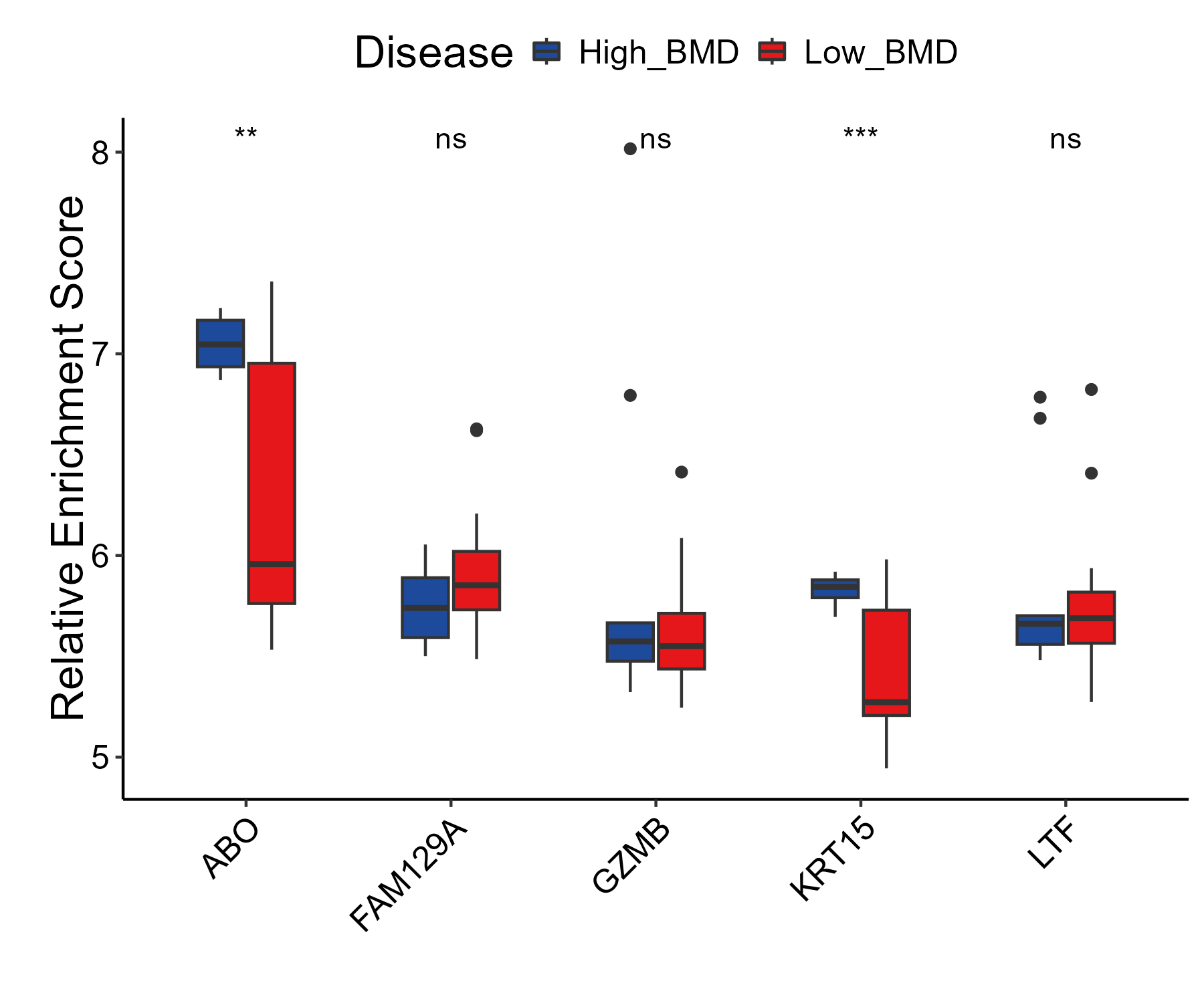

Supplement: Supplementary file 1 [file Supplementaryfile1.zip › Supplementary Material/03_ML/4.9_Common_gene_OP_Valid.pdf.png]

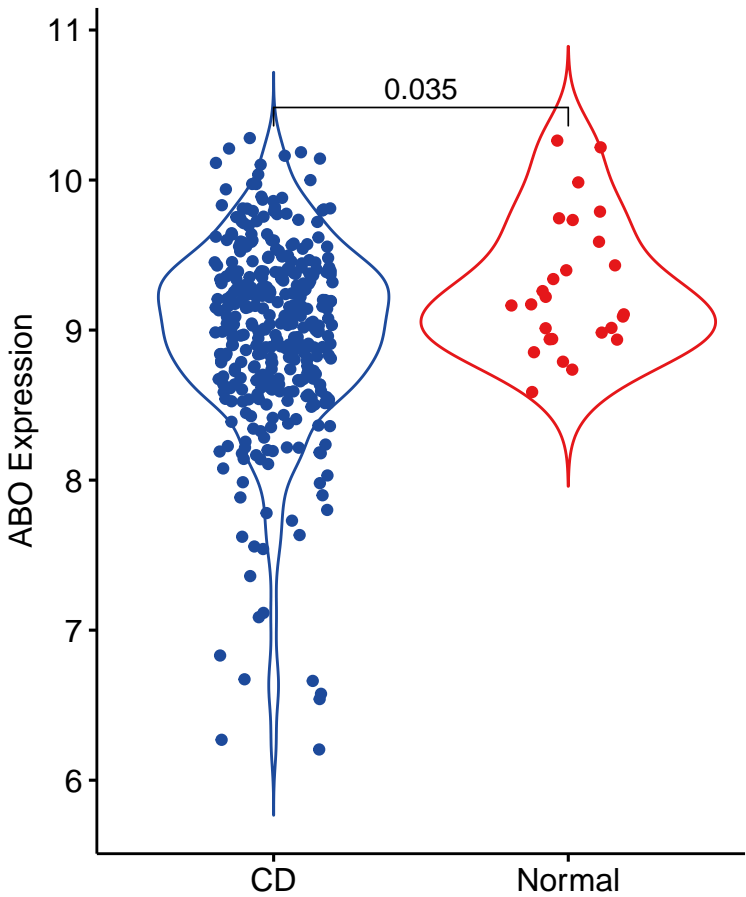

Supplement: Supplementary file 1 [file Supplementaryfile1.zip › Supplementary Material/03_ML/5.1_CD_Train_ABO.pdf]

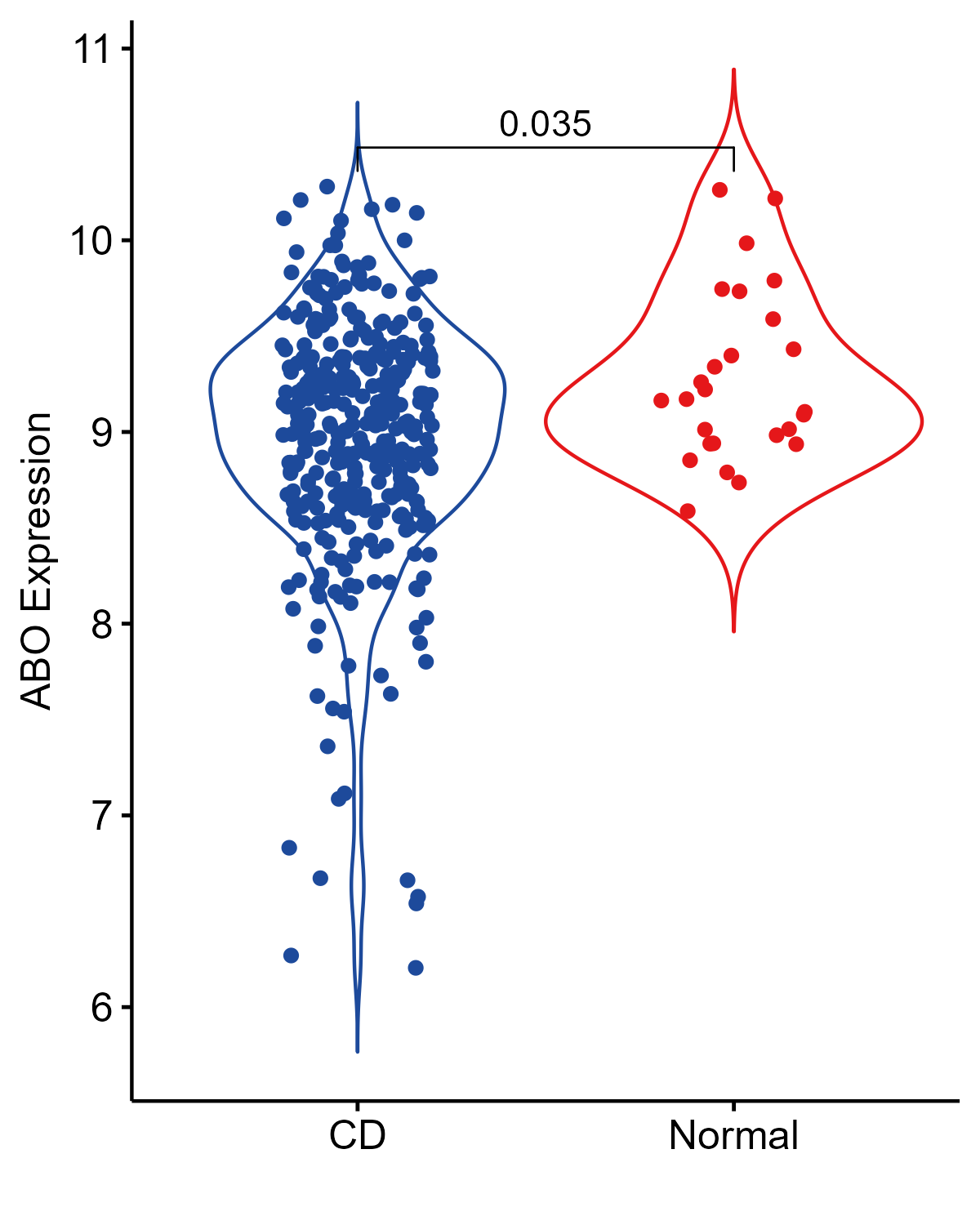

Supplement: Supplementary file 1 [file Supplementaryfile1.zip › Supplementary Material/03_ML/5.1_CD_Train_ABO.png]

FAM129A Expression

10

8

6

4

0.028

CD

Normal

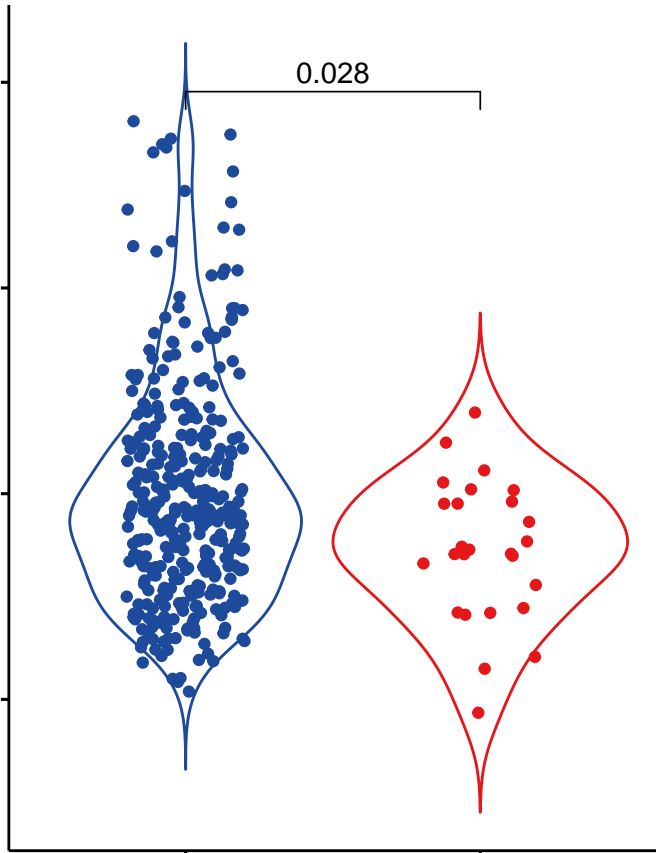

Supplement: Supplementary file 1 [file Supplementaryfile1.zip › Supplementary Material/03_ML/5.1_CD_Train_FAM129A.pdf]

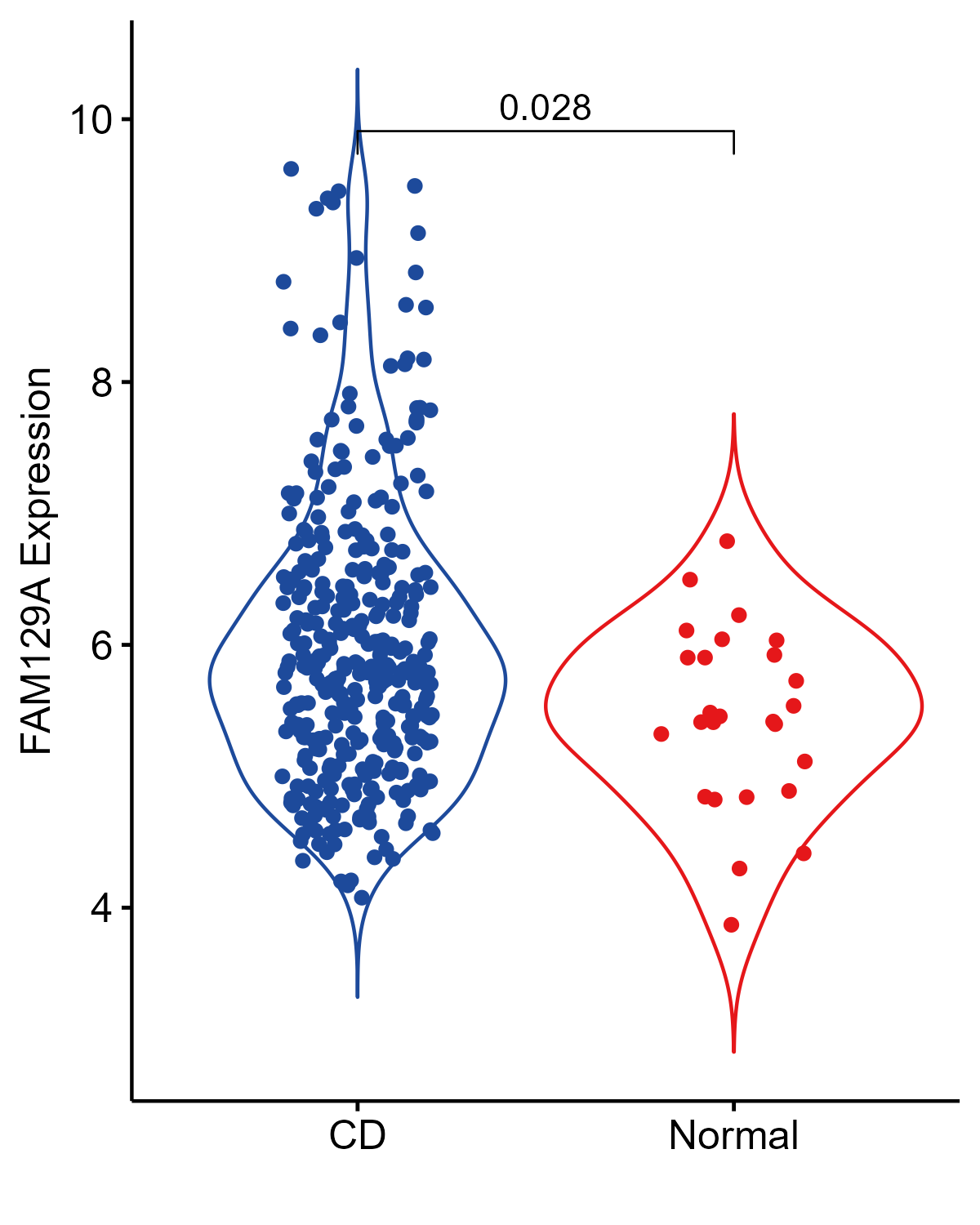

Supplement: Supplementary file 1 [file Supplementaryfile1.zip › Supplementary Material/03_ML/5.1_CD_Train_FAM129A.png]

GZMB Expression

10.0

7.5

5.0

2.5

$8.5e-05$

CD

Normal

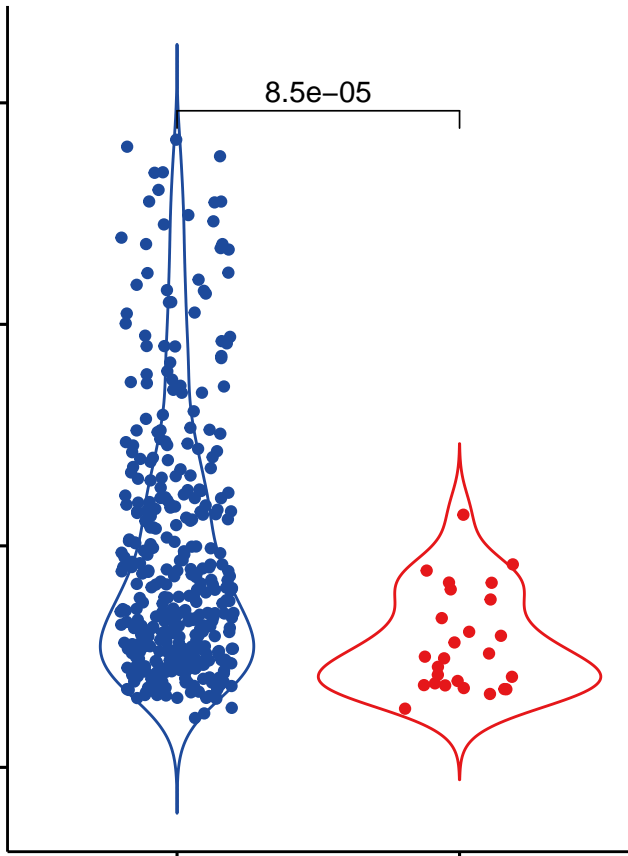

Supplement: Supplementary file 1 [file Supplementaryfile1.zip › Supplementary Material/03_ML/5.1_CD_Train_GZMB.pdf]

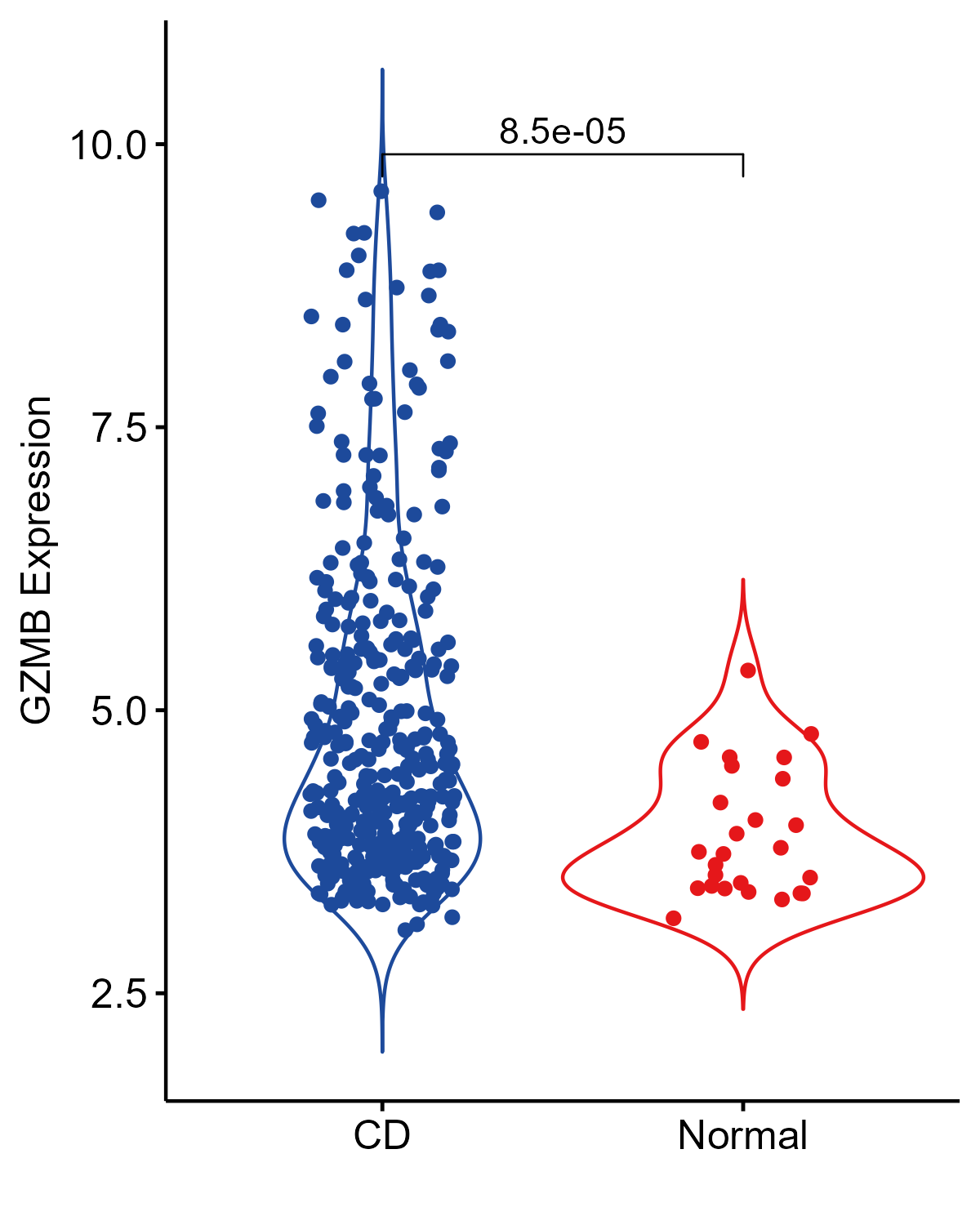

Supplement: Supplementary file 1 [file Supplementaryfile1.zip › Supplementary Material/03_ML/5.1_CD_Train_GZMB.png]

LTF Expression

10.0

7.5

5.0

2.5

0.018

CD

Normal

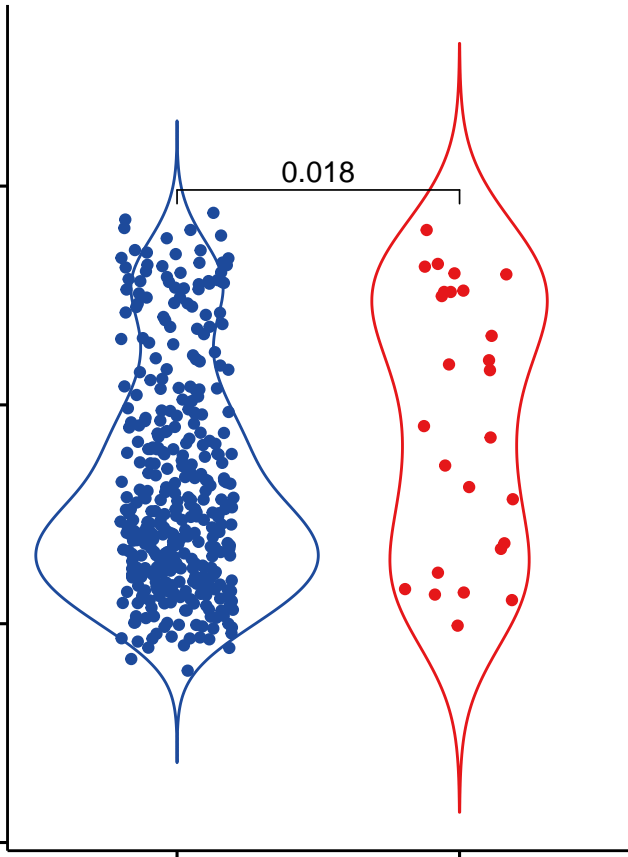

Supplement: Supplementary file 1 [file Supplementaryfile1.zip › Supplementary Material/03_ML/5.1_CD_Train_LTF.pdf]

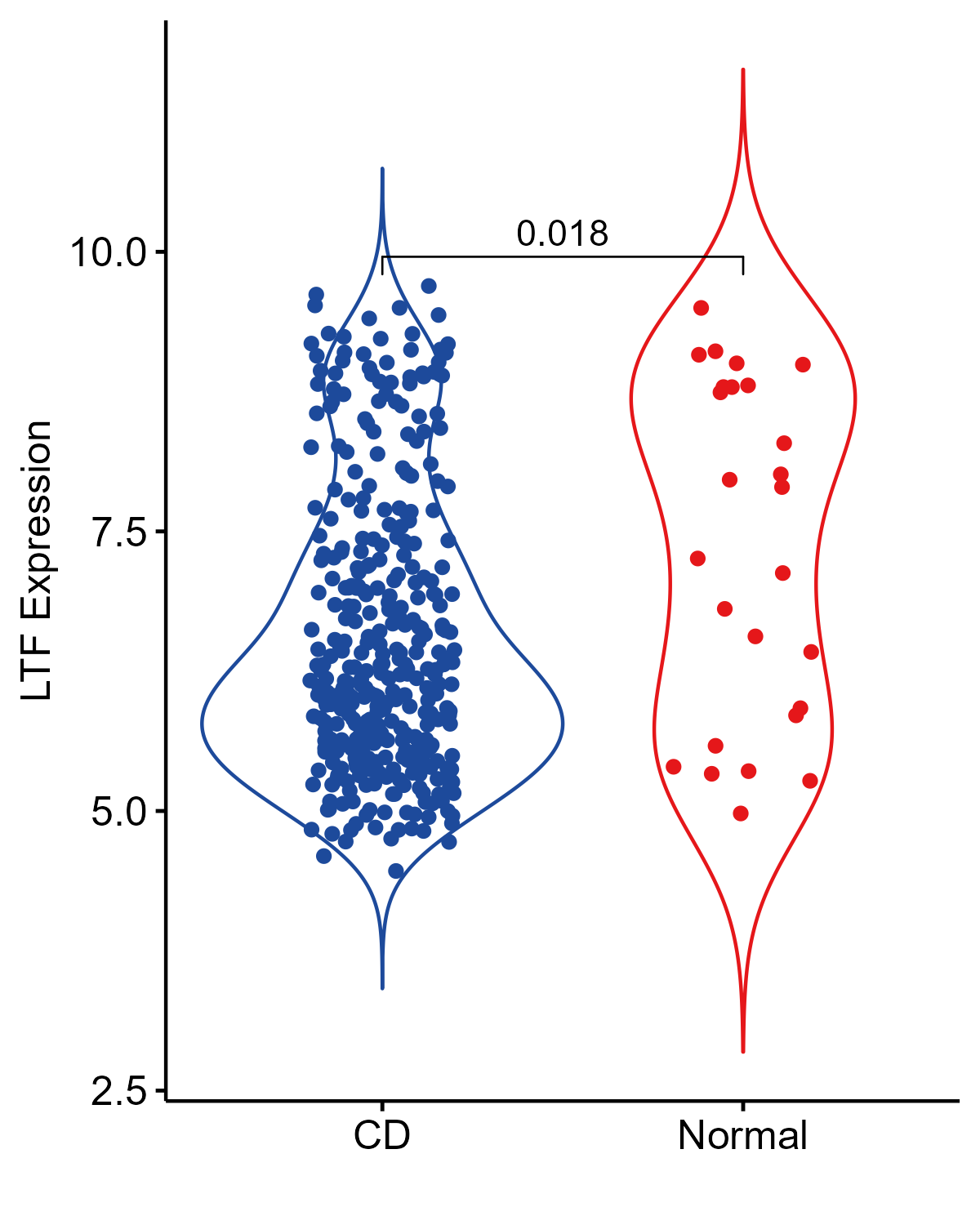

Supplement: Supplementary file 1 [file Supplementaryfile1.zip › Supplementary Material/03_ML/5.1_CD_Train_LTF.png]

## ABO Train

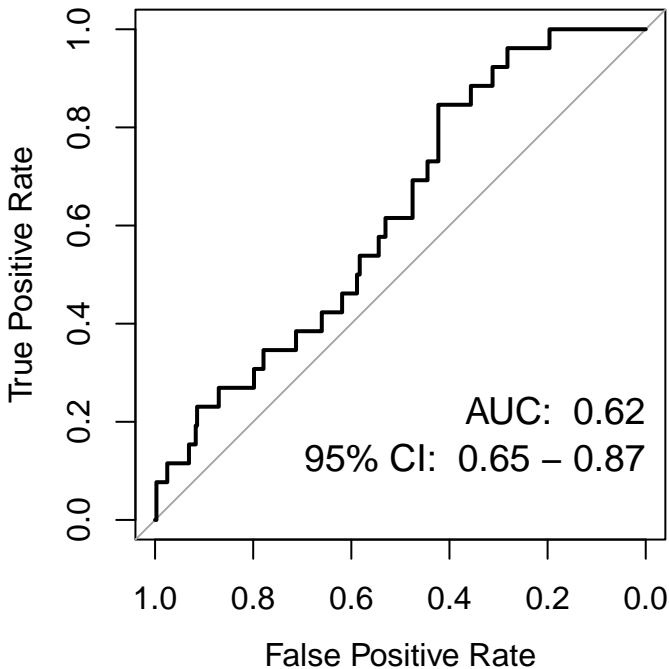

Supplement: Supplementary file 1 [file Supplementaryfile1.zip › Supplementary Material/03_ML/5.1_CD_Train_ROC_ABO.pdf]

## FAM129A Train

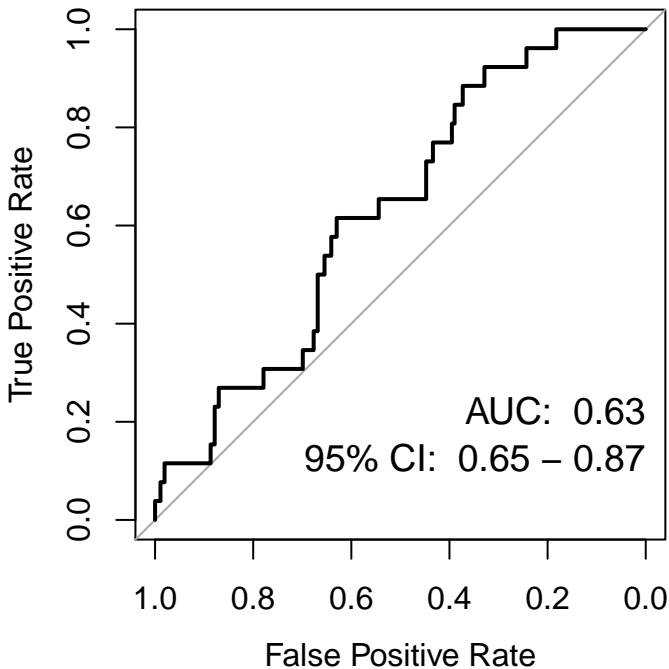

Supplement: Supplementary file 1 [file Supplementaryfile1.zip › Supplementary Material/03_ML/5.1_CD_Train_ROC_FAM129A.pdf]

## GZMB Train

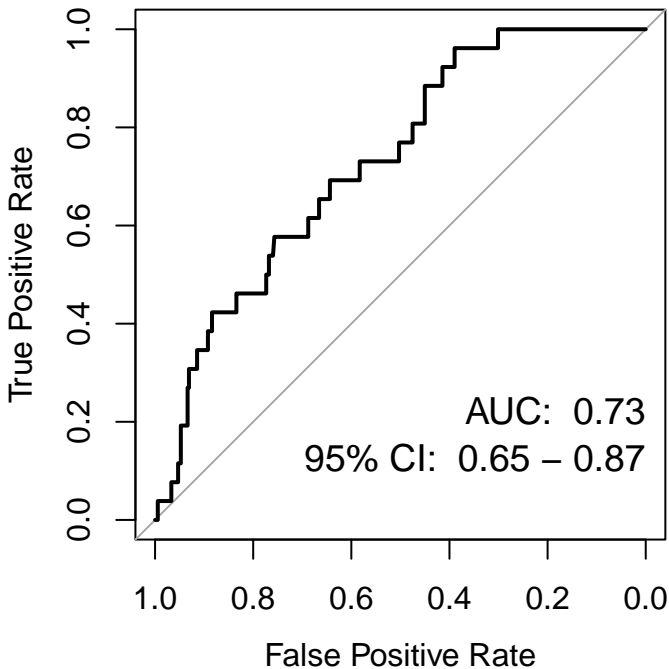

Supplement: Supplementary file 1 [file Supplementaryfile1.zip › Supplementary Material/03_ML/5.1_CD_Train_ROC_GZMB.pdf]

## LTF Train

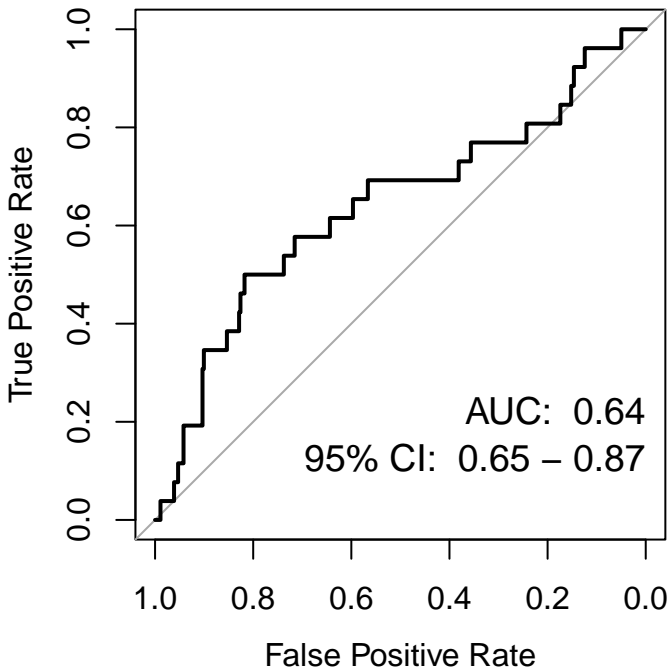

Supplement: Supplementary file 1 [file Supplementaryfile1.zip › Supplementary Material/03_ML/5.1_CD_Train_ROC_LTF.pdf]

ABO Expression

10  
9  
8  
7  
6  
5

$5.1 \times 10^{-5}$

CD

Normal

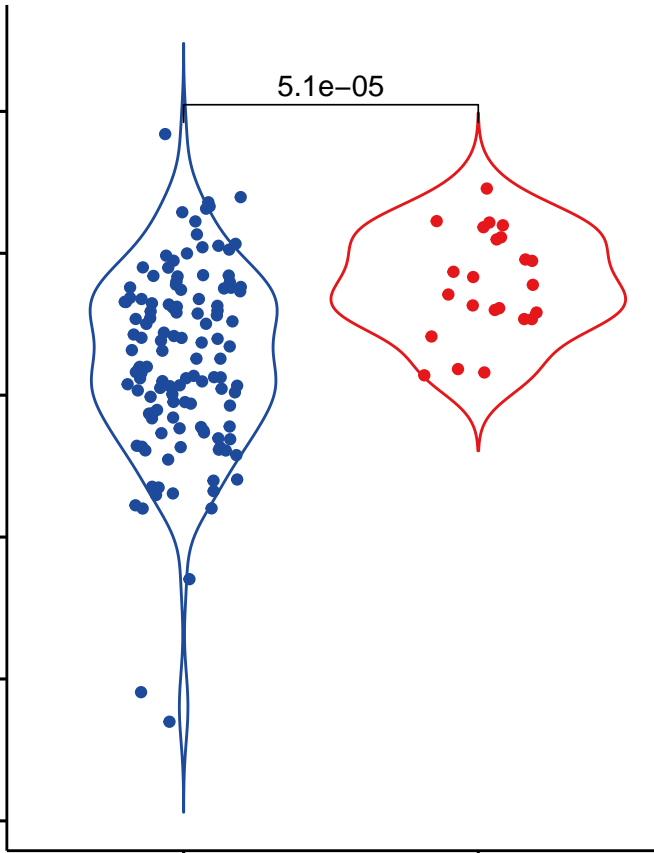

Supplement: Supplementary file 1 [file Supplementaryfile1.zip › Supplementary Material/03_ML/5.2_CD_Valid_ABO.pdf]

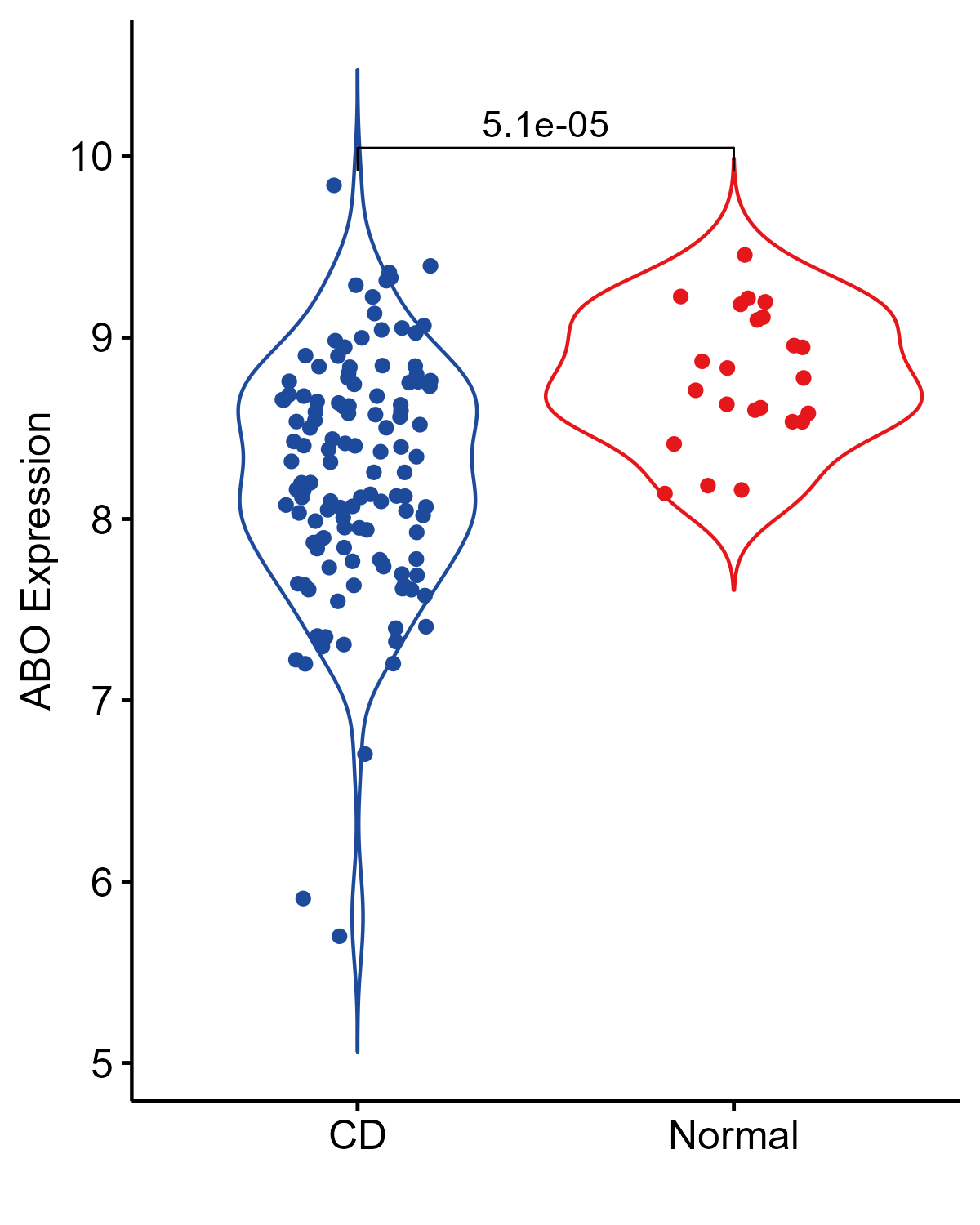

Supplement: Supplementary file 1 [file Supplementaryfile1.zip › Supplementary Material/03_ML/5.2_CD_Valid_ABO.png]

GZMB Expression

10.0

7.5

5.0

2.5

$1.2e-05$

CD

Normal

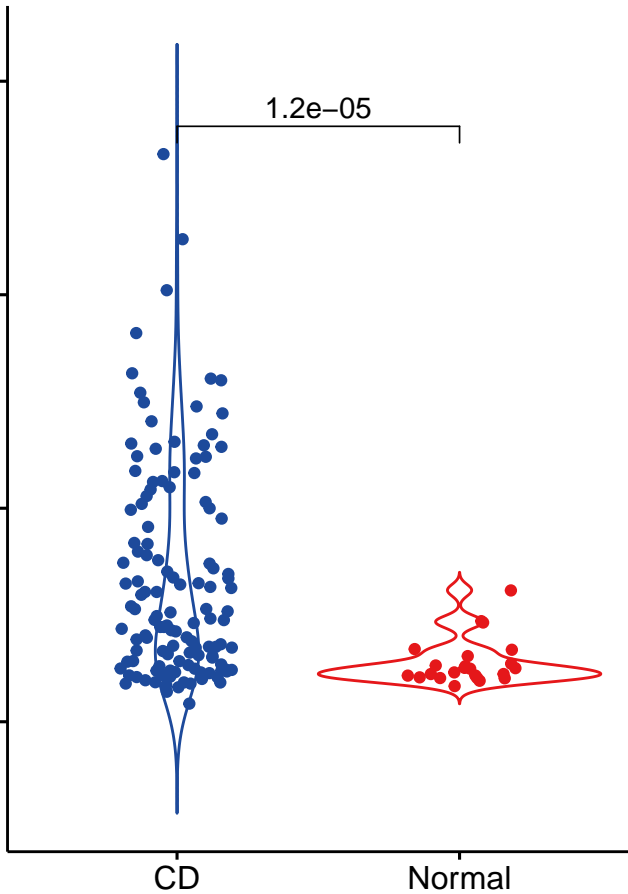

Supplement: Supplementary file 1 [file Supplementaryfile1.zip › Supplementary Material/03_ML/5.2_CD_Valid_GZMB.pdf]

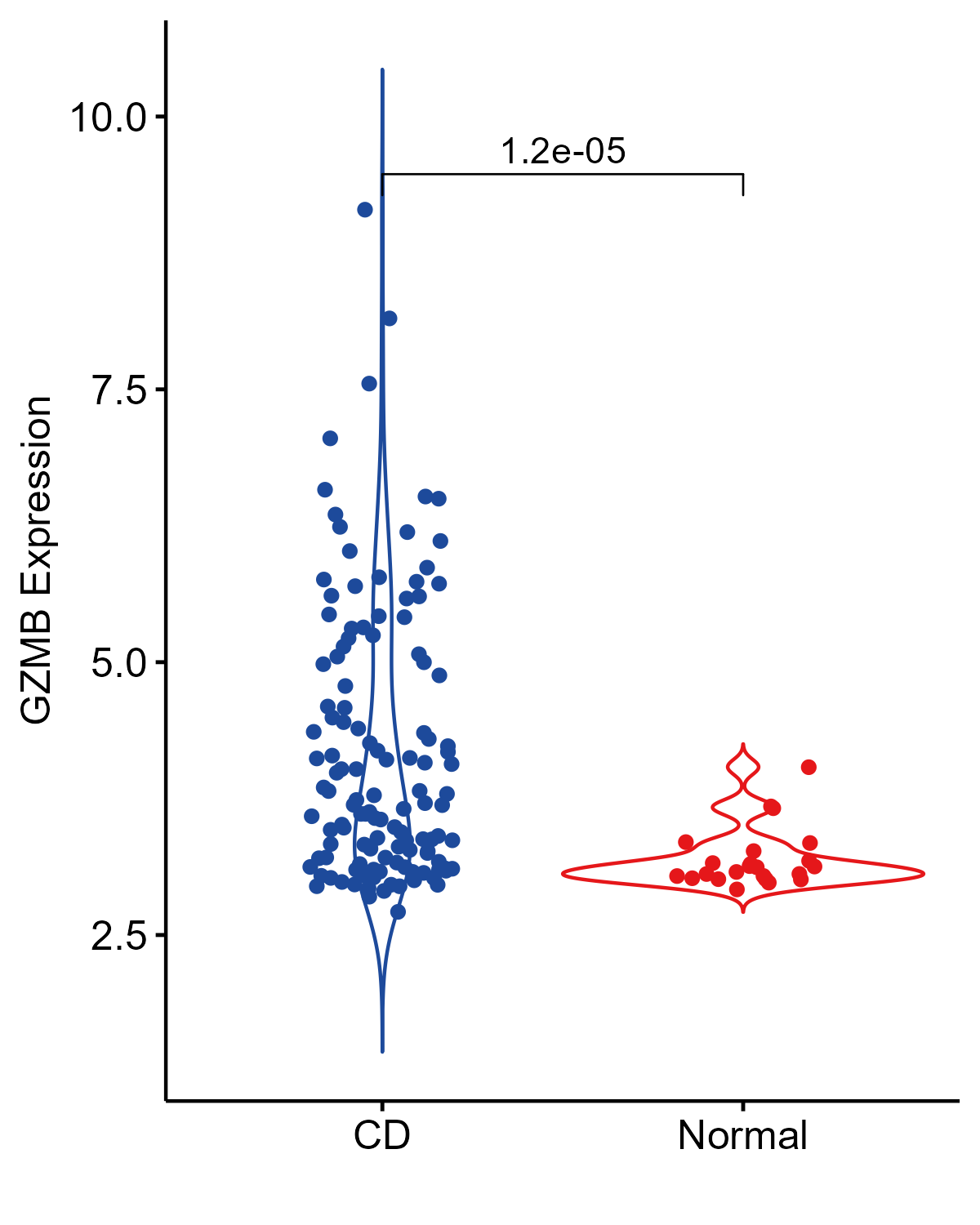

Supplement: Supplementary file 1 [file Supplementaryfile1.zip › Supplementary Material/03_ML/5.2_CD_Valid_GZMB.png]

## ABO Valid

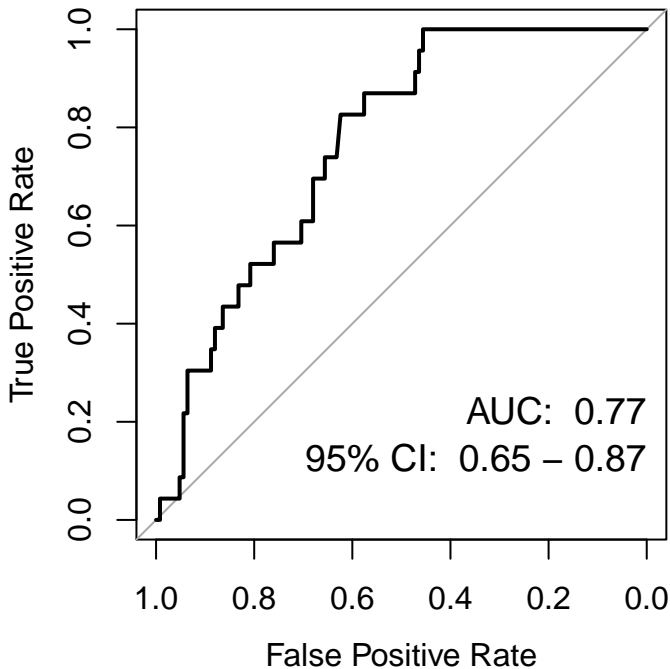

Supplement: Supplementary file 1 [file Supplementaryfile1.zip › Supplementary Material/03_ML/5.2_CD_Valid_ROC_ABO.pdf]

## GZMB Valid

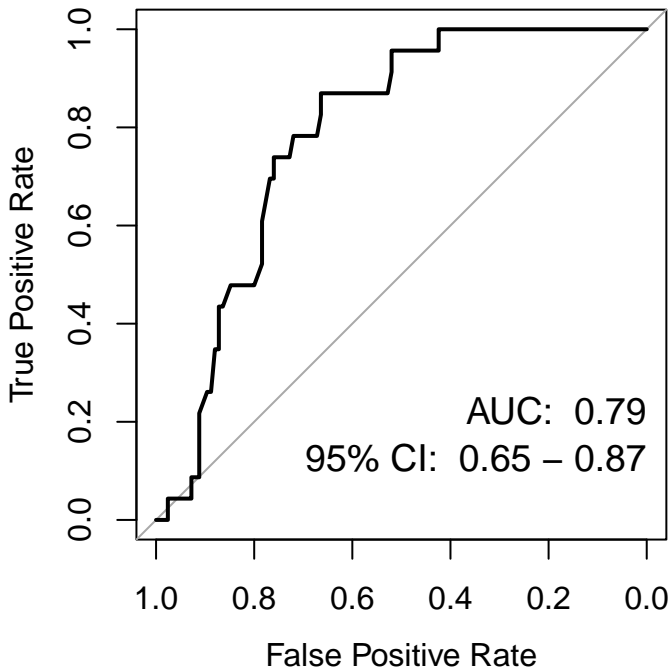

Supplement: Supplementary file 1 [file Supplementaryfile1.zip › Supplementary Material/03_ML/5.2_CD_Valid_ROC_GZMB.pdf]

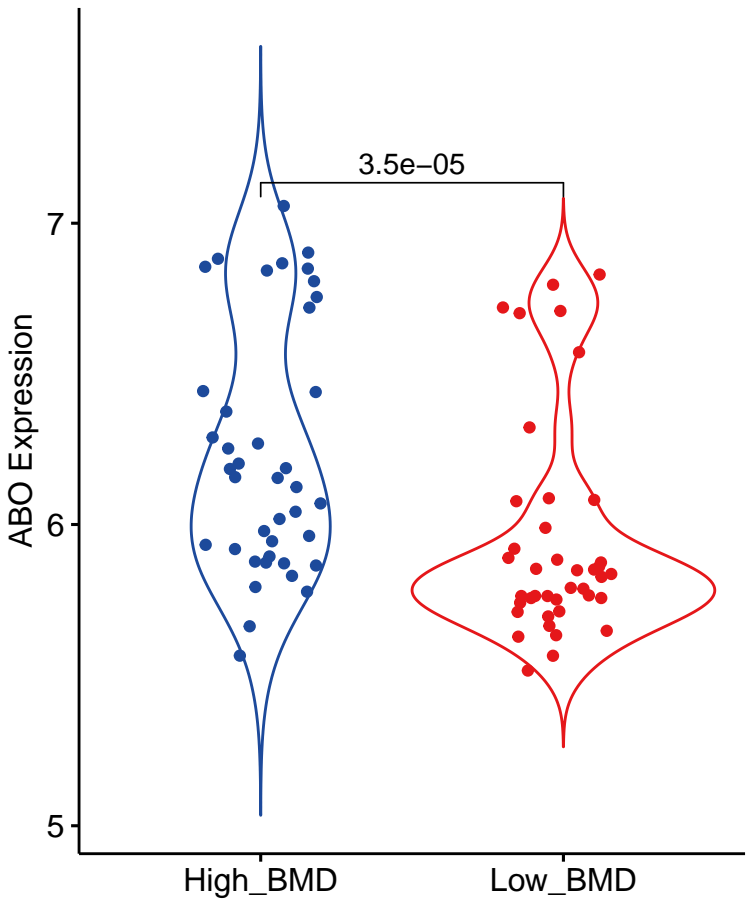

Supplement: Supplementary file 1 [file Supplementaryfile1.zip › Supplementary Material/03_ML/5.3_OP_Train_ABO.pdf]

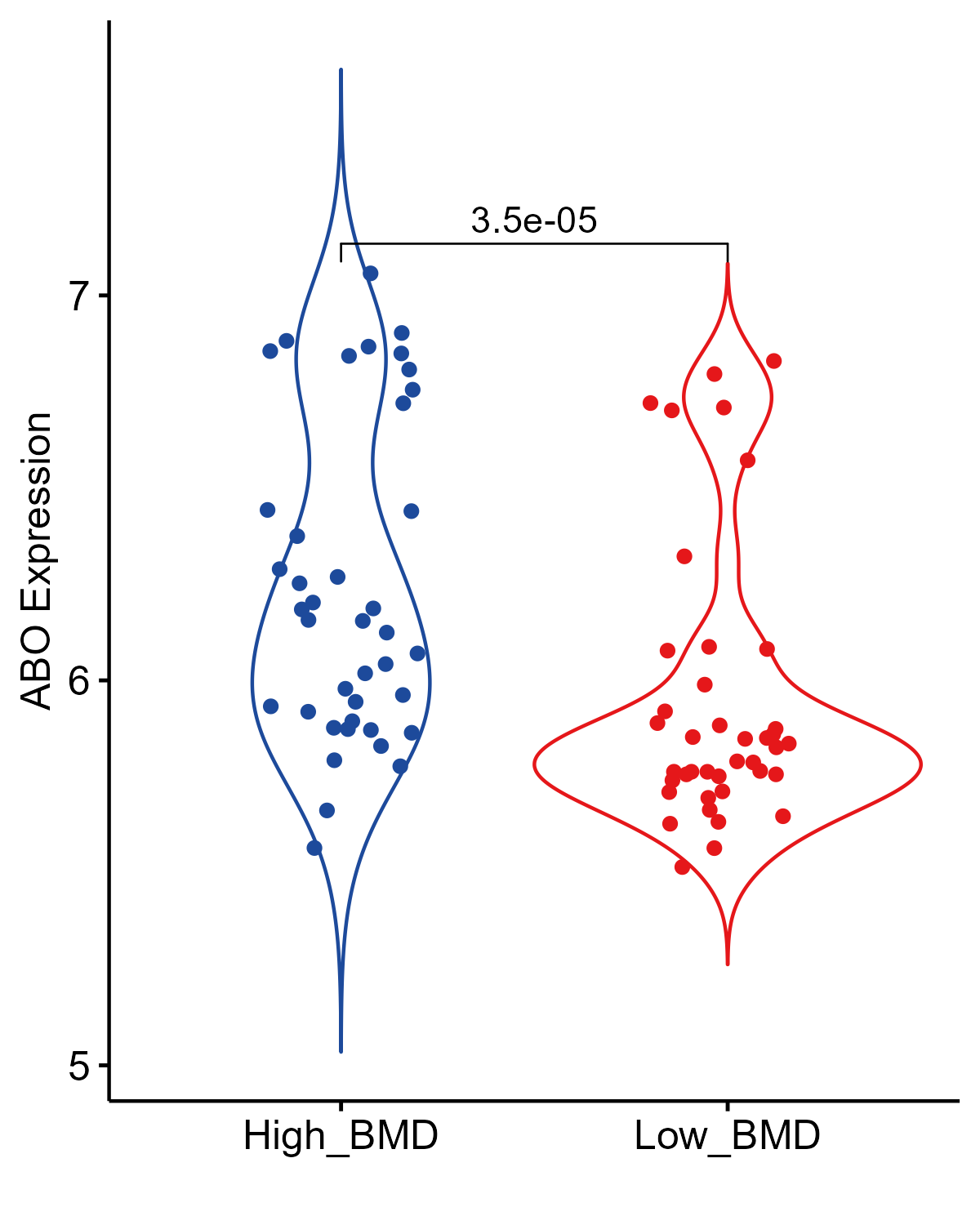

Supplement: Supplementary file 1 [file Supplementaryfile1.zip › Supplementary Material/03_ML/5.3_OP_Train_ABO.png]

FAM129A Expression

10

9

8

7

0.00029

High\_BMD

Low\_BMD

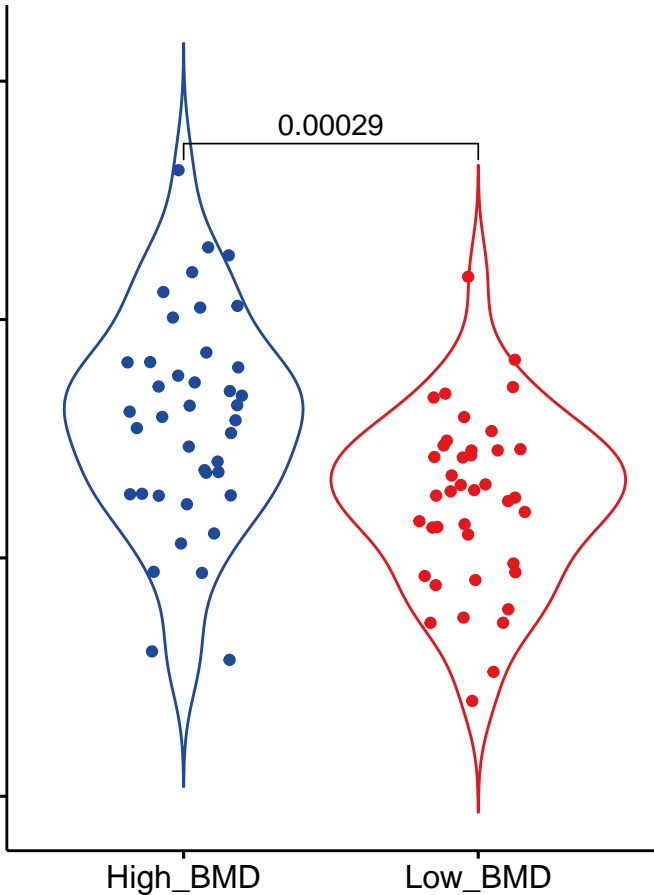

Supplement: Supplementary file 1 [file Supplementaryfile1.zip › Supplementary Material/03_ML/5.3_OP_Train_FAM129A.pdf]

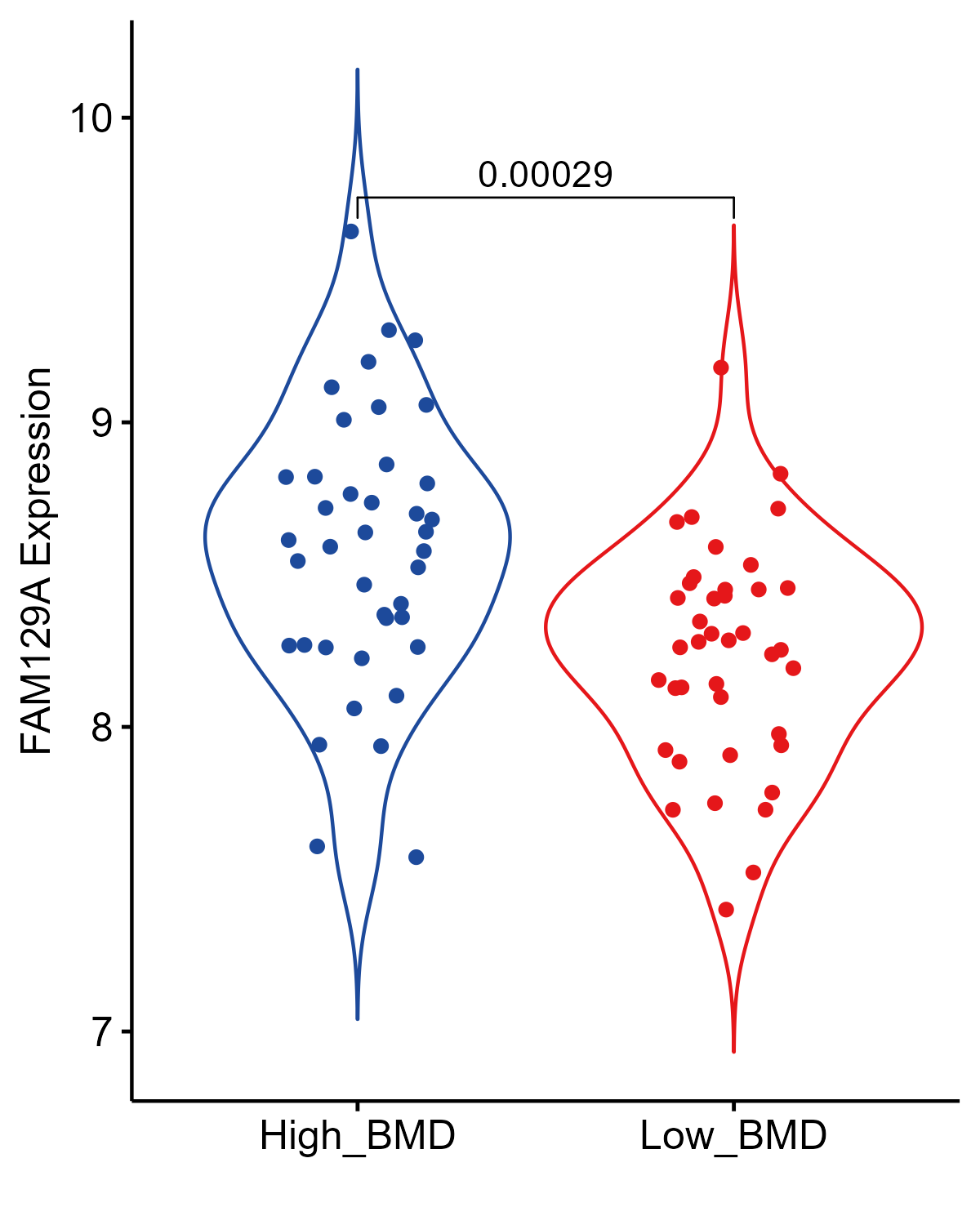

Supplement: Supplementary file 1 [file Supplementaryfile1.zip › Supplementary Material/03_ML/5.3_OP_Train_FAM129A.png]

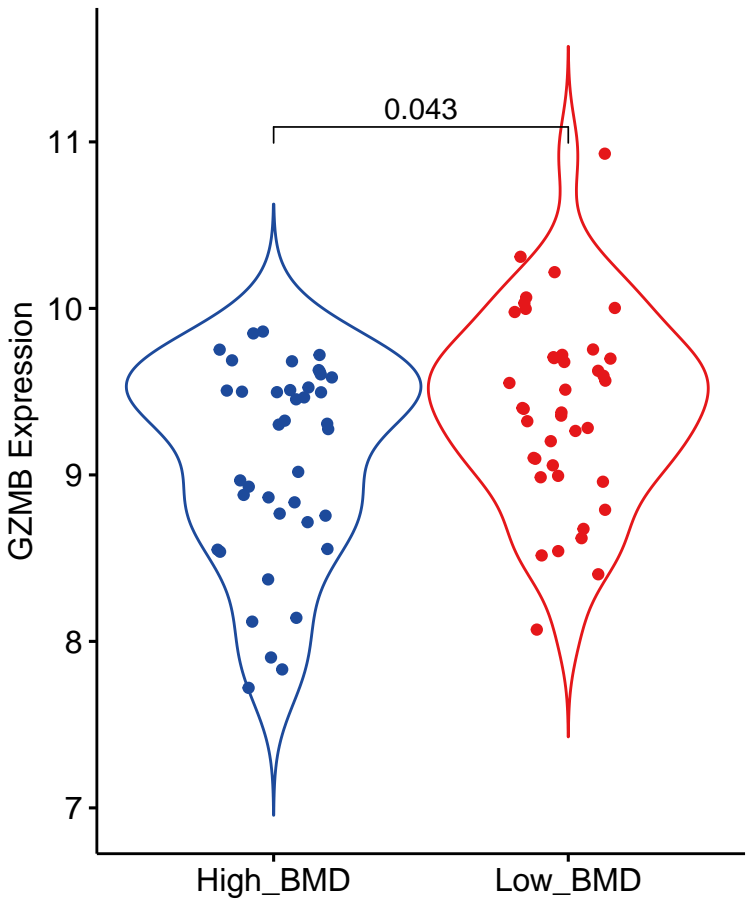

Supplement: Supplementary file 1 [file Supplementaryfile1.zip › Supplementary Material/03_ML/5.3_OP_Train_GZMB.pdf]

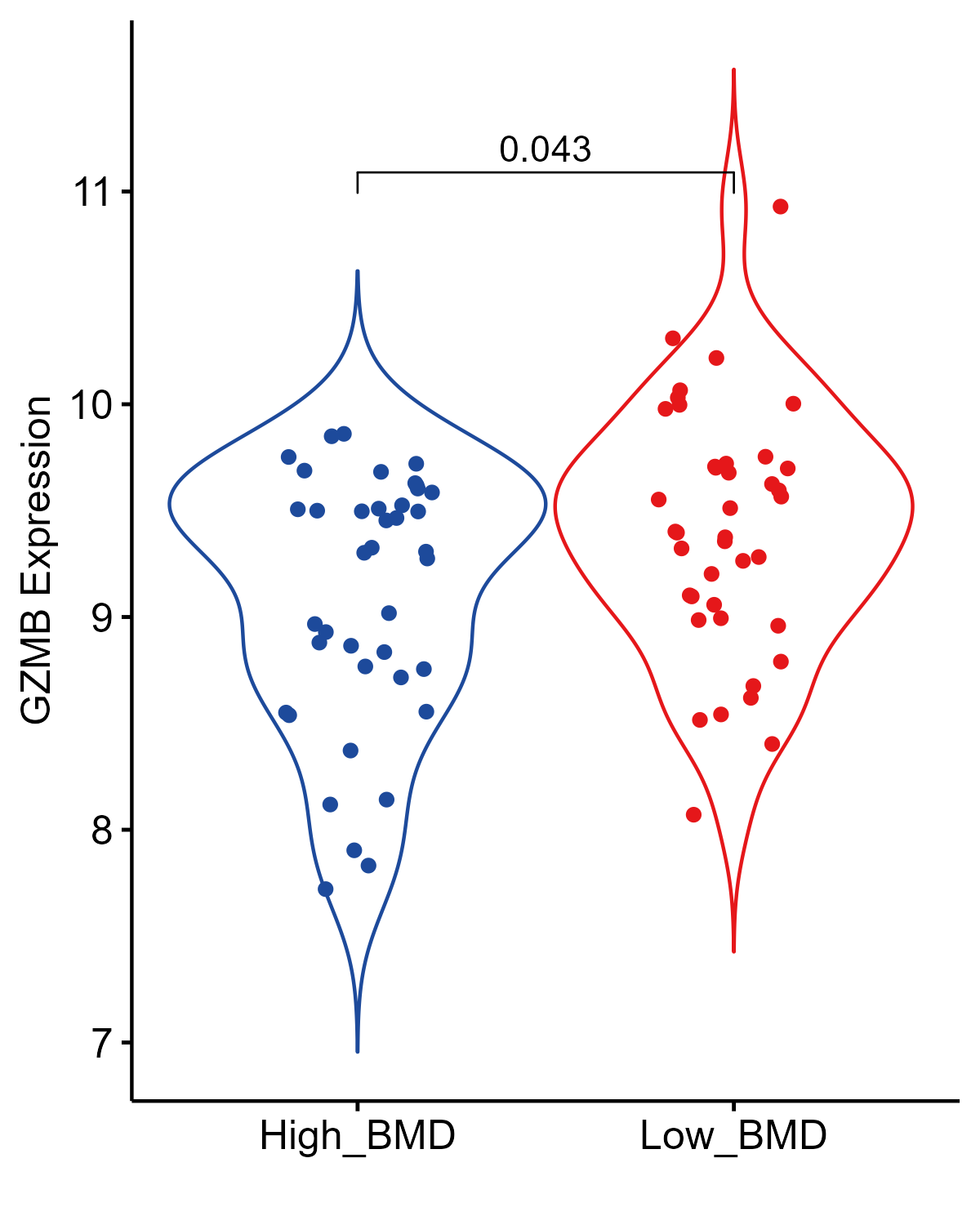

Supplement: Supplementary file 1 [file Supplementaryfile1.zip › Supplementary Material/03_ML/5.3_OP_Train_GZMB.png]
