# Supplementary material for: Identification of shared diagnostic genes between osteoporosis and Crohn’s disease through integrated transcriptomic analysis and machine learning
Source: Front Genet. 2025 Oct 7;16:1609915. doi: 10.3389/fgene.2025.1609915 (PMC12538133; doi:10.3389/fgene.2025.1609915)
Supplement: Supplementary file 1 [file 04_Cluster_dendrogram.pdf › Supplementary Material/02_WGCNA/4.1_WGCNA_OP_GSE56815_bp/04_Cluster_dendrogram.pdf]

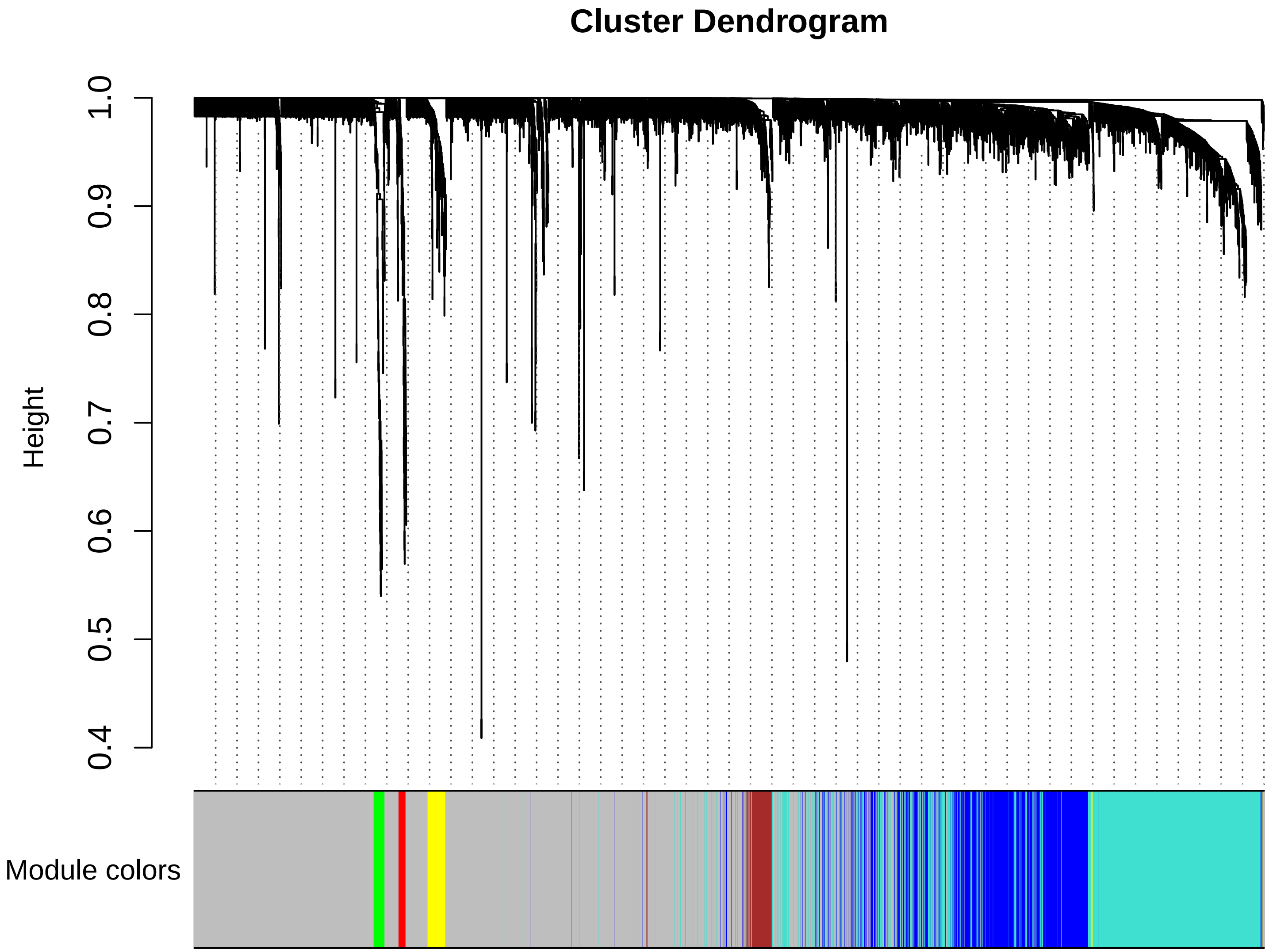
Cluster Dendrogram
Height
1.0
0.9
0.8
0.7
0.6
0.5
0.4
Module colors
